# Study on the region-specific expression of epididymis mRNA in the rams

**DOI:** 10.1371/journal.pone.0245933

**Published:** 2021-01-25

**Authors:** Cuiling Wu, Chunxin Wang, Bo Zhai, Yunhui Zhao, Zhuo Zhao, Zhiyu Yuan, Xuefeng Fu, Mingxin Zhang

**Affiliations:** 1 College of Animal Science, Xinjiang Agricultural University, Urumqi, China; 2 Branch of Animal Husbandry, Jilin Academy of Agricultural Sciences, Gongzhuling, China; 3 Key Laboratory of Genetics Breeding and Reproduction of Xinjiang Wool Sheep & Cashmere-Goat, Institute of Animal Science, Xinjiang Academy of Animal Sciences, Urumqi, China; Universite Clermont Auvergne, FRANCE

## Abstract

The epididymis is divided into three regions including the caput, corpus and cauda. Gene expression profiles in different regions indicate the different functions of epididymis which are crucial for sperm maturation. In this study, three one-year-old rams was used as the experimental animal. Transcriptome sequencing technology was used to sequence mRNA in the caput, corpus and cauda of the epididymis. Based on the spatiotemporal-specific expression pattern in the epididymis, the mRNA expression profiles of the three parts of the epididymis were analysed. Region-specifically expressed genes were analysed by GO and KEGG analyses to screen the key genes involved in sheep sperm maturation. We obtained 129, 54 and 99 specifically expressed genes in the caput, corpus and cauda, respectively. And twenty specific expressed genes related to sperm maturation were used to construct functional networks. The heatmap showed that 6 genes of LCN protein family were highly expressed in the head of epididymis of sheep. We infer that sperm maturation is gradual in the epididymis and that there are significant differences in epididymal gene expression patterns between different species. This provides a data resource for analysing the regulatory mechanism of epididymis genes related to sperm maturation in rams.

## Introduction

In the rams, excellent semen quality is a prerequisite for ideal fecundity and good production performance. With the development of the sheep industry and the wide application of reproductive biotechnology, semen freezing technology and artificial insemination technology have become an important way to promote sheep breeding and improve sheep farming efficiency [1]. In the cryopreservation of semen, a high yield and high quality of semen are necessary. The epididymis plays an important role in sperm maturation, transportation and storage and secretes epididymis fluid, which contains hormones, enzymes and special nutrients that are conducive to sperm maturation [2–4].

High-throughput microarray studies in the human [5], rat [6], mouse [7] and boar [8] have confirmed that the epididymis is a highly regionalized organ. The epididymis of most mammals is mainly divided into three regions: caput, corpus, and cauda. A deeper analysis was done for other mammalian species for which the anatomy of the epididymis allows the discrimination of 10 regions in the mouse, 19 in the rat, and 10 in the boar. It is now well accepted that the sperm maturational changes in the epididymis are mainly caused by the interaction with proteins secreted by epididymal epithelium. Several hundred epididymal proteins, such as cholesterol transfer proteins, glutathione peroxidases, were identified by electrophoresis in the early years [9].

The application of transcriptome methods has provided a dramatic advance in the study of epididymal physiology. With the continuous identification of various epididymal proteins, the protein changes during sperm maturation, including protein addition or removal, exposure or covering, are gradually revealed [10, 11]. The epididymal proteins involved in sperm maturation in recent years include ribonucleases, β-galactosidases, lipid carrier proteins and G-protein coupled receptors. It is worth noting that genes with region-specific expression patterns exist in epididymis, which are regulated by androgens and testicular factors to ensure different functions in the caput, corpus and cauda of the epididymis [12–14]. However, protein and gene expression levels are different in the epididymis of different species [15–17]. The establishment and maintenance of such strict regionalization mechanisms is also unknown. Therefore, we used transcriptome sequencing to analyse mRNA expression profiles in the caput, corpus and cauda of the epididymis in the rams. The results of this study are very important for analysing the regulatory mechanism of epididymal sperm maturation in males to improve semen quality and thus improve the reproductive performance of males.

## Materials and methods

### Animals and epididymis sample collection

This study was carried out in strict accordance with relevant guidelines and regulations by the Ministry of Agriculture of the People’s Republic of China. The Animal Ethics Committee approved the protocol of this study of Jilin Academy of Agricultural Sciences (AWEC2017A01, 9 March 2017).

Rams (Xinji fine wool sheep, n = 3) aged 12 months were sampled from the Branch of Animal Husbandry, Jilin Academy of Agricultural Sciences in Gongzhuling county, Jilin province of China. The experimental sheep were all reared under the same conditions, with fresh and dry grass as the main feed, supplemented with mixed concentrate, natural light and free drinking water. Before the operation, the weight difference was no more than 5 kg (S1 Table). Epididymal tissue was surgically collected and washed with PBS containing 1% Penicillin-Streptomycin (Gibco, USA). The sperm cells were not removed from the epididymis tissue. The each region (caput, corpus and cauda) of epididymides was cut into small pieces of about 2 cm as in the previous studies, which were quickly loaded into liquid nitrogen for mRNA sequencing and later validation [3].

### RNA extraction and sequencing

TRIzol (Life Technologies, USA)was used to extract total RNA from the caput, cauda and corpus of the epididymis, and an Agilent 2100 bioanalyzer (Agilent, USA) was used to determine the total RNA concentration, 28S/18S levels, and RNA integrity number (RIN). Total RNA was treated by mRNA enrichment. The 1 mg RNA per sample was used as input material for the RNA sample preparations. A CDNA library was constructed with mRNA as a template following manufacturer's recommendations (S1 Fig). Finally, transcriptome sequencing was performed on the BGISEQ-500 platform. (Shenzhen Huada Gene Technology Co., Ltd.)

### Quality control, annotation, and expression levels

We removed reads containing adapters, reads containing poly-N sequences and low-quality reads from the raw data with Trimmomatic (v0.36), and clean reads were obtained. Bowtie2 (v2.2.5) was used to compare clean reads to the reference gene sequence (Oar_v4.0), and RSEM (v1.2.8) was then used to calculate the expression levels of genes and transcripts. The Pearson correlation coefficient between each pair of samples was calculated using the “cor” function in R software.

### Analysis of differentially expressed genes

The DEGseq method [18] was used to calculate the differentially expressed genes (DEGs) among different samples. The genes that were up-regulated in pare comparisons among the three groups were taken as the highly expression genes (HEGs). We treated genes expressed only in one group and not in the other two groups as specifically expressed genes (SEGs). The phyper function in R software (https://en.wikipedia.org/wiki/Hypergeometric_distribution) was used to analyse the Gene Ontology (GO) and the Kyoto Encyclopedia of Genes and Genomes (KEGG) pathway enrichment of the DEGs. The significant levels of terms and pathways were correctetd by Q value with a rigorous threshold (Q value ≤ 0.05) by Bonferroni [19].

### Quantitative real-time PCR (qPCR) validation

To verify the RNA-seq results, we randomly selected eight genes from the DEGs and detected the expression level of each gene by quantitative real-time PCR (qPCR) on the same samples. The GAPDH gene was used as an endogenous control. Primer 5.0 software is used to design primers of mRNAs. First-strand cDNA was synthesized with a Transcriptor cDNA Synthesis Kit 2 (Roche, Germany) of 500ng RNA from each sample, and RT-qPCR was performed using the T2×RealStar Green Fast Mixture (Genstar, China). The amplification conditions were 95°C for 3 min, followed by 40 PCR cycles of 95°C for 10 s, 60°C for 15 s, and 72°C for 30 s, performed in a Light Cycler® 480 System (Roche, USA). Nine samples (caput, n = 3; corpus, n = 3; cauda, n = 3) were analyzed for each gene with three replicates. Data analysis using the 2−ΔΔCt method, and Microsoft Excel 2016 is used to generate line diagrams to compare RT-qPCR and RNA-seq results.

## Results

### Identification of mRNAs

The RNA concentration, RIN and A260/A280 quotient were shown in S2 Table. All nine samples can be used to construct the cDNA library. Nine samples were evaluated on the BGISEQ-500 platform, and each sample produced 6.51 GB of data on average. The raw data has been deposited in the Sequence Read Archive (SRA) database (BioProject ID: PRJNA660552).

To ensure the reliability of the results, the reads containing connectors were removed from the original sequencing data. Reads with more than 5% unknown bases (N) and low-quality reads were removed (reads with a mass value less than ten accounting for more than 20% of the total base number were defined as low-quality reads) (S4 Table). Subsequently, we classified and mapped the clean reads to the sheep reference genome assembly (Oar_v4.0). The average mapping ratio of the samples to genomes was 89.21% (S5 Table). We identified 16,809 transcripts, 18,939 known genes, 2,055 novel genes and 2,089 transcripts of novel coding proteins. The similarity of overall gene expression among the samples is shown in S1 Fig.

### Differentially expressed mRNA analysis

**Fig 1:**
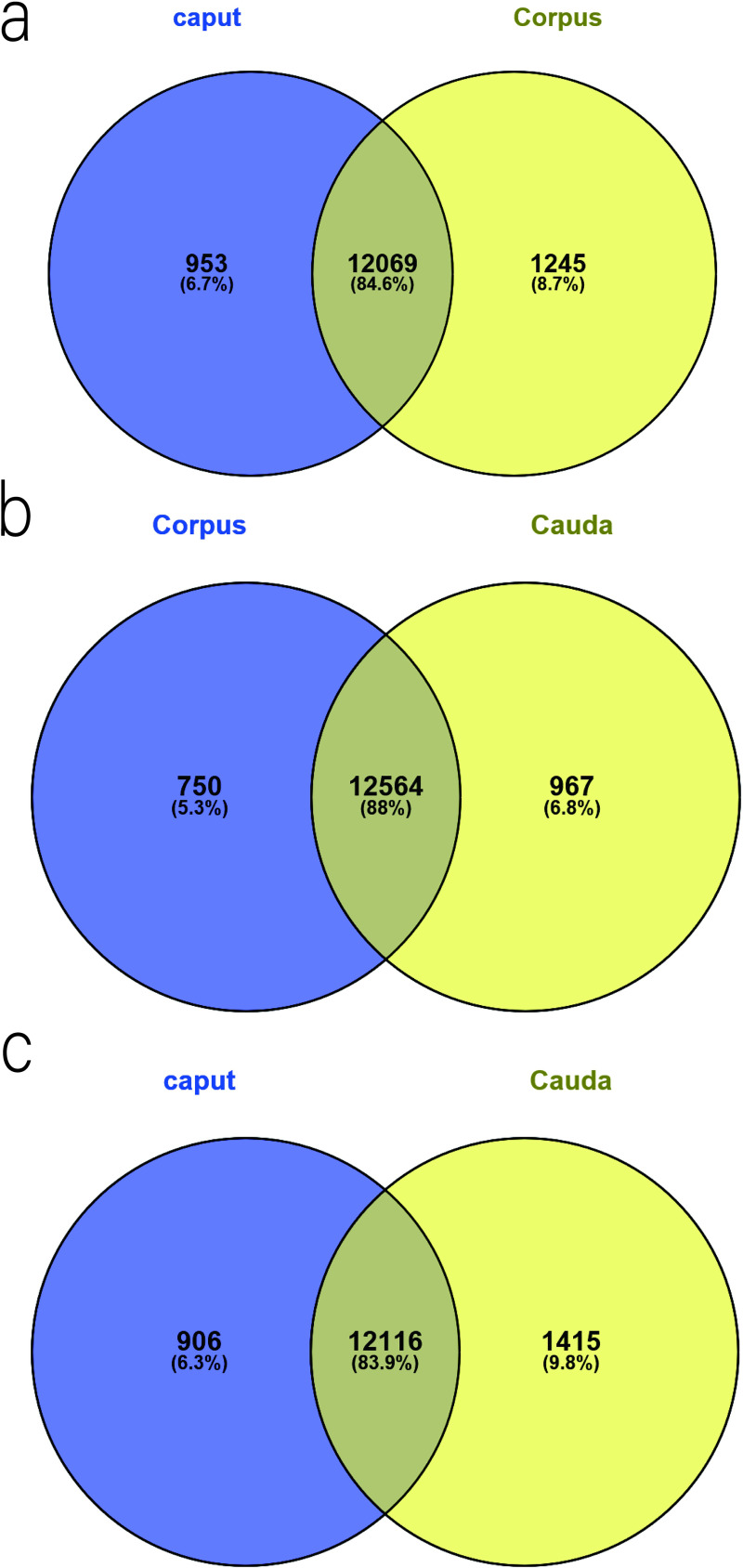
Venn diagram summarizing the number of DEGs in three regions of epididymis. (a) Caput vs Corpus. (b) Cauda vs Corpus. (c) Caput vs Cauda.

**Fig 2:**
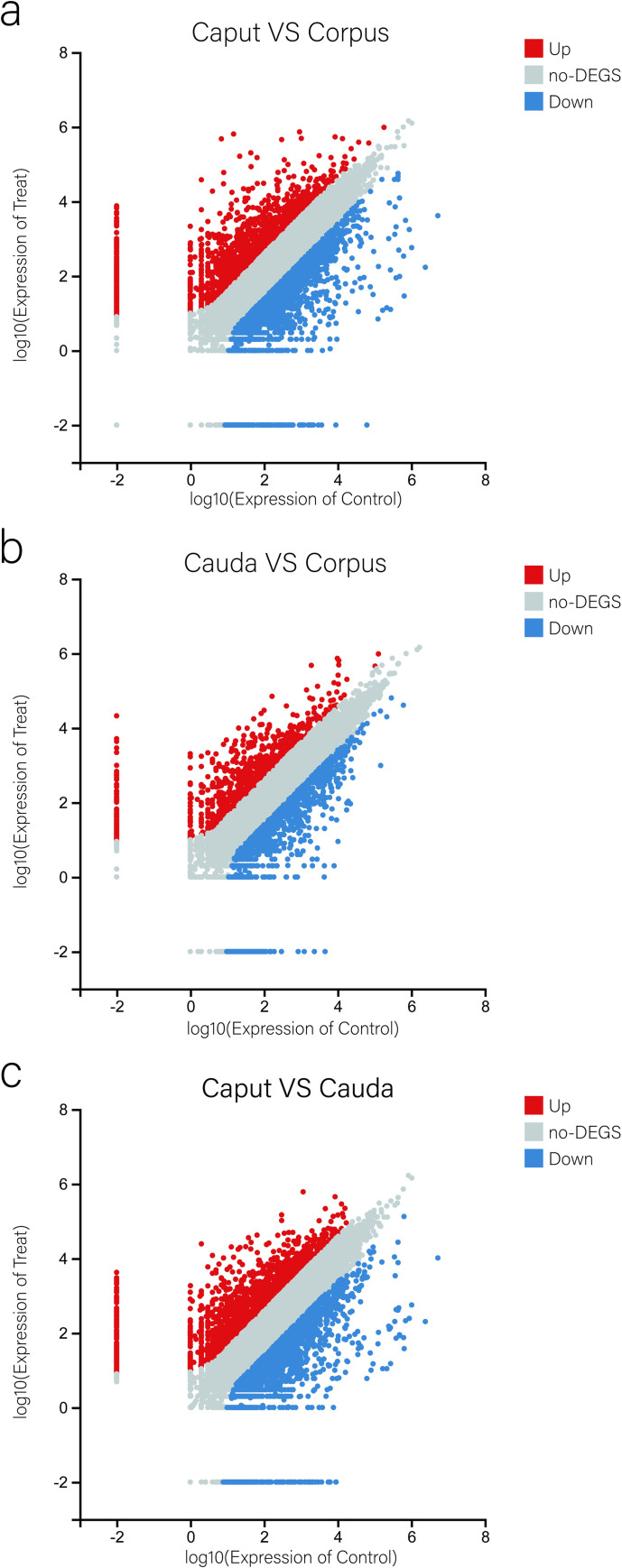
Scatter plot analysis of DEGs in three regions of epididymis. (a) Caput vs Corpus. (b) Cauda vs Corpus. (c) Caput vs Cauda. Gray points indicate no significant differential expression genes (no-DEGs). Red points and blue points represent upregulated and downregulated genes, respectively.

**Fig 3:**
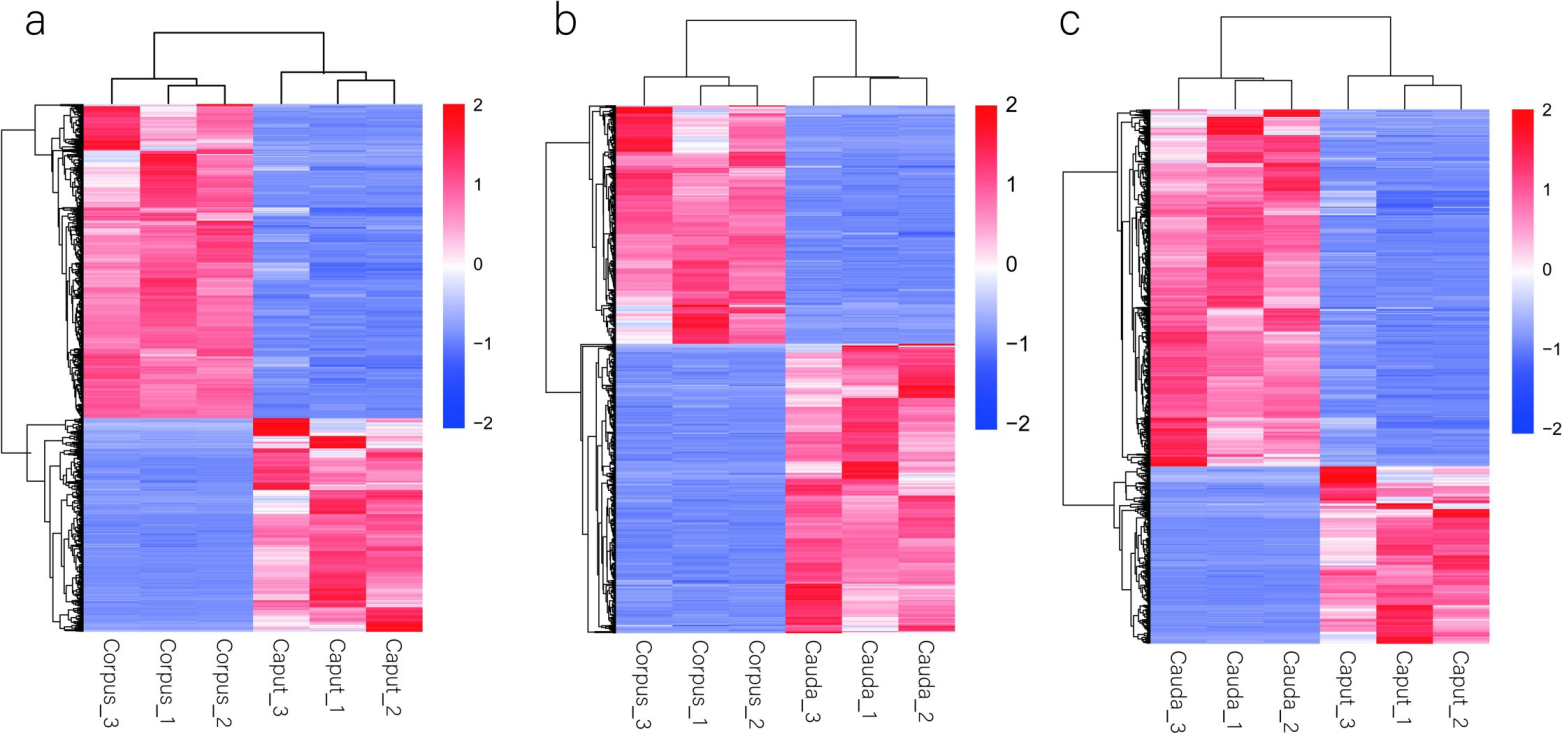
Heat map showing the hierarchical clustering of DEGs in three regions of epididymis. (a) Caput vs Corpus. (b) Cauda vs Corpus. (c) Caput vs Cauda. upregulated and downregulated genes are indicated in red and blue, respectively.

We considered genes whose mRNAs were not expressed with FPKM < 1 and genes exhibiting an expression difference of more than two-fold and a Q value ≤0.001 to be significantly differentially expressed. After the further deletion of discredited data, we obtained 1321 DEGs between the caput and corpus (535 genes more highly expressed in caput and 786 genes in corpus). Similarly, there were 855 DEGs between the corpus and the cauda (386 genes more highly expressed in corpus and 469 genes in cauda). A total of 1564 DEGs were identified between the caput and cauda (519 genes more highly expressed in caput and 1045 genes in cauda) (Figs 1–3, S7 and S8 Tables).

**Fig 4:**
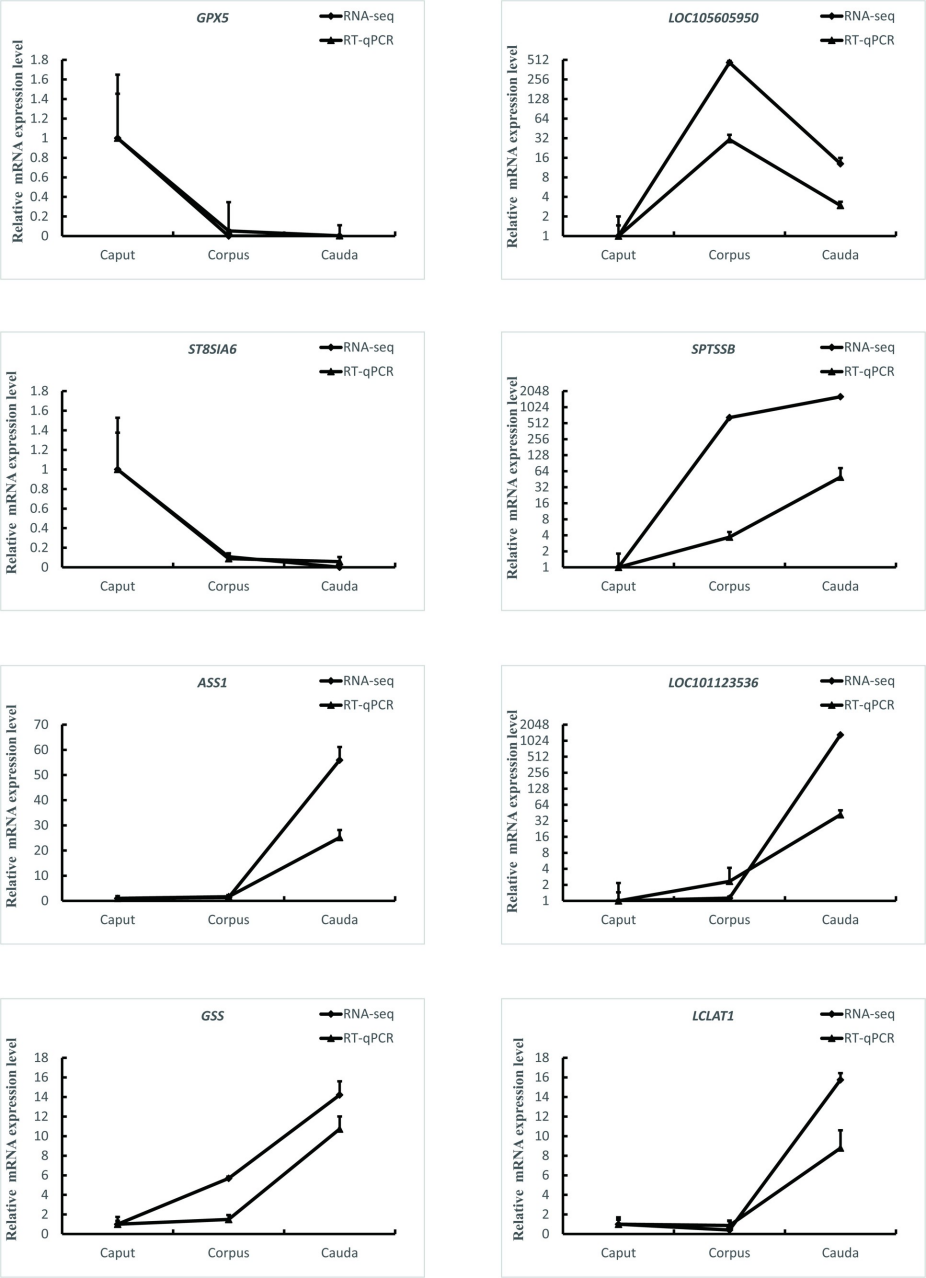
QPCR validation of RNA-seq. Eight DEGs were selected for validation, including GPX5, LOC105605950, ST8SIA6, SPTSSB, ASS1, LOC101123536, GSS, LCLAT1. The x-axis indicates the different parts of the epididymis, and the y-axis indicates the mRNA relative expression level (mean ± SEM).

To validate the RNA-seq results, we determined gene expression levels through qPCR analysis via the 2−ΔΔCt method. The sequences of primers was listed in S3 Table. The results of the comparison between the qPCR and RNA sequencing results are presented in Fig 4, showing that all selected DEGs presented similar expression patterns.

### Region-specific expression gene analysis

**Fig 5:**
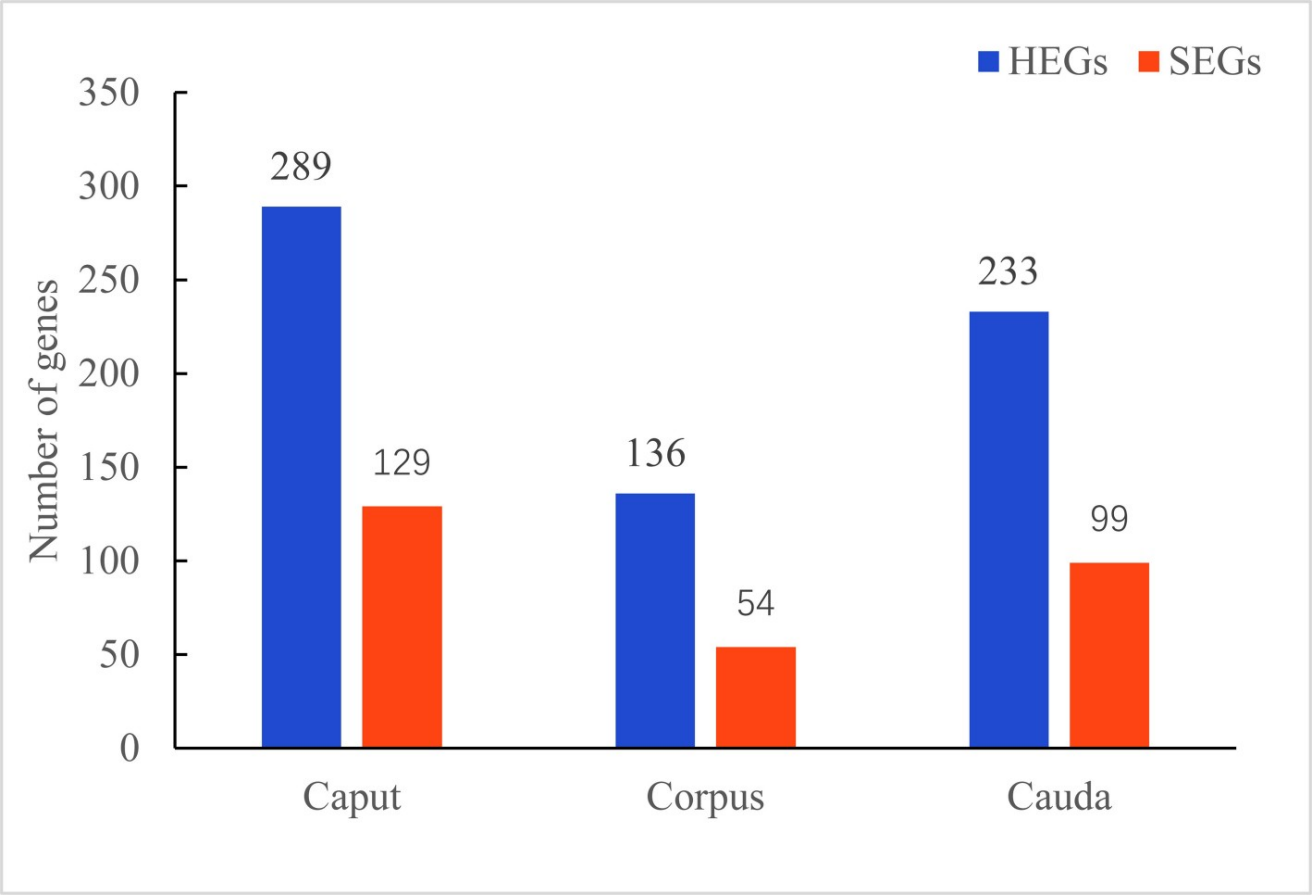
Number of genes showing epididymis-specific expression. HEGs indicate highly expressed genes that are upregulated compared to the other two regions of the epididymis. SEGs indicate specifically expressed genes that are not expressed with FPKM<1 in the other two regions of the epididymis.

After further screening of DEGs, we obtained 289 HEGs and 129 SEGs in the caput of epididymis, 136 HEGs and 54 SEGs in the corpus, and 233 HEGs and 99 SEGs in the cauda. (Fig 5, S9–S12 Tables).

**Fig 6:**
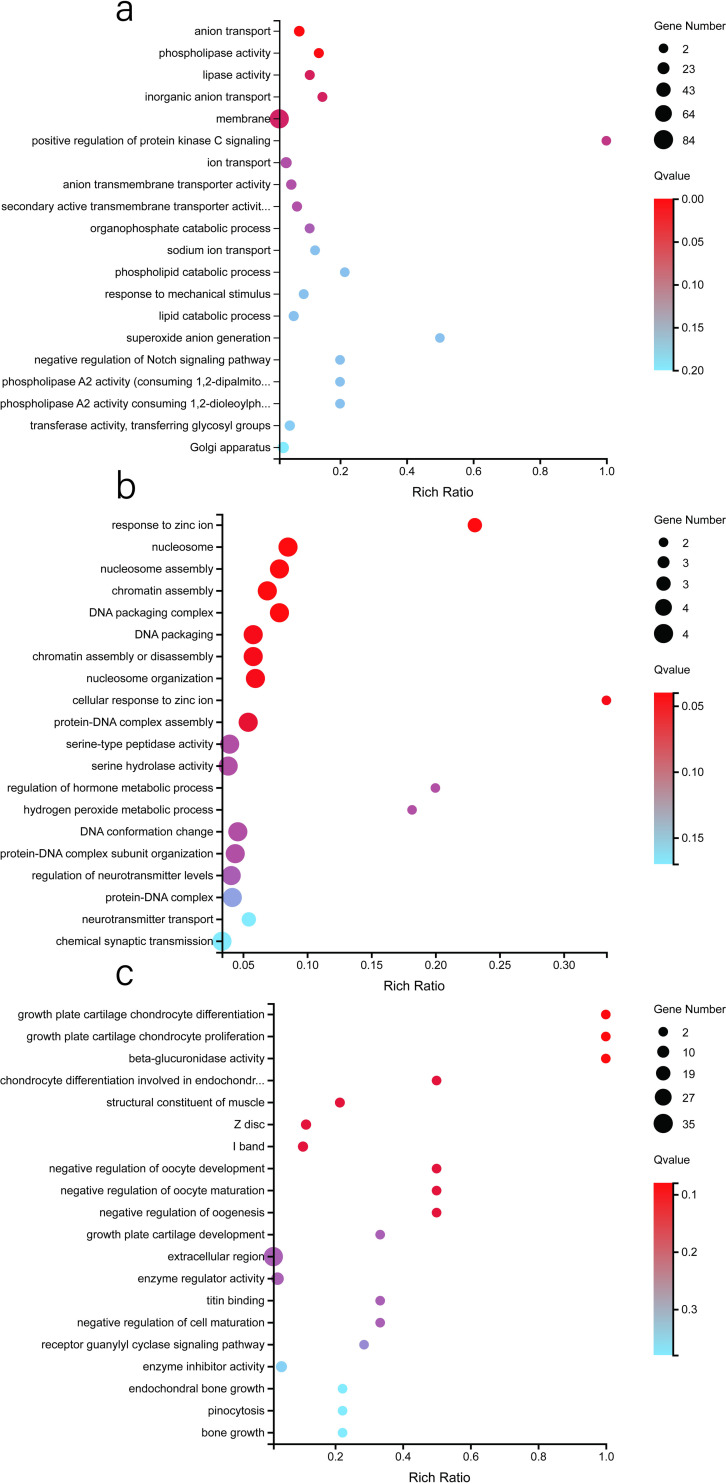
GO analysis bubble diagram of the HEGs in the epididymis. (a). HEGs in the caput. (b). HEGs in the corpus. (c). HEGs in the cauda. The X-axis represents the enrichment ratio (Rich Ratio = Term candidate gene num/Term gene num). The Y-axis represents the GO terms. The size of the bubble represents the number of genes annotated to a GO term, the colour represents the enrichment Q value, where the darker the colour, the smaller the Q value is. The bubble graph shows the top 20 GO terms with the smallest Q values.

**Fig 7:**
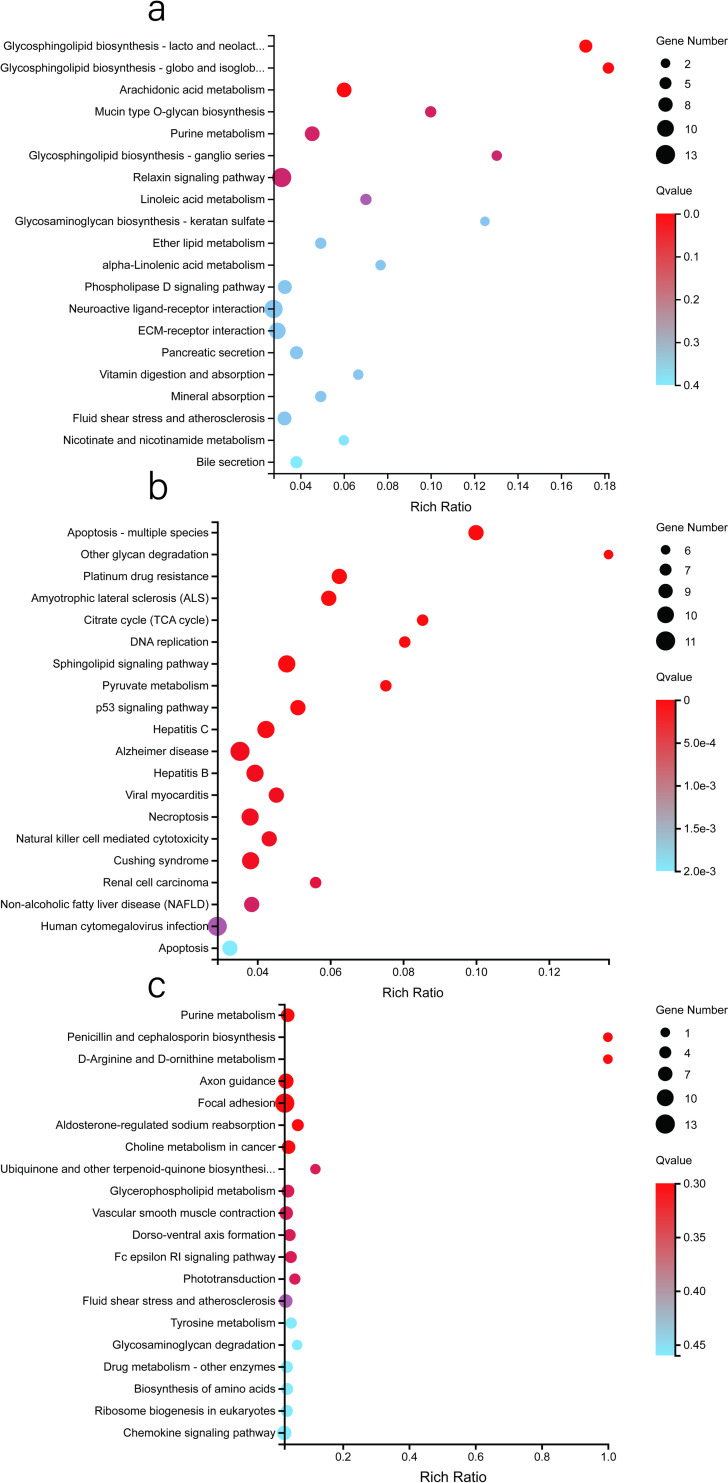
KEGG analysis bubble diagram of the HEGs in the epididymis. (a). HEGs in the caput. (b). HEGs in the corpus. (c). HEGs in the cauda. The X-axis represents the enrichment ratio (Rich ratio = Term candidate gene num/Term gene num). The Y-axis represents the KEGG pathways. The size of the bubble represents the number of genes annotated to a KEGG pathway, the colour represents the enrichment Q value, where the darker the colour, the smaller the Q value is. The bubble graph shows the top 20 GO terms with the smallest Q values.

**Fig 8:**
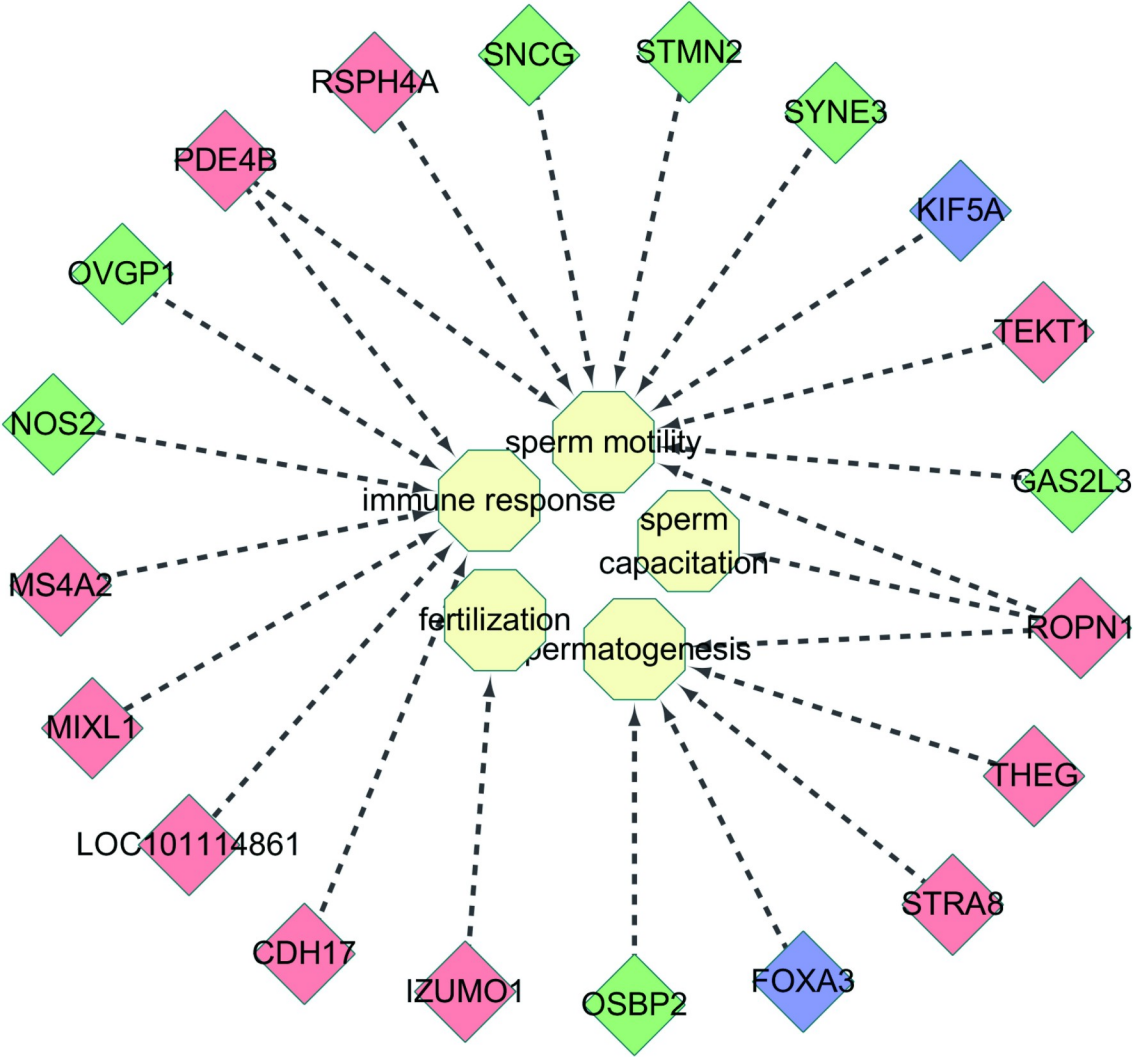
Functional annotation of 20 HEGs. Red, blue, and green represent genes with highly specific expression in the caput, corpus, and cauda of the epididymis, respectively. Yellow indicates sperm maturation-related functions.

According to the GO functional annotation, these HEGs were mainly annotated to the response to stimulus, membrane, binding, membrane part and catalytic activity categories (S2 Fig, Fig 6). According to the KEGG pathway annotation classification and enrichment analysis, these HEGs were mainly related to purine metabolism, protein digestion and absorption, global and overview maps and the biosynthesis of amino acids (S3 Fig, Fig 7). It is worth noting that we found that 20 HEGs were directly annotated to sperm development, immune response, fertilization, sperm movement, and capacitation functions (Fig 8).

### Analysis of gene families associated with sperm maturation

**Fig 9:**
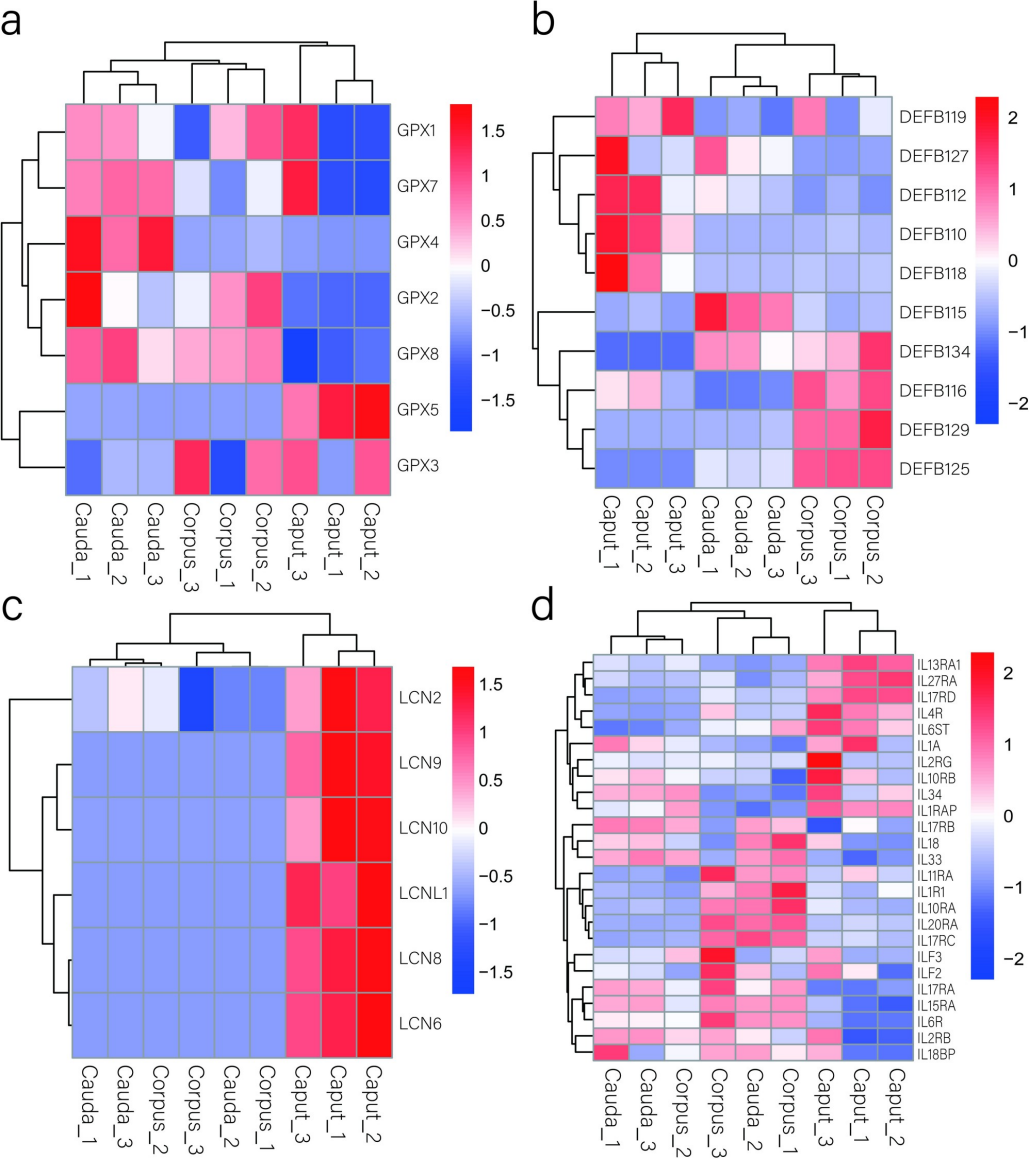
Heat maps of gene family expression profiles in the epididymis. (a). Glutathione peroxidase family. (b). Beta-defensin family. (c). Lipocalin protein family. (d). Interleukin-related genes. Upregulated and downregulated genes are indicated in red and blue, respectively.

We analysed the expression profiles of genes encoding four kinds of sperm maturity-related protein families (Fig 9). Seven genes of the glutathione peroxidase family (GPXs) were found to be expressed in the epididymis of the rams. GPXs have antioxidant functions, and different GPXs exhibit their own unique biological functions. The GPX5 gene showed the highest expression in the caput of the epididymis. The beta-defensin (DEFB) family includes ten genes expressed in the epididymis. Interestingly, the six genes of the lipocalin (LCN) protein family are highly expressed in the caput of the epididymis. In addition, interleukin (IL) plays an important role in a series of processes, such as the maturation, activation, proliferation and immune regulation of immune cells. Twenty-five interleukin-related genes were found to be expressed in the caput, corpus and cauda of the epididymis. IL13RA1 was the most highly expressed gene in the caput. The ILF2 gene presented the highest expression in the corpus and cauda.

## Discussion

Sperm in the epididymis can achieve motility, sperm-egg recognition ability and fertilization ability, indicating the role of the epididymis in sperm maturation [20]. Each species appears to have developed its own strategy for sperm maturation. However, there is a need to identify more specific expression gene in order to obtain greater understanding of sperm mature. In this study, we mainly identified genes specifically expressed in different regions of epididymis in sheep. Overall, the caput expressed the most SEGs and the corpus the fewest.

We mainly performed functional annotation and pathway analysis on the identified specific expressed genes. Studies have shown that changes in the Ca2+ concentration from the caput to the cauda of the epididymis affect almost every step of sperm maturation, especially sperm motility [21, 22]. According to our results, 35 genes involved in ion metabolism were specifically expressed in the caput of the epididymis, 11 in the corpus and 22 in the cauda (S13 Table). The speed and modes of sperm motility are two parameters used to measure movement ability. There are differences in the occurrence and development of sperm motility in the epididymis between different species of animals [23].

Sperm fertilization mainly depends on sperm capacitation. An obvious change before and after sperm capacitation is the redistribution of proteins on the sperm membrane surface, where proteins with a high molecular weight are decreased, and proteins with a low molecular weight are increased [24–26]. Serine protease inhibitory (SERPIN) proteins protect sperm from premature capacitation in the epididymis [27–29]. A total of 8 genes encoding serine protease inhibitor proteins were identified according to their expression. Only the SERPINA1 gene was expressed in the corpus of the epididymis. SERPINA1 is a protein with a highly conserved structure. It can irreversibly inhibit a variety of serine endopeptidases [30]. In addition, RNase9 is one of the epididymis-specific proteins regulated by androgens that belongs to the RNaseA superfamily [31]. Its main function is to assist the epididymis in promoting sperm maturation. Human RNase9 is expressed throughout the epididymis [32]. Rat experiments showed that RNase9 mRNA was only present in the principal cells of the epididymis caput and corpus [33]. However, our experiments show that 13 RNaseA superfamily genes are expressed in the epididymis of the rams, among which the RNase9 gene is highly expressed in the corpus and cauda of the epididymis. RNase10 is highly and specifically expressed in the caput. It is the same for other species for which RNase10 have been identified, such as humans, mice and pigs [34–36].

Epididymis is an important immune organ in male animals. The blood-epididymal barrier (BEB) plays an important role in the establishment of the epididymal environment. The BEB prevents interactions between sperm autoantigens and the immune system. The secretion of cytokines provides a guarantee for the operation of the BEB [3, 20]. We analyzed the expression profiles of 25 interleukin-related genes in epididymis of sheep. According to Fig 9d, we found that some genes in the caput of epididymis had similar expression patterns. The expression levels of IL13RA1, IL4R, IL17RD and IL27RA in the caput of epididymis were higher than that in the corpus and cauda of epididymis, and the expression levels of IL15RA, IL6R and IL17RA were lower than that in the corpus and cauda of epididymis. Therefore, we speculated that the caput immune defense mechanism of epididymis in sheep might be different from the corpus and cauda of epididymis.

In addition, β-defensins are cationic antimicrobial peptides that are involved in maintaining environmental stability in the epididymis, play antibacterial and anti-inflammatory roles, and combine with sperm to regulate sperm maturation [37]. Guyonnet et al. [8] reported 5 β-defensins in the epididymis of adult boar, among which DEFB109 was only highly expressed in the corpus and cauda and not in the caput, which was consistent with our experimental results in the rams. Narciandi et al. [38] identified 19 β-defensins in bulls, including 7 genes that were expressed in the epididymis of the rams in our study: DEFB115, DEFB116, DEFB118, DEFB119, DEFB125, DEFB127, and DEFB129. However, their expression patterns were different in the caput and cauda of the epididymis. Zhang et al. [39] found 8 β-defensin genes in the epididymis of goats, among which DEFB110 and DEFB112 were also expressed in the epididymis of sheep, but the two genes are expressed differently in goats and sheep.

At present, some gene families, such as aquaporins and lipocalins, are considered to exhibit region-specific expression in the epididymis [15, 16, 40]. Our results indicate that the expression pattern of the lipocalin gene is worthy of attention. The lipocalin family plays a role in lipid transport and participates in many biological processes, including the immune response, sperm maturation and storage [41]. Thimon et al. [40] demonstrated the presence of 8 lipocalins in the human epididymis, among which only LCN2 was highly expressed in the caput, while LCN6, LCN8 and LCN10 were highly expressed in the corpus, and LCNL1 was expressed in different regions of the epididymis. Previous studies in goats and pigs have reported that the expression of LCN6 and LCN8 genes in the caput of epididymis was significantly higher than in the corpus and cauda of epididymis. This is consistent with the expression in sheep epididymis. However, the LCN2 gene was expressed in all regions of epididymis of goats, and the expression difference in each region was not significant [8, 42]. In addition, Thimon et al. [40] found 4 aquaporin (AQP) family genes in the human epididymis, including AQP1, AQP2, AQP9, and AQP11. In our experimental results, only AQP1, AQP7, AQP9 and AQP11 were found, and their expression patterns were different from those in the human epididymis. These results indicate that epididymal gene expression patterns differ among species.

## Conclusions

Many scholars agree that study of epididymal protein is the key to a comprehensive understanding of sperm maturation. The process of expression from DNA to mRNA to protein involves a set of elaborate expression regulation mechanisms. In this context, there have been few reports of the sequencing of the sheep epididymal transcriptome. Therefore, the analysis of the regional expression of epididymal genes needs to be paid more attention, which is very important for the establishment of the environment of sperm maturation. However, these regions express genes whose contribution to sperm maturation has not been addressed And we identified genes specifically expressed in different regions of epididymis in sheep. These data provide a useful resource for the analysis of sperm maturation in mammalian epididymis.

## Supporting information

S1 Checklist The ARRIVE guidelines 2.0: Author checklist. (PDF)

S1 Fig The correlation matrix heatmap of 9 samples. The X and Y axes represent each sample. The colour represents the correlation coefficient. Red colour intensity indicates increasing sample correlation. Blue colour intensity indicates decreasing sample correlation.
(DOCX)

S2 Fig GO classification of HEGs in the epididymis. (a). HEGs in the caput. (b). HEGs in the corpus. (c). HEGs in the cauda. The X-axis represents the number of genes annotated to GO entries, and the Y-axis represents the GO functional classification. The blue bar represents molecular functions, the red bar represents cellular components, and the green bar represents biological processes.
(DOCX)

S3 Fig KEGG classification of the HEGs in the epididymis. (a). HEGs in the caput. (b). HEGs in the corpus. (c). HEGs in the cauda. The X-axis represents the number of genes annotated to GO entries, and the Y-axis represents the KEGG pathway classification.
(DOCX)

S1 Table Measurement of growth traits and sperm motility in experimental sheep. (DOCX)

S2 Table Test results for total RNA. (DOCX)

S3 Table Primer sequence information. (DOCX)

S4 Table Summary of the transcriptome sequencing data. (DOCX)

S5 Table Summary of read numbers aligned onto the reference genome. (DOCX)

S6 Table The DEGs list between caput and corpus. (DOCX)

S7 Table The DEGs list between corpus and cauda. (DOCX)

S8 Table The DEGs list between caput and cauda. (DOCX)

S9 Table The SEGs list in different regions of epididymis. (DOCX)

S10 Table 289 highly expressed genes in the caput of the epididymis. (DOCX)

S11 Table 136 highly expressed genes in the corpus of the epididymis. (DOCX)

S12 Table 233 highly expressed genes in the cauda of the epididymis. (DOCX)

S13 Table Summary of SEGs annotated to ion metabolism in different regions of epididymis. (DOCX)
