# Supplementary material for: Study on the region-specific expression of epididymis mRNA in the rams
Source: PLoS One. 2021 Jan 25;16(1):e0245933. doi: 10.1371/journal.pone.0245933 (PMC7833257; doi:10.1371/journal.pone.0245933)
Supplement: S1 Fig — The X and Y axes represent each sample. The colour represents the correlation coefficient. Red colour intensity indicates increasing sample correlation. Blue colour intensity indicates decreasing sample correlation. (DOCX) [file pone.0245933.s002.docx]

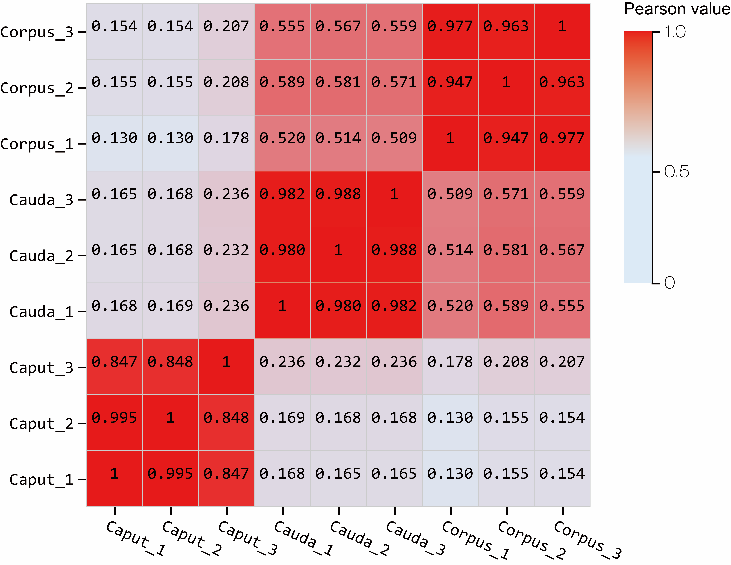


**S1 Fig**. **The correlation matrix heatmap of 9 samples.** The X and Y axes represent each sample. The colour represents the correlation coefficient. Red colour intensity indicates increasing sample correlation. Blue colour intensity indicates decreasing sample correlation.
