# Supplementary material for: Study on the region-specific expression of epididymis mRNA in the rams
Source: PLoS One. 2021 Jan 25;16(1):e0245933. doi: 10.1371/journal.pone.0245933 (PMC7833257; doi:10.1371/journal.pone.0245933)
Supplement: S2 Fig — (a). HEGs in the caput. (b). HEGs in the corpus. (c). HEGs in the cauda. The X-axis represents the number of genes annotated to GO entries, and the Y-axis represents the GO functional classification. The blue bar represents molecular functions, the red bar represents cellular components, and the green bar represents biological processes. (DOCX) [file pone.0245933.s003.docx]

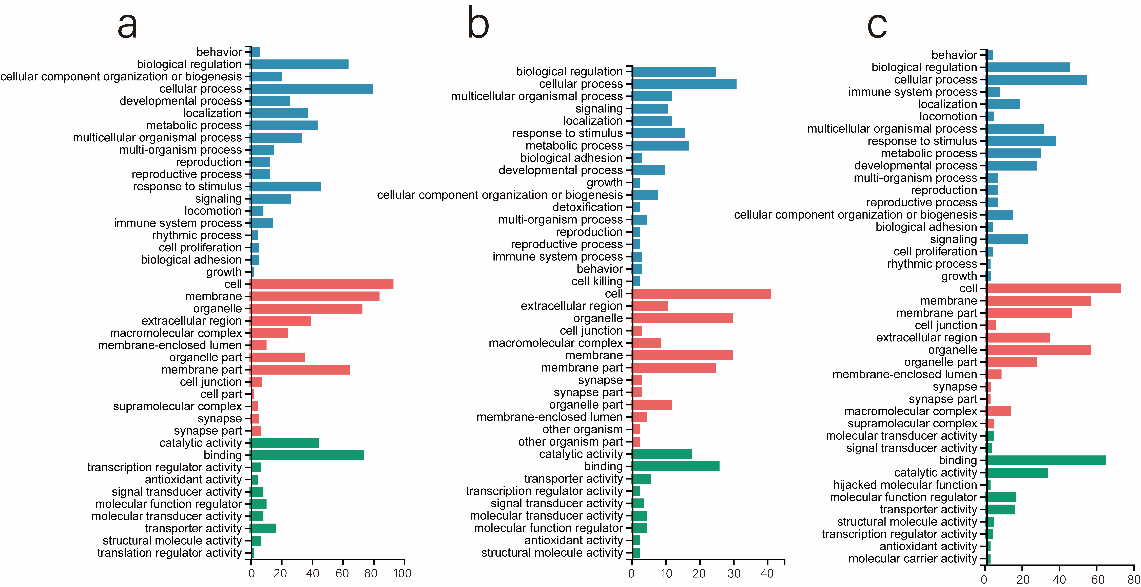


**S2 Fig. GO classiﬁcation of HEGs in the epididymis.** (a). HEGs in the caput. (b). HEGs in the corpus. (c). HEGs in the cauda. The X-axis represents the number of genes annotated to GO entries, and the Y-axis represents the GO functional classification. The blue bar represents molecular functions, the red bar represents cellular components, and the green bar represents biological processes.
