# Supplementary material for: Study on the region-specific expression of epididymis mRNA in the rams
Source: PLoS One. 2021 Jan 25;16(1):e0245933. doi: 10.1371/journal.pone.0245933 (PMC7833257; doi:10.1371/journal.pone.0245933)
Supplement: S1 Table — (DOCX) [file pone.0245933.s005.docx]

# S1 Table. Measurement of growth traits and sperm motility in experimental sheep

| **Individual** | **Body Weight（kg)** | **Body Height (cm)** | **Body Length (cm)** | **Bust(cm)** |
| --- | --- | --- | --- | --- |
| 2036 | 63.5 | 73 | 85 | 98 |
| 2194 | 62.8 | 71 | 88 | 117 |
| 2028 | 60.4 | 64 | 85 | 110 |
