# Supplementary material for: Study on the region-specific expression of epididymis mRNA in the rams
Source: PLoS One. 2021 Jan 25;16(1):e0245933. doi: 10.1371/journal.pone.0245933 (PMC7833257; doi:10.1371/journal.pone.0245933)
Supplement: S2 Table — (DOCX) [file pone.0245933.s006.docx]

# S2 Table. Test results for total RNA

| **Sample** | **Concentration（ng/ul）** | **28S/18S** | **RIN** |
| --- | --- | --- | --- |
| Caput_1 | 880 | 1.5 | 8.7 |
| Caput_2 | 996 | 1.6 | 8.4 |
| Caput_3 | 561 | 1.5 | 8.1 |
| Corpus_1 | 368 | 1.5 | 8.9 |
| Corpus_2 | 582 | 1.6 | 9.2 |
| Corpus_3 | 555 | 1.7 | 9.1 |
| Cauda_1 | 725 | 1.5 | 8.8 |
| Cauda_2 | 528 | 1.5 | 8.3 |
| Cauda_3 | 528 | 1.5 | 8.8 |
