# Supplementary material for: Study on the region-specific expression of epididymis mRNA in the rams
Source: PLoS One. 2021 Jan 25;16(1):e0245933. doi: 10.1371/journal.pone.0245933 (PMC7833257; doi:10.1371/journal.pone.0245933)
Supplement: S3 Table — (DOCX) [file pone.0245933.s007.docx]

# S3 Table. Primer sequence information

| **Gene** | **Primer sequence (5‘-3‘)** | **Annealing temp (℃)** | **Product size /pb** |
| --- | --- | --- | --- |
| GAPDH | F:CAAGTTCCACGGCACAGTCA  R:GGTTCACGCCCATCACAAA | 60 | 248 |
| GPX5 | F: TTGACAATGCGGTGAATAGCC  R: GGGTGGGGTGTCATGGAAAA | 60 | 178 |
| LOC105605950 | F: CATCCTGAGGACAAGCACCA  R: TGGTGTTGCTCTTGTAGTCCC | 60 | 149 |
| ST8SIA6 | F: CCTGATGGAGCGATGCCAAA  R: CTTGTCGTTTCCATGGGCAG | 60 | 128 |
| SPTSSB | F: TGAGGGTCCCTGACATGCTA  R: AGCGCTGCATAAGCAGTAGA | 60 | 128 |
| ASS1 | F: GCCTCACTTTACCCATCTCCC  R: GGCTACAGGGGACTATTGGA | 60 | 132 |
| LOC101123536 | F: TACGTGGTGCATAGCCTGTC  R: TCCATCTCCCCTGCAGACTT | 60 | 98 |
| GSS | F: TGATGAACAAGCACGTGGGA  R: ACCGGACAAAGGATGGAAGG | 60 | 163 |
| LCLAT1 | F: TTCAACACTGTGTAGCAGGGAG  R: GAAGCAGGACTGGACCTGTA | 60 | 117 |
