# Supplementary material for: Study on the region-specific expression of epididymis mRNA in the rams
Source: PLoS One. 2021 Jan 25;16(1):e0245933. doi: 10.1371/journal.pone.0245933 (PMC7833257; doi:10.1371/journal.pone.0245933)
Supplement: S4 Table — (DOCX) [file pone.0245933.s008.docx]

# S4 Table. Summary of the transcriptome sequencing data.

| **Sample** | **Total Raw Reads (M)** | **Total Clean Reads (M)** | **Total Clean Bases(Gb)** | **Clean Reads Q20(%)** | **Clean Reads Q30(%)** | **Clean Reads Ratio(%)** |
| --- | --- | --- | --- | --- | --- | --- |
| Caput_1 | 64 | 62.01 | 6.2 | 97.6 | 90.27 | 96.89 |
| Caput_2 | 70.19 | 67.64 | 6.76 | 97.6 | 89.75 | 96.37 |
| Caput_3 | 68.82 | 66.02 | 6.6 | 97.42 | 89.61 | 95.93 |
| Cauda_1 | 69.32 | 66.56 | 6.66 | 97.53 | 89.93 | 96.03 |
| Cauda_2 | 67.68 | 63.93 | 6.39 | 96.97 | 88.38 | 94.46 |
| Cauda_3 | 70.19 | 65.97 | 6.6 | 96.85 | 88.15 | 94 |
| Corpus_1 | 64 | 61.81 | 6.18 | 97.72 | 90.1 | 96.57 |
| Corpus_2 | 72.69 | 65.4 | 6.54 | 97.44 | 89.28 | 89.97 |
| Corpus_3 | 69.77 | 66.97 | 6.7 | 97.53 | 89.75 | 95.99 |
