# Supplementary material for: Study on the region-specific expression of epididymis mRNA in the rams
Source: PLoS One. 2021 Jan 25;16(1):e0245933. doi: 10.1371/journal.pone.0245933 (PMC7833257; doi:10.1371/journal.pone.0245933)
Supplement: S5 Table — (DOCX) [file pone.0245933.s009.docx]

# S5 Table. Summary of read numbers aligned onto the reference genome.

| **Sample** | **Total Clean Reads (M)** | **Total Mapping(%)** | **Uniquely Mapping(%)** |
| --- | --- | --- | --- |
| Caput_1 | 62.01 | 73 | 60.34 |
| Caput_2 | 67.64 | 72.62 | 59.88 |
| Caput_3 | 66.02 | 73.92 | 60.37 |
| Cauda_1 | 66.56 | 70.97 | 56.46 |
| Cauda_2 | 63.93 | 70.44 | 56.77 |
| Cauda_3 | 65.97 | 71.7 | 58.4 |
| Corpus_1 | 61.81 | 73.39 | 58.62 |
| Corpus_2 | 65.4 | 74.16 | 58.56 |
| Corpus_3 | 66.97 | 73.12 | 59.24 |
