# Supplementary material for: Study on the region-specific expression of epididymis mRNA in the rams
Source: PLoS One. 2021 Jan 25;16(1):e0245933. doi: 10.1371/journal.pone.0245933 (PMC7833257; doi:10.1371/journal.pone.0245933)
Supplement: S6 Table — (DOCX) [file pone.0245933.s010.docx]

# S6 Table. The DEGs list between caput and corpus

| **Gene ID** | **Other Gene ID** | **log2(Caput/Corpus)** | **Pvalue(Caput-vs-Corpus)** | **Qvalue(Caput-vs-Corpus)** |
| --- | --- | --- | --- | --- |
| 442999 | AQP1 | 2.316764554 | 1.78E-125 | 2.25E-125 |
| 443019 | QPCT | -3.854554692 | 0 | 0 |
| 443049 | PDXK | 2.013891085 | 0 | 0 |
| 443066 | ESR2 | -5.147744501 | 0 | 0 |
| 443076 | MET | 2.687879397 | 0 | 0 |
| 443083 | IRF6 | -3.020542511 | 0 | 0 |
| 443098 | SLC17A5 | 2.86354627 | 0 | 0 |
| 443109 | TFAP2A | -2.237175996 | 0 | 0 |
| 443115 | MMP2 | 6.000796138 | 0 | 0 |
| 443154 | HYAL2 | 2.103217072 | 1.77E-307 | 4.21E-307 |
| 443168 | MT3 | 4.803656791 | 0.000128335 | 3.82E-05 |
| 443175 | BMP7 | -2.42494078 | 0 | 0 |
| 443184 | AQP9 | -2.90889755 | 0 | 0 |
| 443199 | TJP3 | -2.862029423 | 0 | 0 |
| 443203 | MS4A2 | 12.2191677 | 0 | 0 |
| 443221 | RNASE10 | 13.78017022 | 0 | 0 |
| 443232 | CD40 | 2.859420062 | 2.77E-35 | 1.67E-35 |
| 443246 | SLC1A3 | -2.644155745 | 5.56E-64 | 4.60E-64 |
| 443279 | CHI3L1 | 5.007435852 | 0 | 0 |
| 443282 | GGTA2P | 3.408072112 | 0 | 0 |
| 443326 | ALOX5AP | -3.758585634 | 0 | 0 |
| 443331 | TIMP1 | 5.399598101 | 0 | 0 |
| 443333 | GHR | -2.104989739 | 0 | 0 |
| 443343 | ALDH1A1 | -2.284742764 | 0 | 0 |
| 443361 | NPY1R | -5.908900377 | 0 | 0 |
| 443384 | ATP1B1 | 3.170969882 | 0 | 0 |
| 443390 | OXT | 2.814966541 | 1.96E-62 | 1.60E-62 |
| 443411 | LOC443411 | 5.107190313 | 0 | 0 |
| 443450 | BCAT2 | -3.840937305 | 0 | 0 |
| 443454 | BMPR1B | -3.916122825 | 0 | 0 |
| 443469 | IGFBP2 | 3.110386859 | 0 | 0 |
| 443505 | SLC34A1 | 9.449572503 | 0 | 0 |
| 443524 | INHBA | 4.062810857 | 0 | 0 |
| 494440 | SLC4A4 | 2.863135417 | 0 | 0 |
| 554321 | LTF | -3.481320714 | 0 | 0 |
| 554323 | TGFB3 | 3.772590593 | 0 | 0 |
| 554328 | PAX4 | -8.404577568 | 8.53E-36 | 5.18E-36 |
| 554335 | ACE | 3.673186859 | 0 | 0 |
| 641305 | PI3 | -2.440593953 | 5.90E-15 | 2.50E-15 |
| 654334 | SLCO2A1 | 4.683495633 | 0 | 0 |
| 678680 | LGMN | 3.846889309 | 0 | 0 |
| 780451 | CES5A | -10.55889785 | 0 | 0 |
| 780475 | CLDN2 | 6.215149386 | 0 | 0 |
| 780521 | LEF1 | 6.36101483 | 0 | 0 |
| 100036756 | BSP5 | -14.21865315 | 0 | 0 |
| 100037669 | NPC2 | -3.378214604 | 0 | 0 |
| 100037695 | TNFRSF11B | 3.790481402 | 8.73E-156 | 1.27E-155 |
| 100127208 | SCD5 | -2.113386891 | 0 | 0 |
| 100142674 | PRKAG3 | -4.742528074 | 0 | 0 |
| 100145856 | HEXA | 3.499758111 | 0 | 0 |
| 100145866 | VDAC1 | 3.062760523 | 0 | 0 |
| 100145875 | PSPH | 2.293041631 | 0 | 0 |
| 100147707 | MC4R | -3.090183785 | 0 | 0 |
| 100147780 | SLC25A33 | -2.108240611 | 1.77E-80 | 1.69E-80 |
| 100169699 | LIPE | -2.591759871 | 2.12E-135 | 2.82E-135 |
| 100170324 | DPP4 | -3.585898737 | 0 | 0 |
| 100174904 | GATM | 2.893095339 | 0 | 0 |
| 100187549 | HPCAL1 | 4.776153276 | 0 | 0 |
| 100192431 | FCGR1A | -6.551002031 | 0 | 0 |
| 100233167 | BSP1 | -10.4672225 | 1.49E-109 | 1.72E-109 |
| 100233168 | BSP5L | -13.89532427 | 0 | 0 |
| 100271925 | HEY2 | 7.06889288 | 0 | 0 |
| 100294558 | SLC26A2 | 2.351815622 | 0 | 0 |
| 100302352 | PRM1 | 3.437117809 | 1.17E-160 | 1.76E-160 |
| 100302354 | SDR16C5 | 7.136583884 | 0 | 0 |
| 100302705 | IZUMO1 | 6.755954834 | 0 | 0 |
| 100307035 | CAT | 2.957269442 | 0 | 0 |
| 100307044 | FHL1 | 2.406460218 | 0 | 0 |
| 100329218 | SREBF1 | -2.350223337 | 0 | 0 |
| 100379136 | DUOXA2 | -5.500884672 | 1.65E-129 | 2.12E-129 |
| 100422802 | PAX2 | -4.848903755 | 0 | 0 |
| 100462691 | CRISP3 | 4.681112878 | 0 | 0 |
| 100462743 | DUOX2 | -7.300541075 | 0 | 0 |
| 100528014 | MYOZ3 | 2.073725164 | 0 | 0 |
| 100568285 | CYP2E1 | 9.453682748 | 0 | 0 |
| 100642181 | SRD5A1 | -2.840598183 | 0 | 0 |
| 100820737 | MFGE8 | -2.361997846 | 0 | 0 |
| 100820744 | ACP5 | -4.011872063 | 0 | 0 |
| 100885761 | WEE1 | -2.018142635 | 0 | 0 |
| 100913163 | GPX5 | 10.31291018 | 0 | 0 |
| 101101858 | HIVEP3 | 4.414798609 | 0 | 0 |
| 101101868 | UPF3B | -2.375648012 | 0 | 0 |
| 101101872 | CTNNAL1 | 2.305363788 | 1.61E-266 | 3.48E-266 |
| 101101881 | TST | 2.610828827 | 6.63E-144 | 9.17E-144 |
| 101101882 | HMCN2 | -3.422997606 | 0 | 0 |
| 101101956 | FGF13 | 3.304940796 | 0 | 0 |
| 101101969 | RASGRP3 | -3.090965269 | 0 | 0 |
| 101101974 | SH2D6 | -3.852285545 | 0 | 0 |
| 101101985 | NIPAL1 | -3.628715816 | 8.02E-181 | 1.30E-180 |
| 101102139 | FAM83F | 3.748469869 | 5.33E-161 | 7.98E-161 |
| 101102144 | PDLIM4 | 2.532585066 | 2.43E-176 | 3.86E-176 |
| 101102158 | MFSD6L | 3.391795612 | 5.10E-293 | 1.18E-292 |
| 101102179 | SPRY1 | 2.002001975 | 0 | 0 |
| 101102187 | PRSS23 | -2.005333655 | 0 | 0 |
| 101102231 | LOC101102231 | -3.258270959 | 1.14E-99 | 1.23E-99 |
| 101102245 | MTHFD1L | -2.292809685 | 0 | 0 |
| 101102316 | TMEM71 | -3.3545776 | 6.94E-280 | 1.55E-279 |
| 101102327 | LOC101102327 | -2.79971891 | 6.46E-282 | 1.45E-281 |
| 101102372 | PLCL1 | 3.397125509 | 9.76E-302 | 2.29E-301 |
| 101102433 | DUSP22 | -3.55994235 | 1.95E-196 | 3.33E-196 |
| 101102456 | SLC24A2 | 3.282240106 | 2.67E-123 | 3.34E-123 |
| 101102499 | KCTD12 | 4.325747895 | 0 | 0 |
| 101102548 | LOC101102548 | -5.656476928 | 0 | 0 |
| 101102594 | GMPR | 2.037493556 | 3.88E-74 | 3.52E-74 |
| 101102644 | GC | -8.451194678 | 0 | 0 |
| 101102692 | LAMA1 | -2.540282323 | 0 | 0 |
| 101102694 | PASK | 2.157891649 | 1.61E-271 | 3.51E-271 |
| 101102696 | QPRT | 5.433310782 | 3.48E-286 | 7.89E-286 |
| 101102761 | HAPLN3 | -2.700660044 | 2.96E-158 | 4.36E-158 |
| 101102868 | TMEM44 | 2.783835132 | 3.76E-261 | 7.98E-261 |
| 101102907 | RTN1 | 4.283144622 | 0 | 0 |
| 101102939 | CRISP1 | -5.376448322 | 0 | 0 |
| 101102970 | LCNL1 | 7.689719129 | 0 | 0 |
| 101103013 | BCL7A | 2.111577054 | 3.22E-111 | 3.76E-111 |
| 101103040 | FOXO4 | -2.057949194 | 0 | 0 |
| 101103123 | TRPM3 | -2.086675674 | 0 | 0 |
| 101103124 | BMX | -5.215235682 | 0 | 0 |
| 101103131 | ZNF275 | -2.48152061 | 0 | 0 |
| 101103164 | TEKT1 | 6.583266722 | 0 | 0 |
| 101103169 | FLVCR1 | 3.106614615 | 0 | 0 |
| 101103174 | LOC101103174 | -2.288830834 | 0 | 0 |
| 101103188 | TDRD9 | -4.358167924 | 0 | 0 |
| 101103205 | C1H2orf54 | 6.159532217 | 2.32E-203 | 4.05E-203 |
| 101103230 | GSAP | -2.080174789 | 1.34E-221 | 2.50E-221 |
| 101103244 | PREX2 | 2.81177324 | 0 | 0 |
| 101103245 | SNX31 | 9.277072639 | 3.78E-114 | 4.48E-114 |
| 101103253 | RGSL1 | 10.87681176 | 0 | 0 |
| 101103273 | APEH | -2.026937566 | 0 | 0 |
| 101103274 | DAAM2 | 4.159894134 | 0 | 0 |
| 101103280 | TIAM1 | -2.035218045 | 5.82E-111 | 6.79E-111 |
| 101103310 | ATP6V1C2 | -4.547074018 | 0 | 0 |
| 101103323 | GJD2 | -9.434150683 | 3.71E-124 | 4.66E-124 |
| 101103346 | LMO2 | 2.010440719 | 0 | 0 |
| 101103369 | IL6R | -2.079758235 | 0 | 0 |
| 101103373 | S1PR3 | 5.409604316 | 9.21E-132 | 1.20E-131 |
| 101103384 | UBXN10 | 2.758474703 | 0 | 0 |
| 101103396 | LOC101103396 | -3.703196831 | 0 | 0 |
| 101103398 | LOC101103398 | -2.626253708 | 0 | 0 |
| 101103426 | ANKH | -2.881316552 | 0 | 0 |
| 101103431 | TFAP2B | -7.05494354 | 0 | 0 |
| 101103475 | SNX24 | 3.225488504 | 0 | 0 |
| 101103584 | LOC101103584 | 9.794029556 | 0 | 0 |
| 101103632 | PLA2G2C | 3.31958553 | 0 | 0 |
| 101103654 | SLAIN1 | -2.18452444 | 0 | 0 |
| 101103656 | JMJD6 | 2.52429615 | 0 | 0 |
| 101103666 | LOC101103666 | -12.20467334 | 0 | 0 |
| 101103676 | INF2 | 3.382684709 | 0 | 0 |
| 101103681 | SYTL2 | -3.190246963 | 0 | 0 |
| 101103684 | C1H1orf146 | -2.022557159 | 4.31E-18 | 1.96E-18 |
| 101103695 | EPHA6 | 4.239475852 | 8.94E-133 | 1.17E-132 |
| 101103697 | AQP7 | -4.906993701 | 0 | 0 |
| 101103717 | PTPRZ1 | 2.564186156 | 0 | 0 |
| 101103729 | VGLL2 | -11.07381908 | 5.26E-299 | 1.23E-298 |
| 101103760 | TSPAN3 | 2.015464628 | 0 | 0 |
| 101103773 | GAP43 | -7.167886763 | 5.12E-227 | 9.76E-227 |
| 101103775 | SPTSSB | -9.349700361 | 0 | 0 |
| 101103801 | ACSS3 | 3.131695497 | 0 | 0 |
| 101103837 | ABTB2 | 2.597205913 | 3.40E-119 | 4.16E-119 |
| 101103838 | PIWIL4 | -6.877757335 | 1.28E-269 | 2.77E-269 |
| 101103907 | ST6GALNAC2 | 2.245788625 | 0 | 0 |
| 101103913 | PMEPA1 | 3.159994367 | 0 | 0 |
| 101103917 | BSPH1 | 2.622847977 | 0 | 0 |
| 101103967 | GDF3 | -11.85988047 | 0 | 0 |
| 101104034 | PPL | 2.316090797 | 0 | 0 |
| 101104067 | CWH43 | -4.742727025 | 0 | 0 |
| 101104089 | FUT4 | 2.825189952 | 1.81E-86 | 1.80E-86 |
| 101104100 | LY6G5C | 14.4013254 | 0 | 0 |
| 101104140 | PTGES | -5.065474329 | 0 | 0 |
| 101104193 | GAL3ST3 | 4.202502256 | 0 | 0 |
| 101104249 | NCOA2 | -2.441082288 | 0 | 0 |
| 101104262 | CST11 | 13.91446632 | 0 | 0 |
| 101104281 | SH3BP4 | -2.519532515 | 0 | 0 |
| 101104282 | MYBPHL | -8.575844415 | 0 | 0 |
| 101104361 | APOD | 2.067862479 | 0 | 0 |
| 101104372 | CA5B | -2.880955519 | 0 | 0 |
| 101104377 | RAP1GAP | 4.005420513 | 4.70E-234 | 9.14E-234 |
| 101104395 | HILPDA | -2.118708986 | 3.49E-118 | 4.24E-118 |
| 101104416 | HHAT | -2.188093473 | 0 | 0 |
| 101104418 | KIAA0513 | 2.613092737 | 2.56E-237 | 5.03E-237 |
| 101104442 | FAM69A | 2.738805646 | 1.18E-54 | 8.96E-55 |
| 101104473 | RND1 | 2.590309509 | 1.44E-47 | 1.01E-47 |
| 101104490 | SLCO4C1 | 3.010452357 | 0 | 0 |
| 101104491 | ARHGEF38 | -2.280856356 | 2.39E-174 | 3.77E-174 |
| 101104496 | GCNT4 | 10.55768342 | 0 | 0 |
| 101104518 | LOC101104518 | 8.591088368 | 1.47E-78 | 1.38E-78 |
| 101104532 | TCTA | -2.447001664 | 0 | 0 |
| 101104548 | C24H16orf89 | -11.16996745 | 0 | 0 |
| 101104557 | AOX1 | -3.038488956 | 0 | 0 |
| 101104568 | LOC101104568 | -4.233324708 | 0 | 0 |
| 101104587 | RHBDF2 | 2.302963207 | 8.90E-213 | 1.61E-212 |
| 101104593 | ENAH | 4.589646305 | 0 | 0 |
| 101104600 | UPK2 | -7.676123473 | 8.81E-24 | 4.46E-24 |
| 101104614 | MCCD1 | 2.736295155 | 2.50E-122 | 3.10E-122 |
| 101104633 | AP1S2 | 2.078420384 | 0 | 0 |
| 101104654 | SLC26A4 | -4.114021456 | 0 | 0 |
| 101104676 | ELF3 | -2.196479615 | 0 | 0 |
| 101104708 | UBASH3A | 2.696741587 | 8.92E-70 | 7.79E-70 |
| 101104709 | GPC1 | 3.508132022 | 0 | 0 |
| 101104720 | ARHGEF4 | -2.93937715 | 1.25E-55 | 9.62E-56 |
| 101104733 | PKP2 | 2.057493403 | 0 | 0 |
| 101104775 | ZCCHC14 | 2.121397089 | 1.50E-137 | 2.01E-137 |
| 101104798 | EEF2KMT | -3.331294169 | 0 | 0 |
| 101104821 | BZW2 | -2.269887851 | 0 | 0 |
| 101104845 | ENTPD6 | 2.652765494 | 0 | 0 |
| 101104858 | TPST2 | 4.332846007 | 0 | 0 |
| 101104863 | CPNE9 | -3.480136175 | 1.77E-50 | 1.28E-50 |
| 101104919 | SLC13A2 | 9.109609946 | 0 | 0 |
| 101104949 | B4GALT4 | 5.486539039 | 0 | 0 |
| 101104951 | APCDD1 | -2.591139326 | 0 | 0 |
| 101104977 | ST3GAL5 | 3.656766976 | 0 | 0 |
| 101105036 | LOC101105036 | -4.325626226 | 8.12E-15 | 3.44E-15 |
| 101105045 | PPM1L | -2.912385359 | 0 | 0 |
| 101105058 | SMPDL3B | 2.105165729 | 5.50E-273 | 1.20E-272 |
| 101105076 | ADAMTS10 | 4.148304962 | 0 | 0 |
| 101105168 | DIAPH3 | -2.444721283 | 3.44E-247 | 7.00E-247 |
| 101105173 | HS3ST3A1 | 8.917516779 | 0 | 0 |
| 101105202 | ARHGAP31 | 3.949332302 | 0 | 0 |
| 101105217 | GRB14 | -2.541769127 | 0 | 0 |
| 101105242 | SLC12A2 | 3.961011033 | 0 | 0 |
| 101105323 | AGR2 | -2.121600921 | 0 | 0 |
| 101105355 | SLC37A4 | -2.600637474 | 0 | 0 |
| 101105382 | CLDN16 | -3.625372411 | 1.14E-235 | 2.22E-235 |
| 101105402 | RERG | 2.593214972 | 0 | 0 |
| 101105405 | LOC101105405 | -9.587544254 | 2.21E-136 | 2.96E-136 |
| 101105433 | THAP8 | -2.152408747 | 1.17E-108 | 1.34E-108 |
| 101105461 | GNAL | -5.791600691 | 3.26E-106 | 3.70E-106 |
| 101105484 | LOC101105484 | 2.06371451 | 0 | 0 |
| 101105504 | RASGRP1 | 2.266628767 | 2.54E-307 | 6.02E-307 |
| 101105537 | PRSS16 | -8.365641905 | 3.08E-267 | 6.65E-267 |
| 101105554 | LOC101105554 | -2.034682035 | 4.10E-158 | 6.04E-158 |
| 101105557 | SHB | 3.005973603 | 1.07E-300 | 2.52E-300 |
| 101105582 | ACSF2 | 2.214844021 | 0 | 0 |
| 101105624 | EFNA1 | -2.005874142 | 0 | 0 |
| 101105629 | VEGFC | 4.773025172 | 0 | 0 |
| 101105652 | HOXA9 | -4.410873513 | 2.28E-217 | 4.20E-217 |
| 101105675 | CD34 | 3.860790643 | 0 | 0 |
| 101105695 | TREM1 | 6.84805091 | 1.34E-54 | 1.02E-54 |
| 101105716 | HSD3B7 | 4.837462848 | 2.84E-95 | 2.99E-95 |
| 101105792 | DLGAP1 | -2.584321696 | 9.95E-97 | 1.06E-96 |
| 101105804 | MSN | 2.296499124 | 0 | 0 |
| 101105839 | MIXL1 | 8.564257944 | 2.59E-151 | 3.71E-151 |
| 101105859 | FAM19A4 | -4.153445251 | 0 | 0 |
| 101105882 | S100G | 11.69886812 | 0 | 0 |
| 101105902 | SLC4A5 | 3.679296452 | 4.27E-83 | 4.13E-83 |
| 101105944 | NRIP3 | 4.676644017 | 0 | 0 |
| 101105968 | IMPA2 | -2.405438808 | 0 | 0 |
| 101105976 | LOC101105976 | 3.478200447 | 0 | 0 |
| 101105997 | LOC101105997 | -2.063163072 | 8.92E-48 | 6.30E-48 |
| 101106094 | NCF2 | 3.171018289 | 0 | 0 |
| 101106095 | C12H1orf95 | 3.061663306 | 0 | 0 |
| 101106160 | SERHL2 | -2.377786964 | 0 | 0 |
| 101106170 | SLC34A2 | 3.331464285 | 0 | 0 |
| 101106250 | LCN8 | 14.43341715 | 0 | 0 |
| 101106266 | F2RL1 | -3.305589676 | 0 | 0 |
| 101106273 | ZACN | -5.951857673 | 1.00E-263 | 2.14E-263 |
| 101106356 | WDR62 | -2.678770599 | 0 | 0 |
| 101106394 | LOC101106394 | -2.015345505 | 0 | 0 |
| 101106407 | CNKSR2 | 4.440281411 | 0 | 0 |
| 101106412 | ASL | -2.212394845 | 0 | 0 |
| 101106422 | INSIG1 | 2.269803273 | 0 | 0 |
| 101106424 | C5H5orf63 | -2.49030315 | 1.07E-236 | 2.09E-236 |
| 101106425 | TRIM7 | -4.581839366 | 4.73E-286 | 1.07E-285 |
| 101106452 | LOC101106452 | 2.531541957 | 0 | 0 |
| 101106473 | VSTM5 | -2.197339339 | 0 | 0 |
| 101106527 | SOWAHB | -2.374510714 | 1.48E-258 | 3.12E-258 |
| 101106534 | LOC101106534 | 3.811034622 | 1.27E-292 | 2.93E-292 |
| 101106543 | SYT9 | -2.67420627 | 1.32E-238 | 2.61E-238 |
| 101106548 | HDDC3 | 2.120630004 | 6.30E-137 | 8.46E-137 |
| 101106554 | GCNT2 | 3.218154662 | 0 | 0 |
| 101106589 | SPRY4 | 2.96552952 | 5.97E-299 | 1.40E-298 |
| 101106610 | LOC101106610 | -4.984254602 | 0 | 0 |
| 101106626 | EGFLAM | 4.920336208 | 0 | 0 |
| 101106664 | PLA2G2F | -4.887816006 | 0 | 0 |
| 101106719 | LAMB2 | -2.444953391 | 0 | 0 |
| 101106720 | LOC101106720 | -3.497659233 | 0 | 0 |
| 101106728 | LOC101106728 | -2.026418311 | 0 | 0 |
| 101106762 | LCN6 | 13.76301866 | 0 | 0 |
| 101106798 | MAN2A2 | 2.99218786 | 0 | 0 |
| 101106811 | CLDN8 | -3.187518794 | 0 | 0 |
| 101106830 | GLIPR2 | 2.443682265 | 6.01E-225 | 1.14E-224 |
| 101106849 | FSIP1 | -2.099827781 | 3.01E-223 | 5.65E-223 |
| 101106861 | MRPL27 | 2.216350349 | 6.99E-124 | 8.76E-124 |
| 101106897 | MGMT | 2.30037556 | 9.14E-232 | 1.77E-231 |
| 101106905 | NINJ1 | -2.507220573 | 0 | 0 |
| 101106908 | FRMPD1 | 4.458466347 | 0 | 0 |
| 101106916 | PLA2G2D | -4.82536089 | 4.06E-280 | 9.05E-280 |
| 101106919 | LOC101106919 | 3.749815574 | 0 | 0 |
| 101106938 | PLPP2 | 3.362510525 | 4.50E-148 | 6.36E-148 |
| 101106995 | LSS | 2.695808709 | 0 | 0 |
| 101106998 | LOC101106998 | -2.002869568 | 1.21E-306 | 2.87E-306 |
| 101107008 | SLC6A15 | -3.291177067 | 0 | 0 |
| 101107046 | NR3C2 | -3.04886203 | 0 | 0 |
| 101107086 | LOC101107086 | -4.279089142 | 0 | 0 |
| 101107103 | SLC16A10 | 4.47084871 | 0 | 0 |
| 101107112 | XYLT2 | 2.358047127 | 4.69E-162 | 7.06E-162 |
| 101107134 | BTG4 | 4.724550246 | 1.05E-86 | 1.04E-86 |
| 101107141 | CADPS | -5.320338395 | 0 | 0 |
| 101107163 | FBXO10 | -2.528605509 | 9.93E-189 | 1.65E-188 |
| 101107175 | ARHGEF10L | 2.325024943 | 6.38E-297 | 1.49E-296 |
| 101107210 | SOX13 | -2.519891282 | 6.40E-275 | 1.41E-274 |
| 101107232 | LOC101107232 | -3.426531706 | 0 | 0 |
| 101107267 | TSPAN19 | 3.538451286 | 2.84E-129 | 3.66E-129 |
| 101107378 | PABPC1L | -4.06207648 | 4.74E-199 | 8.16E-199 |
| 101107386 | LAYN | -2.310741324 | 0 | 0 |
| 101107483 | TRIM45 | -2.22963962 | 0 | 0 |
| 101107499 | NHS | -2.490898038 | 0 | 0 |
| 101107605 | LTBP2 | -2.795922228 | 0 | 0 |
| 101107621 | PFKFB3 | -3.16360989 | 0 | 0 |
| 101107630 | SIK2 | -2.337588527 | 0 | 0 |
| 101107650 | RGS10 | 2.077765969 | 7.76E-114 | 9.18E-114 |
| 101107655 | PXYLP1 | 2.028332259 | 0 | 0 |
| 101107683 | ATP6V1B1 | 7.382882922 | 0 | 0 |
| 101107690 | HK2 | -2.64182822 | 0 | 0 |
| 101107710 | ZDHHC14 | -3.015513882 | 1.51E-104 | 1.69E-104 |
| 101107719 | KISS1 | -6.011192668 | 7.55E-93 | 7.81E-93 |
| 101107762 | MAT1A | -2.873586888 | 0 | 0 |
| 101107767 | RENBP | -3.705264515 | 0 | 0 |
| 101107781 | ABCB5 | -5.494059826 | 0 | 0 |
| 101107790 | SALL2 | 2.364783127 | 1.99E-289 | 4.55E-289 |
| 101107876 | CLDN10 | 6.289878019 | 0 | 0 |
| 101107905 | C19H3orf67 | -2.47295175 | 4.37E-132 | 5.71E-132 |
| 101107915 | VTCN1 | 2.490430648 | 9.15E-143 | 1.26E-142 |
| 101107916 | PDLIM1 | 2.106559627 | 0 | 0 |
| 101107949 | CERCAM | -3.328146218 | 0 | 0 |
| 101107963 | MANBA | -3.066090578 | 0 | 0 |
| 101107976 | C12H1orf105 | -3.865868661 | 4.17E-249 | 8.52E-249 |
| 101107979 | GOLT1A | -6.664539886 | 0 | 0 |
| 101108027 | ARMCX2 | -3.228589592 | 0 | 0 |
| 101108052 | HDC | -4.442489984 | 8.25E-79 | 7.77E-79 |
| 101108061 | RGCC | -2.156450482 | 0 | 0 |
| 101108067 | SUSD4 | -5.545389498 | 0 | 0 |
| 101108090 | PPP1R32 | 4.503163631 | 9.16E-174 | 1.44E-173 |
| 101108123 | CARD10 | 3.613836003 | 0 | 0 |
| 101108169 | FAM3D | -3.751987471 | 0 | 0 |
| 101108171 | LOC101108171 | -2.099745502 | 0 | 0 |
| 101108212 | GUCY2C | -3.896490979 | 0 | 0 |
| 101108234 | SERAC1 | -2.030192955 | 4.55E-254 | 9.41E-254 |
| 101108248 | FUT1 | 10.88580583 | 7.72E-137 | 1.04E-136 |
| 101108270 | CMSS1 | 2.867082171 | 9.59E-209 | 1.71E-208 |
| 101108280 | RUNX1 | 3.732736308 | 0 | 0 |
| 101108281 | DYDC2 | 2.561899044 | 1.56E-64 | 1.30E-64 |
| 101108282 | TENM3 | 3.286906067 | 0 | 0 |
| 101108335 | APRT | 3.971427315 | 4.23E-224 | 7.97E-224 |
| 101108418 | ARRB1 | 2.423333927 | 0 | 0 |
| 101108447 | IQGAP3 | -3.609305768 | 0 | 0 |
| 101108488 | SLC39A8 | -5.299919769 | 0 | 0 |
| 101108524 | FOXP4 | 2.176874114 | 6.96E-97 | 7.42E-97 |
| 101108531 | MAP3K7CL | 2.244836705 | 1.70E-145 | 2.38E-145 |
| 101108560 | SYCP3 | 2.849223293 | 0 | 0 |
| 101108572 | KIAA1211 | -4.628806889 | 0 | 0 |
| 101108589 | SNAP25 | -3.897735926 | 4.80E-266 | 1.03E-265 |
| 101108621 | TVP23A | -4.773085203 | 2.41E-70 | 2.11E-70 |
| 101108654 | LOC101108654 | 5.677260049 | 0 | 0 |
| 101108705 | LOC101108705 | -3.479470677 | 0 | 0 |
| 101108710 | RBP2 | 4.805996929 | 1.31E-142 | 1.81E-142 |
| 101108713 | TTC24 | -3.055666974 | 0 | 0 |
| 101108738 | SLC9A2 | 3.818303567 | 1.34E-242 | 2.68E-242 |
| 101108740 | KIAA0930 | -2.074127424 | 0 | 0 |
| 101108745 | FUT5 | -4.413358453 | 0 | 0 |
| 101108788 | CLCF1 | 2.539780918 | 1.26E-121 | 1.56E-121 |
| 101108821 | CHPT1 | 3.656062109 | 0 | 0 |
| 101108825 | ANO6 | -2.437291621 | 0 | 0 |
| 101108867 | IER3 | 2.930413933 | 0 | 0 |
| 101108877 | TNP2 | 4.060954827 | 8.35E-55 | 6.36E-55 |
| 101108901 | LOC101108901 | 8.08376471 | 5.41E-30 | 3.02E-30 |
| 101108928 | PIPOX | 6.437401664 | 1.23E-183 | 2.00E-183 |
| 101108939 | CAMK1D | -2.895956563 | 0 | 0 |
| 101108977 | RBP1 | 3.468726763 | 0 | 0 |
| 101109034 | DEFB129 | -12.85698996 | 0 | 0 |
| 101109049 | FADS1 | 2.453209644 | 0 | 0 |
| 101109080 | LOC101109080 | -2.163345409 | 4.15E-32 | 2.40E-32 |
| 101109152 | PQLC2 | 2.994277223 | 0 | 0 |
| 101109206 | MFRP | -6.666447414 | 0 | 0 |
| 101109232 | STK32C | 3.181386677 | 3.11E-115 | 3.72E-115 |
| 101109241 | SLC31A2 | -3.516723652 | 0 | 0 |
| 101109259 | WIF1 | 4.927914894 | 3.03E-191 | 5.07E-191 |
| 101109268 | LOC101109268 | 4.006130486 | 2.24E-217 | 4.12E-217 |
| 101109288 | DHRS3 | 2.160302972 | 0 | 0 |
| 101109293 | DEFB127 | 3.136859796 | 4.64E-147 | 6.51E-147 |
| 101109353 | FGF1 | 2.324873237 | 2.90E-84 | 2.83E-84 |
| 101109385 | NLRP5 | -4.105837499 | 4.14E-126 | 5.26E-126 |
| 101109397 | LOC101109397 | -4.580270656 | 9.63E-195 | 1.63E-194 |
| 101109408 | LITAF | 2.756615082 | 0 | 0 |
| 101109421 | AKR7A2 | 4.10725791 | 0 | 0 |
| 101109433 | LRRC23 | 2.754408369 | 8.63E-255 | 1.79E-254 |
| 101109444 | ZNF608 | -2.762586157 | 0 | 0 |
| 101109471 | CCDC3 | -2.536014256 | 0 | 0 |
| 101109490 | IL17RD | 2.250271367 | 0 | 0 |
| 101109506 | MMD2 | 2.018831155 | 0 | 0 |
| 101109515 | CITED1 | 3.225189339 | 0 | 0 |
| 101109536 | GALNT10 | -2.37776385 | 0 | 0 |
| 101109548 | RNF112 | -2.381744694 | 6.92E-244 | 1.39E-243 |
| 101109586 | WBSCR17 | -5.687347012 | 0 | 0 |
| 101109592 | ALS2 | -2.409866558 | 0 | 0 |
| 101109609 | SH3RF3 | 2.132764111 | 1.38E-127 | 1.76E-127 |
| 101109652 | LOC101109652 | -4.706586502 | 7.55E-73 | 6.79E-73 |
| 101109687 | PROKR1 | -5.761211103 | 0 | 0 |
| 101109748 | ZFP57 | -2.776664253 | 0 | 0 |
| 101109766 | SH2D4B | -4.761820377 | 2.32E-205 | 4.08E-205 |
| 101109803 | SNX22 | -4.179243947 | 0 | 0 |
| 101109816 | ADORA1 | 10.87855515 | 1.43E-270 | 3.10E-270 |
| 101109819 | TMCC2 | -2.738437589 | 1.94E-231 | 3.74E-231 |
| 101109821 | DEFB125 | -10.90102134 | 0 | 0 |
| 101109833 | TMEM89 | -3.857847265 | 2.48E-40 | 1.60E-40 |
| 101109840 | SNX7 | -2.003698131 | 7.59E-102 | 8.36E-102 |
| 101109846 | GALNT1 | -3.961047521 | 0 | 0 |
| 101109910 | STC2 | -3.812160689 | 2.90E-266 | 6.25E-266 |
| 101109919 | LOC101109919 | -3.374953939 | 4.74E-90 | 4.82E-90 |
| 101110019 | TPST1 | -2.293875614 | 0 | 0 |
| 101110092 | CPEB1 | 3.553477189 | 7.25E-190 | 1.21E-189 |
| 101110104 | NT5C2 | -2.050504451 | 0 | 0 |
| 101110105 | CRTAC1 | 6.084838543 | 0 | 0 |
| 101110106 | INO80C | -2.633984623 | 0 | 0 |
| 101110109 | DECR2 | 2.694646169 | 3.71E-53 | 2.78E-53 |
| 101110184 | SFTA2 | -2.119770275 | 0 | 0 |
| 101110195 | LOC101110195 | -7.758585634 | 6.46E-25 | 3.33E-25 |
| 101110204 | ILDR2 | -4.739935064 | 0 | 0 |
| 101110235 | MLLT1 | -2.308858013 | 0 | 0 |
| 101110248 | ZAR1L | -2.524162167 | 0 | 0 |
| 101110254 | MYBPH | 7.704322372 | 0 | 0 |
| 101110257 | SPTLC3 | -3.044852067 | 0 | 0 |
| 101110322 | FAM221A | -3.313423488 | 2.67E-268 | 5.77E-268 |
| 101110338 | FILIP1 | -2.941680074 | 0 | 0 |
| 101110343 | CA8 | 3.827928814 | 0 | 0 |
| 101110344 | ETV4 | 4.565319937 | 0 | 0 |
| 101110347 | CACNG4 | 7.188594683 | 1.30E-132 | 1.70E-132 |
| 101110361 | DTX4 | -4.517062541 | 0 | 0 |
| 101110380 | RAMP1 | 2.143143257 | 4.04E-30 | 2.26E-30 |
| 101110407 | SARDH | 4.275574266 | 0 | 0 |
| 101110415 | SV2C | -5.756879312 | 0 | 0 |
| 101110468 | GOLGB1 | -2.598067229 | 0 | 0 |
| 101110562 | SDK1 | -2.088648004 | 5.40E-240 | 1.07E-239 |
| 101110570 | VIL1 | 5.145785072 | 0 | 0 |
| 101110596 | GPX2 | -5.381479461 | 2.25E-187 | 3.71E-187 |
| 101110630 | CLSPN | -3.111501421 | 2.48E-217 | 4.56E-217 |
| 101110645 | PALD1 | -2.700246268 | 3.10E-251 | 6.37E-251 |
| 101110657 | LOC101110657 | 2.176007891 | 3.28E-105 | 3.70E-105 |
| 101110684 | OPLAH | -2.248146164 | 0 | 0 |
| 101110694 | SLC17A9 | 3.907948933 | 0 | 0 |
| 101110712 | SERPINB1 | 3.842020484 | 0 | 0 |
| 101110747 | POC1B | 2.568116034 | 0 | 0 |
| 101110769 | PKIA | -2.726194775 | 0 | 0 |
| 101110783 | HSF4 | -2.752047835 | 0 | 0 |
| 101110813 | CARD11 | -4.370111612 | 0 | 0 |
| 101110817 | SLC7A2 | -3.668071662 | 0 | 0 |
| 101110821 | MBNL3 | -2.799600003 | 2.23E-294 | 5.15E-294 |
| 101110872 | CAPSL | 10.2677649 | 8.00E-99 | 8.62E-99 |
| 101110878 | ISLR | 3.729178575 | 0 | 0 |
| 101110890 | PITPNM1 | 4.327242143 | 0 | 0 |
| 101110894 | MAPRE2 | -2.09706963 | 0 | 0 |
| 101110896 | NME9 | 5.306157131 | 6.97E-280 | 1.55E-279 |
| 101110915 | ENTPD2 | 3.599503488 | 0 | 0 |
| 101110924 | PCED1B | -3.692070957 | 0 | 0 |
| 101110932 | CCDC126 | -3.841237766 | 0 | 0 |
| 101110972 | IQSEC1 | -3.483691073 | 9.81E-123 | 1.22E-122 |
| 101110974 | LOC101110974 | -5.834027319 | 0 | 0 |
| 101111006 | LOC101111006 | 5.1703666 | 0 | 0 |
| 101111023 | BEND4 | -3.329628157 | 0 | 0 |
| 101111037 | FMOD | 2.939176612 | 0 | 0 |
| 101111038 | EPHX1 | 2.33731872 | 0 | 0 |
| 101111048 | WNT11 | 4.874863741 | 1.26E-163 | 1.91E-163 |
| 101111072 | MTMR7 | -3.125897525 | 6.91E-213 | 1.25E-212 |
| 101111138 | LPAR3 | -3.627843536 | 0 | 0 |
| 101111145 | PSTPIP2 | -3.537292241 | 0 | 0 |
| 101111151 | PTGFRN | -2.321126553 | 0 | 0 |
| 101111179 | AMIGO2 | -3.026403905 | 1.07E-270 | 2.33E-270 |
| 101111183 | ADAMTSL2 | 4.787656312 | 0 | 0 |
| 101111208 | CBX8 | 2.137394188 | 0 | 0 |
| 101111209 | CLCN6 | -2.174852426 | 0 | 0 |
| 101111238 | FRRS1 | -2.002226744 | 0 | 0 |
| 101111242 | LOC101111242 | 4.795407118 | 0 | 0 |
| 101111279 | SNX2 | 2.037143395 | 0 | 0 |
| 101111284 | CCDC69 | 4.401388492 | 0 | 0 |
| 101111295 | CDH17 | 3.227865936 | 3.17E-282 | 7.12E-282 |
| 101111297 | MEDAG | -2.423287521 | 0 | 0 |
| 101111299 | SSH2 | 2.052891702 | 5.70E-249 | 1.16E-248 |
| 101111308 | WFDC2 | 2.341178296 | 0 | 0 |
| 101111313 | LOC101111313 | -8.336853482 | 0 | 0 |
| 101111331 | CYYR1 | -2.094193 | 2.80E-292 | 6.44E-292 |
| 101111372 | FUT8 | -3.371081541 | 0 | 0 |
| 101111379 | KRT23 | -6.560842688 | 7.75E-264 | 1.65E-263 |
| 101111412 | SLC15A2 | 9.796004694 | 0 | 0 |
| 101111428 | RPS6KA3 | -3.333250549 | 0 | 0 |
| 101111472 | PLA2G4A | 3.027402267 | 0 | 0 |
| 101111479 | KCTD15 | 3.828271209 | 0 | 0 |
| 101111505 | LOC101111505 | 2.384227522 | 9.46E-130 | 1.22E-129 |
| 101111528 | LOC101111528 | -2.150968024 | 0 | 0 |
| 101111545 | RGMB | -2.24093336 | 0 | 0 |
| 101111562 | NXPH3 | 2.027787313 | 0 | 0 |
| 101111574 | OVCH2 | 14.11849433 | 0 | 0 |
| 101111645 | MTHFR | -2.632678209 | 0 | 0 |
| 101111656 | TP53I11 | -4.600504602 | 0 | 0 |
| 101111669 | LOC101111669 | -9.370020346 | 7.14E-61 | 5.75E-61 |
| 101111672 | ROM1 | 3.196239439 | 1.96E-241 | 3.91E-241 |
| 101111679 | LIPG | -2.411253384 | 0 | 0 |
| 101111708 | GALNT8 | -4.534394546 | 0 | 0 |
| 101111709 | STEAP1 | 5.916840574 | 2.46E-110 | 2.86E-110 |
| 101111717 | SLC22A16 | 2.824465253 | 4.46E-133 | 5.87E-133 |
| 101111719 | LOC101111719 | -2.181926437 | 1.91E-246 | 3.87E-246 |
| 101111724 | MPP2 | -2.91864081 | 0 | 0 |
| 101111757 | PDE4B | 4.580843942 | 0 | 0 |
| 101111766 | ATP2A1 | -4.477315656 | 1.50E-115 | 1.79E-115 |
| 101111824 | WFDC8 | -4.788674363 | 0 | 0 |
| 101111832 | FNIP2 | -2.189102842 | 5.51E-222 | 1.03E-221 |
| 101111860 | TMEM37 | 3.949619826 | 5.13E-172 | 8.02E-172 |
| 101111906 | HEXB | -5.154993823 | 0 | 0 |
| 101111915 | LOC101111915 | -5.731618586 | 0 | 0 |
| 101111950 | SLC3A1 | 6.626777811 | 0 | 0 |
| 101111960 | STEAP2 | 7.31259967 | 0 | 0 |
| 101111972 | DUOX1 | -6.91432409 | 0 | 0 |
| 101112021 | CLIC6 | 3.635672968 | 0 | 0 |
| 101112026 | SMPX | 5.043742863 | 0 | 0 |
| 101112033 | GUCY2F | -6.297005548 | 1.94E-130 | 2.52E-130 |
| 101112081 | WFDC13 | -12.16200658 | 0 | 0 |
| 101112084 | LOC101112084 | -2.402822278 | 0 | 0 |
| 101112113 | PTGR1 | 2.114658718 | 0 | 0 |
| 101112114 | SCTR | 2.062752221 | 0 | 0 |
| 101112133 | LYPD8 | -4.92388555 | 0 | 0 |
| 101112162 | LOC101112162 | -5.740986907 | 0 | 0 |
| 101112196 | RAPGEF4 | 2.467493155 | 6.30E-160 | 9.38E-160 |
| 101112270 | LOC101112270 | -3.64200586 | 0 | 0 |
| 101112277 | GATSL2 | 2.550379036 | 0 | 0 |
| 101112340 | SPINT4 | -15.39022979 | 0 | 0 |
| 101112346 | RCN1 | 2.951907149 | 0 | 0 |
| 101112385 | MON2 | 3.14369121 | 0 | 0 |
| 101112423 | GGT5 | -3.09767428 | 0 | 0 |
| 101112424 | LOXL1 | 2.533689671 | 8.57E-243 | 1.71E-242 |
| 101112455 | TMEM255A | -2.616824163 | 1.31E-166 | 2.01E-166 |
| 101112456 | IRS4 | -6.355299029 | 0 | 0 |
| 101112470 | SLC38A1 | -2.982149735 | 0 | 0 |
| 101112483 | LOC101112483 | -3.704194494 | 0 | 0 |
| 101112544 | PLP2 | -2.154385405 | 0 | 0 |
| 101112591 | RYR1 | -4.012260145 | 9.90E-144 | 1.37E-143 |
| 101112600 | PDGFC | 3.05207284 | 0 | 0 |
| 101112606 | LOC101112606 | -8.058695083 | 0 | 0 |
| 101112618 | MME | 2.782644676 | 0 | 0 |
| 101112628 | LONRF1 | -2.135869881 | 0 | 0 |
| 101112642 | KIAA1324L | 2.565834329 | 1.46E-159 | 2.17E-159 |
| 101112653 | IL20RA | 3.299614285 | 2.05E-141 | 2.81E-141 |
| 101112685 | CST6 | 6.930673201 | 0 | 0 |
| 101112690 | NXPE3 | 2.066183295 | 0 | 0 |
| 101112732 | WFS1 | -2.265562365 | 0 | 0 |
| 101112761 | MS4A7 | 8.896813552 | 0 | 0 |
| 101112780 | CSTA | -9.785057845 | 1.01E-298 | 2.36E-298 |
| 101112806 | BHLHE41 | 2.34929179 | 0 | 0 |
| 101112829 | KIAA0226L | -3.415586247 | 0 | 0 |
| 101112830 | UBL3 | 4.007970176 | 0 | 0 |
| 101112930 | GLB1 | -2.52146809 | 0 | 0 |
| 101112949 | STC1 | -2.302762118 | 1.92E-197 | 3.29E-197 |
| 101112956 | PXDN | 2.61785804 | 3.01E-238 | 5.94E-238 |
| 101112976 | CREB5 | -3.153538887 | 0 | 0 |
| 101112980 | TSPAN5 | -2.517364592 | 0 | 0 |
| 101113028 | FAM78B | 5.833929802 | 3.86E-217 | 7.08E-217 |
| 101113054 | PLB1 | -5.643108416 | 2.49E-307 | 5.92E-307 |
| 101113073 | LOC101113073 | -12.84916807 | 0 | 0 |
| 101113074 | PLCB2 | -3.487541416 | 0 | 0 |
| 101113098 | DEPDC7 | -2.680424339 | 0 | 0 |
| 101113116 | NEURL1 | -2.180360078 | 0 | 0 |
| 101113161 | PYCR1 | 2.399395503 | 0 | 0 |
| 101113165 | RBM17 | 5.192405879 | 0 | 0 |
| 101113189 | LOC101113189 | -2.686882065 | 4.11E-220 | 7.63E-220 |
| 101113190 | S100B | 6.929655873 | 0 | 0 |
| 101113212 | CD164L2 | 2.110425078 | 1.34E-41 | 8.81E-42 |
| 101113240 | LTB4R | 5.295554642 | 0 | 0 |
| 101113262 | LRRC36 | 6.324858702 | 0 | 0 |
| 101113302 | FAM25A | -3.032844477 | 3.03E-69 | 2.63E-69 |
| 101113311 | CHRDL1 | -2.506198472 | 1.70E-114 | 2.02E-114 |
| 101113312 | PTPRN | -2.72454406 | 1.02E-28 | 5.59E-29 |
| 101113331 | LOC101113331 | -9.913956092 | 0 | 0 |
| 101113341 | LOC101113341 | 3.531124427 | 0 | 0 |
| 101113363 | ADGRD1 | -4.013080098 | 0 | 0 |
| 101113389 | CLDN23 | -4.335502258 | 0 | 0 |
| 101113397 | LYPD6B | -2.997356605 | 4.99E-153 | 7.22E-153 |
| 101113407 | MGAM | -11.50950969 | 1.31E-188 | 2.17E-188 |
| 101113467 | CYTIP | 2.932888303 | 0 | 0 |
| 101113480 | SLC1A4 | 5.837636507 | 0 | 0 |
| 101113497 | CIDEB | 3.733267463 | 8.83E-58 | 6.91E-58 |
| 101113519 | TPPP3 | 3.269919613 | 0 | 0 |
| 101113556 | NUPR1 | 2.079630252 | 0 | 0 |
| 101113583 | LOC101113583 | 2.545799689 | 0 | 0 |
| 101113587 | THEG | 7.881998242 | 1.17E-201 | 2.02E-201 |
| 101113588 | UNC13A | -8.909200853 | 0 | 0 |
| 101113635 | DDAH1 | 2.081098342 | 0 | 0 |
| 101113663 | KIF5C | 3.171120684 | 0 | 0 |
| 101113693 | LOC101113693 | 3.300082617 | 5.07E-16 | 2.20E-16 |
| 101113728 | LOC101113728 | -2.417920959 | 0 | 0 |
| 101113735 | GALNT7 | -2.509893547 | 7.60E-290 | 1.73E-289 |
| 101113744 | SCN8A | 2.989988095 | 8.45E-304 | 1.99E-303 |
| 101113761 | LOC101113761 | -7.427766417 | 1.25E-228 | 2.39E-228 |
| 101113769 | TEX30 | 2.225222333 | 1.02E-213 | 1.84E-213 |
| 101113836 | ACVR1B | -2.031152943 | 0 | 0 |
| 101113839 | SEMA3E | -8.365641905 | 1.19E-134 | 1.57E-134 |
| 101113888 | STXBP6 | -2.236067905 | 6.05E-241 | 1.20E-240 |
| 101113902 | RAB26 | 2.772629895 | 2.45E-51 | 1.80E-51 |
| 101113927 | ANKRD23 | -3.211788384 | 0 | 0 |
| 101113959 | PLA2G3 | 4.432853262 | 3.20E-126 | 4.07E-126 |
| 101113980 | EFHD1 | -2.503109188 | 4.70E-216 | 8.60E-216 |
| 101113986 | PEBP4 | -2.223474403 | 0 | 0 |
| 101114022 | SLC30A4 | -2.398288589 | 0 | 0 |
| 101114059 | ASB2 | -5.516677763 | 0 | 0 |
| 101114062 | CUL9 | -2.394138947 | 0 | 0 |
| 101114110 | SPATA18 | 4.903801146 | 0 | 0 |
| 101114112 | TMEM229B | -4.026126648 | 0 | 0 |
| 101114126 | ACSS2 | -3.177414712 | 0 | 0 |
| 101114233 | PPP2R3A | -2.30884551 | 0 | 0 |
| 101114239 | LOC101114239 | 2.141732308 | 0 | 0 |
| 101114273 | ZBTB49 | -2.301475327 | 0 | 0 |
| 101114295 | WFDC11 | -4.20374348 | 0 | 0 |
| 101114300 | NKPD1 | -2.415852728 | 1.81E-98 | 1.95E-98 |
| 101114311 | LOC101114311 | -4.880593777 | 0 | 0 |
| 101114318 | HTATIP2 | -2.297583562 | 0 | 0 |
| 101114362 | PIGH | -2.499800701 | 2.91E-177 | 4.65E-177 |
| 101114392 | NOVA1 | -2.602546158 | 0 | 0 |
| 101114419 | LOC101114419 | -2.528195991 | 2.42E-109 | 2.79E-109 |
| 101114470 | FAM174B | -2.009411783 | 3.33E-55 | 2.55E-55 |
| 101114471 | ENTPD3 | -7.775297887 | 0 | 0 |
| 101114472 | DCLK3 | 5.336799161 | 2.51E-257 | 5.27E-257 |
| 101114531 | C9H8orf88 | -2.315095559 | 1.36E-70 | 1.20E-70 |
| 101114540 | TMC8 | 2.070652525 | 1.22E-111 | 1.43E-111 |
| 101114541 | MFSD4 | -5.699899474 | 0 | 0 |
| 101114544 | GSS | -2.507864489 | 0 | 0 |
| 101114598 | KCNIP3 | 8.08376471 | 1.27E-114 | 1.52E-114 |
| 101114660 | ZBTB7C | 3.637670891 | 0 | 0 |
| 101114662 | EPHB1 | 4.774378998 | 0 | 0 |
| 101114682 | DMD | -2.231106169 | 0 | 0 |
| 101114819 | SLC41A3 | -2.653311166 | 6.24E-155 | 9.07E-155 |
| 101114832 | ACP6 | 2.751899533 | 0 | 0 |
| 101114837 | CELF4 | 6.754202609 | 0 | 0 |
| 101114859 | GALNT6 | 4.2894352 | 0 | 0 |
| 101114861 | LOC101114861 | 8.735693034 | 0 | 0 |
| 101114907 | CHGA | 5.470876973 | 0 | 0 |
| 101114959 | LOC101114959 | -3.023095041 | 1.02E-82 | 9.82E-83 |
| 101115034 | DUOXA1 | -11.55111085 | 0 | 0 |
| 101115062 | MUC15 | 2.029795375 | 0 | 0 |
| 101115118 | WDR86 | 3.684377643 | 4.14E-258 | 8.69E-258 |
| 101115125 | TMEM150C | 3.166210366 | 0 | 0 |
| 101115161 | ITPK1 | 2.006853931 | 6.99E-261 | 1.48E-260 |
| 101115266 | RNF144A | 4.380670538 | 3.11E-240 | 6.18E-240 |
| 101115285 | SHARPIN | 2.350661755 | 0 | 0 |
| 101115292 | ABCA5 | -4.505932284 | 0 | 0 |
| 101115303 | ABCC12 | -5.887343454 | 0 | 0 |
| 101115343 | LOC101115343 | 4.164520548 | 0 | 0 |
| 101115372 | PLA2G4B | 2.119375926 | 0 | 0 |
| 101115379 | SHISA2 | 2.630556543 | 0 | 0 |
| 101115418 | ETV5 | 3.636699376 | 0 | 0 |
| 101115470 | GRN | -2.368473012 | 0 | 0 |
| 101115489 | RUNX2 | 4.033212559 | 0 | 0 |
| 101115505 | SIDT1 | 3.131678643 | 4.75E-166 | 7.28E-166 |
| 101115524 | ATRAID | -2.396386989 | 0 | 0 |
| 101115527 | LOC101115527 | -6.553681771 | 0 | 0 |
| 101115544 | GRAMD2 | -2.01390726 | 0 | 0 |
| 101115554 | HS3ST3B1 | 5.116008156 | 0 | 0 |
| 101115601 | GPRIN2 | 3.894003293 | 3.44E-66 | 2.91E-66 |
| 101115624 | CHRNA9 | 12.10611947 | 1.71E-256 | 3.57E-256 |
| 101115627 | ITPKA | -2.538829476 | 1.49E-89 | 1.50E-89 |
| 101115633 | HOXB1 | -2.172165129 | 5.38E-117 | 6.51E-117 |
| 101115635 | RHBDL3 | 4.330650772 | 0 | 0 |
| 101115646 | LOC101115646 | 2.033888069 | 2.12E-205 | 3.73E-205 |
| 101115657 | NRG4 | -3.447139294 | 0 | 0 |
| 101115696 | METTL7B | -8.290889109 | 0 | 0 |
| 101115699 | DUSP2 | 9.15561877 | 0 | 0 |
| 101115707 | PCSK4 | -2.087419957 | 1.68E-76 | 1.55E-76 |
| 101115711 | KLHL33 | -2.642023858 | 1.19E-106 | 1.35E-106 |
| 101115728 | ZNF536 | 5.042529146 | 0 | 0 |
| 101115732 | LOC101115732 | -4.900823772 | 0 | 0 |
| 101115740 | LOC101115740 | -8.347994039 | 1.33E-67 | 1.14E-67 |
| 101115776 | CAD | -2.052651181 | 0 | 0 |
| 101115792 | EFNA5 | -4.328156489 | 0 | 0 |
| 101115816 | LPCAT2 | 3.10414837 | 0 | 0 |
| 101115835 | HIVEP1 | -2.300729354 | 7.88E-210 | 1.41E-209 |
| 101115917 | ACTN3 | 2.605111111 | 7.48E-116 | 8.97E-116 |
| 101115959 | LOC101115959 | -3.034431259 | 0 | 0 |
| 101115963 | PLK5 | -3.281330473 | 3.03E-245 | 6.12E-245 |
| 101115988 | LOC101115988 | -3.957333875 | 0 | 0 |
| 101116008 | ALPK2 | -2.583057926 | 1.58E-38 | 9.94E-39 |
| 101116011 | LOC101116011 | -3.626532939 | 1.17E-45 | 8.03E-46 |
| 101116016 | C26H8orf4 | 2.071537634 | 6.31E-217 | 1.16E-216 |
| 101116034 | DNAJC5G | -4.891518764 | 1.91E-226 | 3.64E-226 |
| 101116109 | ROPN1 | 6.16321812 | 0 | 0 |
| 101116129 | RIPPLY1 | -2.333559086 | 1.69E-256 | 3.54E-256 |
| 101116130 | SGPP2 | -2.081629902 | 0 | 0 |
| 101116157 | LOC101116157 | -4.347414915 | 2.43E-104 | 2.72E-104 |
| 101116261 | XYLB | 2.710526785 | 0 | 0 |
| 101116286 | LOC101116286 | 4.515430038 | 0 | 0 |
| 101116327 | CARS2 | 3.041953513 | 0 | 0 |
| 101116331 | MXRA7 | 2.512550318 | 0 | 0 |
| 101116425 | TRIM29 | 4.117754774 | 0 | 0 |
| 101116428 | P2RX2 | 2.210777676 | 0 | 0 |
| 101116483 | RNASE12 | -2.189205711 | 0 | 0 |
| 101116493 | SRGAP2 | -3.204343534 | 0 | 0 |
| 101116526 | IGSF9 | -2.701421774 | 0 | 0 |
| 101116544 | NCMAP | 8.730763252 | 0 | 0 |
| 101116550 | PLCXD1 | -2.57187918 | 3.48E-208 | 6.19E-208 |
| 101116568 | ABCA7 | -3.447527454 | 0 | 0 |
| 101116569 | CXXC5 | 3.07843783 | 0 | 0 |
| 101116588 | ST6GALNAC1 | -7.104885947 | 0 | 0 |
| 101116590 | KIAA1462 | 2.009764128 | 1.27E-171 | 1.99E-171 |
| 101116663 | DEPTOR | -2.504004735 | 4.91E-249 | 1.00E-248 |
| 101116679 | LRCOL1 | 2.324203864 | 0 | 0 |
| 101116716 | SLC8A1 | -2.580237478 | 0 | 0 |
| 101116734 | RNASE11 | -3.844755466 | 0 | 0 |
| 101116737 | TONSL | -3.287651485 | 0 | 0 |
| 101116788 | LIPH | 4.143050977 | 2.53E-173 | 3.97E-173 |
| 101116792 | DOC2A | 3.736781947 | 2.33E-111 | 2.73E-111 |
| 101116795 | LOC101116795 | 8.502592348 | 0 | 0 |
| 101116841 | LOC101116841 | 3.658021568 | 1.01E-153 | 1.46E-153 |
| 101116855 | NKD1 | 2.488154965 | 6.41E-223 | 1.20E-222 |
| 101116862 | LOC101116862 | -3.503109188 | 3.78E-239 | 7.48E-239 |
| 101116888 | ZNF41 | -2.089801514 | 0 | 0 |
| 101116936 | CCDC64 | -2.603372504 | 0 | 0 |
| 101116939 | PSTPIP1 | -3.428597831 | 0 | 0 |
| 101117036 | MAP3K13 | -2.671452867 | 0 | 0 |
| 101117092 | PRPSAP1 | -2.031313654 | 0 | 0 |
| 101117095 | C13H20orf85 | 3.530280441 | 2.01E-28 | 1.10E-28 |
| 101117129 | LOC101117129 | -3.918751097 | 0 | 0 |
| 101117158 | VWDE | 6.622741005 | 6.78E-116 | 8.15E-116 |
| 101117176 | RAB11FIP4 | -2.805684701 | 0 | 0 |
| 101117246 | ATP10B | -2.940909178 | 1.42E-284 | 3.21E-284 |
| 101117346 | SLC16A11 | -4.878591909 | 0 | 0 |
| 101117367 | EGLN3 | 2.72242823 | 0 | 0 |
| 101117382 | TAF4B | -3.30705412 | 2.08E-156 | 3.05E-156 |
| 101117383 | CCDC178 | 8.044334565 | 0 | 0 |
| 101117404 | BUB1 | -3.284284099 | 0 | 0 |
| 101117454 | BSN | -2.523112601 | 0 | 0 |
| 101117462 | CLRN3 | 6.767791338 | 4.74E-102 | 5.22E-102 |
| 101117463 | NRIP1 | -2.092851643 | 0 | 0 |
| 101117468 | PRSS27 | 8.286320715 | 1.99E-65 | 1.67E-65 |
| 101117476 | GLDC | -3.412469907 | 0 | 0 |
| 101117552 | MRO | -5.612660942 | 0 | 0 |
| 101117553 | CCBE1 | -2.700100155 | 2.16E-126 | 2.75E-126 |
| 101117567 | TRIM32 | 2.015901046 | 1.13E-142 | 1.56E-142 |
| 101117587 | LOC101117587 | -9.631873568 | 0 | 0 |
| 101117596 | EBPL | 2.466328602 | 9.59E-196 | 1.63E-195 |
| 101117610 | NLRP12 | -7.013004243 | 5.36E-177 | 8.55E-177 |
| 101117611 | TOX3 | -2.892971314 | 0 | 0 |
| 101117636 | KLHL14 | 4.199241927 | 0 | 0 |
| 101117637 | P2RY14 | 2.035117541 | 0 | 0 |
| 101117666 | SHH | -4.296843326 | 0 | 0 |
| 101117670 | ARL9 | -2.879337793 | 1.12E-67 | 9.60E-68 |
| 101117682 | KRT15 | -2.963198536 | 2.01E-283 | 4.52E-283 |
| 101117683 | KSR1 | -3.091339395 | 0 | 0 |
| 101117691 | LOC101117691 | -2.518019922 | 0 | 0 |
| 101117712 | PATE2 | -4.458907209 | 0 | 0 |
| 101117731 | UNC13B | -2.385208416 | 0 | 0 |
| 101117821 | ERICH2 | 2.667125106 | 2.05E-101 | 2.25E-101 |
| 101117846 | MAN2B2 | -8.890623795 | 0 | 0 |
| 101117876 | ESM1 | -3.821304749 | 0 | 0 |
| 101117884 | MSH5 | -2.866922947 | 0 | 0 |
| 101117908 | WBP5 | -2.032760597 | 5.39E-77 | 5.01E-77 |
| 101117955 | LOC101117955 | -3.487410839 | 0 | 0 |
| 101117962 | SORL1 | -2.254089737 | 0 | 0 |
| 101117975 | RHBDL2 | 2.494728889 | 0 | 0 |
| 101117981 | KCNJ15 | -2.475996556 | 0 | 0 |
| 101117988 | ERO1B | 2.16742473 | 0 | 0 |
| 101118004 | LOC101118004 | -8.738407752 | 4.51E-43 | 3.01E-43 |
| 101118010 | ACOXL | -3.672821665 | 0 | 0 |
| 101118012 | ODF3B | 5.927039206 | 4.72E-16 | 2.05E-16 |
| 101118044 | TTC9B | -4.203370476 | 1.63E-33 | 9.58E-34 |
| 101118079 | MYO3B | -2.999600395 | 0 | 0 |
| 101118080 | NABP1 | -2.181880609 | 9.87E-289 | 2.25E-288 |
| 101118082 | INHA | -4.531409096 | 0 | 0 |
| 101118098 | POLM | -2.472761512 | 2.19E-73 | 1.98E-73 |
| 101118112 | MROH5 | -3.288632019 | 2.26E-141 | 3.09E-141 |
| 101118115 | MCF2L | -3.61982865 | 0 | 0 |
| 101118117 | GALR2 | -10.86890557 | 2.71E-135 | 3.60E-135 |
| 101118126 | IRX3 | -4.75022746 | 0 | 0 |
| 101118138 | EVL | -3.153080063 | 0 | 0 |
| 101118144 | RHOC | -2.248457708 | 0 | 0 |
| 101118154 | C1H3orf70 | -2.653939563 | 0 | 0 |
| 101118164 | LOC101118164 | -8.379096318 | 1.44E-220 | 2.67E-220 |
| 101118176 | PARVB | -2.39667725 | 0 | 0 |
| 101118364 | FUCA2 | -2.769263659 | 0 | 0 |
| 101118378 | IRX5 | -6.787528919 | 7.19E-179 | 1.15E-178 |
| 101118417 | FAM167A | -5.665211247 | 0 | 0 |
| 101118435 | KCNH3 | -5.587330101 | 0 | 0 |
| 101118466 | ST8SIA6 | 3.206061912 | 0 | 0 |
| 101118510 | LOC101118510 | -7.581126959 | 5.51E-85 | 5.41E-85 |
| 101118551 | PLD3 | -2.323092364 | 0 | 0 |
| 101118587 | GK | -3.31075295 | 0 | 0 |
| 101118598 | NOL10 | -2.552392323 | 0 | 0 |
| 101118602 | LCN9 | 8.971754902 | 0 | 0 |
| 101118683 | LBX2 | 7.007529124 | 1.45E-118 | 1.77E-118 |
| 101118704 | SCML4 | -2.60962859 | 0 | 0 |
| 101118730 | PITPNM2 | -2.909931285 | 0 | 0 |
| 101118766 | STEAP3 | 2.646852711 | 7.33E-81 | 7.00E-81 |
| 101118813 | SHANK1 | 4.54771181 | 9.51E-154 | 1.38E-153 |
| 101118823 | ARMC12 | -2.252532905 | 5.24E-308 | 1.25E-307 |
| 101118871 | PALM3 | -2.918338508 | 0 | 0 |
| 101118903 | CRYBB1 | 4.962140013 | 0 | 0 |
| 101118937 | SYN3 | -4.886118494 | 7.52E-203 | 1.31E-202 |
| 101118946 | UNC5A | 3.781503648 | 4.81E-210 | 8.61E-210 |
| 101118952 | GNA15 | 2.278933075 | 1.35E-237 | 2.66E-237 |
| 101118984 | MPPED2 | -3.998719637 | 0 | 0 |
| 101118990 | LOC101118990 | -2.695675711 | 0 | 0 |
| 101118994 | SCAMP5 | 2.131393461 | 2.60E-133 | 3.42E-133 |
| 101118999 | LOC101118999 | -2.278517831 | 0 | 0 |
| 101119068 | GCNT7 | -3.785057845 | 1.93E-157 | 2.83E-157 |
| 101119074 | CLEC11A | 3.096349045 | 6.61E-71 | 5.85E-71 |
| 101119104 | RORC | 4.396121623 | 0 | 0 |
| 101119138 | SH3TC1 | 2.456166534 | 5.27E-199 | 9.07E-199 |
| 101119167 | SLC25A47 | -2.332755667 | 4.92E-58 | 3.87E-58 |
| 101119193 | PDIA6 | -2.391629257 | 0 | 0 |
| 101119302 | CCDC151 | -2.760925427 | 7.00E-262 | 1.49E-261 |
| 101119307 | CFI | 4.330357442 | 0 | 0 |
| 101119339 | LHFPL5 | -3.859962654 | 7.95E-174 | 1.25E-173 |
| 101119369 | PDE1A | 2.503350698 | 1.36E-100 | 1.48E-100 |
| 101119383 | KIF5A | -2.697185089 | 8.48E-95 | 8.90E-95 |
| 101119394 | GPAT3 | -2.364501717 | 2.74E-152 | 3.95E-152 |
| 101119401 | COL22A1 | 6.739565487 | 0 | 0 |
| 101119422 | PDZD2 | -2.03873124 | 2.52E-185 | 4.14E-185 |
| 101119425 | WDR25 | -2.541422269 | 9.16E-99 | 9.87E-99 |
| 101119448 | SERINC2 | 3.960296915 | 0 | 0 |
| 101119496 | SMPD3 | -3.796023272 | 0 | 0 |
| 101119513 | SHISA5 | -2.891284975 | 0 | 0 |
| 101119530 | LOC101119530 | -5.089377079 | 0 | 0 |
| 101119553 | SDHAF3 | 2.628570084 | 7.80E-38 | 4.87E-38 |
| 101119596 | KCNQ1 | -2.631347031 | 7.98E-102 | 8.78E-102 |
| 101119662 | SYNE4 | -2.38338535 | 0 | 0 |
| 101119679 | LOC101119679 | -2.395310603 | 6.20E-75 | 5.67E-75 |
| 101119694 | RASEF | -2.182436203 | 5.43E-290 | 1.24E-289 |
| 101119711 | CLEC12B | -7.319602439 | 5.29E-108 | 6.05E-108 |
| 101119723 | PCYOX1L | -2.69902108 | 1.77E-233 | 3.43E-233 |
| 101119737 | NPTX1 | -3.271349165 | 8.60E-115 | 1.03E-114 |
| 101119743 | ARHGEF16 | 2.004178294 | 2.52E-117 | 3.05E-117 |
| 101119787 | RET | -5.17948031 | 0 | 0 |
| 101119822 | KCTD8 | -8.845000385 | 1.43E-264 | 3.07E-264 |
| 101119848 | ITGA2 | 4.219718568 | 0 | 0 |
| 101119855 | LY6G5B | 10.797852 | 0 | 0 |
| 101119942 | PACSIN1 | 2.610453805 | 0 | 0 |
| 101119957 | CCL21 | -2.284984242 | 1.91E-23 | 9.60E-24 |
| 101120004 | PRDM16 | 2.822721697 | 8.53E-276 | 1.88E-275 |
| 101120006 | MPP7 | -2.369529048 | 4.53E-239 | 8.96E-239 |
| 101120058 | ADAM28 | 10.82283871 | 0 | 0 |
| 101120081 | SPOCK1 | -3.924535845 | 0 | 0 |
| 101120123 | SLC5A3 | 2.070926743 | 0 | 0 |
| 101120172 | NDRG1 | 3.70454836 | 0 | 0 |
| 101120179 | LOC101120179 | 3.359618553 | 0 | 0 |
| 101120216 | TMPRSS2 | 2.625897365 | 0 | 0 |
| 101120263 | TSHZ2 | 4.260522975 | 0 | 0 |
| 101120280 | FBXW12 | -4.94973534 | 0 | 0 |
| 101120313 | LCN10 | 10.14637033 | 0 | 0 |
| 101120414 | SPTB | -6.492877093 | 0 | 0 |
| 101120418 | WISP1 | 2.514874195 | 0 | 0 |
| 101120425 | KCNAB3 | -3.510233985 | 9.74E-139 | 1.32E-138 |
| 101120442 | KREMEN1 | 2.553249993 | 0 | 0 |
| 101120455 | LOC101120455 | -3.169067857 | 0 | 0 |
| 101120499 | SMPDL3A | 2.202969268 | 6.08E-117 | 7.35E-117 |
| 101120527 | STRA6 | 2.609668084 | 8.06E-253 | 1.66E-252 |
| 101120530 | SPINK8 | -7.559493431 | 0 | 0 |
| 101120553 | ZDHHC9 | 4.733931709 | 0 | 0 |
| 101120587 | TMEM98 | 3.507002832 | 0 | 0 |
| 101120631 | RASSF4 | -2.060767211 | 0 | 0 |
| 101120639 | SLC46A2 | -6.190092222 | 0 | 0 |
| 101120665 | MAPK10 | -4.000877612 | 6.02E-109 | 6.92E-109 |
| 101120732 | LOC101120732 | -2.172018676 | 4.73E-237 | 9.28E-237 |
| 101120761 | TLCD1 | -2.980454763 | 1.47E-120 | 1.82E-120 |
| 101120781 | BDKRB2 | 2.673875519 | 5.08E-173 | 7.98E-173 |
| 101120797 | LOC101120797 | 5.52722453 | 1.67E-186 | 2.75E-186 |
| 101120824 | AKR1B1 | -4.53877157 | 0 | 0 |
| 101120830 | RGL3 | -4.351068148 | 0 | 0 |
| 101120832 | ETNPPL | -6.186919956 | 0 | 0 |
| 101120850 | LOC101120850 | -2.613306482 | 0 | 0 |
| 101120887 | ITGB5 | 3.694999105 | 0 | 0 |
| 101120891 | TMEM47 | 2.806026279 | 0 | 0 |
| 101120908 | SEMA4F | 2.339049196 | 0 | 0 |
| 101120942 | ADGRG1 | 3.651332634 | 0 | 0 |
| 101120982 | SBK1 | -2.623594422 | 3.62E-32 | 2.09E-32 |
| 101121049 | PPT1 | -2.017983102 | 0 | 0 |
| 101121083 | FAM19A5 | -3.475319159 | 1.22E-159 | 1.82E-159 |
| 101121187 | TEDDM1 | 7.536187661 | 0 | 0 |
| 101121243 | ZMAT1 | -2.333082031 | 4.65E-125 | 5.87E-125 |
| 101121265 | GABRA2 | 5.905861651 | 3.62E-129 | 4.66E-129 |
| 101121283 | DLGAP4 | -2.524827821 | 0 | 0 |
| 101121332 | TRIB2 | 2.232146089 | 0 | 0 |
| 101121367 | TMED6 | -2.149076009 | 0 | 0 |
| 101121383 | LRRC8B | 4.007157593 | 0 | 0 |
| 101121387 | LOC101121387 | -3.716965731 | 0 | 0 |
| 101121397 | LRRC3B | -3.667263267 | 0 | 0 |
| 101121427 | PLEKHH1 | -2.358145651 | 0 | 0 |
| 101121440 | NMNAT2 | -3.561586413 | 0 | 0 |
| 101121521 | C7H14orf105 | 4.006320665 | 0 | 0 |
| 101121541 | CMIP | 2.8116231 | 1.19E-284 | 2.70E-284 |
| 101121552 | AK7 | 4.528344055 | 5.84E-306 | 1.38E-305 |
| 101121563 | LOC101121563 | -2.023789257 | 0 | 0 |
| 101121628 | MSMO1 | 2.048372743 | 0 | 0 |
| 101121631 | NTRK3 | 3.894604968 | 0 | 0 |
| 101121638 | TMEM132A | -3.509599002 | 0 | 0 |
| 101121643 | LOC101121643 | -3.677917074 | 4.45E-52 | 3.28E-52 |
| 101121659 | TTC39B | -2.595916448 | 0 | 0 |
| 101121663 | PRPS2 | -2.100862032 | 0 | 0 |
| 101121665 | TMEM187 | -2.661252037 | 4.00E-224 | 7.54E-224 |
| 101121743 | PDE9A | 3.569609528 | 0 | 0 |
| 101121750 | LOC101121750 | -6.232516822 | 2.48E-19 | 1.16E-19 |
| 101121758 | GLIPR1 | -3.316415044 | 0 | 0 |
| 101121820 | LOC101121820 | -2.046859377 | 0 | 0 |
| 101121831 | MECR | -2.220354183 | 0 | 0 |
| 101121924 | FOXRED2 | -2.280152114 | 0 | 0 |
| 101121944 | ADCY8 | 5.025760529 | 0 | 0 |
| 101121981 | PXDC1 | 2.668441443 | 5.59E-95 | 5.88E-95 |
| 101121985 | OLFML3 | -3.343634773 | 0 | 0 |
| 101122041 | MRC2 | 4.294189627 | 0 | 0 |
| 101122042 | GPR153 | 2.703618125 | 0 | 0 |
| 101122156 | ARHGDIG | 2.665328634 | 3.30E-25 | 1.71E-25 |
| 101122213 | MMP15 | 2.322696515 | 2.04E-83 | 1.98E-83 |
| 101122247 | APOBR | -2.025593404 | 0 | 0 |
| 101122272 | SLC25A40 | -2.507073098 | 1.04E-153 | 1.50E-153 |
| 101122318 | SLF2 | -2.022368209 | 0 | 0 |
| 101122398 | LOC101122398 | -3.828096073 | 0 | 0 |
| 101122440 | DENND4A | 2.094139255 | 5.28E-284 | 1.19E-283 |
| 101122449 | FBXO47 | -2.6786517 | 2.80E-159 | 4.16E-159 |
| 101122455 | CCNY | 2.117312977 | 0 | 0 |
| 101122497 | SIK1 | 2.062464548 | 2.88E-135 | 3.83E-135 |
| 101122507 | FAM49A | 2.789470884 | 2.82E-158 | 4.16E-158 |
| 101122516 | RARRES2 | 2.783690468 | 0 | 0 |
| 101122548 | LOC101122548 | -3.798774724 | 9.67E-168 | 1.49E-167 |
| 101122594 | STRA8 | 7.543196328 | 5.75E-124 | 7.22E-124 |
| 101122606 | ST3GAL1 | 3.615869626 | 0 | 0 |
| 101122639 | TWIST2 | -3.450381257 | 1.25E-65 | 1.05E-65 |
| 101122645 | LOC101122645 | -2.091160973 | 3.95E-09 | 1.42E-09 |
| 101122661 | RHOU | 2.877881376 | 0 | 0 |
| 101122673 | ABCD1 | -3.840990784 | 0 | 0 |
| 101122713 | FAM129A | -2.929969333 | 0 | 0 |
| 101122717 | LOC101122717 | -2.207128272 | 1.06E-166 | 1.63E-166 |
| 101122795 | ENPP3 | -3.612850098 | 0 | 0 |
| 101122804 | CFAP74 | 3.480648333 | 0 | 0 |
| 101122814 | HPD | 10.22752305 | 9.84E-97 | 1.05E-96 |
| 101122835 | ADAMTSL1 | -3.05526848 | 7.29E-224 | 1.37E-223 |
| 101122836 | NUDT11 | -5.5442338 | 0 | 0 |
| 101122839 | ATP11C | -2.840579006 | 0 | 0 |
| 101122905 | DUSP8 | -2.807368007 | 6.94E-63 | 5.69E-63 |
| 101122914 | TMEM8A | 3.217158783 | 0 | 0 |
| 101122960 | CACNB1 | -2.549490035 | 9.40E-232 | 1.82E-231 |
| 101122993 | LTBP3 | 2.684213936 | 0 | 0 |
| 101123015 | MED12 | -2.059530715 | 0 | 0 |
| 101123048 | ENPP1 | 2.749995106 | 0 | 0 |
| 101123095 | LOC101123095 | -6.714115468 | 0 | 0 |
| 101123150 | SEMA5A | 3.230853582 | 0 | 0 |
| 101123195 | ALPL | 2.058546445 | 0 | 0 |
| 101123278 | ST6GALNAC6 | 2.713749818 | 0 | 0 |
| 101123284 | RAB3IP | -2.103450085 | 0 | 0 |
| 101123294 | SLC22A4 | -4.212508563 | 0 | 0 |
| 101123345 | MED12L | -2.012386336 | 3.28E-257 | 6.88E-257 |
| 101123368 | LOC101123368 | -2.453752666 | 0 | 0 |
| 101123371 | FAM131B | -3.406252014 | 0 | 0 |
| 101123393 | SLC19A2 | -2.241869589 | 0 | 0 |
| 101123425 | SV2A | -3.907757136 | 6.67E-212 | 1.20E-211 |
| 101123427 | IVL | -9.610896324 | 0 | 0 |
| 101123437 | AZGP1 | -2.723901104 | 1.61E-290 | 3.69E-290 |
| 101123476 | ATP6V0A1 | -2.308708383 | 0 | 0 |
| 101123484 | SAMHD1 | 2.106651677 | 0 | 0 |
| 101123576 | ATP2C2 | 2.260896788 | 5.42E-47 | 3.79E-47 |
| 101123594 | SEMA4A | -2.051341655 | 2.78E-248 | 5.68E-248 |
| 101123612 | LOC101123612 | 3.818373354 | 2.57E-223 | 4.83E-223 |
| 101123651 | LOC101123651 | -2.322069257 | 0 | 0 |
| 105601862 | GPR75 | 4.875751936 | 5.36E-157 | 7.87E-157 |
| 105602015 | LOC105602015 | -7.445711943 | 0 | 0 |
| 105602028 | LOC105602028 | -3.219300398 | 3.46E-164 | 5.24E-164 |
| 105602415 | C1QTNF5 | -3.481196762 | 0 | 0 |
| 105602432 | LOC105602432 | -2.693764337 | 0 | 0 |
| 105602646 | LOC105602646 | -4.859250223 | 0 | 0 |
| 105602979 | LOC105602979 | -6.676559639 | 0 | 0 |
| 105603000 | GGT1 | -3.611998169 | 0 | 0 |
| 105603154 | LOC105603154 | 3.586827041 | 2.13E-221 | 3.97E-221 |
| 105603738 | DEFB110 | 3.21002107 | 0 | 0 |
| 105603814 | LOC105603814 | -5.837262933 | 3.15E-42 | 2.08E-42 |
| 105604152 | LOC105604152 | -2.972745203 | 1.92E-70 | 1.69E-70 |
| 105604590 | HSBP1L1 | -2.869722739 | 0 | 0 |
| 105604792 | LOC105604792 | 9.3217709 | 0 | 0 |
| 105604882 | LOC105604882 | -2.034394929 | 1.50E-42 | 1.00E-42 |
| 105605056 | LOC105605056 | -5.928790484 | 0 | 0 |
| 105605116 | LOC105605116 | -8.520494912 | 0 | 0 |
| 105605547 | PRRG3 | -3.385936604 | 0 | 0 |
| 105605624 | NXT2 | -2.207397748 | 2.16E-210 | 3.87E-210 |
| 105605702 | MAMLD1 | 2.244373693 | 1.55E-267 | 3.35E-267 |
| 105605834 | LOC105605834 | -2.247067068 | 0 | 0 |
| 105605950 | LOC105605950 | -8.859761831 | 0 | 0 |
| 105605969 | LOC105605969 | -2.662079469 | 8.23E-71 | 7.28E-71 |
| 105606076 | LOC105606076 | 2.43203121 | 3.40E-228 | 6.50E-228 |
| 105606122 | LOC105606122 | -3.530646697 | 1.24E-165 | 1.89E-165 |
| 105606694 | LOC105606694 | -8.983417947 | 0 | 0 |
| 105606698 | LOC105606698 | 7.717113615 | 0 | 0 |
| 105607151 | C2H9orf153 | 3.32938092 | 0 | 0 |
| 105608211 | RAB42 | 3.967322802 | 0 | 0 |
| 105608552 | DEFB134 | -6.096661814 | 0 | 0 |
| 105608895 | LOC105608895 | -4.155361219 | 0 | 0 |
| 105609445 | TGFBR3L | 3.395690007 | 0 | 0 |
| 105609446 | CTXN1 | -3.222121651 | 1.68E-66 | 1.43E-66 |
| 105610450 | NPW | 3.829191883 | 3.03E-13 | 1.24E-13 |
| 105610494 | LOC105610494 | -2.674725662 | 8.21E-159 | 1.21E-158 |
| 105611318 | LOC105611318 | 4.19798599 | 0 | 0 |
| 105611550 | LOC105611550 | 4.289100485 | 0 | 0 |
| 105613233 | LOC105613233 | 9.562006134 | 0 | 0 |
| 105613399 | LOC105613399 | -3.492080163 | 0 | 0 |
| 105613402 | WFDC9 | -3.656678249 | 0 | 0 |
| 105613453 | LOC105613453 | -13.70135425 | 0 | 0 |
| 105613766 | LOC105613766 | -3.742154463 | 0 | 0 |
| 105613767 | RNASE9 | -5.980828241 | 0 | 0 |
| 105613768 | LOC105613768 | -7.090012672 | 0 | 0 |
| 105615088 | LOC105615088 | -4.782671252 | 7.54E-71 | 6.67E-71 |
| 105615499 | LOC105615499 | -2.22048794 | 5.53E-97 | 5.90E-97 |
| 105615508 | LOC105615508 | -2.987361661 | 1.28E-258 | 2.70E-258 |
| 105615865 | LOC105615865 | -5.321543332 | 0 | 0 |
| 105615953 | LOC105615953 | -5.16248652 | 0 | 0 |
| 105616123 | LOC105616123 | -6.336034335 | 0 | 0 |
| 105616470 | CACNG5 | 4.362274296 | 1.03E-227 | 1.97E-227 |
| 105616540 | LOC105616540 | -2.414394084 | 5.17E-75 | 4.73E-75 |
| 105616741 | LOC105616741 | -3.154885943 | 1.02E-53 | 7.68E-54 |
| 105616801 | LOC105616801 | 8.796564482 | 0 | 0 |
| 106990097 | RPL10 | -2.632308916 | 0 | 0 |
| 106990147 | LTB4R2 | 5.166592248 | 2.97E-222 | 5.55E-222 |
| 106990163 | LOC106990163 | -5.134613397 | 0 | 0 |
| 106990245 | SH3GL2 | -4.343008305 | 0 | 0 |
| 106990358 | LOC106990358 | -2.477524916 | 2.83E-79 | 2.68E-79 |
| 106991069 | LOC106991069 | -4.032055997 | 1.29E-23 | 6.49E-24 |
| 106991207 | LOC106991207 | -2.93275652 | 0 | 0 |
| 106991424 | LOC106991424 | 3.003221282 | 1.30E-36 | 8.01E-37 |
| 106991447 | LOC106991447 | 2.304497173 | 0 | 0 |
| 106991450 | LOC106991450 | -5.610237023 | 0 | 0 |
| 106991619 | LOC106991619 | -3.329863219 | 2.47E-64 | 2.05E-64 |
| 106991631 | LOC106991631 | -4.31422556 | 1.29E-74 | 1.17E-74 |
| 106991725 | LOC106991725 | -4.859225847 | 3.17E-61 | 2.55E-61 |
| 106991729 | BDKRB1 | 3.987908472 | 0 | 0 |
| 106991730 | KIF26A | -2.286297572 | 1.87E-98 | 2.01E-98 |
| 106991749 | LOC106991749 | -2.885847633 | 1.65E-118 | 2.01E-118 |
| 106991839 | LBHD1 | -4.069787322 | 3.57E-30 | 2.00E-30 |
| 106991896 | LOC106991896 | -2.700305988 | 5.72E-30 | 3.20E-30 |
| BGI_novel_G000011 | BGI_novel_G000011 | -2.067767452 | 6.78E-143 | 9.35E-143 |
| BGI_novel_G000014 | BGI_novel_G000014 | -4.733073502 | 9.85E-115 | 1.17E-114 |
| BGI_novel_G000015 | BGI_novel_G000015 | -3.13797224 | 0 | 0 |
| BGI_novel_G000016 | BGI_novel_G000016 | 10.58502587 | 1.07E-231 | 2.06E-231 |
| BGI_novel_G000022 | BGI_novel_G000022 | -5.731252811 | 0 | 0 |
| BGI_novel_G000032 | BGI_novel_G000032 | -2.711897587 | 8.36E-34 | 4.95E-34 |
| BGI_novel_G000037 | BGI_novel_G000037 | -2.958411631 | 3.02E-248 | 6.16E-248 |
| BGI_novel_G000043 | BGI_novel_G000043 | -6.91845697 | 6.81E-90 | 6.91E-90 |
| BGI_novel_G000044 | BGI_novel_G000044 | -10.21799296 | 5.28E-96 | 5.59E-96 |
| BGI_novel_G000047 | BGI_novel_G000047 | 3.473865057 | 0 | 0 |
| BGI_novel_G000097 | BGI_novel_G000097 | -2.584770836 | 1.70E-85 | 1.68E-85 |
| BGI_novel_G000098 | BGI_novel_G000098 | -4.058668597 | 0 | 0 |
| BGI_novel_G000107 | BGI_novel_G000107 | 2.572309064 | 0 | 0 |
| BGI_novel_G000118 | BGI_novel_G000118 | -3.37817913 | 0 | 0 |
| BGI_novel_G000119 | BGI_novel_G000119 | 6.587840817 | 0 | 0 |
| BGI_novel_G000122 | BGI_novel_G000122 | -3.384022253 | 0 | 0 |
| BGI_novel_G000133 | BGI_novel_G000133 | -2.960541872 | 5.76E-104 | 6.42E-104 |
| BGI_novel_G000149 | BGI_novel_G000149 | -2.246811843 | 2.99E-47 | 2.10E-47 |
| BGI_novel_G000154 | BGI_novel_G000154 | -6.355556834 | 0 | 0 |
| BGI_novel_G000178 | BGI_novel_G000178 | -2.893845463 | 6.65E-126 | 8.43E-126 |
| BGI_novel_G000187 | BGI_novel_G000187 | -4.481548273 | 9.60E-288 | 2.18E-287 |
| BGI_novel_G000189 | BGI_novel_G000189 | -3.03614759 | 4.28E-65 | 3.58E-65 |
| BGI_novel_G000191 | BGI_novel_G000191 | 2.143881409 | 0 | 0 |
| BGI_novel_G000200 | BGI_novel_G000200 | -2.190077499 | 6.54E-279 | 1.45E-278 |
| BGI_novel_G000202 | BGI_novel_G000202 | -2.87834643 | 0 | 0 |
| BGI_novel_G000204 | BGI_novel_G000204 | -3.118643473 | 8.70E-138 | 1.17E-137 |
| BGI_novel_G000211 | BGI_novel_G000211 | -2.643108416 | 1.69E-25 | 8.80E-26 |
| BGI_novel_G000231 | BGI_novel_G000231 | -7.924487501 | 6.79E-70 | 5.94E-70 |
| BGI_novel_G000235 | BGI_novel_G000235 | -6.707925896 | 1.02E-219 | 1.89E-219 |
| BGI_novel_G000240 | BGI_novel_G000240 | -4.52852165 | 4.15E-265 | 8.91E-265 |
| BGI_novel_G000241 | BGI_novel_G000241 | -3.501497712 | 0 | 0 |
| BGI_novel_G000246 | BGI_novel_G000246 | -2.462211459 | 9.95E-51 | 7.26E-51 |
| BGI_novel_G000248 | BGI_novel_G000248 | -4.566503622 | 0 | 0 |
| BGI_novel_G000253 | BGI_novel_G000253 | -10.09819814 | 4.50E-90 | 4.57E-90 |
| BGI_novel_G000255 | BGI_novel_G000255 | -8.383220787 | 0 | 0 |
| BGI_novel_G000269 | BGI_novel_G000269 | 3.53388959 | 0 | 0 |
| BGI_novel_G000270 | BGI_novel_G000270 | 3.348406112 | 0 | 0 |
| BGI_novel_G000273 | BGI_novel_G000273 | 4.898912865 | 0 | 0 |
| BGI_novel_G000277 | BGI_novel_G000277 | -3.259776934 | 0 | 0 |
| BGI_novel_G000284 | BGI_novel_G000284 | -2.325040737 | 4.74E-114 | 5.61E-114 |
| BGI_novel_G000290 | BGI_novel_G000290 | -4.687054933 | 1.71E-165 | 2.62E-165 |
| BGI_novel_G000300 | BGI_novel_G000300 | -2.493326864 | 1.46E-153 | 2.11E-153 |
| BGI_novel_G000318 | BGI_novel_G000318 | -3.047249446 | 3.92E-98 | 4.20E-98 |
| BGI_novel_G000329 | BGI_novel_G000329 | -2.698630035 | 3.38E-275 | 7.43E-275 |
| BGI_novel_G000330 | BGI_novel_G000330 | -4.199494071 | 0 | 0 |
| BGI_novel_G000332 | BGI_novel_G000332 | -2.947054258 | 3.38E-77 | 3.15E-77 |
| BGI_novel_G000334 | BGI_novel_G000334 | -3.200435964 | 8.78E-127 | 1.12E-126 |
| BGI_novel_G000339 | BGI_novel_G000339 | 14.12535945 | 0 | 0 |
| BGI_novel_G000340 | BGI_novel_G000340 | 8.612351829 | 0 | 0 |
| BGI_novel_G000342 | BGI_novel_G000342 | 11.95248924 | 3.19E-237 | 6.26E-237 |
| BGI_novel_G000343 | BGI_novel_G000343 | 7.086415547 | 0 | 0 |
| BGI_novel_G000344 | BGI_novel_G000344 | 12.54433865 | 0 | 0 |
| BGI_novel_G000345 | BGI_novel_G000345 | 11.7748308 | 1.16E-216 | 2.13E-216 |
| BGI_novel_G000346 | BGI_novel_G000346 | 7.61633587 | 6.36E-283 | 1.43E-282 |
| BGI_novel_G000347 | BGI_novel_G000347 | 9.707916217 | 0 | 0 |
| BGI_novel_G000356 | BGI_novel_G000356 | 11.61281396 | 2.12E-199 | 3.64E-199 |
| BGI_novel_G000357 | BGI_novel_G000357 | 9.29406647 | 0 | 0 |
| BGI_novel_G000358 | BGI_novel_G000358 | 8.033013987 | 4.09E-275 | 9.00E-275 |
| BGI_novel_G000360 | BGI_novel_G000360 | 12.84454531 | 0 | 0 |
| BGI_novel_G000362 | BGI_novel_G000362 | 16.92309565 | 0 | 0 |
| BGI_novel_G000363 | BGI_novel_G000363 | 12.46135344 | 0 | 0 |
| BGI_novel_G000374 | BGI_novel_G000374 | -6.683178231 | 1.69E-96 | 1.79E-96 |
| BGI_novel_G000377 | BGI_novel_G000377 | -3.59616113 | 5.84E-208 | 1.04E-207 |
| BGI_novel_G000381 | BGI_novel_G000381 | -2.6293374 | 0 | 0 |
| BGI_novel_G000382 | BGI_novel_G000382 | -10.95598801 | 1.38E-141 | 1.89E-141 |
| BGI_novel_G000395 | BGI_novel_G000395 | 10.14981393 | 0 | 0 |
| BGI_novel_G000396 | BGI_novel_G000396 | 5.04353778 | 0 | 0 |
| BGI_novel_G000407 | BGI_novel_G000407 | -3.073390609 | 0 | 0 |
| BGI_novel_G000409 | BGI_novel_G000409 | -2.640685013 | 6.39E-149 | 9.07E-149 |
| BGI_novel_G000429 | BGI_novel_G000429 | -5.148886455 | 4.24E-43 | 2.83E-43 |
| BGI_novel_G000447 | BGI_novel_G000447 | -2.062454004 | 1.66E-47 | 1.17E-47 |
| BGI_novel_G000448 | BGI_novel_G000448 | -5.459191467 | 4.29E-81 | 4.10E-81 |
| BGI_novel_G000470 | BGI_novel_G000470 | -12.28621902 | 2.82E-280 | 6.28E-280 |
| BGI_novel_G000471 | BGI_novel_G000471 | -3.615322641 | 2.12E-221 | 3.94E-221 |
| BGI_novel_G000490 | BGI_novel_G000490 | -2.653252116 | 3.50E-181 | 5.68E-181 |
| BGI_novel_G000496 | BGI_novel_G000496 | 5.335942125 | 5.30E-211 | 9.51E-211 |
| BGI_novel_G000500 | BGI_novel_G000500 | -4.479377528 | 1.10E-120 | 1.36E-120 |
| BGI_novel_G000510 | BGI_novel_G000510 | -5.509753956 | 2.31E-65 | 1.94E-65 |
| BGI_novel_G000513 | BGI_novel_G000513 | -2.473973885 | 9.51E-115 | 1.13E-114 |
| BGI_novel_G000544 | BGI_novel_G000544 | -3.287614042 | 0 | 0 |
| BGI_novel_G000547 | BGI_novel_G000547 | -3.02399791 | 0 | 0 |
| BGI_novel_G000560 | BGI_novel_G000560 | -2.130647302 | 1.98E-126 | 2.52E-126 |
| BGI_novel_G000569 | BGI_novel_G000569 | -2.240737329 | 9.12E-50 | 6.59E-50 |
| BGI_novel_G000573 | BGI_novel_G000573 | -8.307385703 | 0 | 0 |
| BGI_novel_G000574 | BGI_novel_G000574 | -6.299654842 | 0 | 0 |
| BGI_novel_G000576 | BGI_novel_G000576 | -2.136353688 | 5.22E-279 | 1.16E-278 |
| BGI_novel_G000578 | BGI_novel_G000578 | -4.440614243 | 0 | 0 |
| BGI_novel_G000618 | BGI_novel_G000618 | -5.710321239 | 0 | 0 |
| BGI_novel_G000624 | BGI_novel_G000624 | -3.840199399 | 4.13E-63 | 3.40E-63 |
| BGI_novel_G000626 | BGI_novel_G000626 | -4.627703055 | 3.93E-178 | 6.30E-178 |
| BGI_novel_G000632 | BGI_novel_G000632 | -7.503990039 | 0 | 0 |
| BGI_novel_G000637 | BGI_novel_G000637 | -2.811053054 | 1.78E-11 | 6.90E-12 |
| BGI_novel_G000651 | BGI_novel_G000651 | 4.418652089 | 4.97E-169 | 7.70E-169 |
| BGI_novel_G000657 | BGI_novel_G000657 | 4.922301287 | 8.98E-37 | 5.53E-37 |
| BGI_novel_G000659 | BGI_novel_G000659 | -9.026065944 | 4.62E-99 | 4.99E-99 |
| BGI_novel_G000662 | BGI_novel_G000662 | 7.246023582 | 2.75E-245 | 5.55E-245 |
| BGI_novel_G000663 | BGI_novel_G000663 | 4.525454493 | 1.87E-176 | 2.98E-176 |
| BGI_novel_G000665 | BGI_novel_G000665 | 11.70799465 | 2.25E-209 | 4.02E-209 |
| BGI_novel_G000666 | BGI_novel_G000666 | 9.043616673 | 1.43E-198 | 2.47E-198 |
| BGI_novel_G000667 | BGI_novel_G000667 | 11.24405141 | 7.15E-165 | 1.09E-164 |
| BGI_novel_G000669 | BGI_novel_G000669 | 5.948773377 | 0 | 0 |
| BGI_novel_G000670 | BGI_novel_G000670 | 6.386611667 | 0 | 0 |
| BGI_novel_G000684 | BGI_novel_G000684 | -2.441087545 | 0 | 0 |
| BGI_novel_G000695 | BGI_novel_G000695 | -10.3638868 | 9.97E-104 | 1.11E-103 |
| BGI_novel_G000711 | BGI_novel_G000711 | -10.34093434 | 0 | 0 |
| BGI_novel_G000718 | BGI_novel_G000718 | -2.40464969 | 1.01E-104 | 1.13E-104 |
| BGI_novel_G000762 | BGI_novel_G000762 | -10.71105726 | 1.48E-124 | 1.86E-124 |
| BGI_novel_G000790 | BGI_novel_G000790 | -3.181002663 | 2.34E-105 | 2.64E-105 |
| BGI_novel_G000801 | BGI_novel_G000801 | -2.725207455 | 0 | 0 |
| BGI_novel_G000811 | BGI_novel_G000811 | -3.509144923 | 0 | 0 |
| BGI_novel_G000821 | BGI_novel_G000821 | 4.822627494 | 0 | 0 |
| BGI_novel_G000822 | BGI_novel_G000822 | 5.058572758 | 0 | 0 |
| BGI_novel_G000823 | BGI_novel_G000823 | 4.410808576 | 0 | 0 |
| BGI_novel_G000824 | BGI_novel_G000824 | 4.801551847 | 8.82E-191 | 1.48E-190 |
| BGI_novel_G000825 | BGI_novel_G000825 | 4.708921679 | 0 | 0 |
| BGI_novel_G000826 | BGI_novel_G000826 | 3.518056711 | 2.49E-195 | 4.23E-195 |
| BGI_novel_G000829 | BGI_novel_G000829 | -10.89037021 | 8.08E-137 | 1.08E-136 |
| BGI_novel_G000830 | BGI_novel_G000830 | -6.340446501 | 0 | 0 |
| BGI_novel_G000831 | BGI_novel_G000831 | -6.348764656 | 0 | 0 |
| BGI_novel_G000832 | BGI_novel_G000832 | -7.965355492 | 0 | 0 |
| BGI_novel_G000838 | BGI_novel_G000838 | 7.025312955 | 2.36E-149 | 3.36E-149 |
| BGI_novel_G000844 | BGI_novel_G000844 | -3.664222835 | 1.83E-207 | 3.24E-207 |
| BGI_novel_G000845 | BGI_novel_G000845 | -3.114714918 | 7.15E-146 | 1.00E-145 |
| BGI_novel_G000848 | BGI_novel_G000848 | -7.834215035 | 6.56E-228 | 1.25E-227 |
| BGI_novel_G000851 | BGI_novel_G000851 | -2.680147194 | 1.73E-222 | 3.23E-222 |
| BGI_novel_G000870 | BGI_novel_G000870 | -8.355368714 | 0 | 0 |
| BGI_novel_G000894 | BGI_novel_G000894 | -3.813747007 | 1.67E-107 | 1.91E-107 |
| BGI_novel_G000898 | BGI_novel_G000898 | -2.771624983 | 0 | 0 |
| BGI_novel_G000909 | BGI_novel_G000909 | -3.098155073 | 1.89E-141 | 2.58E-141 |
| BGI_novel_G000912 | BGI_novel_G000912 | -7.274319214 | 6.12E-225 | 1.16E-224 |
| BGI_novel_G000913 | BGI_novel_G000913 | -3.73606971 | 0 | 0 |
| BGI_novel_G000914 | BGI_novel_G000914 | -6.094361297 | 0 | 0 |
| BGI_novel_G000920 | BGI_novel_G000920 | -3.688715882 | 0 | 0 |
| BGI_novel_G000930 | BGI_novel_G000930 | -4.164162804 | 2.21E-197 | 3.78E-197 |
| BGI_novel_G000936 | BGI_novel_G000936 | -5.932734853 | 0 | 0 |
| BGI_novel_G000937 | BGI_novel_G000937 | -2.269736482 | 0 | 0 |
| BGI_novel_G000943 | BGI_novel_G000943 | -3.747039699 | 1.37E-139 | 1.86E-139 |
| BGI_novel_G000944 | BGI_novel_G000944 | -3.770329974 | 3.34E-157 | 4.91E-157 |
| BGI_novel_G000954 | BGI_novel_G000954 | -3.658239838 | 1.55E-106 | 1.76E-106 |
| BGI_novel_G000965 | BGI_novel_G000965 | -9.140612366 | 1.41E-105 | 1.60E-105 |
| BGI_novel_G000971 | BGI_novel_G000971 | -7.70413785 | 1.94E-91 | 1.98E-91 |
| BGI_novel_G000983 | BGI_novel_G000983 | 6.547072353 | 3.44E-213 | 6.21E-213 |
| BGI_novel_G000984 | BGI_novel_G000984 | 5.608517238 | 0 | 0 |
| BGI_novel_G000985 | BGI_novel_G000985 | 3.332989307 | 8.83E-220 | 1.64E-219 |
| BGI_novel_G000987 | BGI_novel_G000987 | 6.575446937 | 0 | 0 |
| BGI_novel_G000988 | BGI_novel_G000988 | 4.393681254 | 0 | 0 |
| BGI_novel_G000999 | BGI_novel_G000999 | -7.780679404 | 5.35E-283 | 1.20E-282 |
| BGI_novel_G001001 | BGI_novel_G001001 | -2.960778134 | 0 | 0 |
| BGI_novel_G001002 | BGI_novel_G001002 | 3.662183611 | 0 | 0 |
| BGI_novel_G001003 | BGI_novel_G001003 | 3.509818542 | 6.10E-125 | 7.69E-125 |
| BGI_novel_G001012 | BGI_novel_G001012 | 2.812141466 | 6.86E-65 | 5.73E-65 |
| BGI_novel_G001014 | BGI_novel_G001014 | 7.156173205 | 0 | 0 |
| BGI_novel_G001020 | BGI_novel_G001020 | -3.796048446 | 1.20E-166 | 1.85E-166 |
| BGI_novel_G001025 | BGI_novel_G001025 | 4.139351164 | 0 | 0 |
| BGI_novel_G001031 | BGI_novel_G001031 | 4.554297322 | 2.95E-49 | 2.12E-49 |
| BGI_novel_G001040 | BGI_novel_G001040 | -3.415750249 | 0 | 0 |
| BGI_novel_G001042 | BGI_novel_G001042 | -8.982808886 | 2.08E-191 | 3.49E-191 |
| BGI_novel_G001044 | BGI_novel_G001044 | 5.004308585 | 0 | 0 |
| BGI_novel_G001050 | BGI_novel_G001050 | -3.631854149 | 5.70E-208 | 1.01E-207 |
| BGI_novel_G001060 | BGI_novel_G001060 | 12.27628155 | 0 | 0 |
| BGI_novel_G001061 | BGI_novel_G001061 | 10.72620633 | 0 | 0 |
| BGI_novel_G001069 | BGI_novel_G001069 | -6.162459276 | 2.07E-35 | 1.25E-35 |
| BGI_novel_G001080 | BGI_novel_G001080 | -7.409690491 | 4.47E-114 | 5.29E-114 |
| BGI_novel_G001081 | BGI_novel_G001081 | -6.285229175 | 0 | 0 |
| BGI_novel_G001082 | BGI_novel_G001082 | -3.302088626 | 1.61E-95 | 1.69E-95 |
| BGI_novel_G001086 | BGI_novel_G001086 | -2.661576777 | 5.23E-174 | 8.26E-174 |
| BGI_novel_G001103 | BGI_novel_G001103 | -6.445926426 | 0 | 0 |
| BGI_novel_G001104 | BGI_novel_G001104 | 12.08547778 | 0 | 0 |
| BGI_novel_G001110 | BGI_novel_G001110 | 3.686938759 | 4.46E-206 | 7.88E-206 |
| BGI_novel_G001116 | BGI_novel_G001116 | -5.216586851 | 2.82E-200 | 4.86E-200 |
| BGI_novel_G001117 | BGI_novel_G001117 | -3.221670742 | 0 | 0 |
| BGI_novel_G001118 | BGI_novel_G001118 | -7.960355007 | 1.49E-158 | 2.20E-158 |
| BGI_novel_G001119 | BGI_novel_G001119 | -16.05851742 | 0 | 0 |
| BGI_novel_G001121 | BGI_novel_G001121 | 2.181413274 | 4.57E-24 | 2.33E-24 |
| BGI_novel_G001126 | BGI_novel_G001126 | 3.244087128 | 1.87E-92 | 1.93E-92 |
| BGI_novel_G001134 | BGI_novel_G001134 | -2.330826011 | 0 | 0 |
| BGI_novel_G001135 | BGI_novel_G001135 | -2.918022238 | 0 | 0 |
| BGI_novel_G001139 | BGI_novel_G001139 | 9.104826325 | 8.24E-53 | 6.14E-53 |
| BGI_novel_G001148 | BGI_novel_G001148 | -7.373062174 | 2.20E-75 | 2.02E-75 |
| BGI_novel_G001159 | BGI_novel_G001159 | -4.546729952 | 2.00E-85 | 1.97E-85 |
| BGI_novel_G001162 | BGI_novel_G001162 | -5.411436191 | 4.56E-304 | 1.08E-303 |
| BGI_novel_G001173 | BGI_novel_G001173 | -8.838715858 | 0 | 0 |
| BGI_novel_G001201 | BGI_novel_G001201 | 2.68377928 | 5.70E-43 | 3.81E-43 |
| BGI_novel_G001209 | BGI_novel_G001209 | -8.016396174 | 0 | 0 |
| BGI_novel_G001222 | BGI_novel_G001222 | -3.770776882 | 0 | 0 |
| BGI_novel_G001223 | BGI_novel_G001223 | -6.165642306 | 6.63E-74 | 6.01E-74 |
| BGI_novel_G001224 | BGI_novel_G001224 | -7.992476698 | 2.07E-259 | 4.38E-259 |
| BGI_novel_G001225 | BGI_novel_G001225 | -5.170240767 | 1.56E-59 | 1.24E-59 |
| BGI_novel_G001228 | BGI_novel_G001228 | -5.350352049 | 0 | 0 |
| BGI_novel_G001237 | BGI_novel_G001237 | -4.335894562 | 1.13E-63 | 9.36E-64 |
| BGI_novel_G001238 | BGI_novel_G001238 | -3.154782512 | 6.01E-240 | 1.19E-239 |
| BGI_novel_G001239 | BGI_novel_G001239 | -6.263854644 | 0 | 0 |
| BGI_novel_G001241 | BGI_novel_G001241 | -7.466794284 | 0 | 0 |
| BGI_novel_G001244 | BGI_novel_G001244 | -5.559867487 | 0 | 0 |
| BGI_novel_G001247 | BGI_novel_G001247 | -4.670411977 | 3.45E-307 | 8.18E-307 |
| BGI_novel_G001248 | BGI_novel_G001248 | -7.292794834 | 4.65E-266 | 1.00E-265 |
| BGI_novel_G001249 | BGI_novel_G001249 | -4.999950846 | 0 | 0 |
| BGI_novel_G001259 | BGI_novel_G001259 | -2.338566445 | 9.88E-244 | 1.98E-243 |
| BGI_novel_G001264 | BGI_novel_G001264 | -5.077574474 | 7.14E-100 | 7.75E-100 |
| BGI_novel_G001270 | BGI_novel_G001270 | -3.726881598 | 0 | 0 |
| BGI_novel_G001273 | BGI_novel_G001273 | -5.190868249 | 1.10E-259 | 2.34E-259 |
| BGI_novel_G001274 | BGI_novel_G001274 | -5.477626422 | 4.46E-262 | 9.50E-262 |
| BGI_novel_G001275 | BGI_novel_G001275 | -3.843941405 | 3.96E-250 | 8.13E-250 |
| BGI_novel_G001277 | BGI_novel_G001277 | -3.265667477 | 0 | 0 |
| BGI_novel_G001284 | BGI_novel_G001284 | 11.57193004 | 0 | 0 |
| BGI_novel_G001297 | BGI_novel_G001297 | -5.443321269 | 5.52E-155 | 8.04E-155 |
| BGI_novel_G001298 | BGI_novel_G001298 | -7.737560851 | 2.86E-93 | 2.97E-93 |
| BGI_novel_G001299 | BGI_novel_G001299 | -7.009322681 | 2.86E-16 | 1.25E-16 |
| BGI_novel_G001308 | BGI_novel_G001308 | -7.987689273 | 4.38E-108 | 5.02E-108 |
| BGI_novel_G001315 | BGI_novel_G001315 | -13.18235365 | 0 | 0 |
| BGI_novel_G001317 | BGI_novel_G001317 | -12.46207398 | 2.82E-306 | 6.69E-306 |
| BGI_novel_G001319 | BGI_novel_G001319 | -3.83981998 | 0 | 0 |
| BGI_novel_G001326 | BGI_novel_G001326 | -13.32975678 | 0 | 0 |
| BGI_novel_G001327 | BGI_novel_G001327 | -3.922065241 | 0 | 0 |
| BGI_novel_G001337 | BGI_novel_G001337 | -2.772901374 | 1.99E-20 | 9.47E-21 |
| BGI_novel_G001344 | BGI_novel_G001344 | -2.045654374 | 0 | 0 |
| BGI_novel_G001348 | BGI_novel_G001348 | -5.528940889 | 2.44E-226 | 4.64E-226 |
| BGI_novel_G001357 | BGI_novel_G001357 | -5.905771711 | 1.98E-72 | 1.78E-72 |
| BGI_novel_G001367 | BGI_novel_G001367 | -3.674960479 | 7.50E-186 | 1.23E-185 |
| BGI_novel_G001375 | BGI_novel_G001375 | -8.613866651 | 0 | 0 |
| BGI_novel_G001380 | BGI_novel_G001380 | 2.85348319 | 0 | 0 |
| BGI_novel_G001381 | BGI_novel_G001381 | 9.858551589 | 0 | 0 |
| BGI_novel_G001382 | BGI_novel_G001382 | 7.150164875 | 0 | 0 |
| BGI_novel_G001432 | BGI_novel_G001432 | -4.094692664 | 7.59E-35 | 4.56E-35 |
| BGI_novel_G001434 | BGI_novel_G001434 | -2.126827206 | 3.76E-27 | 2.01E-27 |
| BGI_novel_G001443 | BGI_novel_G001443 | -3.328796309 | 0 | 0 |
| BGI_novel_G001456 | BGI_novel_G001456 | -9.027564849 | 1.01E-181 | 1.64E-181 |
| BGI_novel_G001457 | BGI_novel_G001457 | -8.596155169 | 8.18E-40 | 5.25E-40 |
| BGI_novel_G001468 | BGI_novel_G001468 | 3.189827229 | 8.34E-208 | 1.48E-207 |
| BGI_novel_G001469 | BGI_novel_G001469 | 2.81564857 | 0 | 0 |
| BGI_novel_G001470 | BGI_novel_G001470 | 2.937010748 | 1.05E-261 | 2.23E-261 |
| BGI_novel_G001472 | BGI_novel_G001472 | 2.665328634 | 5.20E-119 | 6.35E-119 |
| BGI_novel_G001473 | BGI_novel_G001473 | 2.823926123 | 0 | 0 |
| BGI_novel_G001474 | BGI_novel_G001474 | 2.664727092 | 0 | 0 |
| BGI_novel_G001475 | BGI_novel_G001475 | 2.709079266 | 0 | 0 |
| BGI_novel_G001488 | BGI_novel_G001488 | -4.607039161 | 4.79E-73 | 4.31E-73 |
| BGI_novel_G001490 | BGI_novel_G001490 | -5.567647436 | 0 | 0 |
| BGI_novel_G001496 | BGI_novel_G001496 | -4.109956819 | 1.10E-53 | 8.28E-54 |
| BGI_novel_G001511 | BGI_novel_G001511 | -7.603610974 | 7.97E-23 | 3.96E-23 |
| BGI_novel_G001521 | BGI_novel_G001521 | -3.206966045 | 9.20E-45 | 6.27E-45 |
| BGI_novel_G001528 | BGI_novel_G001528 | -4.055934771 | 2.36E-241 | 4.71E-241 |
| BGI_novel_G001533 | BGI_novel_G001533 | 6.031453735 | 0 | 0 |
| BGI_novel_G001534 | BGI_novel_G001534 | 6.545925283 | 0 | 0 |
| BGI_novel_G001541 | BGI_novel_G001541 | 3.46368368 | 0 | 0 |
| BGI_novel_G001555 | BGI_novel_G001555 | -3.352132908 | 0 | 0 |
| BGI_novel_G001556 | BGI_novel_G001556 | -3.269387152 | 2.18E-305 | 5.14E-305 |
| BGI_novel_G001559 | BGI_novel_G001559 | -3.474829418 | 0 | 0 |
| BGI_novel_G001560 | BGI_novel_G001560 | 7.814859959 | 0 | 0 |
| BGI_novel_G001561 | BGI_novel_G001561 | 3.418726402 | 0 | 0 |
| BGI_novel_G001562 | BGI_novel_G001562 | 5.274043164 | 0 | 0 |
| BGI_novel_G001563 | BGI_novel_G001563 | -2.94751032 | 0 | 0 |
| BGI_novel_G001566 | BGI_novel_G001566 | -3.122417387 | 1.35E-111 | 1.58E-111 |
| BGI_novel_G001573 | BGI_novel_G001573 | -5.610164797 | 5.01E-180 | 8.09E-180 |
| BGI_novel_G001579 | BGI_novel_G001579 | 4.689520359 | 7.12E-166 | 1.09E-165 |
| BGI_novel_G001642 | BGI_novel_G001642 | -6.503805397 | 0 | 0 |
| BGI_novel_G001643 | BGI_novel_G001643 | -7.775241721 | 0 | 0 |
| BGI_novel_G001644 | BGI_novel_G001644 | -8.490377676 | 0 | 0 |
| BGI_novel_G001647 | BGI_novel_G001647 | -7.35390068 | 1.55E-218 | 2.87E-218 |
| BGI_novel_G001648 | BGI_novel_G001648 | -3.208138634 | 2.85E-83 | 2.76E-83 |
| BGI_novel_G001653 | BGI_novel_G001653 | 2.191712447 | 0 | 0 |
| BGI_novel_G001671 | BGI_novel_G001671 | -2.756086594 | 1.91E-222 | 3.58E-222 |
| BGI_novel_G001699 | BGI_novel_G001699 | 6.225120559 | 2.73E-19 | 1.27E-19 |
| BGI_novel_G001729 | BGI_novel_G001729 | -6.227663091 | 0 | 0 |
| BGI_novel_G001735 | BGI_novel_G001735 | 2.581264369 | 3.81E-10 | 1.42E-10 |
| BGI_novel_G001750 | BGI_novel_G001750 | -2.190008138 | 0 | 0 |
| BGI_novel_G001761 | BGI_novel_G001761 | -2.769656406 | 0 | 0 |
| BGI_novel_G001766 | BGI_novel_G001766 | -7.349715044 | 0 | 0 |
| BGI_novel_G001767 | BGI_novel_G001767 | 4.752101399 | 3.50E-185 | 5.74E-185 |
| BGI_novel_G001768 | BGI_novel_G001768 | 9.481576306 | 0 | 0 |
| BGI_novel_G001770 | BGI_novel_G001770 | -2.373395544 | 5.73E-53 | 4.28E-53 |
| BGI_novel_G001783 | BGI_novel_G001783 | -3.794965539 | 2.78E-157 | 4.09E-157 |
| BGI_novel_G001801 | BGI_novel_G001801 | -9.552766106 | 0 | 0 |
| BGI_novel_G001802 | BGI_novel_G001802 | -7.466942582 | 0 | 0 |
| BGI_novel_G001809 | BGI_novel_G001809 | -3.099149838 | 0 | 0 |
| BGI_novel_G001815 | BGI_novel_G001815 | -3.202350998 | 1.44E-47 | 1.01E-47 |
| BGI_novel_G001821 | BGI_novel_G001821 | -3.208308891 | 2.42E-173 | 3.81E-173 |
| BGI_novel_G001834 | BGI_novel_G001834 | -4.025300386 | 6.15E-101 | 6.74E-101 |
| BGI_novel_G001836 | BGI_novel_G001836 | -4.552870762 | 0 | 0 |
| BGI_novel_G001849 | BGI_novel_G001849 | -2.008571039 | 8.34E-137 | 1.12E-136 |
| BGI_novel_G001884 | BGI_novel_G001884 | -2.166575268 | 9.45E-162 | 1.42E-161 |
| BGI_novel_G001889 | BGI_novel_G001889 | 8.707039437 | 1.95E-42 | 1.29E-42 |
| BGI_novel_G001911 | BGI_novel_G001911 | -3.967000985 | 1.15E-89 | 1.16E-89 |
| BGI_novel_G001916 | BGI_novel_G001916 | -3.153778424 | 4.21E-203 | 7.37E-203 |
| BGI_novel_G001917 | BGI_novel_G001917 | -3.949563937 | 3.48E-69 | 3.02E-69 |
| BGI_novel_G001935 | BGI_novel_G001935 | -5.396015554 | 1.70E-21 | 8.26E-22 |
| BGI_novel_G001948 | BGI_novel_G001948 | -8.630861998 | 0 | 0 |
| BGI_novel_G001971 | BGI_novel_G001971 | 4.455733487 | 2.25E-11 | 8.70E-12 |
| BGI_novel_G001976 | BGI_novel_G001976 | -8.332642277 | 2.17E-34 | 1.30E-34 |
| BGI_novel_G001989 | BGI_novel_G001989 | 2.398400312 | 2.41E-06 | 7.81E-07 |
| BGI_novel_G002039 | BGI_novel_G002039 | -5.426909562 | 0 | 0 |
| BGI_novel_G002056 | BGI_novel_G002056 | -5.644630503 | 1.23E-164 | 1.88E-164 |
| BGI_novel_G002062 | BGI_novel_G002062 | -2.528443109 | 2.26E-44 | 1.53E-44 |
| BGI_novel_G002067 | BGI_novel_G002067 | -3.647554321 | 1.78E-11 | 6.90E-12 |
| BGI_novel_G002085 | BGI_novel_G002085 | 2.663815909 | 6.82E-132 | 8.89E-132 |
| BGI_novel_G002086 | BGI_novel_G002086 | 2.663815909 | 6.82E-132 | 8.89E-132 |
