# Supplementary material for: Study on the region-specific expression of epididymis mRNA in the rams
Source: PLoS One. 2021 Jan 25;16(1):e0245933. doi: 10.1371/journal.pone.0245933 (PMC7833257; doi:10.1371/journal.pone.0245933)
Supplement: S7 Table — (DOCX) [file pone.0245933.s011.docx]

# S7 Table. The DEGs list between corpus and cauda

| **Gene ID** | **Other Gene ID** | **log2(Corpus/Cauda)** | **Pvalue(Corpus -vs- Cauda)** | **Qvalue(Corpus -vs- Cauda)** |
| --- | --- | --- | --- | --- |
| 443013 | PTGER2 | -2.110765402 | 6.15E-80 | 9.74E-80 |
| 443019 | QPCT | 6.273510311 | 0 | 0 |
| 443038 | CAPN3 | -2.685604372 | 0 | 0 |
| 443079 | KRT25 | 2.261631526 | 0 | 0 |
| 443098 | SLC17A5 | -2.134169069 | 0 | 0 |
| 443209 | ITGB6 | 2.98830436 | 0 | 0 |
| 443246 | SLC1A3 | 4.372763558 | 5.37E-94 | 9.49E-94 |
| 443265 | TIE1 | 2.172446225 | 5.11E-199 | 1.55E-198 |
| 443305 | SELP | -5.941080873 | 0 | 0 |
| 443306 | FGF2 | -2.846388714 | 1.95E-16 | 1.33E-16 |
| 443323 | FST | -3.064255697 | 8.81E-99 | 1.61E-98 |
| 443361 | NPY1R | 2.557658533 | 0 | 0 |
| 443384 | ATP1B1 | -2.824055611 | 0 | 0 |
| 443388 | SERPINA1 | -12.11604314 | 0 | 0 |
| 443408 | LPL | -2.323905962 | 7.07E-72 | 1.04E-71 |
| 443419 | STAT5A | -2.046489163 | 0 | 0 |
| 443488 | SCNN1A | -4.856189698 | 0 | 0 |
| 443517 | CNN1 | -2.055685322 | 0 | 0 |
| 492300 | SLC5A1 | -6.748909123 | 0 | 0 |
| 493773 | NPPC | -4.446965546 | 3.70E-63 | 5.02E-63 |
| 554254 | TLR7 | -3.168847841 | 0 | 0 |
| 554321 | LTF | 4.623990102 | 0 | 0 |
| 554328 | PAX4 | 7.367650635 | 9.36E-39 | 9.57E-39 |
| 554333 | MAFB | 2.198203607 | 8.60E-139 | 2.01E-138 |
| 641305 | PI3 | -2.664562092 | 1.71E-93 | 3.00E-93 |
| 780451 | CES5A | 2.097756118 | 0 | 0 |
| 780454 | FURIN | -2.149243802 | 0 | 0 |
| 780475 | CLDN2 | 6.729651492 | 1.46E-248 | 5.41E-248 |
| 780497 | TFF3 | -7.088511237 | 3.95E-32 | 3.67E-32 |
| 780509 | MB | -3.259974441 | 7.52E-223 | 2.51E-222 |
| 100034665 | NOX4 | 7.799661213 | 0 | 0 |
| 100127212 | ST3GAL4 | -2.588764395 | 0 | 0 |
| 100141299 | CD9 | -2.69489312 | 0 | 0 |
| 100145866 | VDAC1 | -2.279029521 | 0 | 0 |
| 100145875 | PSPH | -3.022088244 | 0 | 0 |
| 100145881 | ARL4A | 2.828206479 | 1.12E-212 | 3.58E-212 |
| 100170316 | ADRA1D | -2.716681166 | 6.52E-92 | 1.14E-91 |
| 100170317 | ADRA2A | -3.998300468 | 2.50E-269 | 9.82E-269 |
| 100170324 | DPP4 | -2.803958025 | 0 | 0 |
| 100171395 | RGN | -3.529654628 | 0 | 0 |
| 100174904 | GATM | -2.513493923 | 0 | 0 |
| 100189620 | ME1 | -2.35437261 | 3.11E-240 | 1.11E-239 |
| 100192422 | MMP9 | 3.458624295 | 7.66E-86 | 1.28E-85 |
| 100196903 | IDO1 | 3.853270005 | 1.25E-58 | 1.61E-58 |
| 100272215 | MLPH | -6.302689476 | 1.28E-129 | 2.84E-129 |
| 100302062 | REM1 | -2.292182474 | 0 | 0 |
| 100302354 | SDR16C5 | -3.501998285 | 1.06E-31 | 9.80E-32 |
| 100302549 | SLC1A5 | 3.507287453 | 0 | 0 |
| 100307044 | FHL1 | -2.625122279 | 0 | 0 |
| 100462650 | SLC26A3 | -2.110943217 | 0 | 0 |
| 100505405 | PEG3 | 2.394475975 | 4.20E-226 | 1.41E-225 |
| 100526668 | TTPA | -3.825948859 | 0 | 0 |
| 100820756 | SRD5A2 | 7.019792002 | 0 | 0 |
| 101101956 | FGF13 | -3.475269231 | 0 | 0 |
| 101101968 | CAPG | -4.598382821 | 0 | 0 |
| 101101969 | RASGRP3 | 3.140877549 | 0 | 0 |
| 101102077 | SELL | -8.142214404 | 0 | 0 |
| 101102136 | NCS1 | -3.058557768 | 0 | 0 |
| 101102178 | LOC101102178 | 3.322156181 | 0 | 0 |
| 101102187 | PRSS23 | 2.89026641 | 0 | 0 |
| 101102225 | VAMP5 | -2.039875017 | 0 | 0 |
| 101102355 | EVA1C | -3.249847038 | 1.05E-257 | 4.01E-257 |
| 101102364 | PIGQ | -2.034646962 | 0 | 0 |
| 101102457 | TSPAN7 | -2.027615195 | 8.24E-223 | 2.75E-222 |
| 101102594 | GMPR | -2.212895197 | 2.62E-91 | 4.54E-91 |
| 101102647 | SCG5 | -4.101565931 | 0 | 0 |
| 101102704 | NHSL2 | -2.067713533 | 0 | 0 |
| 101102747 | ZNF648 | -3.010726803 | 1.42E-141 | 3.36E-141 |
| 101102875 | GALNT3 | -2.581966144 | 0 | 0 |
| 101102909 | TTC9 | 2.057327961 | 4.58E-127 | 1.00E-126 |
| 101102920 | WFDC1 | -2.295646991 | 0 | 0 |
| 101102986 | RORA | -2.087420902 | 5.72E-99 | 1.05E-98 |
| 101103020 | KCNK5 | 4.060539929 | 0 | 0 |
| 101103054 | IL1R1 | -2.417544784 | 2.80E-248 | 1.03E-247 |
| 101103057 | GRHL1 | 2.224069235 | 0 | 0 |
| 101103059 | C3H12orf75 | -2.369014266 | 0 | 0 |
| 101103077 | PPP1R1B | -2.848419563 | 0 | 0 |
| 101103094 | DCLK2 | -2.501954172 | 2.23E-164 | 5.92E-164 |
| 101103096 | LOC101103096 | 2.52320401 | 0 | 0 |
| 101103160 | MAP3K9 | 2.193160008 | 5.67E-226 | 1.91E-225 |
| 101103188 | TDRD9 | 2.077115047 | 0 | 0 |
| 101103192 | FGD2 | 2.917993901 | 2.61E-118 | 5.44E-118 |
| 101103211 | C2H9orf91 | -2.192511179 | 1.93E-138 | 4.50E-138 |
| 101103268 | WDR66 | -2.735787533 | 1.17E-158 | 3.05E-158 |
| 101103269 | RLBP1 | -7.700931733 | 0 | 0 |
| 101103276 | CTPS1 | -2.422345072 | 0 | 0 |
| 101103323 | GJD2 | 5.81226125 | 1.26E-162 | 3.34E-162 |
| 101103365 | PLSCR4 | -2.318367176 | 0 | 0 |
| 101103367 | TFR2 | -2.99226846 | 0 | 0 |
| 101103405 | SCEL | -3.489630608 | 5.38E-276 | 2.17E-275 |
| 101103617 | SHE | -2.018352685 | 1.28E-280 | 5.21E-280 |
| 101103622 | ACE2 | -2.248371484 | 2.52E-99 | 4.63E-99 |
| 101103631 | LOC101103631 | -2.355471816 | 0 | 0 |
| 101103681 | SYTL2 | 3.361057634 | 0 | 0 |
| 101103696 | TSPEAR | -6.69579585 | 0 | 0 |
| 101103697 | AQP7 | 8.151941613 | 0 | 0 |
| 101103729 | VGLL2 | 9.451929642 | 0 | 0 |
| 101103733 | SLCO5A1 | 6.707614455 | 1.72E-196 | 5.17E-196 |
| 101103739 | TCAP | -4.498178772 | 0 | 0 |
| 101103801 | ACSS3 | -3.511727152 | 0 | 0 |
| 101103826 | PNMT | -3.974823056 | 0 | 0 |
| 101103838 | PIWIL4 | 4.608169645 | 1.78E-262 | 6.87E-262 |
| 101103884 | LCLAT1 | -5.261800856 | 0 | 0 |
| 101103891 | PRRT4 | -2.38601786 | 2.19E-126 | 4.78E-126 |
| 101103892 | SMIM3 | -2.818637434 | 0 | 0 |
| 101103926 | KIF7 | -2.158913809 | 3.31E-101 | 6.16E-101 |
| 101103967 | GDF3 | 8.164742051 | 0 | 0 |
| 101104031 | ROGDI | -2.084734239 | 2.60E-237 | 9.14E-237 |
| 101104034 | PPL | -2.533128509 | 0 | 0 |
| 101104040 | ZNF385D | 4.185477998 | 1.90E-255 | 7.17E-255 |
| 101104057 | ATP6V0A4 | 3.218281739 | 0 | 0 |
| 101104144 | IMPDH1 | -2.657258895 | 0 | 0 |
| 101104163 | SHISA6 | -5.647018266 | 0 | 0 |
| 101104179 | DNAH5 | 5.709632318 | 0 | 0 |
| 101104222 | LOC101104222 | -7.084709667 | 0 | 0 |
| 101104256 | RTN4RL1 | -2.556249916 | 0 | 0 |
| 101104287 | SERPINB8 | -2.526343546 | 1.72E-102 | 3.21E-102 |
| 101104292 | SEC14L5 | -4.843035202 | 2.62E-138 | 6.10E-138 |
| 101104311 | TMEM213 | 5.849889073 | 0 | 0 |
| 101104335 | HSPB6 | -2.316834165 | 1.70E-129 | 3.77E-129 |
| 101104467 | TNNT3 | -5.134416617 | 0 | 0 |
| 101104474 | CPM | 6.503252898 | 0 | 0 |
| 101104513 | NPL | -3.682848024 | 0 | 0 |
| 101104541 | S100A5 | -2.823305732 | 4.08E-28 | 3.53E-28 |
| 101104600 | UPK2 | -5.404855991 | 0 | 0 |
| 101104644 | RNF186 | 4.582798773 | 0 | 0 |
| 101104663 | SLBP | -2.171309134 | 0 | 0 |
| 101104674 | ARHGAP44 | -5.270267999 | 1.62E-251 | 6.08E-251 |
| 101104679 | TAT | -4.911293362 | 0 | 0 |
| 101104908 | MTHFD2L | -2.126232865 | 2.05E-106 | 3.94E-106 |
| 101104952 | PCOLCE2 | -2.347119577 | 2.74E-71 | 3.99E-71 |
| 101105033 | SLC6A6 | -2.438183398 | 0 | 0 |
| 101105051 | DIRAS2 | -4.368453719 | 5.80E-123 | 1.24E-122 |
| 101105058 | SMPDL3B | 4.385046132 | 1.81E-124 | 3.89E-124 |
| 101105108 | CSPG4 | -2.856623735 | 7.31E-263 | 2.82E-262 |
| 101105140 | ZNF185 | 3.014845178 | 0 | 0 |
| 101105168 | DIAPH3 | 2.37394658 | 6.01E-236 | 2.10E-235 |
| 101105208 | LOC101105208 | -2.718008175 | 1.38E-113 | 2.78E-113 |
| 101105239 | HOXA10 | -4.8364225 | 0 | 0 |
| 101105265 | LOC101105265 | -6.048922609 | 8.70E-156 | 2.23E-155 |
| 101105293 | S100A2 | -2.411299974 | 0 | 0 |
| 101105382 | CLDN16 | 2.633769468 | 1.00E-173 | 2.76E-173 |
| 101105400 | HSD17B6 | -3.289725379 | 0 | 0 |
| 101105432 | IRF8 | -2.129583228 | 0 | 0 |
| 101105461 | GNAL | 5.584748757 | 8.73E-105 | 1.66E-104 |
| 101105518 | CST7 | -2.876925571 | 7.57E-67 | 1.06E-66 |
| 101105537 | PRSS16 | 8.328714972 | 6.40E-265 | 2.49E-264 |
| 101105541 | ECM1 | -4.035325653 | 0 | 0 |
| 101105583 | GSDMA | -2.950766641 | 3.23E-58 | 4.15E-58 |
| 101105592 | JPH2 | -2.227532467 | 2.12E-92 | 3.71E-92 |
| 101105619 | PLS1 | 2.092652752 | 0 | 0 |
| 101105659 | FGFRL1 | 2.491982892 | 2.43E-120 | 5.12E-120 |
| 101105760 | CCDC170 | -2.858296074 | 1.49E-156 | 3.82E-156 |
| 101105792 | DLGAP1 | 3.093883117 | 2.33E-114 | 4.73E-114 |
| 101105855 | PTPN9 | -2.345296873 | 0 | 0 |
| 101105908 | STRIP2 | 2.450093437 | 1.16E-74 | 1.75E-74 |
| 101105968 | IMPA2 | 2.04511725 | 0 | 0 |
| 101105976 | LOC101105976 | 6.539688199 | 0 | 0 |
| 101106130 | CFAP57 | 2.364283784 | 5.39E-179 | 1.52E-178 |
| 101106194 | SDCBP2 | -2.267223534 | 5.88E-69 | 8.39E-69 |
| 101106213 | AMIGO1 | 2.018944619 | 0 | 0 |
| 101106245 | LOC101106245 | -2.141502533 | 1.78E-209 | 5.60E-209 |
| 101106273 | ZACN | 5.462511019 | 6.67E-260 | 2.55E-259 |
| 101106315 | LOC101106315 | -3.401416027 | 0 | 0 |
| 101106349 | UCN3 | -7.162511818 | 1.35E-17 | 9.49E-18 |
| 101106370 | SRGAP3 | -4.753586393 | 0 | 0 |
| 101106384 | LOC101106384 | 2.217166975 | 0 | 0 |
| 101106480 | PLA1A | -3.355156896 | 0 | 0 |
| 101106534 | LOC101106534 | -4.264501696 | 0 | 0 |
| 101106548 | HDDC3 | -2.365611254 | 1.86E-182 | 5.33E-182 |
| 101106577 | LOC101106577 | -3.164927432 | 5.25E-151 | 1.31E-150 |
| 101106601 | WDFY2 | -2.245926149 | 0 | 0 |
| 101106664 | PLA2G2F | 3.305858045 | 0 | 0 |
| 101106686 | SEL1L3 | 4.016386234 | 0 | 0 |
| 101106798 | MAN2A2 | -3.837204214 | 0 | 0 |
| 101106808 | BCAR3 | -2.607929045 | 2.11E-156 | 5.41E-156 |
| 101106812 | ST3GAL6 | 3.664409086 | 0 | 0 |
| 101106830 | GLIPR2 | -3.419697148 | 0 | 0 |
| 101106849 | FSIP1 | 2.547092053 | 3.12E-277 | 1.27E-276 |
| 101106884 | C18H15orf39 | -2.087704136 | 1.60E-240 | 5.73E-240 |
| 101106905 | NINJ1 | 2.170552308 | 0 | 0 |
| 101106916 | PLA2G2D | 2.796846197 | 2.24E-188 | 6.56E-188 |
| 101106919 | LOC101106919 | -2.492513872 | 0 | 0 |
| 101106938 | PLPP2 | -2.286078514 | 8.05E-48 | 9.31E-48 |
| 101107008 | SLC6A15 | 9.729983565 | 1.42E-293 | 6.03E-293 |
| 101107028 | IPCEF1 | 4.22253237 | 3.87E-238 | 1.37E-237 |
| 101107049 | FES | -2.84938225 | 0 | 0 |
| 101107141 | CADPS | 4.710226129 | 0 | 0 |
| 101107151 | GRK5 | -3.772362769 | 0 | 0 |
| 101107225 | GAS2L1 | -2.206553836 | 0 | 0 |
| 101107260 | LOC101107260 | -6.424455339 | 0 | 0 |
| 101107276 | EPDR1 | -2.663863161 | 0 | 0 |
| 101107321 | ATP13A5 | -3.516311688 | 1.37E-144 | 3.29E-144 |
| 101107327 | SRGN | -3.576875266 | 0 | 0 |
| 101107370 | TEN1 | 2.665475865 | 4.43E-123 | 9.46E-123 |
| 101107422 | CD99L2 | -2.110335294 | 2.47E-265 | 9.61E-265 |
| 101107611 | NBEA | -2.127414567 | 0 | 0 |
| 101107719 | KISS1 | 4.166910813 | 1.15E-83 | 1.89E-83 |
| 101107790 | SALL2 | -4.438969678 | 0 | 0 |
| 101107799 | CPQ | -3.172232619 | 0 | 0 |
| 101107841 | HKDC1 | -2.602628098 | 0 | 0 |
| 101107907 | C20H6orf136 | -12.22777022 | 2.21E-269 | 8.70E-269 |
| 101107949 | CERCAM | 2.172641164 | 0 | 0 |
| 101107979 | GOLT1A | 4.319019085 | 0 | 0 |
| 101108088 | DCDC2 | -3.834170806 | 0 | 0 |
| 101108169 | FAM3D | 3.842172456 | 0 | 0 |
| 101108229 | ART3 | -4.510226293 | 0 | 0 |
| 101108275 | ZKSCAN2 | -2.047334569 | 8.46E-103 | 1.59E-102 |
| 101108280 | RUNX1 | -2.302703849 | 0 | 0 |
| 101108409 | CDNF | -2.162270746 | 2.33E-89 | 3.98E-89 |
| 101108488 | SLC39A8 | 4.731053436 | 0 | 0 |
| 101108515 | ELOVL7 | -4.054083614 | 0 | 0 |
| 101108621 | TVP23A | 7.195589889 | 3.13E-68 | 4.45E-68 |
| 101108630 | PGM5 | -2.518713815 | 0 | 0 |
| 101108683 | TUB | -3.487607192 | 1.20E-56 | 1.51E-56 |
| 101108713 | TTC24 | 2.988813459 | 0 | 0 |
| 101108733 | MGAT4C | -8.159072734 | 0 | 0 |
| 101108745 | FUT5 | 2.238667794 | 0 | 0 |
| 101108750 | CXCL11 | -2.39468526 | 2.88E-26 | 2.40E-26 |
| 101108802 | GUSB | -2.419945182 | 0 | 0 |
| 101108816 | PTPRU | 2.820696118 | 0 | 0 |
| 101108820 | MGAT4A | -3.74981826 | 0 | 0 |
| 101108888 | LIMS2 | -2.006504775 | 4.43E-212 | 1.41E-211 |
| 101108954 | PEBP1 | -2.301457188 | 0 | 0 |
| 101109034 | DEFB129 | 4.316237292 | 0 | 0 |
| 101109035 | LOC101109035 | -2.861860383 | 1.38E-144 | 3.32E-144 |
| 101109049 | FADS1 | -2.373720253 | 0 | 0 |
| 101109058 | VEPH1 | -2.585769824 | 6.17E-129 | 1.36E-128 |
| 101109074 | FAM46B | -2.477015239 | 3.15E-90 | 5.40E-90 |
| 101109111 | LOC101109111 | -2.316546038 | 8.24E-95 | 1.46E-94 |
| 101109206 | MFRP | 3.35085984 | 0 | 0 |
| 101109219 | LOC101109219 | -3.714193122 | 2.59E-283 | 1.06E-282 |
| 101109293 | DEFB127 | -4.042527847 | 0 | 0 |
| 101109397 | LOC101109397 | 4.465341211 | 3.17E-190 | 9.33E-190 |
| 101109506 | MMD2 | 7.109892767 | 0 | 0 |
| 101109515 | CITED1 | -2.696284313 | 0 | 0 |
| 101109585 | TSPAN2 | -2.230431399 | 0 | 0 |
| 101109628 | DLX4 | -4.612298734 | 0 | 0 |
| 101109659 | ROR1 | -2.618191302 | 6.03E-167 | 1.61E-166 |
| 101109687 | PROKR1 | 5.163168411 | 0 | 0 |
| 101109730 | CES2 | 2.394800718 | 9.09E-55 | 1.13E-54 |
| 101109766 | SH2D4B | 2.253587725 | 2.82E-109 | 5.53E-109 |
| 101109833 | TMEM89 | 4.93639755 | 1.14E-44 | 1.27E-44 |
| 101109887 | TMEM181 | -2.325332681 | 1.93E-246 | 7.06E-246 |
| 101109910 | STC2 | 2.269998448 | 6.41E-160 | 1.67E-159 |
| 101109916 | TGM5 | -4.032984422 | 0 | 0 |
| 101109927 | DAGLA | 2.18317063 | 2.89E-122 | 6.13E-122 |
| 101109939 | LOC101109939 | -3.604464319 | 5.58E-105 | 1.06E-104 |
| 101109975 | TAGAP | -2.502183194 | 3.09E-118 | 6.42E-118 |
| 101110032 | L1CAM | -4.69299207 | 0 | 0 |
| 101110074 | PRKCA | -2.184225563 | 0 | 0 |
| 101110079 | DEFB115 | -2.62280761 | 0 | 0 |
| 101110105 | CRTAC1 | -3.258878607 | 4.35E-136 | 1.00E-135 |
| 101110152 | FARP1 | -2.354497981 | 0 | 0 |
| 101110189 | THRSP | -5.277535999 | 3.74E-190 | 1.10E-189 |
| 101110195 | LOC101110195 | 3.262227082 | 6.15E-21 | 4.65E-21 |
| 101110343 | CA8 | 2.12985877 | 4.51E-49 | 5.29E-49 |
| 101110391 | PAK3 | -2.751505103 | 0 | 0 |
| 101110407 | SARDH | -4.800683413 | 0 | 0 |
| 101110410 | PLEKHG6 | -2.164789309 | 0 | 0 |
| 101110427 | LOC101110427 | 2.454611232 | 1.96E-97 | 3.54E-97 |
| 101110433 | CUBN | 3.956106751 | 0 | 0 |
| 101110463 | RAVER2 | -2.534018013 | 0 | 0 |
| 101110562 | SDK1 | 2.372227091 | 1.00E-276 | 4.05E-276 |
| 101110610 | LOC101110610 | 3.861149592 | 0 | 0 |
| 101110626 | PFKFB4 | -2.203746285 | 0 | 0 |
| 101110651 | GPR155 | -2.055367019 | 0 | 0 |
| 101110712 | SERPINB1 | -2.956009963 | 0 | 0 |
| 101110739 | DGKK | -4.444794109 | 0 | 0 |
| 101110748 | GPRC5A | 6.200390875 | 1.14E-71 | 1.66E-71 |
| 101110769 | PKIA | 7.73545433 | 0 | 0 |
| 101110790 | ZBTB16 | -2.488680569 | 5.98E-52 | 7.20E-52 |
| 101110813 | CARD11 | 3.087319406 | 0 | 0 |
| 101110855 | LOC101110855 | -4.415779555 | 0 | 0 |
| 101110896 | NME9 | -4.567207373 | 2.20E-159 | 5.72E-159 |
| 101110897 | METRN | -3.076465458 | 6.79E-32 | 6.27E-32 |
| 101110915 | ENTPD2 | -2.682800384 | 2.05E-125 | 4.43E-125 |
| 101110974 | LOC101110974 | 4.861558646 | 0 | 0 |
| 101111031 | TEX26 | 5.24103286 | 1.43E-180 | 4.05E-180 |
| 101111056 | NT5DC2 | 2.367838991 | 0 | 0 |
| 101111105 | PM20D2 | 2.785594345 | 0 | 0 |
| 101111148 | FAM173A | -2.589542952 | 0 | 0 |
| 101111169 | ABCA2 | -3.831268321 | 0 | 0 |
| 101111237 | DNAJC6 | -4.70986113 | 0 | 0 |
| 101111242 | LOC101111242 | 6.145258214 | 0 | 0 |
| 101111294 | STMN2 | -2.748071585 | 2.00E-48 | 2.32E-48 |
| 101111297 | MEDAG | 2.247384175 | 0 | 0 |
| 101111308 | WFDC2 | -5.040754878 | 0 | 0 |
| 101111312 | CATSPERG | 2.906360723 | 0 | 0 |
| 101111381 | PM20D1 | 2.18916362 | 4.26E-44 | 4.69E-44 |
| 101111416 | HAGHL | 2.545600155 | 1.44E-229 | 4.91E-229 |
| 101111429 | C2H2orf88 | -2.883851177 | 3.66E-73 | 5.42E-73 |
| 101111442 | NT5DC3 | -2.223960104 | 0 | 0 |
| 101111505 | LOC101111505 | 6.52136005 | 5.38E-45 | 6.01E-45 |
| 101111562 | NXPH3 | 3.663206209 | 3.66E-282 | 1.50E-281 |
| 101111615 | S100A14 | -2.867277482 | 0 | 0 |
| 101111639 | CNR1 | 3.863116324 | 0 | 0 |
| 101111647 | CSRP1 | -2.265352508 | 0 | 0 |
| 101111650 | ID1 | -2.213137891 | 1.06E-71 | 1.55E-71 |
| 101111669 | LOC101111669 | 5.163168411 | 6.90E-78 | 1.07E-77 |
| 101111755 | TEKT2 | 2.099221495 | 4.52E-52 | 5.45E-52 |
| 101111810 | SLC22A2 | 6.504517185 | 0 | 0 |
| 101111824 | WFDC8 | 3.974281105 | 1.14E-291 | 4.82E-291 |
| 101111874 | KIAA1211L | -2.791314977 | 0 | 0 |
| 101111915 | LOC101111915 | 5.093787609 | 0 | 0 |
| 101111939 | GLIS3 | -5.297932028 | 0 | 0 |
| 101111950 | SLC3A1 | -7.958986298 | 0 | 0 |
| 101111972 | DUOX1 | 2.28429352 | 0 | 0 |
| 101111999 | TCN1 | 10.95403521 | 2.05E-284 | 8.47E-284 |
| 101112033 | GUCY2F | -2.487155314 | 0 | 0 |
| 101112075 | MMP28 | -2.105178643 | 1.49E-85 | 2.47E-85 |
| 101112081 | WFDC13 | 3.458855646 | 0 | 0 |
| 101112092 | TTC21A | 3.624804119 | 4.08E-158 | 1.06E-157 |
| 101112114 | SCTR | 8.005690188 | 0 | 0 |
| 101112133 | LYPD8 | 6.223993604 | 0 | 0 |
| 101112173 | GAS2 | 2.516960804 | 0 | 0 |
| 101112232 | MAP7 | 2.01982695 | 0 | 0 |
| 101112296 | LOC101112296 | -7.347925327 | 5.62E-180 | 1.59E-179 |
| 101112340 | SPINT4 | 5.866132752 | 0 | 0 |
| 101112346 | RCN1 | 2.834544715 | 0 | 0 |
| 101112377 | CFAP221 | 4.119663003 | 1.69E-267 | 6.59E-267 |
| 101112406 | RAC3 | -2.786632812 | 2.94E-94 | 5.20E-94 |
| 101112423 | GGT5 | 3.388953888 | 0 | 0 |
| 101112452 | NEFM | -2.162067791 | 0 | 0 |
| 101112518 | CSRNP1 | 2.008835258 | 2.75E-74 | 4.11E-74 |
| 101112591 | RYR1 | -2.436076892 | 0 | 0 |
| 101112606 | LOC101112606 | 5.761901023 | 0 | 0 |
| 101112618 | MME | -3.334336245 | 0 | 0 |
| 101112653 | IL20RA | -6.009333892 | 0 | 0 |
| 101112748 | PRDX6 | 2.887528659 | 0 | 0 |
| 101112771 | STMND1 | 3.946593317 | 2.21E-118 | 4.61E-118 |
| 101112780 | CSTA | 3.355813489 | 0 | 0 |
| 101112830 | UBL3 | -2.972468472 | 0 | 0 |
| 101112833 | RASL10B | -4.801027568 | 0 | 0 |
| 101112874 | FCRL3 | -3.592744146 | 0 | 0 |
| 101112882 | EPB41L5 | 2.469437605 | 0 | 0 |
| 101112941 | INPP5D | 2.613190951 | 0 | 0 |
| 101112958 | HPGD | -2.013150999 | 0 | 0 |
| 101112976 | CREB5 | 2.032673135 | 3.39E-205 | 1.05E-204 |
| 101113073 | LOC101113073 | 4.839468976 | 0 | 0 |
| 101113074 | PLCB2 | 3.402197354 | 0 | 0 |
| 101113126 | FMN2 | -7.172780154 | 1.08E-129 | 2.41E-129 |
| 101113147 | CYFIP2 | -2.492394363 | 0 | 0 |
| 101113149 | GPX4 | -2.166887564 | 0 | 0 |
| 101113165 | RBM17 | -4.572140815 | 0 | 0 |
| 101113199 | NKX3-1 | 3.120576535 | 7.11E-78 | 1.10E-77 |
| 101113259 | LOC101113259 | -4.544023461 | 1.08E-48 | 1.26E-48 |
| 101113331 | LOC101113331 | 4.753666453 | 0 | 0 |
| 101113357 | LOC101113357 | -2.068250569 | 2.67E-41 | 2.83E-41 |
| 101113394 | EXTL1 | -7.559516223 | 0 | 0 |
| 101113407 | MGAM | 8.472582753 | 5.61E-288 | 2.33E-287 |
| 101113409 | PPP2R2B | 2.214060493 | 3.07E-201 | 9.39E-201 |
| 101113410 | NIPAL4 | -2.307678186 | 3.76E-172 | 1.03E-171 |
| 101113512 | ITPKB | 2.045557305 | 0 | 0 |
| 101113556 | NUPR1 | -2.270950521 | 0 | 0 |
| 101113588 | UNC13A | 2.816157316 | 0 | 0 |
| 101113676 | CBARP | -2.185181461 | 2.14E-216 | 6.98E-216 |
| 101113693 | LOC101113693 | -5.22305336 | 7.34E-71 | 1.07E-70 |
| 101113728 | LOC101113728 | 2.885031481 | 0 | 0 |
| 101113761 | LOC101113761 | 4.380656417 | 9.18E-226 | 3.09E-225 |
| 101113764 | PAQR5 | 2.403211175 | 3.41E-131 | 7.64E-131 |
| 101113823 | LOC101113823 | 9.692989358 | 7.01E-145 | 1.69E-144 |
| 101113842 | TSPAN33 | 3.455414738 | 0 | 0 |
| 101113986 | PEBP4 | 3.468547448 | 0 | 0 |
| 101113990 | GALNT5 | -8.912812051 | 0 | 0 |
| 101114004 | SLC4A8 | -2.698830727 | 0 | 0 |
| 101114008 | ABO | 3.886571903 | 3.20E-275 | 1.29E-274 |
| 101114027 | PDE10A | -3.186883439 | 0 | 0 |
| 101114042 | LOC101114042 | 3.903498522 | 0 | 0 |
| 101114051 | THYN1 | 2.50046264 | 0 | 0 |
| 101114059 | ASB2 | 3.544751577 | 0 | 0 |
| 101114114 | RRAGD | -2.172531357 | 0 | 0 |
| 101114192 | C5H19orf24 | -2.817373297 | 7.99E-146 | 1.94E-145 |
| 101114255 | NCK2 | -2.714563986 | 0 | 0 |
| 101114256 | ACTG2 | -2.712915363 | 0 | 0 |
| 101114311 | LOC101114311 | 3.95563221 | 0 | 0 |
| 101114314 | CRIP3 | 2.692277677 | 6.70E-156 | 1.72E-155 |
| 101114341 | SYT2 | -2.602884438 | 1.44E-101 | 2.68E-101 |
| 101114470 | FAM174B | 2.949764773 | 8.46E-84 | 1.39E-83 |
| 101114541 | MFSD4 | 4.603101085 | 0 | 0 |
| 101114565 | KLHL31 | -4.258759564 | 0 | 0 |
| 101114590 | TSC22D3 | -3.451068356 | 0 | 0 |
| 101114598 | KCNIP3 | -12.00692617 | 0 | 0 |
| 101114681 | FAM229A | -2.126740687 | 3.56E-29 | 3.14E-29 |
| 101114849 | LOC101114849 | 2.001789523 | 0 | 0 |
| 101114853 | LOC101114853 | -2.759188712 | 1.26E-107 | 2.46E-107 |
| 101114925 | GPR146 | -2.888280465 | 1.04E-21 | 7.96E-22 |
| 101115012 | ASS1 | -5.338124353 | 0 | 0 |
| 101115020 | NOTCH1 | -2.486181934 | 4.71E-290 | 1.97E-289 |
| 101115022 | GRIP1 | -2.09551308 | 0 | 0 |
| 101115034 | DUOXA1 | 2.448929359 | 0 | 0 |
| 101115035 | THSD4 | 2.368869642 | 0 | 0 |
| 101115062 | MUC15 | 2.250194885 | 0 | 0 |
| 101115082 | GJA5 | -2.319443274 | 9.91E-173 | 2.72E-172 |
| 101115130 | GJB7 | -4.971338128 | 9.04E-231 | 3.10E-230 |
| 101115236 | LOC101115236 | -2.128929218 | 5.55E-168 | 1.49E-167 |
| 101115292 | ABCA5 | 3.805260119 | 0 | 0 |
| 101115303 | ABCC12 | 6.260313845 | 0 | 0 |
| 101115315 | MAML3 | -2.636322358 | 0 | 0 |
| 101115323 | PHACTR1 | -2.08998358 | 3.95E-115 | 8.07E-115 |
| 101115414 | EFNA3 | -2.406930546 | 2.40E-145 | 5.81E-145 |
| 101115627 | ITPKA | 4.733228089 | 1.81E-142 | 4.30E-142 |
| 101115696 | METTL7B | 2.599351356 | 0 | 0 |
| 101115726 | NEURL2 | -3.302261948 | 3.87E-46 | 4.38E-46 |
| 101115732 | LOC101115732 | 8.694650658 | 0 | 0 |
| 101115740 | LOC101115740 | 4.610627388 | 3.60E-74 | 5.39E-74 |
| 101115803 | CAB39L | -2.086029097 | 6.22E-235 | 2.16E-234 |
| 101115816 | LPCAT2 | -2.320558235 | 3.52E-133 | 8.00E-133 |
| 101115829 | DAO | -2.965826889 | 0 | 0 |
| 101115830 | CLMN | -2.467408674 | 0 | 0 |
| 101115870 | PDE3A | 2.502824099 | 4.54E-61 | 6.03E-61 |
| 101115959 | LOC101115959 | 3.910447592 | 0 | 0 |
| 101115961 | CNN2 | -2.16316034 | 0 | 0 |
| 101115963 | PLK5 | -2.043436658 | 0 | 0 |
| 101115988 | LOC101115988 | 8.102259163 | 0 | 0 |
| 101115996 | SLC44A4 | -3.010794232 | 0 | 0 |
| 101116029 | AIM1L | -6.718201785 | 3.51E-241 | 1.26E-240 |
| 101116051 | FABP6 | -6.999013086 | 0 | 0 |
| 101116069 | SLC16A6 | -4.569799818 | 0 | 0 |
| 101116106 | PDZK1 | 6.259462072 | 0 | 0 |
| 101116128 | FAM189A2 | -2.175956939 | 0 | 0 |
| 101116129 | RIPPLY1 | 8.614948994 | 0 | 0 |
| 101116157 | LOC101116157 | 4.079162436 | 1.26E-99 | 2.32E-99 |
| 101116204 | RNF122 | 2.070200788 | 3.18E-36 | 3.13E-36 |
| 101116286 | LOC101116286 | -2.750214732 | 3.33E-125 | 7.19E-125 |
| 101116428 | P2RX2 | -3.995409608 | 0 | 0 |
| 101116471 | PROM2 | -7.265817198 | 0 | 0 |
| 101116570 | LOC101116570 | -9.693652624 | 1.12E-141 | 2.65E-141 |
| 101116580 | SMOC2 | -2.144724553 | 0 | 0 |
| 101116664 | TNFRSF19 | -2.182590402 | 1.90E-195 | 5.69E-195 |
| 101116679 | LRCOL1 | -2.471554787 | 0 | 0 |
| 101116683 | CCK | -8.712708901 | 2.69E-82 | 4.35E-82 |
| 101116697 | LRP8 | -2.474497842 | 8.80E-246 | 3.22E-245 |
| 101116704 | SIT1 | 2.823813245 | 2.57E-180 | 7.28E-180 |
| 101116716 | SLC8A1 | 2.806718271 | 0 | 0 |
| 101116798 | SLC28A3 | 6.037781174 | 0 | 0 |
| 101116803 | FMNL2 | -2.54451107 | 0 | 0 |
| 101116804 | AFF2 | 2.390658187 | 0 | 0 |
| 101116818 | GPSM1 | -4.358587802 | 0 | 0 |
| 101116828 | LOC101116828 | 3.807232286 | 0 | 0 |
| 101116840 | THBS2 | 2.053266409 | 1.95E-82 | 3.16E-82 |
| 101116862 | LOC101116862 | 3.107990616 | 2.28E-213 | 7.30E-213 |
| 101116919 | SACS | -2.669330151 | 0 | 0 |
| 101116925 | ESPN | 2.275900189 | 0 | 0 |
| 101116939 | PSTPIP1 | 3.6105055 | 0 | 0 |
| 101116951 | TMC5 | 7.321387912 | 1.70E-109 | 3.35E-109 |
| 101117042 | HIP1 | -3.396097916 | 0 | 0 |
| 101117144 | LOC101117144 | -4.009769829 | 2.50E-105 | 4.77E-105 |
| 101117167 | SPTBN5 | 2.186366343 | 5.05E-161 | 1.32E-160 |
| 101117184 | LOC101117184 | 4.467652904 | 0 | 0 |
| 101117210 | GLIS1 | -3.985056802 | 1.40E-194 | 4.19E-194 |
| 101117223 | RNF128 | 2.446697202 | 0 | 0 |
| 101117226 | VGLL1 | -5.529083828 | 7.89E-88 | 1.33E-87 |
| 101117247 | FLT4 | -2.045020779 | 8.87E-176 | 2.46E-175 |
| 101117255 | JPH1 | -2.189069141 | 0 | 0 |
| 101117258 | SERPINF2 | 2.425414708 | 0 | 0 |
| 101117297 | CCL26 | -4.745946849 | 1.76E-110 | 3.48E-110 |
| 101117299 | LOC101117299 | -4.481544082 | 0 | 0 |
| 101117314 | SLC30A2 | -5.077622921 | 4.82E-149 | 1.19E-148 |
| 101117322 | QSOX2 | -2.484664316 | 0 | 0 |
| 101117380 | LOXL4 | -9.453188979 | 0 | 0 |
| 101117403 | B3GNT2 | -2.725815796 | 0 | 0 |
| 101117454 | BSN | 6.159990774 | 0 | 0 |
| 101117480 | KIAA1161 | 2.223733781 | 5.98E-215 | 1.94E-214 |
| 101117482 | LOC101117482 | 7.44797248 | 0 | 0 |
| 101117541 | SLC29A1 | -2.417331426 | 0 | 0 |
| 101117552 | MRO | 2.030343245 | 0 | 0 |
| 101117553 | CCBE1 | 3.217695289 | 8.95E-148 | 2.19E-147 |
| 101117562 | STAT4 | 3.43819758 | 1.38E-203 | 4.26E-203 |
| 101117587 | LOC101117587 | 6.187305481 | 0 | 0 |
| 101117636 | KLHL14 | -7.567693812 | 0 | 0 |
| 101117637 | P2RY14 | 2.101977758 | 0 | 0 |
| 101117670 | ARL9 | 2.695569472 | 1.47E-62 | 1.99E-62 |
| 101117691 | LOC101117691 | 3.477872793 | 0 | 0 |
| 101117712 | PATE2 | 2.493261403 | 0 | 0 |
| 101117736 | MAP7D3 | -2.983532899 | 0 | 0 |
| 101117815 | SH2B2 | -3.461695958 | 2.10E-283 | 8.67E-283 |
| 101117846 | MAN2B2 | 5.476409445 | 0 | 0 |
| 101117861 | CA6 | -7.698564719 | 1.72E-178 | 4.83E-178 |
| 101117934 | MYZAP | -2.093529312 | 3.07E-40 | 3.21E-40 |
| 101117952 | RNF207 | -2.346606396 | 3.03E-152 | 7.60E-152 |
| 101117955 | LOC101117955 | 3.535335534 | 0 | 0 |
| 101117966 | MMP17 | -6.818953707 | 1.18E-231 | 4.07E-231 |
| 101118054 | AIFM3 | -2.365304852 | 1.06E-57 | 1.36E-57 |
| 101118117 | GALR2 | 7.16068526 | 1.71E-208 | 5.38E-208 |
| 101118145 | MAP4K2 | -2.05871167 | 0 | 0 |
| 101118148 | ATP1A2 | 2.162106658 | 0 | 0 |
| 101118164 | LOC101118164 | 7.46649752 | 4.28E-237 | 1.51E-236 |
| 101118216 | LOC101118216 | -7.819204305 | 0 | 0 |
| 101118266 | FGL2 | -2.549310613 | 0 | 0 |
| 101118304 | OCLN | 2.181422037 | 0 | 0 |
| 101118332 | PLAT | -2.475921782 | 0 | 0 |
| 101118337 | TNC | -2.843438426 | 0 | 0 |
| 101118389 | DEGS2 | -2.728835366 | 1.81E-47 | 2.07E-47 |
| 101118410 | PRKCB | -2.341514288 | 0 | 0 |
| 101118419 | FRZB | -3.41962443 | 0 | 0 |
| 101118466 | ST8SIA6 | 6.663189719 | 0 | 0 |
| 101118487 | CTH | -2.517953758 | 0 | 0 |
| 101118551 | PLD3 | -2.210754592 | 0 | 0 |
| 101118555 | TAGLN | -2.263071104 | 0 | 0 |
| 101118587 | GK | 2.632438083 | 0 | 0 |
| 101118602 | LCN9 | 2.802333823 | 1.23E-271 | 4.89E-271 |
| 101118626 | ACOX1 | 2.741832717 | 0 | 0 |
| 101118665 | FGL1 | 2.533586353 | 1.61E-34 | 1.54E-34 |
| 101118698 | EDIL3 | -3.666352717 | 0 | 0 |
| 101118717 | LPGAT1 | -2.159738778 | 5.03E-252 | 1.89E-251 |
| 101118751 | LOC101118751 | -2.192719332 | 5.73E-87 | 9.63E-87 |
| 101118806 | EYA2 | -2.556339422 | 1.47E-229 | 5.01E-229 |
| 101118879 | TPD52L1 | 3.00152064 | 0 | 0 |
| 101118886 | FBF1 | 2.888697123 | 0 | 0 |
| 101118933 | FNDC4 | -2.256965892 | 2.68E-222 | 8.93E-222 |
| 101118949 | ABLIM3 | -3.412130219 | 0 | 0 |
| 101118975 | SMOX | -2.140899273 | 0 | 0 |
| 101118994 | SCAMP5 | -2.119408219 | 1.15E-129 | 2.56E-129 |
| 101119084 | HPDL | -2.702022893 | 2.11E-39 | 2.17E-39 |
| 101119095 | AFAP1L2 | -2.559959618 | 0 | 0 |
| 101119099 | GPR160 | 3.853460146 | 0 | 0 |
| 101119104 | RORC | -5.062868685 | 0 | 0 |
| 101119141 | RNF217 | 2.116374608 | 0 | 0 |
| 101119210 | ADAMTSL5 | -3.955457451 | 0 | 0 |
| 101119226 | LYNX1 | 3.08409684 | 9.01E-134 | 2.05E-133 |
| 101119252 | ABCB9 | -2.124470453 | 7.26E-155 | 1.85E-154 |
| 101119307 | CFI | -2.591931111 | 8.15E-199 | 2.47E-198 |
| 101119350 | SLC22A18 | -3.164586147 | 2.73E-217 | 8.94E-217 |
| 101119383 | KIF5A | 2.105669304 | 1.61E-70 | 2.33E-70 |
| 101119394 | GPAT3 | 2.040381106 | 1.24E-125 | 2.69E-125 |
| 101119430 | GPHA2 | -5.330103 | 9.52E-53 | 1.15E-52 |
| 101119448 | SERINC2 | -3.070833755 | 1.75E-202 | 5.37E-202 |
| 101119530 | LOC101119530 | 2.732272199 | 5.63E-245 | 2.05E-244 |
| 101119536 | SMARCA1 | -2.393089111 | 0 | 0 |
| 101119600 | TAGLN3 | -3.195407771 | 6.30E-267 | 2.45E-266 |
| 101119607 | NAT16 | 2.137161661 | 9.54E-44 | 1.05E-43 |
| 101119620 | LOC101119620 | 2.332809297 | 7.75E-181 | 2.20E-180 |
| 101119694 | RASEF | 2.252832533 | 1.11E-297 | 4.78E-297 |
| 101119695 | TMSB4X | 2.29763971 | 0 | 0 |
| 101119711 | CLEC12B | -3.666908061 | 0 | 0 |
| 101119731 | CD24 | 3.242101099 | 0 | 0 |
| 101119766 | TREX1 | -2.20890718 | 1.27E-30 | 1.15E-30 |
| 101119787 | RET | 2.419236544 | 0 | 0 |
| 101119804 | LOC101119804 | -2.932825573 | 7.56E-107 | 1.46E-106 |
| 101119894 | GPD1 | -13.19128933 | 0 | 0 |
| 101119942 | PACSIN1 | -2.821536239 | 0 | 0 |
| 101119956 | FAM3B | 2.378020437 | 0 | 0 |
| 101120016 | RAPSN | -3.230184009 | 1.42E-79 | 2.24E-79 |
| 101120029 | LOC101120029 | -2.104121336 | 1.86E-131 | 4.18E-131 |
| 101120035 | TMPRSS7 | -9.305858586 | 4.86E-227 | 1.64E-226 |
| 101120047 | RASGEF1A | -3.234776227 | 1.07E-260 | 4.10E-260 |
| 101120060 | LOC101120060 | -4.606291652 | 1.06E-77 | 1.63E-77 |
| 101120081 | SPOCK1 | 5.493329973 | 0 | 0 |
| 101120110 | FAM134B | 4.430018985 | 0 | 0 |
| 101120208 | S100A10 | -3.471718024 | 0 | 0 |
| 101120216 | TMPRSS2 | -3.210689186 | 0 | 0 |
| 101120351 | ELSPBP1 | 3.03016628 | 0 | 0 |
| 101120397 | GREB1 | -2.78348223 | 0 | 0 |
| 101120398 | CCDC85A | 2.636850313 | 6.74E-168 | 1.81E-167 |
| 101120418 | WISP1 | 3.089150259 | 0 | 0 |
| 101120487 | FKBP9 | -2.040635393 | 0 | 0 |
| 101120499 | SMPDL3A | -2.662918307 | 1.82E-196 | 5.48E-196 |
| 101120611 | POMGNT2 | -2.090714296 | 2.92E-59 | 3.79E-59 |
| 101120614 | MS4A8 | 5.667210917 | 2.02E-171 | 5.51E-171 |
| 101120630 | KALRN | 3.111167073 | 0 | 0 |
| 101120631 | RASSF4 | 2.636450658 | 0 | 0 |
| 101120726 | SLITRK4 | 4.704998599 | 0 | 0 |
| 101120766 | SERTAD4 | 2.050073749 | 0 | 0 |
| 101120781 | BDKRB2 | -2.284926973 | 9.34E-110 | 1.84E-109 |
| 101120830 | RGL3 | 4.554344913 | 0 | 0 |
| 101120879 | HSPA12A | -2.020909497 | 0 | 0 |
| 101120931 | CARD14 | 2.090230276 | 0 | 0 |
| 101120946 | IL10RA | -2.040850029 | 5.42E-105 | 1.03E-104 |
| 101120972 | GPAM | 2.624932044 | 0 | 0 |
| 101120988 | PTH2R | -11.12756715 | 0 | 0 |
| 101120990 | EDA | 2.296272513 | 6.98E-96 | 1.25E-95 |
| 101121016 | PKIB | -3.173033814 | 0 | 0 |
| 101121018 | MAP3K5 | 2.99582397 | 0 | 0 |
| 101121040 | EMB | -2.956187623 | 0 | 0 |
| 101121051 | ACTA2 | -2.091713502 | 0 | 0 |
| 101121082 | LOC101121082 | -2.704473146 | 3.96E-45 | 4.43E-45 |
| 101121083 | FAM19A5 | 7.345282822 | 7.98E-184 | 2.29E-183 |
| 101121099 | MDGA2 | -3.605807578 | 2.22E-93 | 3.90E-93 |
| 101121132 | SLC25A24 | -2.160261709 | 3.23E-226 | 1.09E-225 |
| 101121159 | LOC101121159 | -2.429274757 | 0 | 0 |
| 101121185 | ALOX15B | -5.697175351 | 0 | 0 |
| 101121444 | LAMA5 | -2.672935026 | 0 | 0 |
| 101121452 | LOC101121452 | 2.055897089 | 2.20E-48 | 2.56E-48 |
| 101121458 | SLC25A21 | -2.26555946 | 5.27E-56 | 6.62E-56 |
| 101121514 | LOC101121514 | -2.816741373 | 4.12E-104 | 7.79E-104 |
| 101121528 | DHX58 | 2.200826537 | 6.28E-147 | 1.53E-146 |
| 101121541 | CMIP | -2.190673664 | 1.18E-138 | 2.75E-138 |
| 101121563 | LOC101121563 | 3.181012233 | 0 | 0 |
| 101121663 | PRPS2 | -3.478268319 | 0 | 0 |
| 101121682 | DOK7 | -5.899477413 | 2.55E-85 | 4.22E-85 |
| 101121716 | SEMA3B | -2.242947393 | 0 | 0 |
| 101121758 | GLIPR1 | 3.814938305 | 0 | 0 |
| 101121820 | LOC101121820 | 2.17802911 | 0 | 0 |
| 101121831 | MECR | 2.883670536 | 0 | 0 |
| 101121871 | SKAP1 | 2.132707427 | 0 | 0 |
| 101121923 | LOC101121923 | -3.775160648 | 5.47E-160 | 1.43E-159 |
| 101121924 | FOXRED2 | -2.364067703 | 0 | 0 |
| 101121934 | RASGRF2 | -2.623861031 | 5.04E-206 | 1.57E-205 |
| 101122001 | NOSTRIN | -2.183124232 | 7.63E-74 | 1.14E-73 |
| 101122126 | PLCG2 | -2.740378741 | 0 | 0 |
| 101122152 | NANOS1 | -4.199332092 | 5.72E-84 | 9.40E-84 |
| 101122160 | ZNF365 | -2.663634273 | 1.36E-232 | 4.67E-232 |
| 101122194 | ACTN1 | -2.279743232 | 0 | 0 |
| 101122213 | MMP15 | -2.093463909 | 2.80E-62 | 3.76E-62 |
| 101122233 | SYT6 | -5.217332738 | 0 | 0 |
| 101122348 | GNPTAB | -2.942437043 | 0 | 0 |
| 101122401 | ADCY9 | -2.118009222 | 1.02E-93 | 1.79E-93 |
| 101122406 | MUC20 | -3.918871663 | 0 | 0 |
| 101122543 | SULT2B1 | -2.803746955 | 5.30E-20 | 3.93E-20 |
| 101122618 | NNAT | -2.221058601 | 6.51E-193 | 1.94E-192 |
| 101122707 | PLXDC1 | -2.048882066 | 2.65E-123 | 5.67E-123 |
| 101122711 | RGS1 | 2.443421888 | 6.73E-41 | 7.11E-41 |
| 101122803 | LOC101122803 | -5.188894675 | 0 | 0 |
| 101122804 | CFAP74 | -4.634774445 | 0 | 0 |
| 101122806 | SLC24A3 | -2.191565829 | 1.45E-134 | 3.31E-134 |
| 101122883 | GRAMD1A | -2.727521059 | 0 | 0 |
| 101122895 | DNASE1L3 | -5.513805279 | 0 | 0 |
| 101122905 | DUSP8 | 2.623599686 | 6.10E-58 | 7.83E-58 |
| 101122996 | PLEKHS1 | -7.884977843 | 7.84E-249 | 2.90E-248 |
| 101123024 | STAC3 | -2.764869388 | 8.59E-100 | 1.58E-99 |
| 101123054 | TNFSF13 | -2.510312438 | 3.52E-151 | 8.77E-151 |
| 101123070 | TMEM120B | -2.56114687 | 0 | 0 |
| 101123162 | DHCR24 | -2.191658164 | 8.60E-121 | 1.81E-120 |
| 101123200 | A2ML1 | 2.122049424 | 0 | 0 |
| 101123247 | PIK3R3 | -3.700313228 | 0 | 0 |
| 101123278 | ST6GALNAC6 | -2.837574974 | 0 | 0 |
| 101123308 | SLA | 2.840665558 | 9.81E-113 | 1.97E-112 |
| 101123341 | SH3BGR | -2.320251281 | 5.49E-158 | 1.42E-157 |
| 101123371 | FAM131B | 2.470547132 | 5.76E-305 | 2.51E-304 |
| 101123427 | IVL | 3.020477229 | 0 | 0 |
| 101123536 | LOC101123536 | -10.18790629 | 0 | 0 |
| 101123576 | ATP2C2 | -2.336297869 | 1.04E-50 | 1.24E-50 |
| 101123624 | ETV1 | 3.630761945 | 3.78E-274 | 1.51E-273 |
| 101123666 | FHDC1 | -2.12508966 | 8.28E-143 | 1.98E-142 |
| 105601850 | LOC105601850 | -3.721399569 | 2.64E-41 | 2.81E-41 |
| 105601863 | STON1 | -2.154442388 | 5.57E-129 | 1.23E-128 |
| 105602015 | LOC105602015 | 3.463500802 | 0 | 0 |
| 105602415 | C1QTNF5 | 3.377028797 | 0 | 0 |
| 105602646 | LOC105602646 | 3.11801696 | 0 | 0 |
| 105602976 | LOC105602976 | 2.656213591 | 4.56E-192 | 1.36E-191 |
| 105602979 | LOC105602979 | 3.981834806 | 0 | 0 |
| 105603000 | GGT1 | 3.632671502 | 0 | 0 |
| 105603154 | LOC105603154 | -2.837819939 | 1.32E-106 | 2.54E-106 |
| 105603239 | C18H14orf132 | -6.774695788 | 1.27E-26 | 1.07E-26 |
| 105603910 | MYLK4 | -2.817564567 | 0 | 0 |
| 105604082 | LOC105604082 | -4.253993517 | 2.33E-215 | 7.58E-215 |
| 105604152 | LOC105604152 | 3.078776224 | 7.16E-72 | 1.05E-71 |
| 105604619 | FLYWCH1 | -2.017523901 | 6.76E-217 | 2.21E-216 |
| 105604882 | LOC105604882 | -3.648868117 | 0 | 0 |
| 105605046 | ADIRF | -2.987159153 | 4.25E-229 | 1.45E-228 |
| 105605056 | LOC105605056 | 3.50086899 | 0 | 0 |
| 105605116 | LOC105605116 | 5.482279712 | 0 | 0 |
| 105605237 | LOC105605237 | -2.139191262 | 4.10E-18 | 2.91E-18 |
| 105605547 | PRRG3 | 3.123005996 | 0 | 0 |
| 105605561 | CDR1 | -2.748390025 | 3.41E-32 | 3.17E-32 |
| 105605702 | MAMLD1 | -5.83707622 | 0 | 0 |
| 105605950 | LOC105605950 | 5.16560243 | 0 | 0 |
| 105606696 | LOC105606696 | -6.471839876 | 0 | 0 |
| 105606698 | LOC105606698 | -5.088319236 | 0 | 0 |
| 105606699 | LOC105606699 | 3.015351747 | 0 | 0 |
| 105606703 | SPINK13 | 4.160240023 | 0 | 0 |
| 105608428 | LOC105608428 | 5.05423404 | 0 | 0 |
| 105608603 | LOC105608603 | -3.776357911 | 0 | 0 |
| 105608895 | LOC105608895 | 2.629137166 | 0 | 0 |
| 105609102 | LOC105609102 | -2.391897528 | 1.77E-46 | 2.01E-46 |
| 105609482 | NPTXR | -2.476545493 | 0 | 0 |
| 105609552 | ZSWIM1 | -2.232303829 | 5.30E-70 | 7.65E-70 |
| 105609865 | TUBB4A | 3.912450298 | 0 | 0 |
| 105610024 | TNNT2 | -2.194469784 | 5.82E-119 | 1.22E-118 |
| 105610939 | LOC105610939 | 6.05423404 | 1.69E-09 | 9.32E-10 |
| 105611318 | LOC105611318 | 4.551733699 | 6.54E-71 | 9.50E-71 |
| 105612422 | TMEM210 | -2.083854874 | 4.97E-08 | 2.59E-08 |
| 105613453 | LOC105613453 | 4.412045721 | 0 | 0 |
| 105613568 | PHLDA2 | -6.093924733 | 7.85E-129 | 1.73E-128 |
| 105613768 | LOC105613768 | 6.395053395 | 0 | 0 |
| 105615088 | LOC105615088 | 3.812858515 | 1.91E-62 | 2.57E-62 |
| 105615177 | KCNH2 | -3.303931573 | 2.16E-154 | 5.47E-154 |
| 105615865 | LOC105615865 | 3.505881159 | 0 | 0 |
| 105615953 | LOC105615953 | 3.724096705 | 0 | 0 |
| 105616123 | LOC105616123 | 3.43879041 | 0 | 0 |
| 105616429 | SMIM6 | -3.551538398 | 6.32E-84 | 1.04E-83 |
| 105616470 | CACNG5 | -4.040424303 | 5.00E-173 | 1.38E-172 |
| 106990163 | LOC106990163 | -3.94269176 | 0 | 0 |
| 106990376 | SPINK14 | -10.05473629 | 0 | 0 |
| 106991069 | LOC106991069 | 7.664155829 | 6.62E-24 | 5.29E-24 |
| 106991088 | LOC106991088 | -3.508313684 | 1.50E-55 | 1.87E-55 |
| 106991450 | LOC106991450 | 3.568578027 | 0 | 0 |
| 106991452 | LOC106991452 | -4.662979224 | 3.85E-211 | 1.22E-210 |
| 106991631 | LOC106991631 | 8.345109411 | 2.43E-68 | 3.46E-68 |
| 106991725 | LOC106991725 | 3.599675717 | 1.27E-51 | 1.52E-51 |
| 106991896 | LOC106991896 | 2.766472548 | 2.48E-30 | 2.23E-30 |
| BGI_novel_G000010 | BGI_novel_G000010 | -2.490033848 | 9.25E-165 | 2.46E-164 |
| BGI_novel_G000022 | BGI_novel_G000022 | 2.201746848 | 3.80E-163 | 1.01E-162 |
| BGI_novel_G000024 | BGI_novel_G000024 | 8.786982079 | 0 | 0 |
| BGI_novel_G000030 | BGI_novel_G000030 | -2.918469571 | 2.32E-131 | 5.22E-131 |
| BGI_novel_G000032 | BGI_novel_G000032 | 2.358376003 | 8.57E-29 | 7.52E-29 |
| BGI_novel_G000043 | BGI_novel_G000043 | 6.001334309 | 9.85E-92 | 1.71E-91 |
| BGI_novel_G000044 | BGI_novel_G000044 | 10.18106603 | 2.49E-95 | 4.42E-95 |
| BGI_novel_G000062 | BGI_novel_G000062 | -2.611225113 | 1.28E-152 | 3.23E-152 |
| BGI_novel_G000070 | BGI_novel_G000070 | -2.111745362 | 1.08E-44 | 1.20E-44 |
| BGI_novel_G000089 | BGI_novel_G000089 | -2.494025962 | 2.01E-148 | 4.94E-148 |
| BGI_novel_G000178 | BGI_novel_G000178 | 2.338189728 | 8.09E-99 | 1.48E-98 |
| BGI_novel_G000180 | BGI_novel_G000180 | 3.353268645 | 1.06E-43 | 1.16E-43 |
| BGI_novel_G000181 | BGI_novel_G000181 | -6.821131361 | 4.83E-53 | 5.88E-53 |
| BGI_novel_G000187 | BGI_novel_G000187 | 2.042691848 | 1.59E-138 | 3.71E-138 |
| BGI_novel_G000190 | BGI_novel_G000190 | 2.106322551 | 2.64E-17 | 1.84E-17 |
| BGI_novel_G000199 | BGI_novel_G000199 | -5.565319029 | 4.21E-123 | 8.99E-123 |
| BGI_novel_G000202 | BGI_novel_G000202 | 2.051609788 | 0 | 0 |
| BGI_novel_G000234 | BGI_novel_G000234 | 6.492063119 | 0 | 0 |
| BGI_novel_G000246 | BGI_novel_G000246 | 2.868698012 | 9.26E-59 | 1.20E-58 |
| BGI_novel_G000248 | BGI_novel_G000248 | 3.858097966 | 0 | 0 |
| BGI_novel_G000255 | BGI_novel_G000255 | 7.2402088 | 0 | 0 |
| BGI_novel_G000277 | BGI_novel_G000277 | 2.014177154 | 0 | 0 |
| BGI_novel_G000283 | BGI_novel_G000283 | -3.738075621 | 0 | 0 |
| BGI_novel_G000290 | BGI_novel_G000290 | 7.140698131 | 4.41E-162 | 1.16E-161 |
| BGI_novel_G000316 | BGI_novel_G000316 | -3.650335242 | 3.08E-214 | 9.91E-214 |
| BGI_novel_G000321 | BGI_novel_G000321 | -11.31283938 | 1.04E-168 | 2.79E-168 |
| BGI_novel_G000325 | BGI_novel_G000325 | 2.792924987 | 0 | 0 |
| BGI_novel_G000367 | BGI_novel_G000367 | -3.973127949 | 1.35E-183 | 3.87E-183 |
| BGI_novel_G000368 | BGI_novel_G000368 | -2.085114523 | 5.54E-122 | 1.17E-121 |
| BGI_novel_G000376 | BGI_novel_G000376 | 4.707139958 | 2.15E-192 | 6.38E-192 |
| BGI_novel_G000377 | BGI_novel_G000377 | 3.442152083 | 1.58E-198 | 4.77E-198 |
| BGI_novel_G000382 | BGI_novel_G000382 | 10.91906108 | 1.17E-140 | 2.77E-140 |
| BGI_novel_G000444 | BGI_novel_G000444 | -7.949994309 | 7.65E-208 | 2.40E-207 |
| BGI_novel_G000445 | BGI_novel_G000445 | -4.6375134 | 7.12E-298 | 3.07E-297 |
| BGI_novel_G000448 | BGI_novel_G000448 | 6.31534933 | 1.13E-79 | 1.78E-79 |
| BGI_novel_G000452 | BGI_novel_G000452 | -3.467135115 | 3.54E-155 | 9.04E-155 |
| BGI_novel_G000470 | BGI_novel_G000470 | 2.382198717 | 0 | 0 |
| BGI_novel_G000510 | BGI_novel_G000510 | 7.019379401 | 3.62E-62 | 4.86E-62 |
| BGI_novel_G000517 | BGI_novel_G000517 | -2.496860147 | 0 | 0 |
| BGI_novel_G000544 | BGI_novel_G000544 | 2.277788032 | 0 | 0 |
| BGI_novel_G000573 | BGI_novel_G000573 | 3.338389884 | 0 | 0 |
| BGI_novel_G000574 | BGI_novel_G000574 | 5.013553498 | 0 | 0 |
| BGI_novel_G000618 | BGI_novel_G000618 | 3.584984052 | 0 | 0 |
| BGI_novel_G000623 | BGI_novel_G000623 | 2.017397272 | 4.21E-29 | 3.71E-29 |
| BGI_novel_G000632 | BGI_novel_G000632 | 7.071774415 | 0 | 0 |
| BGI_novel_G000634 | BGI_novel_G000634 | -2.618971408 | 9.07E-40 | 9.42E-40 |
| BGI_novel_G000659 | BGI_novel_G000659 | 4.131158016 | 1.65E-111 | 3.29E-111 |
| BGI_novel_G000705 | BGI_novel_G000705 | 2.719762969 | 0 | 0 |
| BGI_novel_G000711 | BGI_novel_G000711 | 8.337719916 | 0 | 0 |
| BGI_novel_G000713 | BGI_novel_G000713 | -4.357299794 | 2.45E-154 | 6.21E-154 |
| BGI_novel_G000762 | BGI_novel_G000762 | 4.281812908 | 1.67E-181 | 4.75E-181 |
| BGI_novel_G000774 | BGI_novel_G000774 | -2.034850479 | 1.60E-243 | 5.81E-243 |
| BGI_novel_G000800 | BGI_novel_G000800 | 3.946887774 | 6.33E-243 | 2.29E-242 |
| BGI_novel_G000801 | BGI_novel_G000801 | 13.05077233 | 0 | 0 |
| BGI_novel_G000802 | BGI_novel_G000802 | 3.11546186 | 0 | 0 |
| BGI_novel_G000829 | BGI_novel_G000829 | 3.089633365 | 5.56E-162 | 1.46E-161 |
| BGI_novel_G000830 | BGI_novel_G000830 | 3.944363751 | 0 | 0 |
| BGI_novel_G000831 | BGI_novel_G000831 | 4.329996315 | 0 | 0 |
| BGI_novel_G000832 | BGI_novel_G000832 | 4.800969218 | 0 | 0 |
| BGI_novel_G000845 | BGI_novel_G000845 | 2.143195657 | 3.60E-96 | 6.44E-96 |
| BGI_novel_G000848 | BGI_novel_G000848 | 4.752800371 | 7.76E-241 | 2.77E-240 |
| BGI_novel_G000867 | BGI_novel_G000867 | -7.5363016 | 0 | 0 |
| BGI_novel_G000868 | BGI_novel_G000868 | -8.21244125 | 7.55E-32 | 6.97E-32 |
| BGI_novel_G000870 | BGI_novel_G000870 | 8.878871271 | 0 | 0 |
| BGI_novel_G000913 | BGI_novel_G000913 | 5.096118894 | 0 | 0 |
| BGI_novel_G000915 | BGI_novel_G000915 | -2.443831991 | 1.27E-191 | 3.77E-191 |
| BGI_novel_G000920 | BGI_novel_G000920 | 2.623643944 | 0 | 0 |
| BGI_novel_G000936 | BGI_novel_G000936 | 3.777932664 | 0 | 0 |
| BGI_novel_G000944 | BGI_novel_G000944 | 2.590560434 | 6.95E-111 | 1.38E-110 |
| BGI_novel_G000999 | BGI_novel_G000999 | 2.220190516 | 3.14E-157 | 8.07E-157 |
| BGI_novel_G001017 | BGI_novel_G001017 | 2.787787688 | 7.66E-167 | 2.05E-166 |
| BGI_novel_G001020 | BGI_novel_G001020 | 2.380043312 | 2.18E-106 | 4.18E-106 |
| BGI_novel_G001024 | BGI_novel_G001024 | -5.247547922 | 4.20E-72 | 6.17E-72 |
| BGI_novel_G001025 | BGI_novel_G001025 | -3.917020167 | 0 | 0 |
| BGI_novel_G001069 | BGI_novel_G001069 | -3.256626921 | 5.87E-244 | 2.13E-243 |
| BGI_novel_G001070 | BGI_novel_G001070 | -3.743119965 | 5.67E-95 | 1.01E-94 |
| BGI_novel_G001082 | BGI_novel_G001082 | 4.249807896 | 3.89E-112 | 7.78E-112 |
| BGI_novel_G001086 | BGI_novel_G001086 | 2.428032864 | 2.96E-155 | 7.56E-155 |
| BGI_novel_G001088 | BGI_novel_G001088 | -2.770818473 | 1.45E-187 | 4.22E-187 |
| BGI_novel_G001103 | BGI_novel_G001103 | 6.15478661 | 0 | 0 |
| BGI_novel_G001112 | BGI_novel_G001112 | -3.080381545 | 3.61E-107 | 6.99E-107 |
| BGI_novel_G001118 | BGI_novel_G001118 | 4.60149998 | 1.97E-167 | 5.28E-167 |
| BGI_novel_G001119 | BGI_novel_G001119 | 7.91231949 | 0 | 0 |
| BGI_novel_G001134 | BGI_novel_G001134 | 2.515554062 | 0 | 0 |
| BGI_novel_G001135 | BGI_novel_G001135 | 3.049135126 | 0 | 0 |
| BGI_novel_G001149 | BGI_novel_G001149 | -3.070411871 | 0 | 0 |
| BGI_novel_G001150 | BGI_novel_G001150 | -4.106570593 | 1.72E-174 | 4.76E-174 |
| BGI_novel_G001151 | BGI_novel_G001151 | -2.358300464 | 9.20E-109 | 1.80E-108 |
| BGI_novel_G001152 | BGI_novel_G001152 | -3.836255417 | 2.43E-151 | 6.07E-151 |
| BGI_novel_G001159 | BGI_novel_G001159 | 2.31997846 | 2.53E-48 | 2.94E-48 |
| BGI_novel_G001162 | BGI_novel_G001162 | 3.010644037 | 3.50E-214 | 1.13E-213 |
| BGI_novel_G001203 | BGI_novel_G001203 | -4.392539118 | 0 | 0 |
| BGI_novel_G001206 | BGI_novel_G001206 | -4.718062365 | 2.53E-82 | 4.10E-82 |
| BGI_novel_G001207 | BGI_novel_G001207 | -2.663587381 | 0 | 0 |
| BGI_novel_G001209 | BGI_novel_G001209 | 4.604539594 | 0 | 0 |
| BGI_novel_G001237 | BGI_novel_G001237 | 5.298967629 | 3.80E-67 | 5.35E-67 |
| BGI_novel_G001238 | BGI_novel_G001238 | 2.432520395 | 1.98E-181 | 5.63E-181 |
| BGI_novel_G001239 | BGI_novel_G001239 | 5.952777993 | 0 | 0 |
| BGI_novel_G001241 | BGI_novel_G001241 | 6.091385007 | 0 | 0 |
| BGI_novel_G001244 | BGI_novel_G001244 | 3.916853865 | 0 | 0 |
| BGI_novel_G001247 | BGI_novel_G001247 | 4.981778538 | 0 | 0 |
| BGI_novel_G001248 | BGI_novel_G001248 | 6.014348816 | 3.61E-278 | 1.47E-277 |
| BGI_novel_G001249 | BGI_novel_G001249 | 3.57711169 | 1.42E-300 | 6.14E-300 |
| BGI_novel_G001269 | BGI_novel_G001269 | 2.671762921 | 2.27E-60 | 2.99E-60 |
| BGI_novel_G001270 | BGI_novel_G001270 | 4.710737013 | 0 | 0 |
| BGI_novel_G001273 | BGI_novel_G001273 | 2.206250189 | 2.45E-131 | 5.51E-131 |
| BGI_novel_G001274 | BGI_novel_G001274 | 2.179985461 | 1.43E-129 | 3.18E-129 |
| BGI_novel_G001310 | BGI_novel_G001310 | -2.073435124 | 2.52E-54 | 3.12E-54 |
| BGI_novel_G001315 | BGI_novel_G001315 | 7.364591712 | 0 | 0 |
| BGI_novel_G001317 | BGI_novel_G001317 | 7.669191281 | 0 | 0 |
| BGI_novel_G001326 | BGI_novel_G001326 | 13.29282985 | 0 | 0 |
| BGI_novel_G001327 | BGI_novel_G001327 | 3.175367276 | 0 | 0 |
| BGI_novel_G001341 | BGI_novel_G001341 | -2.20630702 | 1.34E-54 | 1.66E-54 |
| BGI_novel_G001348 | BGI_novel_G001348 | 3.686851126 | 1.64E-188 | 4.81E-188 |
| BGI_novel_G001357 | BGI_novel_G001357 | 3.731341254 | 5.95E-61 | 7.89E-61 |
| BGI_novel_G001365 | BGI_novel_G001365 | -2.061169454 | 4.74E-77 | 7.28E-77 |
| BGI_novel_G001369 | BGI_novel_G001369 | -2.551869589 | 2.24E-67 | 3.16E-67 |
| BGI_novel_G001375 | BGI_novel_G001375 | 2.368804009 | 0 | 0 |
| BGI_novel_G001388 | BGI_novel_G001388 | -4.084722313 | 1.17E-154 | 2.98E-154 |
| BGI_novel_G001424 | BGI_novel_G001424 | 2.688216881 | 3.85E-149 | 9.48E-149 |
| BGI_novel_G001443 | BGI_novel_G001443 | 4.172247744 | 0 | 0 |
| BGI_novel_G001455 | BGI_novel_G001455 | -4.560966433 | 6.93E-237 | 2.43E-236 |
| BGI_novel_G001515 | BGI_novel_G001515 | -3.259471371 | 3.82E-79 | 6.00E-79 |
| BGI_novel_G001528 | BGI_novel_G001528 | 5.19041047 | 3.46E-263 | 1.34E-262 |
| BGI_novel_G001530 | BGI_novel_G001530 | 4.633948463 | 1.05E-154 | 2.66E-154 |
| BGI_novel_G001560 | BGI_novel_G001560 | 3.82925836 | 0 | 0 |
| BGI_novel_G001561 | BGI_novel_G001561 | 6.517088527 | 0 | 0 |
| BGI_novel_G001562 | BGI_novel_G001562 | 7.078768451 | 0 | 0 |
| BGI_novel_G001586 | BGI_novel_G001586 | -3.05690163 | 1.12E-96 | 2.01E-96 |
| BGI_novel_G001610 | BGI_novel_G001610 | -3.088099254 | 6.27E-229 | 2.13E-228 |
| BGI_novel_G001620 | BGI_novel_G001620 | -2.074480581 | 1.38E-77 | 2.13E-77 |
| BGI_novel_G001640 | BGI_novel_G001640 | -3.847882296 | 0 | 0 |
| BGI_novel_G001641 | BGI_novel_G001641 | -5.944468447 | 1.09E-214 | 3.54E-214 |
| BGI_novel_G001643 | BGI_novel_G001643 | 4.546224131 | 0 | 0 |
| BGI_novel_G001644 | BGI_novel_G001644 | 4.73397371 | 0 | 0 |
| BGI_novel_G001647 | BGI_novel_G001647 | 3.580008153 | 2.08E-189 | 6.09E-189 |
| BGI_novel_G001684 | BGI_novel_G001684 | -3.419784732 | 7.67E-305 | 3.35E-304 |
| BGI_novel_G001685 | BGI_novel_G001685 | -7.020391806 | 1.39E-283 | 5.72E-283 |
| BGI_novel_G001686 | BGI_novel_G001686 | -6.356351816 | 0 | 0 |
| BGI_novel_G001694 | BGI_novel_G001694 | 3.591810379 | 1.09E-104 | 2.07E-104 |
| BGI_novel_G001701 | BGI_novel_G001701 | -3.102004647 | 4.44E-211 | 1.41E-210 |
| BGI_novel_G001751 | BGI_novel_G001751 | -3.442104421 | 6.23E-234 | 2.15E-233 |
| BGI_novel_G001760 | BGI_novel_G001760 | -3.233053959 | 7.52E-84 | 1.23E-83 |
| BGI_novel_G001763 | BGI_novel_G001763 | 6.667189509 | 0 | 0 |
| BGI_novel_G001765 | BGI_novel_G001765 | 10.68793876 | 3.40E-247 | 1.25E-246 |
| BGI_novel_G001766 | BGI_novel_G001766 | 6.988836128 | 0 | 0 |
| BGI_novel_G001770 | BGI_novel_G001770 | 3.876555647 | 7.88E-82 | 1.27E-81 |
| BGI_novel_G001771 | BGI_novel_G001771 | 4.264671495 | 5.17E-213 | 1.65E-212 |
| BGI_novel_G001801 | BGI_novel_G001801 | 8.921803089 | 0 | 0 |
| BGI_novel_G001802 | BGI_novel_G001802 | 6.858373734 | 0 | 0 |
| BGI_novel_G001817 | BGI_novel_G001817 | -2.974313838 | 0 | 0 |
| BGI_novel_G001877 | BGI_novel_G001877 | -2.58057701 | 2.03E-116 | 4.17E-116 |
| BGI_novel_G001891 | BGI_novel_G001891 | 2.520509767 | 3.16E-33 | 2.98E-33 |
| BGI_novel_G001920 | BGI_novel_G001920 | -3.348676503 | 1.41E-21 | 1.08E-21 |
| BGI_novel_G001948 | BGI_novel_G001948 | 6.409099821 | 0 | 0 |
| BGI_novel_G001957 | BGI_novel_G001957 | -2.134326205 | 9.78E-21 | 7.36E-21 |
| BGI_novel_G001976 | BGI_novel_G001976 | 4.488360422 | 3.44E-37 | 3.45E-37 |
| BGI_novel_G002004 | BGI_novel_G002004 | 5.235804345 | 1.63E-28 | 1.42E-28 |
| BGI_novel_G002039 | BGI_novel_G002039 | 2.141972284 | 3.64E-268 | 1.42E-267 |
| BGI_novel_G002040 | BGI_novel_G002040 | -8.295041659 | 2.37E-33 | 2.24E-33 |
| BGI_novel_G002056 | BGI_novel_G002056 | 3.350636212 | 2.65E-127 | 5.82E-127 |
| BGI_novel_G002085 | BGI_novel_G002085 | 8.085608477 | 2.56E-30 | 2.30E-30 |
| BGI_novel_G002086 | BGI_novel_G002086 | 8.085608477 | 2.56E-30 | 2.30E-30 |
