# Supplementary material for: Study on the region-specific expression of epididymis mRNA in the rams
Source: PLoS One. 2021 Jan 25;16(1):e0245933. doi: 10.1371/journal.pone.0245933 (PMC7833257; doi:10.1371/journal.pone.0245933)
Supplement: S8 Table — (DOCX) [file pone.0245933.s012.docx]

# S8 Table. The DEGs list between caput and cauda

| **Gene ID** | **Other Gene ID** | **log2(Caput/Cauda)** | **Pvalue(Caput-vs-Cauda)** | **Qvalue(Caput-vs-Cauda)** |
| --- | --- | --- | --- | --- |
| 443033 | SFTPA1 | -4.59166 | 0 | 0 |
| 443038 | CAPN3 | -3.50079 | 0 | 0 |
| 443049 | PDXK | 2.960256 | 0 | 0 |
| 443066 | ESR2 | -6.93868 | 0 | 0 |
| 443078 | NOS2 | -3.49131 | 1.18E-111 | 9.46E-112 |
| 443083 | IRF6 | -3.27134 | 0 | 0 |
| 443109 | TFAP2A | -2.30227 | 0 | 0 |
| 443115 | MMP2 | 4.530127 | 0 | 0 |
| 443174 | BMP6 | 3.653152 | 1.97E-217 | 2.52E-217 |
| 443184 | AQP9 | -3.21351 | 0 | 0 |
| 443199 | TJP3 | -2.2248 | 0 | 0 |
| 443203 | MS4A2 | 10.60098 | 0 | 0 |
| 443221 | RNASE10 | 13.50663 | 0 | 0 |
| 443232 | CD40 | 3.09921 | 1.36E-37 | 6.13E-38 |
| 443264 | LEPR | -4.1561 | 2.60E-171 | 2.77E-171 |
| 443279 | CHI3L1 | 5.114069 | 0 | 0 |
| 443282 | GGTA2P | 3.97007 | 0 | 0 |
| 443305 | SELP | -5.55889 | 0 | 0 |
| 443325 | IGF2 | -2.08166 | 0 | 0 |
| 443326 | ALOX5AP | -4.44501 | 0 | 0 |
| 443331 | TIMP1 | 4.247136 | 0 | 0 |
| 443360 | HOXC9 | -4.57091 | 2.19E-33 | 9.41E-34 |
| 443388 | SERPINA1 | -8.31239 | 0 | 0 |
| 443390 | OXT | 3.755743 | 8.38E-79 | 5.48E-79 |
| 443411 | LOC443411 | 4.719229 | 0 | 0 |
| 443436 | CD247 | 2.225769 | 0 | 0 |
| 443450 | BCAT2 | -3.4297 | 0 | 0 |
| 443454 | BMPR1B | -5.64267 | 0 | 0 |
| 443460 | PTGS2 | -2.31066 | 8.89E-174 | 9.57E-174 |
| 443488 | SCNN1A | -4.92051 | 0 | 0 |
| 443505 | SLC34A1 | 12.87578 | 0 | 0 |
| 443514 | SLC1A1 | -3.09184 | 1.68E-85 | 1.15E-85 |
| 443517 | CNN1 | -2.28553 | 0 | 0 |
| 443524 | INHBA | 5.634779 | 0 | 0 |
| 443532 | CXCR4 | 2.347319 | 2.18E-243 | 3.02E-243 |
| 492300 | SLC5A1 | -10.2337 | 0 | 0 |
| 493773 | NPPC | -5.81018 | 3.47E-67 | 2.08E-67 |
| 494440 | SLC4A4 | 3.942801 | 0 | 0 |
| 554254 | TLR7 | -3.54914 | 0 | 0 |
| 554323 | TGFB3 | 2.54619 | 0 | 0 |
| 554335 | ACE | 3.548036 | 0 | 0 |
| 641305 | PI3 | -5.10516 | 2.34E-146 | 2.24E-146 |
| 654334 | SLCO2A1 | 3.413661 | 0 | 0 |
| 678680 | LGMN | 4.036276 | 0 | 0 |
| 780451 | CES5A | -8.46114 | 0 | 0 |
| 780456 | ACSS1 | -2.27625 | 0 | 0 |
| 780468 | ARG2 | -2.57057 | 5.05E-105 | 3.89E-105 |
| 780469 | CTNNBL1 | 2.156264 | 0 | 0 |
| 780475 | CLDN2 | 12.9448 | 0 | 0 |
| 780506 | NAMPT | -2.37558 | 0 | 0 |
| 780509 | MB | -5.37915 | 8.07E-291 | 1.28E-290 |
| 780521 | LEF1 | 5.974359 | 0 | 0 |
| 1E+08 | NOX4 | 7.797149 | 0 | 0 |
| 1E+08 | CYB561 | 2.305733 | 0 | 0 |
| 1E+08 | CTTNBP2 | 2.507394 | 1.45E-287 | 2.28E-287 |
| 1E+08 | ST3GAL4 | -5.30605 | 0 | 0 |
| 1E+08 | PLIN5 | -2.37361 | 2.12E-175 | 2.30E-175 |
| 1E+08 | CITED2 | -2.06048 | 0 | 0 |
| 1E+08 | FABP4 | -3.20112 | 8.45E-47 | 4.23E-47 |
| 1E+08 | CCND1 | -2.33285 | 0 | 0 |
| 1E+08 | FCGRT | 2.700707 | 0 | 0 |
| 1E+08 | HEXA | 3.580653 | 0 | 0 |
| 1E+08 | MC4R | -2.75135 | 0 | 0 |
| 1E+08 | SLC25A33 | -3.19896 | 1.03E-262 | 1.52E-262 |
| 1E+08 | LIPE | -2.49212 | 8.49E-120 | 7.11E-120 |
| 1E+08 | CRY2 | -2.36554 | 0 | 0 |
| 1E+08 | ADRA1D | -3.11466 | 4.22E-105 | 3.25E-105 |
| 1E+08 | DPP4 | -6.38986 | 0 | 0 |
| 1E+08 | RGN | -3.96567 | 0 | 0 |
| 1E+08 | ITM2B | 2.215414 | 0 | 0 |
| 1E+08 | HPCAL1 | 3.754022 | 0 | 0 |
| 1E+08 | MMP9 | 5.232667 | 0 | 0 |
| 1E+08 | DBP | -2.01636 | 0 | 0 |
| 1E+08 | HLF | -2.73957 | 1.48E-252 | 2.10E-252 |
| 1E+08 | FCGR1A | -6.75734 | 0 | 0 |
| 1E+08 | FOXP3 | -2.33371 | 0 | 0 |
| 1E+08 | HEY2 | 5.76003 | 0 | 0 |
| 1E+08 | MLPH | -3.06935 | 6.33E-95 | 4.59E-95 |
| 1E+08 | MIP | -2.02906 | 2.28E-41 | 1.07E-41 |
| 1E+08 | LYN | -2.09862 | 0 | 0 |
| 1E+08 | ARL4C | 2.033462 | 1.65E-59 | 9.31E-60 |
| 1E+08 | ARL8A | -2.20353 | 8.04E-236 | 1.09E-235 |
| 1E+08 | SDR16C5 | 3.634586 | 0 | 0 |
| 1E+08 | SLC1A5 | 4.105092 | 0 | 0 |
| 1E+08 | IZUMO1 | 8.574087 | 0 | 0 |
| 1E+08 | KRT17 | 4.517662 | 4.45E-126 | 3.86E-126 |
| 1E+08 | CAT | 4.401729 | 0 | 0 |
| 1E+08 | SREBF1 | -2.63128 | 0 | 0 |
| 1E+08 | DUOXA2 | -5.39448 | 7.70E-119 | 6.41E-119 |
| 1E+08 | PAX2 | -5.28552 | 0 | 0 |
| 1E+08 | SLC26A3 | -3.56653 | 0 | 0 |
| 1E+08 | CRISP3 | 4.397605 | 0 | 0 |
| 1E+08 | DUOX2 | -6.39678 | 0 | 0 |
| 1.01E+08 | TTPA | -11.7051 | 3.50E-206 | 4.31E-206 |
| 1.01E+08 | UGT1A1 | -2.23465 | 2.04E-60 | 1.16E-60 |
| 1.01E+08 | CYP2E1 | 12.00542 | 1.61E-246 | 2.25E-246 |
| 1.01E+08 | KCNMB4 | -2.36326 | 4.21E-130 | 3.74E-130 |
| 1.01E+08 | ACP5 | -3.76677 | 0 | 0 |
| 1.01E+08 | SRD5A2 | 6.46723 | 0 | 0 |
| 1.01E+08 | GADD45G | -2.03981 | 2.40E-56 | 1.32E-56 |
| 1.01E+08 | GPX5 | 8.962367 | 0 | 0 |
| 1.01E+08 | HIVEP3 | 3.585603 | 0 | 0 |
| 1.01E+08 | UPF3B | -2.40518 | 0 | 0 |
| 1.01E+08 | HMCN2 | -4.16547 | 0 | 0 |
| 1.01E+08 | C7H14orf93 | -2.42258 | 0 | 0 |
| 1.01E+08 | VOPP1 | -2.60606 | 0 | 0 |
| 1.01E+08 | CAPG | -3.23878 | 0 | 0 |
| 1.01E+08 | SH2D6 | -4.99083 | 0 | 0 |
| 1.01E+08 | TCF7 | 2.616031 | 0 | 0 |
| 1.01E+08 | NIPAL1 | -3.05629 | 7.62E-105 | 5.86E-105 |
| 1.01E+08 | FMN1 | -2.304 | 5.49E-201 | 6.62E-201 |
| 1.01E+08 | SELL | -4.33553 | 0 | 0 |
| 1.01E+08 | NCS1 | -2.51539 | 0 | 0 |
| 1.01E+08 | MFSD6L | 3.739448 | 0 | 0 |
| 1.01E+08 | LOC101102178 | 2.319542 | 0 | 0 |
| 1.01E+08 | VAMP5 | -2.13013 | 0 | 0 |
| 1.01E+08 | LOC101102231 | -2.42321 | 6.97E-42 | 3.30E-42 |
| 1.01E+08 | IL27RA | 2.098647 | 5.04E-166 | 5.25E-166 |
| 1.01E+08 | MTHFD1L | -2.32949 | 0 | 0 |
| 1.01E+08 | TMEM71 | -3.68545 | 0 | 0 |
| 1.01E+08 | LOC101102327 | -3.79873 | 0 | 0 |
| 1.01E+08 | EVA1C | -2.80103 | 3.15E-224 | 4.13E-224 |
| 1.01E+08 | PMAIP1 | 2.62983 | 6.43E-166 | 6.69E-166 |
| 1.01E+08 | PLCL1 | 2.833382 | 1.21E-252 | 1.71E-252 |
| 1.01E+08 | TRIB1 | -2.25976 | 9.35E-136 | 8.54E-136 |
| 1.01E+08 | DUSP22 | -2.64844 | 1.49E-79 | 9.77E-80 |
| 1.01E+08 | GCAT | -2.0863 | 0 | 0 |
| 1.01E+08 | KCTD12 | 4.483812 | 0 | 0 |
| 1.01E+08 | LOC101102548 | -6.57186 | 0 | 0 |
| 1.01E+08 | TPX2 | -2.87547 | 0 | 0 |
| 1.01E+08 | GC | -9.02248 | 0 | 0 |
| 1.01E+08 | SCG5 | -5.49893 | 0 | 0 |
| 1.01E+08 | FADS6 | -2.49271 | 1.08E-56 | 5.94E-57 |
| 1.01E+08 | PASK | 2.088357 | 1.18E-257 | 1.70E-257 |
| 1.01E+08 | QPRT | 3.607524 | 3.15E-235 | 4.27E-235 |
| 1.01E+08 | TMEM2 | 2.070844 | 0 | 0 |
| 1.01E+08 | MID1IP1 | -2.8221 | 0 | 0 |
| 1.01E+08 | NHSL2 | -3.55886 | 0 | 0 |
| 1.01E+08 | CLIP4 | -2.69716 | 0 | 0 |
| 1.01E+08 | ZNF648 | -6.72867 | 2.04E-194 | 2.41E-194 |
| 1.01E+08 | HAPLN3 | -2.94999 | 6.15E-203 | 7.49E-203 |
| 1.01E+08 | LYRM9 | -2.1467 | 1.37E-264 | 2.02E-264 |
| 1.01E+08 | PLSCR1 | -3.49078 | 0 | 0 |
| 1.01E+08 | PPP1R3B | -2.1804 | 0 | 0 |
| 1.01E+08 | MLLT3 | 2.549281 | 1.31E-162 | 1.34E-162 |
| 1.01E+08 | GALNT3 | -2.70672 | 0 | 0 |
| 1.01E+08 | RTN1 | 3.735343 | 2.31E-298 | 3.72E-298 |
| 1.01E+08 | CRISP1 | -4.80298 | 0 | 0 |
| 1.01E+08 | LCNL1 | 7.317004 | 0 | 0 |
| 1.01E+08 | KCNK5 | 4.209384 | 0 | 0 |
| 1.01E+08 | GRHL1 | 2.341849 | 0 | 0 |
| 1.01E+08 | NLK | -2.02454 | 0 | 0 |
| 1.01E+08 | PPP1R1B | -3.17616 | 0 | 0 |
| 1.01E+08 | LOC101103112 | -6.40132 | 4.74E-138 | 4.36E-138 |
| 1.01E+08 | BMX | -4.38332 | 0 | 0 |
| 1.01E+08 | ZNF275 | -3.89272 | 0 | 0 |
| 1.01E+08 | TEKT1 | 7.871966 | 4.26E-303 | 6.99E-303 |
| 1.01E+08 | LOC101103174 | -2.74688 | 0 | 0 |
| 1.01E+08 | TDRD9 | -2.28105 | 1.12E-96 | 8.23E-97 |
| 1.01E+08 | C1H2orf54 | 7.126303 | 1.31E-192 | 1.53E-192 |
| 1.01E+08 | C2H9orf91 | -2.12502 | 2.26E-133 | 2.04E-133 |
| 1.01E+08 | PREX2 | 3.265193 | 0 | 0 |
| 1.01E+08 | SNX31 | 7.658881 | 2.09E-134 | 1.89E-134 |
| 1.01E+08 | RGSL1 | 10.90093 | 0 | 0 |
| 1.01E+08 | RLBP1 | -9.28959 | 0 | 0 |
| 1.01E+08 | DAAM2 | 3.660176 | 0 | 0 |
| 1.01E+08 | CTPS1 | -2.68015 | 0 | 0 |
| 1.01E+08 | TIAM1 | -2.34605 | 2.01E-161 | 2.05E-161 |
| 1.01E+08 | STXBP1 | 3.483043 | 0 | 0 |
| 1.01E+08 | ATP6V1C2 | -3.4117 | 0 | 0 |
| 1.01E+08 | PLSCR4 | -3.48429 | 0 | 0 |
| 1.01E+08 | TFR2 | -4.89775 | 0 | 0 |
| 1.01E+08 | IL6R | -2.80246 | 0 | 0 |
| 1.01E+08 | S1PR3 | 4.218834 | 6.94E-120 | 5.81E-120 |
| 1.01E+08 | UBXN10 | 3.351256 | 0 | 0 |
| 1.01E+08 | TTC27 | 2.070793 | 0 | 0 |
| 1.01E+08 | LOC101103396 | -3.37357 | 0 | 0 |
| 1.01E+08 | LOC101103398 | -3.7402 | 0 | 0 |
| 1.01E+08 | SCEL | -5.19377 | 0 | 0 |
| 1.01E+08 | TFAP2B | -5.99788 | 0 | 0 |
| 1.01E+08 | SNX24 | 3.836116 | 0 | 0 |
| 1.01E+08 | ROS1 | 2.7486 | 0 | 0 |
| 1.01E+08 | DUSP4 | -2.5603 | 2.36E-280 | 3.64E-280 |
| 1.01E+08 | KDM6A | -2.25034 | 0 | 0 |
| 1.01E+08 | LOC101103584 | 10.01796 | 0 | 0 |
| 1.01E+08 | SHE | -2.69051 | 0 | 0 |
| 1.01E+08 | ACE2 | -3.04057 | 1.91E-137 | 1.76E-137 |
| 1.01E+08 | LOC101103631 | -3.86506 | 0 | 0 |
| 1.01E+08 | PLA2G2C | 2.916843 | 0 | 0 |
| 1.01E+08 | WDYHV1 | -2.64842 | 0 | 0 |
| 1.01E+08 | SLAIN1 | -2.63293 | 0 | 0 |
| 1.01E+08 | LOC101103666 | -11.5892 | 0 | 0 |
| 1.01E+08 | INPP5J | 3.068848 | 0 | 0 |
| 1.01E+08 | INF2 | 2.687801 | 0 | 0 |
| 1.01E+08 | EPHA6 | 4.118784 | 3.03E-129 | 2.69E-129 |
| 1.01E+08 | TSPEAR | -6.31247 | 0 | 0 |
| 1.01E+08 | TCAP | -6.71297 | 0 | 0 |
| 1.01E+08 | ARHGEF3 | 2.040517 | 1.75E-275 | 2.65E-275 |
| 1.01E+08 | GAP43 | -8.7324 | 0 | 0 |
| 1.01E+08 | SPTSSB | -10.6487 | 0 | 0 |
| 1.01E+08 | IKZF2 | -2.12623 | 0 | 0 |
| 1.01E+08 | PNMT | -5.4293 | 0 | 0 |
| 1.01E+08 | LCLAT1 | -3.97725 | 0 | 0 |
| 1.01E+08 | PRRT4 | -3.47815 | 3.02E-183 | 3.39E-183 |
| 1.01E+08 | SMIM3 | -2.70756 | 0 | 0 |
| 1.01E+08 | PMEPA1 | 2.303345 | 0 | 0 |
| 1.01E+08 | BSPH1 | 3.226638 | 0 | 0 |
| 1.01E+08 | CWH43 | -5.42943 | 0 | 0 |
| 1.01E+08 | FUT4 | 2.548036 | 9.19E-77 | 5.91E-77 |
| 1.01E+08 | LY6G5C | 14.11309 | 0 | 0 |
| 1.01E+08 | PTGES | -5.28105 | 0 | 0 |
| 1.01E+08 | IMPDH1 | -3.80022 | 0 | 0 |
| 1.01E+08 | SHISA6 | -4.89583 | 0 | 0 |
| 1.01E+08 | GAL3ST3 | 3.468834 | 0 | 0 |
| 1.01E+08 | SLC9A9 | -2.87847 | 0 | 0 |
| 1.01E+08 | LOC101104222 | -6.76648 | 0 | 0 |
| 1.01E+08 | NCOA2 | -2.43467 | 0 | 0 |
| 1.01E+08 | RTN4RL1 | -2.77768 | 0 | 0 |
| 1.01E+08 | CST11 | 14.22916 | 0 | 0 |
| 1.01E+08 | FBXO24 | -2.08838 | 1.45E-112 | 1.17E-112 |
| 1.01E+08 | SEC14L5 | -7.23905 | 6.47E-135 | 5.89E-135 |
| 1.01E+08 | TMEM213 | 4.512218 | 0 | 0 |
| 1.01E+08 | HSPB6 | -2.55582 | 5.25E-145 | 4.97E-145 |
| 1.01E+08 | CA5B | -4.18234 | 0 | 0 |
| 1.01E+08 | RAP1GAP | 2.565016 | 5.11E-157 | 5.11E-157 |
| 1.01E+08 | KIAA0513 | 2.335373 | 1.31E-206 | 1.62E-206 |
| 1.01E+08 | LOC101104423 | -2.65547 | 5.45E-86 | 3.74E-86 |
| 1.01E+08 | TNNT3 | -5.10249 | 0 | 0 |
| 1.01E+08 | SLCO4C1 | 5.67508 | 0 | 0 |
| 1.01E+08 | GCNT4 | 11.3389 | 0 | 0 |
| 1.01E+08 | NPL | -2.20392 | 0 | 0 |
| 1.01E+08 | LOC101104518 | 7.565019 | 9.21E-86 | 6.31E-86 |
| 1.01E+08 | TCTA | -2.38521 | 0 | 0 |
| 1.01E+08 | SLC6A20 | -2.30209 | 8.16E-191 | 9.46E-191 |
| 1.01E+08 | S100A5 | -3.50508 | 6.81E-34 | 2.94E-34 |
| 1.01E+08 | AGFG2 | -2.29014 | 0 | 0 |
| 1.01E+08 | C24H16orf89 | -9.37845 | 0 | 0 |
| 1.01E+08 | LOC101104568 | -4.01591 | 0 | 0 |
| 1.01E+08 | ENAH | 4.081665 | 0 | 0 |
| 1.01E+08 | UPK2 | -13.081 | 0 | 0 |
| 1.01E+08 | MCCD1 | 5.790529 | 7.85E-187 | 8.96E-187 |
| 1.01E+08 | AP1S2 | 2.458097 | 0 | 0 |
| 1.01E+08 | RNF186 | 3.885669 | 8.05E-208 | 1.00E-207 |
| 1.01E+08 | SDR9C7 | -5.25286 | 1.13E-54 | 6.12E-55 |
| 1.01E+08 | SLC26A4 | -4.33201 | 0 | 0 |
| 1.01E+08 | ELF3 | -2.6457 | 0 | 0 |
| 1.01E+08 | TAT | -3.93656 | 0 | 0 |
| 1.01E+08 | GPC1 | 3.789777 | 0 | 0 |
| 1.01E+08 | EEF2KMT | -3.08253 | 0 | 0 |
| 1.01E+08 | BZW2 | -3.18473 | 0 | 0 |
| 1.01E+08 | MYO1F | 2.090087 | 0 | 0 |
| 1.01E+08 | ENTPD6 | 2.795653 | 0 | 0 |
| 1.01E+08 | TPST2 | 4.311473 | 0 | 0 |
| 1.01E+08 | CPNE9 | -4.41423 | 9.33E-109 | 7.36E-109 |
| 1.01E+08 | MYOM1 | 2.102137 | 1.32E-42 | 6.32E-43 |
| 1.01E+08 | NIN | -2.46669 | 0 | 0 |
| 1.01E+08 | SLC13A2 | 8.491419 | 0 | 0 |
| 1.01E+08 | UCP2 | -2.42818 | 0 | 0 |
| 1.01E+08 | B4GALT4 | 5.899083 | 0 | 0 |
| 1.01E+08 | APCDD1 | -3.30496 | 0 | 0 |
| 1.01E+08 | ST3GAL5 | 2.202581 | 3.52E-226 | 4.64E-226 |
| 1.01E+08 | PPM1L | -2.89351 | 0 | 0 |
| 1.01E+08 | SMPDL3B | 6.490212 | 0 | 0 |
| 1.01E+08 | ADAMTS10 | 3.413267 | 0 | 0 |
| 1.01E+08 | CSPG4 | -2.41119 | 3.52E-219 | 4.52E-219 |
| 1.01E+08 | ANO9 | 3.058435 | 2.57E-216 | 3.28E-216 |
| 1.01E+08 | HS3ST3A1 | 9.22815 | 0 | 0 |
| 1.01E+08 | ARHGAP31 | 3.456015 | 0 | 0 |
| 1.01E+08 | HOXA10 | -6.20935 | 0 | 0 |
| 1.01E+08 | SLC12A2 | 3.124605 | 0 | 0 |
| 1.01E+08 | PI4K2B | -2.04808 | 0 | 0 |
| 1.01E+08 | LOC101105265 | -7.37455 | 1.44E-146 | 1.37E-146 |
| 1.01E+08 | RGS22 | 4.657394 | 0 | 0 |
| 1.01E+08 | SLC37A4 | -2.03451 | 0 | 0 |
| 1.01E+08 | PCP4L1 | -2.30721 | 1.55E-57 | 8.60E-58 |
| 1.01E+08 | HSD17B6 | -5.08521 | 0 | 0 |
| 1.01E+08 | LOC101105405 | -10.8789 | 1.55E-269 | 2.33E-269 |
| 1.01E+08 | PRTFDC1 | -2.69013 | 1.60E-194 | 1.89E-194 |
| 1.01E+08 | ZNF114 | -2.59975 | 0 | 0 |
| 1.01E+08 | ABAT | -2.28429 | 0 | 0 |
| 1.01E+08 | ARL10 | 3.000255 | 0 | 0 |
| 1.01E+08 | RASGRP1 | 2.624499 | 0 | 0 |
| 1.01E+08 | CST7 | -3.93309 | 3.39E-86 | 2.33E-86 |
| 1.01E+08 | ECM1 | -6.09233 | 0 | 0 |
| 1.01E+08 | C1H3orf58 | -2.34992 | 0 | 0 |
| 1.01E+08 | LOC101105554 | -2.74766 | 0 | 0 |
| 1.01E+08 | SHB | 3.129789 | 0 | 0 |
| 1.01E+08 | GSDMA | -4.19839 | 5.33E-76 | 3.41E-76 |
| 1.01E+08 | EFNA1 | -2.57954 | 0 | 0 |
| 1.01E+08 | VEGFC | 3.667388 | 0 | 0 |
| 1.01E+08 | CTPS2 | -2.35497 | 0 | 0 |
| 1.01E+08 | HOXA9 | -5.72708 | 0 | 0 |
| 1.01E+08 | CD34 | 3.642847 | 0 | 0 |
| 1.01E+08 | TREM1 | 8.814822 | 1.53E-45 | 7.55E-46 |
| 1.01E+08 | HSD3B7 | 2.908115 | 5.28E-67 | 3.15E-67 |
| 1.01E+08 | PSD4 | -2.77095 | 0 | 0 |
| 1.01E+08 | RNASEH2A | -2.13485 | 0 | 0 |
| 1.01E+08 | PDE6H | -3.0274 | 1.79E-27 | 7.09E-28 |
| 1.01E+08 | ENTPD5 | -2.23535 | 0 | 0 |
| 1.01E+08 | MIXL1 | 6.361104 | 9.55E-177 | 1.04E-176 |
| 1.01E+08 | FAM19A4 | -4.35359 | 0 | 0 |
| 1.01E+08 | S100G | 9.370183 | 0 | 0 |
| 1.01E+08 | NRIP3 | 3.395188 | 0 | 0 |
| 1.01E+08 | SLC29A2 | -2.25804 | 2.20E-209 | 2.74E-209 |
| 1.01E+08 | CACUL1 | -2.3439 | 0 | 0 |
| 1.01E+08 | LOC101105976 | 10.01789 | 0 | 0 |
| 1.01E+08 | SH3PXD2B | 2.114382 | 0 | 0 |
| 1.01E+08 | HOXD1 | -2.49806 | 7.26E-61 | 4.13E-61 |
| 1.01E+08 | NCF2 | 3.009741 | 0 | 0 |
| 1.01E+08 | LRIG1 | -2.47416 | 0 | 0 |
| 1.01E+08 | CFAP57 | 2.010114 | 2.60E-115 | 2.12E-115 |
| 1.01E+08 | SLC34A2 | 3.51478 | 0 | 0 |
| 1.01E+08 | SDCBP2 | -4.53145 | 2.89E-124 | 2.49E-124 |
| 1.01E+08 | LOC101106245 | -2.84348 | 4.06E-289 | 6.43E-289 |
| 1.01E+08 | LCN8 | 14.01024 | 0 | 0 |
| 1.01E+08 | FAT2 | -3.00701 | 0 | 0 |
| 1.01E+08 | F2RL1 | -4.49256 | 0 | 0 |
| 1.01E+08 | PGRMC1 | 3.024422 | 0 | 0 |
| 1.01E+08 | LOC101106315 | -2.25915 | 1.94E-255 | 2.78E-255 |
| 1.01E+08 | UCN3 | -7.16621 | 1.30E-17 | 4.40E-18 |
| 1.01E+08 | SRGAP3 | -5.24155 | 0 | 0 |
| 1.01E+08 | CACNA1D | -2.54918 | 0 | 0 |
| 1.01E+08 | SVBP | -2.30984 | 8.04E-292 | 1.28E-291 |
| 1.01E+08 | PLAC9 | -2.24502 | 2.88E-84 | 1.95E-84 |
| 1.01E+08 | LOC101106395 | -3.91851 | 8.17E-73 | 5.10E-73 |
| 1.01E+08 | TFE3 | -2.67478 | 0 | 0 |
| 1.01E+08 | ASL | -2.27666 | 0 | 0 |
| 1.01E+08 | C5H5orf63 | -2.49378 | 5.83E-235 | 7.91E-235 |
| 1.01E+08 | TRIM7 | -4.91819 | 0 | 0 |
| 1.01E+08 | LOC101106452 | 2.043889 | 0 | 0 |
| 1.01E+08 | VSTM5 | -2.78894 | 0 | 0 |
| 1.01E+08 | PLA1A | -2.49161 | 0 | 0 |
| 1.01E+08 | SOWAHB | -2.35433 | 1.30E-249 | 1.83E-249 |
| 1.01E+08 | ABHD12 | 2.070003 | 0 | 0 |
| 1.01E+08 | SYT9 | -4.0057 | 0 | 0 |
| 1.01E+08 | TAMM41 | -2.41048 | 1.48E-259 | 2.15E-259 |
| 1.01E+08 | GCNT2 | 2.188708 | 3.08E-208 | 3.84E-208 |
| 1.01E+08 | LOC101106577 | -2.80203 | 1.28E-134 | 1.16E-134 |
| 1.01E+08 | POLR3G | -2.43063 | 1.92E-107 | 1.50E-107 |
| 1.01E+08 | SLC25A42 | -2.0553 | 1.59E-193 | 1.87E-193 |
| 1.01E+08 | SPRY4 | 3.015865 | 2.26E-300 | 3.68E-300 |
| 1.01E+08 | LOC101106610 | -6.17347 | 0 | 0 |
| 1.01E+08 | EGFLAM | 4.751867 | 0 | 0 |
| 1.01E+08 | SEL1L3 | 4.537002 | 0 | 0 |
| 1.01E+08 | LAMB2 | -2.18488 | 0 | 0 |
| 1.01E+08 | LOC101106720 | -3.77369 | 0 | 0 |
| 1.01E+08 | LOC101106728 | -2.54225 | 0 | 0 |
| 1.01E+08 | LOC101106730 | -2.69554 | 1.35E-300 | 2.20E-300 |
| 1.01E+08 | AMN1 | -2.13325 | 0 | 0 |
| 1.01E+08 | LCN6 | 13.85532 | 0 | 0 |
| 1.01E+08 | DDC | -2.02675 | 0 | 0 |
| 1.01E+08 | CLDN8 | -2.68292 | 0 | 0 |
| 1.01E+08 | ST3GAL6 | 3.59933 | 1.35E-307 | 2.23E-307 |
| 1.01E+08 | LOC101106886 | -2.01963 | 0 | 0 |
| 1.01E+08 | FRMPD1 | 4.808329 | 0 | 0 |
| 1.01E+08 | C21H11orf80 | -3.44759 | 2.60E-239 | 3.56E-239 |
| 1.01E+08 | LOC101106998 | -3.18748 | 0 | 0 |
| 1.01E+08 | ZBED3 | -2.36687 | 0 | 0 |
| 1.01E+08 | IPCEF1 | 3.451606 | 5.74E-123 | 4.89E-123 |
| 1.01E+08 | NR3C2 | -2.42399 | 0 | 0 |
| 1.01E+08 | DCBLD2 | 3.470199 | 0 | 0 |
| 1.01E+08 | ATP13A4 | -3.94089 | 0 | 0 |
| 1.01E+08 | HSPA6 | -2.88323 | 9.03E-166 | 9.37E-166 |
| 1.01E+08 | LOC101107086 | -3.29401 | 0 | 0 |
| 1.01E+08 | SLC16A10 | 4.756253 | 0 | 0 |
| 1.01E+08 | LOC101107119 | -4.63242 | 2.52E-134 | 2.29E-134 |
| 1.01E+08 | BTG4 | 4.509968 | 3.38E-84 | 2.29E-84 |
| 1.01E+08 | GRK5 | -3.5051 | 0 | 0 |
| 1.01E+08 | WDR17 | -2.05112 | 0 | 0 |
| 1.01E+08 | SOX13 | -3.40307 | 0 | 0 |
| 1.01E+08 | LOC101107232 | -3.7936 | 0 | 0 |
| 1.01E+08 | LOC101107260 | -5.57254 | 0 | 0 |
| 1.01E+08 | TSPAN19 | 5.75315 | 2.20E-156 | 2.19E-156 |
| 1.01E+08 | ATP13A5 | -6.10497 | 1.82E-177 | 1.99E-177 |
| 1.01E+08 | SRGN | -2.50589 | 0 | 0 |
| 1.01E+08 | ARMC9 | 2.126608 | 0 | 0 |
| 1.01E+08 | TEN1 | 3.384086 | 1.24E-252 | 1.76E-252 |
| 1.01E+08 | PABPC1L | -5.25055 | 0 | 0 |
| 1.01E+08 | MAATS1 | 2.017764 | 8.71E-193 | 1.02E-192 |
| 1.01E+08 | SMS | 2.193729 | 0 | 0 |
| 1.01E+08 | LOC101107486 | -5.09955 | 1.66E-111 | 1.33E-111 |
| 1.01E+08 | ENTPD1 | -2.01359 | 0 | 0 |
| 1.01E+08 | HDAC7 | -2.21246 | 0 | 0 |
| 1.01E+08 | LTBP2 | -3.9285 | 0 | 0 |
| 1.01E+08 | NBEA | -3.07322 | 0 | 0 |
| 1.01E+08 | PFKFB3 | -2.05997 | 0 | 0 |
| 1.01E+08 | ZNF793 | -2.36918 | 6.44E-112 | 5.17E-112 |
| 1.01E+08 | TMEM144 | -2.3878 | 0 | 0 |
| 1.01E+08 | PTPRG | 2.145349 | 0 | 0 |
| 1.01E+08 | RGS10 | 2.350096 | 1.04E-130 | 9.23E-131 |
| 1.01E+08 | PXYLP1 | 2.737358 | 0 | 0 |
| 1.01E+08 | ATP6V1B1 | 10.93462 | 6.84E-282 | 1.06E-281 |
| 1.01E+08 | HK2 | -3.60188 | 0 | 0 |
| 1.01E+08 | FAM110A | -2.0827 | 1.84E-148 | 1.77E-148 |
| 1.01E+08 | USP13 | -2.00729 | 2.25E-140 | 2.08E-140 |
| 1.01E+08 | LDLRAD4 | 3.105207 | 0 | 0 |
| 1.01E+08 | MAT1A | -3.22927 | 0 | 0 |
| 1.01E+08 | SCARA3 | 2.186821 | 3.30E-207 | 4.09E-207 |
| 1.01E+08 | RENBP | -4.63097 | 0 | 0 |
| 1.01E+08 | ABCB5 | -6.39249 | 0 | 0 |
| 1.01E+08 | SALL2 | -2.07419 | 0 | 0 |
| 1.01E+08 | CPQ | -2.05355 | 0 | 0 |
| 1.01E+08 | CLDN10 | 6.240347 | 0 | 0 |
| 1.01E+08 | C20H6orf136 | -12.2315 | 1.51E-269 | 2.27E-269 |
| 1.01E+08 | VTCN1 | 2.687499 | 2.33E-153 | 2.29E-153 |
| 1.01E+08 | MANBA | -4.18133 | 0 | 0 |
| 1.01E+08 | ASB5 | -2.18667 | 1.87E-52 | 9.89E-53 |
| 1.01E+08 | ARMCX2 | -2.56572 | 0 | 0 |
| 1.01E+08 | RGCC | -2.24466 | 0 | 0 |
| 1.01E+08 | SUSD4 | -5.02152 | 0 | 0 |
| 1.01E+08 | LOC101108071 | -4.01068 | 1.46E-17 | 4.94E-18 |
| 1.01E+08 | CDT1 | 2.508117 | 8.53E-138 | 7.83E-138 |
| 1.01E+08 | PPP1R32 | 6.972435 | 5.37E-175 | 5.81E-175 |
| 1.01E+08 | CARD10 | 2.954565 | 0 | 0 |
| 1.01E+08 | PCDH10 | 5.415461 | 5.74E-144 | 5.42E-144 |
| 1.01E+08 | LRP2 | -2.89956 | 0 | 0 |
| 1.01E+08 | ART3 | -4.33155 | 0 | 0 |
| 1.01E+08 | SERAC1 | -2.13198 | 1.15E-286 | 1.81E-286 |
| 1.01E+08 | LY96 | -2.60479 | 6.26E-173 | 6.71E-173 |
| 1.01E+08 | FUT1 | 6.347957 | 1.91E-220 | 2.46E-220 |
| 1.01E+08 | DYDC2 | 2.113633 | 4.11E-51 | 2.15E-51 |
| 1.01E+08 | TENM3 | 3.703561 | 0 | 0 |
| 1.01E+08 | RAPGEF5 | 2.068817 | 1.05E-134 | 9.58E-135 |
| 1.01E+08 | APRT | 3.855901 | 1.91E-217 | 2.45E-217 |
| 1.01E+08 | FN3K | -2.68756 | 2.86E-66 | 1.70E-66 |
| 1.01E+08 | TNFRSF4 | -2.19824 | 9.22E-62 | 5.29E-62 |
| 1.01E+08 | CDNF | -3.26189 | 5.24E-138 | 4.82E-138 |
| 1.01E+08 | ARRB1 | 2.398972 | 0 | 0 |
| 1.01E+08 | ELOVL5 | -3.37175 | 0 | 0 |
| 1.01E+08 | RGAG4 | -2.00544 | 0 | 0 |
| 1.01E+08 | ELOVL7 | -4.7403 | 0 | 0 |
| 1.01E+08 | HMGN5 | -2.55061 | 2.73E-100 | 2.05E-100 |
| 1.01E+08 | SYCP3 | 2.40521 | 0 | 0 |
| 1.01E+08 | KIAA1211 | -2.76273 | 0 | 0 |
| 1.01E+08 | SNAP25 | -4.78726 | 0 | 0 |
| 1.01E+08 | PFKP | -3.23941 | 0 | 0 |
| 1.01E+08 | PGM5 | -2.04715 | 0 | 0 |
| 1.01E+08 | LOC101108654 | 6.704879 | 0 | 0 |
| 1.01E+08 | TUB | -3.02182 | 2.98E-50 | 1.55E-50 |
| 1.01E+08 | TAOK3 | -2.22637 | 0 | 0 |
| 1.01E+08 | FAM183A | 6.621285 | 5.83E-48 | 2.95E-48 |
| 1.01E+08 | LOC101108705 | -2.14685 | 0 | 0 |
| 1.01E+08 | FAM46C | -2.0221 | 0 | 0 |
| 1.01E+08 | MGAT4C | -9.81812 | 0 | 0 |
| 1.01E+08 | SLC9A2 | 4.422505 | 8.46E-261 | 1.24E-260 |
| 1.01E+08 | FUT5 | -2.17469 | 1.21E-132 | 1.09E-132 |
| 1.01E+08 | GDAP1 | -2.39416 | 6.14E-303 | 1.01E-302 |
| 1.01E+08 | GUSB | -4.07026 | 0 | 0 |
| 1.01E+08 | PTPRU | 2.208872 | 0 | 0 |
| 1.01E+08 | CHPT1 | 3.161192 | 0 | 0 |
| 1.01E+08 | LOC101108901 | 3.880611 | 2.30E-29 | 9.37E-30 |
| 1.01E+08 | PIPOX | 6.574098 | 1.29E-180 | 1.43E-180 |
| 1.01E+08 | IVNS1ABP | 2.152485 | 0 | 0 |
| 1.01E+08 | LOC101108937 | -2.01547 | 0 | 0 |
| 1.01E+08 | CAMK1D | -3.93472 | 0 | 0 |
| 1.01E+08 | PEBP1 | -2.34706 | 0 | 0 |
| 1.01E+08 | RBP1 | 2.591161 | 0 | 0 |
| 1.01E+08 | FABP1 | -4.09582 | 4.55E-34 | 1.97E-34 |
| 1.01E+08 | WIPI1 | -2.26181 | 0 | 0 |
| 1.01E+08 | DEFB129 | -8.54075 | 0 | 0 |
| 1.01E+08 | LOC101109035 | -3.29218 | 9.41E-165 | 9.72E-165 |
| 1.01E+08 | LOC101109079 | -2.15176 | 2.08E-36 | 9.26E-37 |
| 1.01E+08 | FAM216B | 8.031851 | 1.80E-112 | 1.45E-112 |
| 1.01E+08 | MFRP | -3.31559 | 1.79E-159 | 1.81E-159 |
| 1.01E+08 | LOC101109219 | -2.31115 | 2.11E-179 | 2.33E-179 |
| 1.01E+08 | STK32C | 2.264482 | 6.56E-80 | 4.32E-80 |
| 1.01E+08 | SLC31A2 | -3.11834 | 0 | 0 |
| 1.01E+08 | THNSL2 | -2.38151 | 3.28E-131 | 2.93E-131 |
| 1.01E+08 | WIF1 | 5.190142 | 1.66E-191 | 1.94E-191 |
| 1.01E+08 | LOC101109268 | 2.843619 | 1.58E-162 | 1.61E-162 |
| 1.01E+08 | NLRP5 | -5.54389 | 0 | 0 |
| 1.01E+08 | LITAF | 2.623633 | 0 | 0 |
| 1.01E+08 | AKR7A2 | 3.504275 | 0 | 0 |
| 1.01E+08 | LRRC23 | 2.504353 | 2.57E-227 | 3.40E-227 |
| 1.01E+08 | ZNF608 | -2.34508 | 0 | 0 |
| 1.01E+08 | MMD2 | 9.128724 | 0 | 0 |
| 1.01E+08 | WNT7B | -2.87552 | 0 | 0 |
| 1.01E+08 | GALNT10 | -2.93919 | 0 | 0 |
| 1.01E+08 | STBD1 | -2.73161 | 0 | 0 |
| 1.01E+08 | WBSCR17 | -7.6784 | 0 | 0 |
| 1.01E+08 | DLX4 | -4.98981 | 0 | 0 |
| 1.01E+08 | SNN | -2.01727 | 3.34E-267 | 4.96E-267 |
| 1.01E+08 | LOC101109683 | -9.78176 | 3.39E-75 | 2.15E-75 |
| 1.01E+08 | RADIL | 3.450188 | 4.65E-129 | 4.11E-129 |
| 1.01E+08 | ARAP2 | 2.201062 | 0 | 0 |
| 1.01E+08 | CCDC158 | -2.02372 | 0 | 0 |
| 1.01E+08 | SNX22 | -5.40195 | 0 | 0 |
| 1.01E+08 | ADORA1 | 2.202589 | 3.71E-222 | 4.81E-222 |
| 1.01E+08 | DEFB125 | -9.26928 | 0 | 0 |
| 1.01E+08 | SNX7 | -2.01709 | 1.68E-102 | 1.28E-102 |
| 1.01E+08 | GALNT1 | -2.22871 | 0 | 0 |
| 1.01E+08 | TGM5 | -8.9792 | 0 | 0 |
| 1.01E+08 | LOC101109919 | -2.56865 | 5.04E-40 | 2.34E-40 |
| 1.01E+08 | DAGLA | 2.49334 | 6.90E-177 | 7.52E-177 |
| 1.01E+08 | LOC101109939 | -4.20961 | 4.88E-116 | 3.99E-116 |
| 1.01E+08 | TAGAP | -2.13696 | 2.45E-98 | 1.81E-98 |
| 1.01E+08 | L1CAM | -5.92563 | 0 | 0 |
| 1.01E+08 | CPEB1 | 2.208787 | 2.28E-116 | 1.87E-116 |
| 1.01E+08 | NT5C2 | -3.03057 | 0 | 0 |
| 1.01E+08 | CRTAC1 | 2.82596 | 0 | 0 |
| 1.01E+08 | DECR2 | 2.531357 | 3.26E-49 | 1.67E-49 |
| 1.01E+08 | LOC101110116 | -2.07759 | 3.71E-183 | 4.16E-183 |
| 1.01E+08 | LOC101110133 | -2.02088 | 2.96E-48 | 1.50E-48 |
| 1.01E+08 | LOC101110150 | -2.17885 | 0 | 0 |
| 1.01E+08 | LCMT2 | -2.16221 | 7.82E-179 | 8.60E-179 |
| 1.01E+08 | SFTA2 | -3.60057 | 0 | 0 |
| 1.01E+08 | LOC101110185 | -3.57653 | 9.38E-82 | 6.26E-82 |
| 1.01E+08 | THRSP | -6.67355 | 1.07E-187 | 1.22E-187 |
| 1.01E+08 | ILDR2 | -3.8161 | 0 | 0 |
| 1.01E+08 | MLLT1 | -4.15882 | 0 | 0 |
| 1.01E+08 | ZAR1L | -2.38918 | 6.50E-293 | 1.04E-292 |
| 1.01E+08 | MYBPH | 2.351813 | 0 | 0 |
| 1.01E+08 | SPTLC3 | -2.31052 | 0 | 0 |
| 1.01E+08 | FAM221A | -4.52442 | 0 | 0 |
| 1.01E+08 | FILIP1 | -2.55628 | 0 | 0 |
| 1.01E+08 | CA8 | 5.957788 | 0 | 0 |
| 1.01E+08 | ETV4 | 5.901585 | 0 | 0 |
| 1.01E+08 | CACNG4 | 4.400478 | 3.60E-129 | 3.19E-129 |
| 1.01E+08 | DTX4 | -4.44918 | 0 | 0 |
| 1.01E+08 | TSPAN6 | 2.508593 | 0 | 0 |
| 1.01E+08 | SV2C | -6.02585 | 0 | 0 |
| 1.01E+08 | ANKRD13B | -2.32593 | 7.26E-124 | 6.23E-124 |
| 1.01E+08 | CUBN | 3.029453 | 0 | 0 |
| 1.01E+08 | C14H19orf33 | -3.05203 | 2.37E-18 | 8.13E-19 |
| 1.01E+08 | GOLGB1 | -2.4237 | 0 | 0 |
| 1.01E+08 | RNASE13 | -3.71357 | 0 | 0 |
| 1.01E+08 | GPX2 | -5.34933 | 4.43E-181 | 4.93E-181 |
| 1.01E+08 | LOC101110610 | 2.994795 | 0 | 0 |
| 1.01E+08 | PLK2 | -2.38011 | 0 | 0 |
| 1.01E+08 | PFKFB4 | -3.75555 | 0 | 0 |
| 1.01E+08 | TBC1D16 | -2.11339 | 0 | 0 |
| 1.01E+08 | SLC17A9 | 4.537685 | 0 | 0 |
| 1.01E+08 | DGKK | -5.00597 | 0 | 0 |
| 1.01E+08 | POC1B | 3.902551 | 0 | 0 |
| 1.01E+08 | PKIA | 5.00926 | 6.33E-217 | 8.09E-217 |
| 1.01E+08 | HSF4 | -2.79528 | 0 | 0 |
| 1.01E+08 | ELOVL1 | 2.666334 | 0 | 0 |
| 1.01E+08 | SLC7A2 | -4.84304 | 0 | 0 |
| 1.01E+08 | MBNL3 | -3.54162 | 0 | 0 |
| 1.01E+08 | LOC101110855 | -3.78514 | 0 | 0 |
| 1.01E+08 | CAPSL | 6.912608 | 8.46E-141 | 7.86E-141 |
| 1.01E+08 | ISLR | 3.205778 | 0 | 0 |
| 1.01E+08 | PITPNM1 | 4.03601 | 0 | 0 |
| 1.01E+08 | MAPRE2 | -2.34278 | 0 | 0 |
| 1.01E+08 | METRN | -3.68331 | 8.10E-37 | 3.62E-37 |
| 1.01E+08 | PCED1B | -3.43666 | 0 | 0 |
| 1.01E+08 | CCDC126 | -4.78925 | 0 | 0 |
| 1.01E+08 | IQSEC1 | -3.04009 | 6.39E-80 | 4.21E-80 |
| 1.01E+08 | CNDP1 | -3.66621 | 0 | 0 |
| 1.01E+08 | LOC101111006 | 5.17069 | 0 | 0 |
| 1.01E+08 | KLHL42 | -2.04268 | 0 | 0 |
| 1.01E+08 | BEND4 | -4.49901 | 0 | 0 |
| 1.01E+08 | WNT11 | 4.426597 | 6.72E-156 | 6.69E-156 |
| 1.01E+08 | NT5DC2 | 2.066881 | 2.24E-300 | 3.66E-300 |
| 1.01E+08 | EME2 | -2.07988 | 0 | 0 |
| 1.01E+08 | MTMR7 | -4.73935 | 0 | 0 |
| 1.01E+08 | PRELID2 | -2.59662 | 2.70E-207 | 3.35E-207 |
| 1.01E+08 | CD7 | -2.48944 | 2.35E-88 | 1.64E-88 |
| 1.01E+08 | LPAR3 | -5.48209 | 0 | 0 |
| 1.01E+08 | PSTPIP2 | -4.9295 | 0 | 0 |
| 1.01E+08 | FAM173A | -2.68949 | 0 | 0 |
| 1.01E+08 | PTGFRN | -2.51775 | 0 | 0 |
| 1.01E+08 | MAP7D2 | -2.4242 | 0 | 0 |
| 1.01E+08 | TMEM164 | -2.44118 | 3.62E-86 | 2.49E-86 |
| 1.01E+08 | ABCA2 | -2.9712 | 0 | 0 |
| 1.01E+08 | AMIGO2 | -2.71294 | 1.61E-193 | 1.89E-193 |
| 1.01E+08 | ADAMTSL2 | 4.754428 | 0 | 0 |
| 1.01E+08 | ATP2A3 | -2.84539 | 0 | 0 |
| 1.01E+08 | CBX8 | 2.072183 | 0 | 0 |
| 1.01E+08 | DNAJC6 | -5.29087 | 0 | 0 |
| 1.01E+08 | LOC101111242 | 10.94067 | 0 | 0 |
| 1.01E+08 | USP27X | -2.07952 | 1.84E-124 | 1.58E-124 |
| 1.01E+08 | SNX2 | 2.783578 | 0 | 0 |
| 1.01E+08 | CATSPERD | -3.22749 | 1.93E-256 | 2.78E-256 |
| 1.01E+08 | CCDC69 | 3.414619 | 0 | 0 |
| 1.01E+08 | STMN2 | -3.14605 | 4.17E-55 | 2.26E-55 |
| 1.01E+08 | CDH17 | 4.609675 | 0 | 0 |
| 1.01E+08 | WFDC2 | -2.69958 | 0 | 0 |
| 1.01E+08 | LOC101111313 | -9.36728 | 0 | 0 |
| 1.01E+08 | ZDHHC2 | -2.40232 | 1.03E-234 | 1.40E-234 |
| 1.01E+08 | FUT8 | -3.5924 | 0 | 0 |
| 1.01E+08 | KRT23 | -5.63932 | 5.38E-141 | 5.00E-141 |
| 1.01E+08 | PM20D1 | 3.806954 | 8.37E-226 | 1.10E-225 |
| 1.01E+08 | TSPAN18 | -2.46712 | 0 | 0 |
| 1.01E+08 | LOC101111397 | -2.91514 | 0 | 0 |
| 1.01E+08 | CHEK1 | -4.53689 | 1.10E-83 | 7.42E-84 |
| 1.01E+08 | SLC15A2 | 7.93635 | 0 | 0 |
| 1.01E+08 | HAGHL | 3.567144 | 0 | 0 |
| 1.01E+08 | RPS6KA3 | -3.83876 | 0 | 0 |
| 1.01E+08 | NT5DC3 | -3.66973 | 0 | 0 |
| 1.01E+08 | PLA2G4A | 2.638375 | 0 | 0 |
| 1.01E+08 | KCTD15 | 3.201363 | 0 | 0 |
| 1.01E+08 | LOC101111505 | 8.905588 | 7.77E-186 | 8.83E-186 |
| 1.01E+08 | SLC36A3 | -2.72238 | 9.06E-68 | 5.45E-68 |
| 1.01E+08 | NXPH3 | 5.690994 | 0 | 0 |
| 1.01E+08 | OVCH2 | 11.2202 | 0 | 0 |
| 1.01E+08 | MICU3 | -2.01453 | 3.04E-152 | 2.98E-152 |
| 1.01E+08 | S100A14 | -3.88877 | 0 | 0 |
| 1.01E+08 | ID1 | -2.40948 | 2.70E-79 | 1.77E-79 |
| 1.01E+08 | TP53I11 | -4.92519 | 0 | 0 |
| 1.01E+08 | GSTT2B | -2.56769 | 0 | 0 |
| 1.01E+08 | DNPH1 | -2.01181 | 1.43E-214 | 1.82E-214 |
| 1.01E+08 | ROM1 | 2.508278 | 4.72E-187 | 5.40E-187 |
| 1.01E+08 | GALNT8 | -5.81756 | 0 | 0 |
| 1.01E+08 | STEAP1 | 4.408922 | 2.76E-102 | 2.09E-102 |
| 1.01E+08 | SLC22A16 | 4.719683 | 1.36E-189 | 1.57E-189 |
| 1.01E+08 | LOC101111719 | -2.55041 | 0 | 0 |
| 1.01E+08 | PDE4B | 4.341164 | 0 | 0 |
| 1.01E+08 | ATP2A1 | -5.23788 | 5.87E-202 | 7.12E-202 |
| 1.01E+08 | SLC22A2 | 7.894239 | 0 | 0 |
| 1.01E+08 | WNK4 | -2.88725 | 4.65E-223 | 6.05E-223 |
| 1.01E+08 | FNIP2 | -2.14484 | 1.32E-207 | 1.63E-207 |
| 1.01E+08 | STOML1 | -2.10311 | 2.62E-110 | 2.09E-110 |
| 1.01E+08 | TMEM37 | 4.059349 | 6.30E-173 | 6.75E-173 |
| 1.01E+08 | SH3BGRL3 | -2.17162 | 0 | 0 |
| 1.01E+08 | KIAA1211L | -2.0727 | 0 | 0 |
| 1.01E+08 | MYLK2 | -3.77546 | 1.53E-64 | 8.98E-65 |
| 1.01E+08 | HEXB | -3.59331 | 0 | 0 |
| 1.01E+08 | STEAP2 | 5.797484 | 0 | 0 |
| 1.01E+08 | DUOX1 | -4.63003 | 0 | 0 |
| 1.01E+08 | PVRL2 | -2.05002 | 0 | 0 |
| 1.01E+08 | TCN1 | 9.466899 | 3.48E-128 | 3.06E-128 |
| 1.01E+08 | EFCC1 | -3.60368 | 1.23E-48 | 6.29E-49 |
| 1.01E+08 | CLIC6 | 4.148268 | 0 | 0 |
| 1.01E+08 | SMPX | 7.629424 | 2.31E-305 | 3.80E-305 |
| 1.01E+08 | GUCY2F | -8.78416 | 0 | 0 |
| 1.01E+08 | FBXO2 | -2.02602 | 6.56E-73 | 4.10E-73 |
| 1.01E+08 | WFDC13 | -8.70315 | 0 | 0 |
| 1.01E+08 | LOC101112084 | -2.31388 | 0 | 0 |
| 1.01E+08 | TTC21A | 4.978488 | 0 | 0 |
| 1.01E+08 | SCTR | 10.06844 | 0 | 0 |
| 1.01E+08 | CEP85 | -2.01177 | 8.22E-172 | 8.78E-172 |
| 1.01E+08 | LOC101112162 | -5.05929 | 0 | 0 |
| 1.01E+08 | NME6 | -2.06778 | 0 | 0 |
| 1.01E+08 | ABHD11 | -2.34477 | 0 | 0 |
| 1.01E+08 | RAPGEF4 | 2.321559 | 2.21E-147 | 2.12E-147 |
| 1.01E+08 | WDR41 | -2.16037 | 0 | 0 |
| 1.01E+08 | MAP7 | 3.592969 | 0 | 0 |
| 1.01E+08 | LOC101112270 | -2.30836 | 0 | 0 |
| 1.01E+08 | GATSL2 | 3.279383 | 0 | 0 |
| 1.01E+08 | LOC101112287 | 2.361262 | 0 | 0 |
| 1.01E+08 | LOC101112296 | -5.14999 | 1.65E-189 | 1.90E-189 |
| 1.01E+08 | SPINT4 | -9.5241 | 0 | 0 |
| 1.01E+08 | RCN1 | 5.786452 | 0 | 0 |
| 1.01E+08 | CFAP221 | 3.677512 | 8.88E-184 | 1.00E-183 |
| 1.01E+08 | MON2 | 2.969027 | 0 | 0 |
| 1.01E+08 | RAC3 | -3.06897 | 1.31E-103 | 1.00E-103 |
| 1.01E+08 | NEFM | -4.91112 | 0 | 0 |
| 1.01E+08 | TMEM255A | -3.91878 | 0 | 0 |
| 1.01E+08 | IRS4 | -7.49383 | 0 | 0 |
| 1.01E+08 | LOC101112483 | -2.81352 | 0 | 0 |
| 1.01E+08 | AADACL3 | 2.697078 | 4.26E-53 | 2.27E-53 |
| 1.01E+08 | PLP2 | -2.43248 | 0 | 0 |
| 1.01E+08 | RYR1 | -6.44834 | 0 | 0 |
| 1.01E+08 | PDGFC | 2.809508 | 0 | 0 |
| 1.01E+08 | LONRF1 | -3.20817 | 0 | 0 |
| 1.01E+08 | IL20RA | -2.70972 | 0 | 0 |
| 1.01E+08 | CST6 | 6.708588 | 0 | 0 |
| 1.01E+08 | CTSC | 2.962399 | 0 | 0 |
| 1.01E+08 | PRDX6 | 4.267135 | 0 | 0 |
| 1.01E+08 | MS4A7 | 9.749414 | 0 | 0 |
| 1.01E+08 | CSTA | -6.42924 | 1.75E-41 | 8.23E-42 |
| 1.01E+08 | PRDX4 | -2.61193 | 0 | 0 |
| 1.01E+08 | BHLHE41 | 2.462831 | 0 | 0 |
| 1.01E+08 | JAK3 | 2.180238 | 6.11E-129 | 5.39E-129 |
| 1.01E+08 | PAG1 | -2.06913 | 7.01E-269 | 1.05E-268 |
| 1.01E+08 | RASL10B | -3.76331 | 0 | 0 |
| 1.01E+08 | SNX21 | -2.32013 | 0 | 0 |
| 1.01E+08 | LOC101112843 | -2.53919 | 1.09E-251 | 1.55E-251 |
| 1.01E+08 | CEP164 | -2.21507 | 0 | 0 |
| 1.01E+08 | FCRL3 | -2.94285 | 0 | 0 |
| 1.01E+08 | INPP5D | 4.365551 | 0 | 0 |
| 1.01E+08 | STC1 | -4.05692 | 0 | 0 |
| 1.01E+08 | PXDN | 2.054949 | 6.07E-177 | 6.61E-177 |
| 1.01E+08 | TSPAN5 | -2.15098 | 0 | 0 |
| 1.01E+08 | LOC101112981 | -5.59589 | 1.28E-80 | 8.45E-81 |
| 1.01E+08 | CBLN3 | -2.16855 | 0 | 0 |
| 1.01E+08 | FAM78B | 6.100261 | 4.71E-214 | 5.96E-214 |
| 1.01E+08 | PLB1 | -3.6539 | 1.72E-65 | 1.02E-65 |
| 1.01E+08 | LOC101113073 | -8.0097 | 0 | 0 |
| 1.01E+08 | CD3EAP | -2.07016 | 0 | 0 |
| 1.01E+08 | FMN2 | -5.08902 | 1.64E-134 | 1.49E-134 |
| 1.01E+08 | HABP4 | -2.2246 | 0 | 0 |
| 1.01E+08 | GPX4 | -2.629 | 0 | 0 |
| 1.01E+08 | PYCR1 | 2.734889 | 0 | 0 |
| 1.01E+08 | S100B | 6.226852 | 0 | 0 |
| 1.01E+08 | CD164L2 | 2.576767 | 1.04E-51 | 5.46E-52 |
| 1.01E+08 | LTB4R | 4.748536 | 0 | 0 |
| 1.01E+08 | TMEM63C | -2.47872 | 0 | 0 |
| 1.01E+08 | LOC101113259 | -4.79495 | 9.97E-50 | 5.15E-50 |
| 1.01E+08 | LRRC36 | 6.217629 | 0 | 0 |
| 1.01E+08 | C7H15orf52 | -3.09139 | 0 | 0 |
| 1.01E+08 | LOC101113341 | 5.330786 | 0 | 0 |
| 1.01E+08 | LOC101113357 | -5.34646 | 1.22E-86 | 8.44E-87 |
| 1.01E+08 | ADGRD1 | -4.87916 | 0 | 0 |
| 1.01E+08 | CLDN23 | -5.19142 | 0 | 0 |
| 1.01E+08 | EXTL1 | -8.07184 | 0 | 0 |
| 1.01E+08 | ZNF367 | -2.40694 | 0 | 0 |
| 1.01E+08 | PPP2R2B | 4.176126 | 0 | 0 |
| 1.01E+08 | NIPAL4 | -3.81561 | 3.98E-278 | 6.09E-278 |
| 1.01E+08 | FBXW4 | -2.17263 | 0 | 0 |
| 1.01E+08 | SLC1A4 | 4.531966 | 0 | 0 |
| 1.01E+08 | SS18L1 | -2.10444 | 8.07E-195 | 9.52E-195 |
| 1.01E+08 | TPPP3 | 3.545641 | 0 | 0 |
| 1.01E+08 | THEG | 7.263807 | 1.08E-209 | 1.35E-209 |
| 1.01E+08 | DCUN1D4 | -2.84001 | 0 | 0 |
| 1.01E+08 | TMEM201 | -2.98725 | 0 | 0 |
| 1.01E+08 | DDAH1 | 3.116319 | 0 | 0 |
| 1.01E+08 | KIF5C | 3.031607 | 0 | 0 |
| 1.01E+08 | RIN1 | -2.58712 | 1.14E-185 | 1.30E-185 |
| 1.01E+08 | GALNT7 | -2.97262 | 0 | 0 |
| 1.01E+08 | MAP3K6 | -2.57654 | 0 | 0 |
| 1.01E+08 | TEX30 | 2.050332 | 8.11E-191 | 9.41E-191 |
| 1.01E+08 | HRASLS5 | -2.24752 | 0 | 0 |
| 1.01E+08 | LOC101113823 | 10.56484 | 7.03E-232 | 9.44E-232 |
| 1.01E+08 | PARD3B | -2.30478 | 0 | 0 |
| 1.01E+08 | LOC101113860 | 5.266854 | 7.52E-75 | 4.77E-75 |
| 1.01E+08 | MSRB1 | -2.43828 | 0 | 0 |
| 1.01E+08 | ANKRD23 | -2.2306 | 1.59E-205 | 1.96E-205 |
| 1.01E+08 | SYTL1 | -3.14502 | 0 | 0 |
| 1.01E+08 | SLC4A8 | -3.65199 | 0 | 0 |
| 1.01E+08 | ABO | 3.270338 | 1.73E-158 | 1.74E-158 |
| 1.01E+08 | SLC30A4 | -2.12899 | 0 | 0 |
| 1.01E+08 | PDE10A | -4.681 | 0 | 0 |
| 1.01E+08 | LOC101114042 | 3.781854 | 0 | 0 |
| 1.01E+08 | CUL9 | -2.28034 | 0 | 0 |
| 1.01E+08 | LOC101114082 | 2.893423 | 0 | 0 |
| 1.01E+08 | SPATA18 | 4.58171 | 0 | 0 |
| 1.01E+08 | TMEM229B | -3.97177 | 0 | 0 |
| 1.01E+08 | RRAGD | -3.57365 | 0 | 0 |
| 1.01E+08 | H6PD | -2.43909 | 0 | 0 |
| 1.01E+08 | ACSS2 | -3.96965 | 0 | 0 |
| 1.01E+08 | CXHXorf58 | -2.30207 | 8.09E-106 | 6.26E-106 |
| 1.01E+08 | C5H19orf24 | -2.9676 | 1.14E-153 | 1.12E-153 |
| 1.01E+08 | RASGEF1B | -2.39494 | 0 | 0 |
| 1.01E+08 | OAT | -2.43553 | 0 | 0 |
| 1.01E+08 | PPP2R3A | -2.17647 | 0 | 0 |
| 1.01E+08 | NCK2 | -2.76501 | 0 | 0 |
| 1.01E+08 | ACTG2 | -2.0499 | 0 | 0 |
| 1.01E+08 | ZBTB49 | -2.39103 | 0 | 0 |
| 1.01E+08 | WFDC11 | -3.08957 | 0 | 0 |
| 1.01E+08 | NKPD1 | -4.33199 | 0 | 0 |
| 1.01E+08 | CRIP3 | 2.846644 | 9.78E-184 | 1.10E-183 |
| 1.01E+08 | HTATIP2 | -3.334 | 0 | 0 |
| 1.01E+08 | PARP14 | 2.025658 | 0 | 0 |
| 1.01E+08 | SYT2 | -4.6947 | 4.27E-159 | 4.30E-159 |
| 1.01E+08 | PLIN4 | -3.27971 | 5.79E-232 | 7.78E-232 |
| 1.01E+08 | NOVA1 | -2.26191 | 9.23E-301 | 1.51E-300 |
| 1.01E+08 | LOC101114419 | -3.06492 | 7.68E-190 | 8.88E-190 |
| 1.01E+08 | GPR37L1 | -2.55693 | 2.86E-45 | 1.41E-45 |
| 1.01E+08 | PMFBP1 | -3.74221 | 8.83E-230 | 1.18E-229 |
| 1.01E+08 | ENTPD3 | -6.16055 | 0 | 0 |
| 1.01E+08 | DCLK3 | 5.198338 | 2.86E-253 | 4.06E-253 |
| 1.01E+08 | TMC8 | 2.417164 | 5.38E-133 | 4.86E-133 |
| 1.01E+08 | GSS | -3.82855 | 0 | 0 |
| 1.01E+08 | KLHL31 | -7.03505 | 0 | 0 |
| 1.01E+08 | BCL9 | 2.040469 | 0 | 0 |
| 1.01E+08 | TSC22D3 | -2.20882 | 0 | 0 |
| 1.01E+08 | OBSL1 | -2.7108 | 0 | 0 |
| 1.01E+08 | KCNIP3 | -3.92316 | 0 | 0 |
| 1.01E+08 | ZBTB7C | 3.369287 | 0 | 0 |
| 1.01E+08 | EPHB1 | 4.217588 | 0 | 0 |
| 1.01E+08 | DMD | -2.24403 | 0 | 0 |
| 1.01E+08 | LOC101114720 | -5.90729 | 9.92E-30 | 4.06E-30 |
| 1.01E+08 | SLC41A3 | -2.53561 | 1.47E-134 | 1.34E-134 |
| 1.01E+08 | ACP6 | 2.888063 | 0 | 0 |
| 1.01E+08 | CREG1 | -2.0684 | 0 | 0 |
| 1.01E+08 | CELF4 | 5.720974 | 0 | 0 |
| 1.01E+08 | GALNT6 | 2.988585 | 0 | 0 |
| 1.01E+08 | LOC101114861 | 8.595549 | 0 | 0 |
| 1.01E+08 | CHGA | 5.75315 | 0 | 0 |
| 1.01E+08 | ATF5 | 2.479376 | 0 | 0 |
| 1.01E+08 | ASS1 | -5.80539 | 0 | 0 |
| 1.01E+08 | NOTCH1 | -2.26002 | 8.65E-260 | 1.26E-259 |
| 1.01E+08 | GRIP1 | -3.79276 | 0 | 0 |
| 1.01E+08 | DUOXA1 | -9.10218 | 2.22E-202 | 2.70E-202 |
| 1.01E+08 | MUC15 | 4.27999 | 0 | 0 |
| 1.01E+08 | CDR2 | -2.46533 | 0 | 0 |
| 1.01E+08 | WDR86 | 2.989211 | 4.32E-214 | 5.47E-214 |
| 1.01E+08 | TMEM150C | 4.959344 | 0 | 0 |
| 1.01E+08 | GJB7 | -2.61748 | 1.34E-145 | 1.28E-145 |
| 1.01E+08 | LOC101115136 | -2.62055 | 1.53E-186 | 1.74E-186 |
| 1.01E+08 | RNF144A | 4.165238 | 1.93E-231 | 2.59E-231 |
| 1.01E+08 | SLC5A6 | -2.04794 | 0 | 0 |
| 1.01E+08 | GLI4 | -2.22688 | 0 | 0 |
| 1.01E+08 | RABGAP1L | -2.31194 | 0 | 0 |
| 1.01E+08 | SLC5A12 | -2.41446 | 0 | 0 |
| 1.01E+08 | KLF15 | -2.72989 | 0 | 0 |
| 1.01E+08 | LOC101115343 | 3.869964 | 0 | 0 |
| 1.01E+08 | PLA2G4B | 3.375517 | 0 | 0 |
| 1.01E+08 | SHISA2 | 2.930172 | 0 | 0 |
| 1.01E+08 | EFNA3 | -3.98179 | 1.52E-229 | 2.03E-229 |
| 1.01E+08 | ETV5 | 3.704625 | 0 | 0 |
| 1.01E+08 | APC2 | -2.36179 | 8.22E-276 | 1.25E-275 |
| 1.01E+08 | GRN | -2.74699 | 0 | 0 |
| 1.01E+08 | RUNX2 | 4.122319 | 0 | 0 |
| 1.01E+08 | MLXIPL | -2.45743 | 0 | 0 |
| 1.01E+08 | SIDT1 | 2.876057 | 1.83E-151 | 1.79E-151 |
| 1.01E+08 | LOC101115527 | -4.76978 | 0 | 0 |
| 1.01E+08 | GRAMD2 | -3.54976 | 0 | 0 |
| 1.01E+08 | HS3ST3B1 | 6.884675 | 0 | 0 |
| 1.01E+08 | BATF3 | -3.2292 | 2.58E-201 | 3.12E-201 |
| 1.01E+08 | MOCOS | -2.20782 | 0 | 0 |
| 1.01E+08 | GPRIN2 | 2.638382 | 5.86E-47 | 2.94E-47 |
| 1.01E+08 | GALNT13 | -4.53145 | 2.89E-124 | 2.49E-124 |
| 1.01E+08 | STYK1 | -3.62047 | 1.26E-132 | 1.14E-132 |
| 1.01E+08 | CHRNA9 | 8.478342 | 0 | 0 |
| 1.01E+08 | RHBDL3 | 3.687368 | 0 | 0 |
| 1.01E+08 | NRG4 | -4.50579 | 0 | 0 |
| 1.01E+08 | SUN1 | -2.32958 | 0 | 0 |
| 1.01E+08 | METTL7B | -5.69154 | 0 | 0 |
| 1.01E+08 | DUSP2 | 9.420731 | 0 | 0 |
| 1.01E+08 | KLHL33 | -4.64007 | 0 | 0 |
| 1.01E+08 | ZNF536 | 5.157944 | 0 | 0 |
| 1.01E+08 | LOC101115732 | 3.793827 | 0 | 0 |
| 1.01E+08 | CAD | -2.66098 | 0 | 0 |
| 1.01E+08 | C5H19orf38 | -2.41818 | 1.96E-58 | 1.10E-58 |
| 1.01E+08 | EFNA5 | -3.73885 | 0 | 0 |
| 1.01E+08 | CCNO | -2.51924 | 1.78E-99 | 1.33E-99 |
| 1.01E+08 | DAO | -2.48894 | 0 | 0 |
| 1.01E+08 | CLMN | -2.21908 | 0 | 0 |
| 1.01E+08 | SYT17 | 5.310817 | 0 | 0 |
| 1.01E+08 | PDE3A | 4.096198 | 2.24E-277 | 3.41E-277 |
| 1.01E+08 | CALR3 | -2.36909 | 2.43E-205 | 2.99E-205 |
| 1.01E+08 | ACTN3 | 2.375082 | 2.56E-103 | 1.95E-103 |
| 1.01E+08 | PLK5 | -5.32477 | 0 | 0 |
| 1.01E+08 | LOC101115988 | 4.144925 | 7.31E-136 | 6.68E-136 |
| 1.01E+08 | SLC44A4 | -2.06483 | 0 | 0 |
| 1.01E+08 | C26H8orf4 | 2.174269 | 6.91E-228 | 9.16E-228 |
| 1.01E+08 | AIM1L | -4.1367 | 3.69E-224 | 4.82E-224 |
| 1.01E+08 | DNAJC5G | -4.98122 | 2.30E-239 | 3.15E-239 |
| 1.01E+08 | TMEM173 | -2.02444 | 0 | 0 |
| 1.01E+08 | FABP6 | -8.38122 | 0 | 0 |
| 1.01E+08 | SLC16A6 | -5.09957 | 0 | 0 |
| 1.01E+08 | PDZK1 | 7.97506 | 0 | 0 |
| 1.01E+08 | ROPN1 | 7.129989 | 0 | 0 |
| 1.01E+08 | RIPPLY1 | 6.28139 | 4.46E-94 | 3.22E-94 |
| 1.01E+08 | DCDC2B | 2.375882 | 2.13E-167 | 2.23E-167 |
| 1.01E+08 | XYLB | 3.190355 | 0 | 0 |
| 1.01E+08 | CARS2 | 3.356176 | 0 | 0 |
| 1.01E+08 | MXRA7 | 2.030877 | 0 | 0 |
| 1.01E+08 | TRIM29 | 5.426156 | 0 | 0 |
| 1.01E+08 | NEU1 | -2.41563 | 0 | 0 |
| 1.01E+08 | PROM2 | -8.96922 | 0 | 0 |
| 1.01E+08 | EPPK1 | 2.4578 | 0 | 0 |
| 1.01E+08 | HIGD1B | -2.49038 | 3.08E-35 | 1.35E-35 |
| 1.01E+08 | SRGAP2 | -2.5052 | 0 | 0 |
| 1.01E+08 | ANO1 | -2.98416 | 0 | 0 |
| 1.01E+08 | NCMAP | 7.691031 | 0 | 0 |
| 1.01E+08 | PLCXD1 | -3.87425 | 0 | 0 |
| 1.01E+08 | ABCA7 | -4.11757 | 0 | 0 |
| 1.01E+08 | CXXC5 | 4.728252 | 0 | 0 |
| 1.01E+08 | LOC101116570 | -9.69735 | 8.69E-142 | 8.11E-142 |
| 1.01E+08 | ST6GALNAC1 | -6.50192 | 0 | 0 |
| 1.01E+08 | DEPTOR | -2.65754 | 4.22E-292 | 6.71E-292 |
| 1.01E+08 | RNASE11 | -3.75581 | 0 | 0 |
| 1.01E+08 | TONSL | -4.02405 | 0 | 0 |
| 1.01E+08 | LIPH | 4.43541 | 2.04E-177 | 2.23E-177 |
| 1.01E+08 | DOC2A | 7.476143 | 2.02E-120 | 1.70E-120 |
| 1.01E+08 | CIART | -2.90723 | 8.87E-247 | 1.24E-246 |
| 1.01E+08 | LOC101116795 | 8.51999 | 0 | 0 |
| 1.01E+08 | SLC28A3 | 4.679469 | 0 | 0 |
| 1.01E+08 | CDK16 | -2.20611 | 0 | 0 |
| 1.01E+08 | FMNL2 | -2.6754 | 0 | 0 |
| 1.01E+08 | AFF2 | 4.119927 | 0 | 0 |
| 1.01E+08 | GPSM1 | -3.16964 | 0 | 0 |
| 1.01E+08 | LOC101116828 | 3.632686 | 0 | 0 |
| 1.01E+08 | LOC101116841 | 6.385605 | 1.35E-179 | 1.49E-179 |
| 1.01E+08 | FGF9 | -2.34276 | 0 | 0 |
| 1.01E+08 | ZNF41 | -2.55344 | 0 | 0 |
| 1.01E+08 | TXNDC16 | -2.01497 | 0 | 0 |
| 1.01E+08 | SACS | -4.15846 | 0 | 0 |
| 1.01E+08 | LOC101116937 | -2.16106 | 0 | 0 |
| 1.01E+08 | HIP1 | -3.31179 | 0 | 0 |
| 1.01E+08 | PRPSAP1 | -2.39978 | 0 | 0 |
| 1.01E+08 | C13H20orf85 | 4.760086 | 4.55E-33 | 1.94E-33 |
| 1.01E+08 | LOC101117129 | -4.77976 | 0 | 0 |
| 1.01E+08 | ZNF157 | -2.25745 | 1.16E-208 | 1.45E-208 |
| 1.01E+08 | LOC101117144 | -5.64574 | 2.37E-118 | 1.97E-118 |
| 1.01E+08 | RTKN | 2.619951 | 4.09E-137 | 3.74E-137 |
| 1.01E+08 | VWDE | 6.326478 | 7.32E-116 | 5.97E-116 |
| 1.01E+08 | MATK | 3.033462 | 1.27E-78 | 8.30E-79 |
| 1.01E+08 | SPTBN5 | 2.862445 | 0 | 0 |
| 1.01E+08 | RAB11FIP4 | -3.16734 | 0 | 0 |
| 1.01E+08 | NMT2 | 2.041076 | 1.30E-98 | 9.67E-99 |
| 1.01E+08 | LOC101117184 | 2.542072 | 5.41E-68 | 3.26E-68 |
| 1.01E+08 | STARD5 | -2.1131 | 2.64E-121 | 2.23E-121 |
| 1.01E+08 | GLIS1 | -4.827 | 2.62E-213 | 3.31E-213 |
| 1.01E+08 | RNF128 | 2.870279 | 0 | 0 |
| 1.01E+08 | VGLL1 | -6.53278 | 1.00E-86 | 6.92E-87 |
| 1.01E+08 | FLT4 | -4.59061 | 0 | 0 |
| 1.01E+08 | JPH1 | -3.18842 | 0 | 0 |
| 1.01E+08 | SERPINF2 | 3.064974 | 0 | 0 |
| 1.01E+08 | CCL26 | -4.46014 | 6.38E-108 | 5.01E-108 |
| 1.01E+08 | LOC101117299 | -3.24532 | 0 | 0 |
| 1.01E+08 | SLC30A2 | -6.00732 | 2.55E-151 | 2.49E-151 |
| 1.01E+08 | SLC16A11 | -3.17069 | 2.79E-302 | 4.56E-302 |
| 1.01E+08 | EGLN3 | 2.848902 | 0 | 0 |
| 1.01E+08 | LOXL4 | -4.95439 | 0 | 0 |
| 1.01E+08 | TAF4B | -4.36638 | 0 | 0 |
| 1.01E+08 | CCDC178 | 8.596068 | 0 | 0 |
| 1.01E+08 | BUB1 | -3.76457 | 0 | 0 |
| 1.01E+08 | CLRN3 | 9.734563 | 3.65E-75 | 2.32E-75 |
| 1.01E+08 | NRIP1 | -2.39322 | 0 | 0 |
| 1.01E+08 | PRSS27 | 7.253092 | 9.64E-71 | 5.94E-71 |
| 1.01E+08 | GLDC | -3.82129 | 0 | 0 |
| 1.01E+08 | LOC101117482 | 7.502489 | 0 | 0 |
| 1.01E+08 | TPM4 | -2.40873 | 0 | 0 |
| 1.01E+08 | SLC29A1 | -2.76683 | 0 | 0 |
| 1.01E+08 | MRO | -3.58232 | 2.01E-178 | 2.21E-178 |
| 1.01E+08 | STAT4 | 3.147645 | 5.54E-155 | 5.49E-155 |
| 1.01E+08 | LOC101117587 | -3.44457 | 0 | 0 |
| 1.01E+08 | MRAP2 | -2.91932 | 1.02E-121 | 8.59E-122 |
| 1.01E+08 | TOX3 | -3.43377 | 0 | 0 |
| 1.01E+08 | PPARD | -2.26465 | 0 | 0 |
| 1.01E+08 | PYROXD2 | -3.12928 | 7.40E-171 | 7.86E-171 |
| 1.01E+08 | KLHL14 | -3.36845 | 0 | 0 |
| 1.01E+08 | P2RY14 | 4.137095 | 0 | 0 |
| 1.01E+08 | SHH | -3.44191 | 3.43E-165 | 3.55E-165 |
| 1.01E+08 | KRT15 | -3.15972 | 0 | 0 |
| 1.01E+08 | ULK1 | -2.30631 | 0 | 0 |
| 1.01E+08 | UNC13B | -2.20585 | 0 | 0 |
| 1.01E+08 | LOC101117767 | -2.6575 | 0 | 0 |
| 1.01E+08 | RHBDD2 | -2.10717 | 0 | 0 |
| 1.01E+08 | SH2B2 | -2.986 | 3.04E-249 | 4.26E-249 |
| 1.01E+08 | NACC2 | -2.72731 | 2.55E-286 | 4.01E-286 |
| 1.01E+08 | MAN2B2 | -3.41421 | 0 | 0 |
| 1.01E+08 | CA6 | -7.38033 | 2.39E-183 | 2.69E-183 |
| 1.01E+08 | CFAP100 | -2.42413 | 0 | 0 |
| 1.01E+08 | MSH5 | -2.89967 | 0 | 0 |
| 1.01E+08 | RNF207 | -4.69809 | 7.36E-269 | 1.10E-268 |
| 1.01E+08 | SORL1 | -2.28976 | 0 | 0 |
| 1.01E+08 | MMP17 | -6.82265 | 6.61E-232 | 8.87E-232 |
| 1.01E+08 | RHBDL2 | 3.083069 | 0 | 0 |
| 1.01E+08 | LOC101118004 | -9.90111 | 3.61E-80 | 2.38E-80 |
| 1.01E+08 | ACOXL | -5.40685 | 0 | 0 |
| 1.01E+08 | ODF3B | 6.89381 | 2.01E-15 | 6.50E-16 |
| 1.01E+08 | TTC9B | -5.21684 | 1.84E-70 | 1.13E-70 |
| 1.01E+08 | INHA | -5.73787 | 0 | 0 |
| 1.01E+08 | POLM | -2.4755 | 8.52E-73 | 5.32E-73 |
| 1.01E+08 | MROH5 | -3.28197 | 6.49E-139 | 5.99E-139 |
| 1.01E+08 | IRX3 | -6.0127 | 0 | 0 |
| 1.01E+08 | EVL | -2.99431 | 0 | 0 |
| 1.01E+08 | RHOC | -3.06173 | 0 | 0 |
| 1.01E+08 | C2H9orf64 | 2.51499 | 2.86E-149 | 2.77E-149 |
| 1.01E+08 | LOC101118216 | -7.72664 | 0 | 0 |
| 1.01E+08 | FGL2 | -3.37534 | 0 | 0 |
| 1.01E+08 | OCLN | 2.235792 | 0 | 0 |
| 1.01E+08 | KIAA0319 | -3.29583 | 0 | 0 |
| 1.01E+08 | DHCR7 | -2.68187 | 0 | 0 |
| 1.01E+08 | STYXL1 | -2.85851 | 2.22E-198 | 2.65E-198 |
| 1.01E+08 | PLAT | -2.37053 | 0 | 0 |
| 1.01E+08 | TNC | -4.1492 | 0 | 0 |
| 1.01E+08 | KCNT1 | 4.405144 | 1.22E-181 | 1.37E-181 |
| 1.01E+08 | FUCA2 | -3.57322 | 0 | 0 |
| 1.01E+08 | NUDCD1 | -2.13372 | 0 | 0 |
| 1.01E+08 | IRX5 | -6.9096 | 4.96E-191 | 5.77E-191 |
| 1.01E+08 | DEGS2 | -5.67113 | 4.97E-73 | 3.11E-73 |
| 1.01E+08 | FAM167A | -4.33208 | 0 | 0 |
| 1.01E+08 | KCNH3 | -5.15081 | 0 | 0 |
| 1.01E+08 | LOC101118448 | -2.45082 | 3.50E-59 | 1.97E-59 |
| 1.01E+08 | ST8SIA6 | 9.869252 | 0 | 0 |
| 1.01E+08 | CTH | -3.8655 | 0 | 0 |
| 1.01E+08 | SLC2A10 | 2.765965 | 0 | 0 |
| 1.01E+08 | PLD3 | -4.53385 | 0 | 0 |
| 1.01E+08 | LCN9 | 11.77409 | 0 | 0 |
| 1.01E+08 | NFATC4 | -2.67473 | 2.79E-260 | 4.07E-260 |
| 1.01E+08 | ACOX1 | 3.510771 | 0 | 0 |
| 1.01E+08 | PHF8 | -2.44786 | 0 | 0 |
| 1.01E+08 | LBX2 | 6.652372 | 1.86E-119 | 1.55E-119 |
| 1.01E+08 | EDIL3 | -5.65053 | 0 | 0 |
| 1.01E+08 | SCML4 | -3.39327 | 0 | 0 |
| 1.01E+08 | CRYL1 | -2.52811 | 0 | 0 |
| 1.01E+08 | PITPNM2 | -2.60117 | 0 | 0 |
| 1.01E+08 | DUSP5 | 2.477948 | 4.95E-268 | 7.37E-268 |
| 1.01E+08 | STEAP3 | 3.548036 | 1.55E-104 | 1.19E-104 |
| 1.01E+08 | FAM117A | -2.48658 | 0 | 0 |
| 1.01E+08 | EYA2 | -3.13208 | 3.83E-282 | 5.95E-282 |
| 1.01E+08 | SHANK1 | 4.848902 | 1.20E-155 | 1.19E-155 |
| 1.01E+08 | ARMC12 | -3.43378 | 0 | 0 |
| 1.01E+08 | CLRN1 | -9.61057 | 1.42E-68 | 8.60E-69 |
| 1.01E+08 | TPD52L1 | 3.691475 | 0 | 0 |
| 1.01E+08 | FBF1 | 3.473131 | 0 | 0 |
| 1.01E+08 | CRYBB1 | 3.928911 | 0 | 0 |
| 1.01E+08 | ABLIM3 | -3.4815 | 0 | 0 |
| 1.01E+08 | GNA15 | 3.582033 | 0 | 0 |
| 1.01E+08 | PLEKHD1 | -3.60908 | 6.35E-157 | 6.34E-157 |
| 1.01E+08 | SMOX | -2.39806 | 0 | 0 |
| 1.01E+08 | ADAMTS18 | 2.610904 | 0 | 0 |
| 1.01E+08 | CNTD2 | -2.14862 | 0 | 0 |
| 1.01E+08 | MPPED2 | -3.83778 | 0 | 0 |
| 1.01E+08 | LOC101118990 | -3.65035 | 0 | 0 |
| 1.01E+08 | CLEC11A | 2.836349 | 1.54E-64 | 9.03E-65 |
| 1.01E+08 | HPDL | -3.74928 | 3.05E-52 | 1.61E-52 |
| 1.01E+08 | AFAP1L2 | -3.27732 | 0 | 0 |
| 1.01E+08 | GPR160 | 3.213515 | 0 | 0 |
| 1.01E+08 | SLC25A47 | -2.60314 | 8.18E-78 | 5.31E-78 |
| 1.01E+08 | ADAMTSL5 | -4.07573 | 0 | 0 |
| 1.01E+08 | BEND3 | -2.37047 | 3.21E-67 | 1.93E-67 |
| 1.01E+08 | LYNX1 | 4.175124 | 0 | 0 |
| 1.01E+08 | LHFPL5 | -4.37984 | 7.68E-264 | 1.13E-263 |
| 1.01E+08 | COL22A1 | 4.998517 | 0 | 0 |
| 1.01E+08 | PDZD2 | -2.6493 | 0 | 0 |
| 1.01E+08 | GPHA2 | -8.38216 | 1.32E-45 | 6.54E-46 |
| 1.01E+08 | GNAO1 | -3.09159 | 0 | 0 |
| 1.01E+08 | SMPD3 | -4.68982 | 0 | 0 |
| 1.01E+08 | SHISA5 | -3.15282 | 0 | 0 |
| 1.01E+08 | SMARCA1 | -3.88606 | 0 | 0 |
| 1.01E+08 | KCNQ1 | -4.27042 | 0 | 0 |
| 1.01E+08 | TAGLN3 | -7.11471 | 0 | 0 |
| 1.01E+08 | GLRA3 | -7.51985 | 0 | 0 |
| 1.01E+08 | LOC101119648 | 2.211883 | 1.03E-193 | 1.21E-193 |
| 1.01E+08 | CHMP4C | -2.20405 | 5.62E-279 | 8.63E-279 |
| 1.01E+08 | CLEC12B | -10.9865 | 0 | 0 |
| 1.01E+08 | PCYOX1L | -2.34307 | 1.78E-154 | 1.76E-154 |
| 1.01E+08 | ARHGEF16 | 2.561187 | 1.66E-155 | 1.64E-155 |
| 1.01E+08 | BLVRB | -2.24942 | 0 | 0 |
| 1.01E+08 | RET | -2.76024 | 0 | 0 |
| 1.01E+08 | LOC101119804 | -4.56976 | 1.55E-146 | 1.48E-146 |
| 1.01E+08 | PPM1K | -2.46542 | 0 | 0 |
| 1.01E+08 | ITGA2 | 4.046009 | 0 | 0 |
| 1.01E+08 | LY6G5B | 10.79109 | 0 | 0 |
| 1.01E+08 | IFNLR1 | -2.28962 | 1.20E-106 | 9.31E-107 |
| 1.01E+08 | GPD1 | -3.04661 | 0 | 0 |
| 1.01E+08 | CX3CL1 | -2.01189 | 0 | 0 |
| 1.01E+08 | KIF21A | -2.19157 | 0 | 0 |
| 1.01E+08 | PRDM16 | 3.05136 | 3.36E-294 | 5.37E-294 |
| 1.01E+08 | MPP7 | -3.04803 | 0 | 0 |
| 1.01E+08 | RAPSN | -3.65919 | 6.44E-88 | 4.48E-88 |
| 1.01E+08 | TMPRSS7 | -8.30956 | 9.61E-256 | 1.38E-255 |
| 1.01E+08 | RASGEF1A | -2.20173 | 1.06E-173 | 1.14E-173 |
| 1.01E+08 | ADAM28 | 12.97442 | 0 | 0 |
| 1.01E+08 | LOC101120060 | -3.77349 | 7.09E-70 | 4.34E-70 |
| 1.01E+08 | LOC101120103 | -9.30839 | 3.22E-58 | 1.80E-58 |
| 1.01E+08 | FAM134B | 2.448403 | 0 | 0 |
| 1.01E+08 | SLC5A3 | 2.420062 | 0 | 0 |
| 1.01E+08 | LOC101120179 | 3.416014 | 0 | 0 |
| 1.01E+08 | S100A10 | -2.3574 | 0 | 0 |
| 1.01E+08 | TSHZ2 | 3.37652 | 0 | 0 |
| 1.01E+08 | FBXW12 | -4.07713 | 0 | 0 |
| 1.01E+08 | ADAMTS15 | -2.66206 | 0 | 0 |
| 1.01E+08 | CCDC68 | -5.26361 | 1.04E-81 | 6.96E-82 |
| 1.01E+08 | LCN10 | 13.40592 | 0 | 0 |
| 1.01E+08 | ELSPBP1 | 3.212518 | 0 | 0 |
| 1.01E+08 | MRPS6 | 2.340772 | 0 | 0 |
| 1.01E+08 | SPTB | -6.99982 | 0 | 0 |
| 1.01E+08 | WISP1 | 5.604024 | 0 | 0 |
| 1.01E+08 | WWOX | -2.28463 | 1.01E-198 | 1.21E-198 |
| 1.01E+08 | SLC43A1 | -2.25531 | 1.14E-292 | 1.82E-292 |
| 1.01E+08 | KREMEN1 | 2.978703 | 0 | 0 |
| 1.01E+08 | NAALAD2 | -2.08865 | 0 | 0 |
| 1.01E+08 | LOC101120455 | -2.87128 | 0 | 0 |
| 1.01E+08 | PRDM15 | -2.3855 | 2.35E-268 | 3.49E-268 |
| 1.01E+08 | STRA6 | 3.83644 | 0 | 0 |
| 1.01E+08 | SPINK8 | -7.09011 | 0 | 0 |
| 1.01E+08 | ANKRD22 | 5.10518 | 1.73E-131 | 1.55E-131 |
| 1.01E+08 | ZDHHC9 | 4.199096 | 0 | 0 |
| 1.01E+08 | EPHB6 | -2.85045 | 1.43E-114 | 1.17E-114 |
| 1.01E+08 | TMEM98 | 2.96935 | 0 | 0 |
| 1.01E+08 | SLC46A2 | -5.52853 | 0 | 0 |
| 1.01E+08 | MAPK10 | -4.40325 | 3.09E-149 | 2.99E-149 |
| 1.01E+08 | SLITRK4 | 5.028732 | 0 | 0 |
| 1.01E+08 | LOC101120732 | -3.14506 | 0 | 0 |
| 1.01E+08 | EZH2 | -2.36022 | 9.72E-277 | 1.48E-276 |
| 1.01E+08 | EPB41L2 | 2.953889 | 0 | 0 |
| 1.01E+08 | TLCD1 | -3.08751 | 1.55E-132 | 1.39E-132 |
| 1.01E+08 | AKR1B1 | -4.89482 | 0 | 0 |
| 1.01E+08 | ETNPPL | -6.79448 | 0 | 0 |
| 1.01E+08 | HSPA12A | -2.47581 | 0 | 0 |
| 1.01E+08 | ITGB5 | 3.465785 | 0 | 0 |
| 1.01E+08 | ADGRG1 | 4.820412 | 0 | 0 |
| 1.01E+08 | ZMYND10 | 2.110984 | 1.89E-300 | 3.08E-300 |
| 1.01E+08 | LOC101120969 | -6.20819 | 2.14E-258 | 3.10E-258 |
| 1.01E+08 | GPAM | 3.907322 | 0 | 0 |
| 1.01E+08 | RHOBTB1 | -2.17673 | 0 | 0 |
| 1.01E+08 | PTH2R | -5.07025 | 0 | 0 |
| 1.01E+08 | PKIB | -2.34405 | 3.22E-277 | 4.90E-277 |
| 1.01E+08 | MAP3K5 | 2.095464 | 1.10E-205 | 1.35E-205 |
| 1.01E+08 | PPT1 | -2.11176 | 0 | 0 |
| 1.01E+08 | LOC101121082 | -2.62528 | 8.11E-44 | 3.93E-44 |
| 1.01E+08 | MDGA2 | -3.06202 | 1.18E-81 | 7.87E-82 |
| 1.01E+08 | LOC101121159 | -5.20134 | 0 | 0 |
| 1.01E+08 | ALOX15B | -4.89589 | 0 | 0 |
| 1.01E+08 | TEDDM1 | 9.797406 | 0 | 0 |
| 1.01E+08 | C13H20orf27 | -2.21526 | 0 | 0 |
| 1.01E+08 | CABLES2 | -2.06233 | 0 | 0 |
| 1.01E+08 | ZMAT1 | -3.38698 | 0 | 0 |
| 1.01E+08 | GABRA2 | 4.955095 | 4.84E-125 | 4.19E-125 |
| 1.01E+08 | CDC45 | -2.05386 | 5.32E-53 | 2.83E-53 |
| 1.01E+08 | LRRC8B | 3.682392 | 0 | 0 |
| 1.01E+08 | LRRC3B | -4.21632 | 0 | 0 |
| 1.01E+08 | WNT5B | -2.54824 | 9.20E-80 | 6.05E-80 |
| 1.01E+08 | RIMS2 | -2.65062 | 4.88E-160 | 4.94E-160 |
| 1.01E+08 | NMNAT2 | -2.6921 | 0 | 0 |
| 1.01E+08 | LAMA5 | -2.51788 | 0 | 0 |
| 1.01E+08 | PIK3R1 | -2.65772 | 0 | 0 |
| 1.01E+08 | GREB1L | -3.71963 | 0 | 0 |
| 1.01E+08 | AMDHD1 | -2.12489 | 3.61E-85 | 2.46E-85 |
| 1.01E+08 | DHX58 | 2.256301 | 1.75E-157 | 1.75E-157 |
| 1.01E+08 | AK7 | 5.020206 | 0 | 0 |
| 1.01E+08 | NTRK3 | 3.617736 | 0 | 0 |
| 1.01E+08 | TMEM132A | -2.02345 | 1.31E-219 | 1.69E-219 |
| 1.01E+08 | LOC101121643 | -2.62648 | 2.56E-19 | 8.93E-20 |
| 1.01E+08 | PARD6G | -2.11606 | 4.39E-164 | 4.52E-164 |
| 1.01E+08 | TTC39B | -2.38798 | 0 | 0 |
| 1.01E+08 | PRPS2 | -5.57913 | 0 | 0 |
| 1.01E+08 | TMEM187 | -2.73783 | 1.16E-240 | 1.60E-240 |
| 1.01E+08 | DOK7 | -6.90318 | 1.15E-82 | 7.69E-83 |
| 1.01E+08 | SLC25A32 | -2.23712 | 0 | 0 |
| 1.01E+08 | SEMA3B | -2.43369 | 0 | 0 |
| 1.01E+08 | SLC22A20 | -10.379 | 2.15E-103 | 1.64E-103 |
| 1.01E+08 | PDE9A | 2.354346 | 3.11E-220 | 4.00E-220 |
| 1.01E+08 | LOC101121750 | -7.12439 | 8.76E-33 | 3.72E-33 |
| 1.01E+08 | MICAL2 | -2.57386 | 0 | 0 |
| 1.01E+08 | RPS6KA6 | -3.20894 | 0 | 0 |
| 1.01E+08 | FOXRED2 | -4.64422 | 0 | 0 |
| 1.01E+08 | RASGRF2 | -2.63834 | 1.63E-207 | 2.02E-207 |
| 1.01E+08 | ADCY8 | 4.790488 | 0 | 0 |
| 1.01E+08 | NT5C | 2.365172 | 8.56E-35 | 3.73E-35 |
| 1.01E+08 | TNNI3 | -2.68941 | 2.05E-229 | 2.73E-229 |
| 1.01E+08 | OLFML3 | -3.92471 | 0 | 0 |
| 1.01E+08 | NOSTRIN | -2.36599 | 2.57E-81 | 1.71E-81 |
| 1.01E+08 | C7H14orf37 | 2.220995 | 1.64E-121 | 1.39E-121 |
| 1.01E+08 | MRC2 | 4.064119 | 0 | 0 |
| 1.01E+08 | GPR153 | 3.416344 | 0 | 0 |
| 1.01E+08 | NANOS1 | -2.64115 | 1.15E-57 | 6.36E-58 |
| 1.01E+08 | ARHGDIG | 2.417975 | 1.57E-22 | 5.78E-23 |
| 1.01E+08 | ZNF365 | -2.57242 | 2.85E-224 | 3.73E-224 |
| 1.01E+08 | FZD6 | -2.04928 | 0 | 0 |
| 1.01E+08 | SEMA3F | -2.07198 | 0 | 0 |
| 1.01E+08 | SYT6 | -9.97592 | 0 | 0 |
| 1.01E+08 | APOBR | -2.25658 | 0 | 0 |
| 1.01E+08 | SLC25A40 | -2.3514 | 5.21E-127 | 4.55E-127 |
| 1.01E+08 | SLF2 | -2.16293 | 0 | 0 |
| 1.01E+08 | LOC101122329 | -2.97509 | 0 | 0 |
| 1.01E+08 | LOC101122398 | -2.81284 | 0 | 0 |
| 1.01E+08 | MUC20 | -3.98728 | 0 | 0 |
| 1.01E+08 | FBXO47 | -2.24347 | 8.59E-96 | 6.26E-96 |
| 1.01E+08 | IFNGR2 | 2.350033 | 0 | 0 |
| 1.01E+08 | SIK1 | 2.246713 | 1.14E-148 | 1.10E-148 |
| 1.01E+08 | OSGIN1 | -2.46271 | 9.80E-205 | 1.20E-204 |
| 1.01E+08 | LOC101122548 | -3.00915 | 3.52E-80 | 2.32E-80 |
| 1.01E+08 | MAGED1 | -2.36674 | 0 | 0 |
| 1.01E+08 | STRA8 | 5.509968 | 3.64E-132 | 3.27E-132 |
| 1.01E+08 | ST3GAL1 | 4.630751 | 0 | 0 |
| 1.01E+08 | NNAT | -2.71518 | 1.09E-242 | 1.51E-242 |
| 1.01E+08 | EPS8L1 | -2.03528 | 1.71E-191 | 1.98E-191 |
| 1.01E+08 | TWIST2 | -2.86409 | 2.84E-37 | 1.27E-37 |
| 1.01E+08 | LOC101122645 | -3.03693 | 1.33E-23 | 4.97E-24 |
| 1.01E+08 | RHOU | 2.043011 | 0 | 0 |
| 1.01E+08 | ABCD1 | -5.09724 | 0 | 0 |
| 1.01E+08 | PLXDC1 | -2.64017 | 2.63E-166 | 2.75E-166 |
| 1.01E+08 | FAM129A | -2.28281 | 0 | 0 |
| 1.01E+08 | CLPSL2 | -2.05693 | 0 | 0 |
| 1.01E+08 | SEMA4G | 2.348098 | 0 | 0 |
| 1.01E+08 | LIPI | 2.245827 | 6.41E-98 | 4.74E-98 |
| 1.01E+08 | ENPP3 | -3.30468 | 0 | 0 |
| 1.01E+08 | LOC101122803 | -5.84371 | 0 | 0 |
| 1.01E+08 | HPD | 5.609332 | 1.25E-141 | 1.17E-141 |
| 1.01E+08 | MRGPRF | -2.31832 | 2.78E-185 | 3.15E-185 |
| 1.01E+08 | ADAMTSL1 | -3.69604 | 0 | 0 |
| 1.01E+08 | NUDT11 | -6.58981 | 0 | 0 |
| 1.01E+08 | ATP11C | -2.42887 | 0 | 0 |
| 1.01E+08 | GRAMD1A | -2.23 | 0 | 0 |
| 1.01E+08 | DNASE1L3 | -5.95732 | 0 | 0 |
| 1.01E+08 | CITED4 | 2.311326 | 0 | 0 |
| 1.01E+08 | TMEM8A | 2.577133 | 6.50E-283 | 1.01E-282 |
| 1.01E+08 | CACNB1 | -3.86873 | 0 | 0 |
| 1.01E+08 | LTBP3 | 2.356437 | 0 | 0 |
| 1.01E+08 | TMEM159 | 2.255821 | 0 | 0 |
| 1.01E+08 | MED12 | -2.46478 | 0 | 0 |
| 1.01E+08 | STAC3 | -3.45578 | 2.08E-122 | 1.77E-122 |
| 1.01E+08 | ENPP1 | 4.650004 | 0 | 0 |
| 1.01E+08 | TMEM120B | -2.81766 | 0 | 0 |
| 1.01E+08 | LOC101123095 | -7.60271 | 0 | 0 |
| 1.01E+08 | LOC101123139 | 2.149951 | 1.22E-74 | 7.73E-75 |
| 1.01E+08 | ARAP1 | 2.152346 | 0 | 0 |
| 1.01E+08 | SEMA5A | 3.258166 | 0 | 0 |
| 1.01E+08 | TMEFF1 | -3.05485 | 6.59E-127 | 5.75E-127 |
| 1.01E+08 | A2ML1 | 2.542272 | 0 | 0 |
| 1.01E+08 | PIK3R3 | -4.60661 | 0 | 0 |
| 1.01E+08 | ABTB1 | -2.16264 | 0 | 0 |
| 1.01E+08 | SLC22A4 | -3.59977 | 0 | 0 |
| 1.01E+08 | DAPK2 | 2.225798 | 5.12E-112 | 4.11E-112 |
| 1.01E+08 | SLA | 3.156829 | 8.60E-155 | 8.51E-155 |
| 1.01E+08 | FLNB | -2.31276 | 0 | 0 |
| 1.01E+08 | SH3BGR | -2.54061 | 1.72E-175 | 1.87E-175 |
| 1.01E+08 | MED12L | -3.52668 | 0 | 0 |
| 1.01E+08 | SLC19A2 | -2.27479 | 0 | 0 |
| 1.01E+08 | IVL | -6.59042 | 0 | 0 |
| 1.01E+08 | AZGP1 | -2.7624 | 2.70E-299 | 4.38E-299 |
| 1.01E+08 | TMEM27 | -2.12892 | 0 | 0 |
| 1.01E+08 | ATP6V0A1 | -2.40615 | 0 | 0 |
| 1.01E+08 | KCNG1 | -2.10988 | 5.44E-117 | 4.47E-117 |
| 1.01E+08 | LOC101123536 | -10.3615 | 0 | 0 |
| 1.01E+08 | NLN | 2.414518 | 0 | 0 |
| 1.01E+08 | LOC101123612 | 4.345575 | 4.66E-238 | 6.38E-238 |
| 1.01E+08 | ETV1 | 3.028192 | 7.18E-156 | 7.14E-156 |
| 1.01E+08 | F5 | -3.08144 | 0 | 0 |
| 1.01E+08 | LOC101123651 | -3.60077 | 0 | 0 |
| 1.01E+08 | FHDC1 | -2.38485 | 2.83E-164 | 2.92E-164 |
| 1.06E+08 | LOC105601850 | -4.18882 | 2.00E-44 | 9.76E-45 |
| 1.06E+08 | LOC105601854 | -2.04589 | 3.08E-69 | 1.87E-69 |
| 1.06E+08 | GPR75 | 2.890352 | 3.76E-109 | 2.97E-109 |
| 1.06E+08 | LOC105602015 | -3.98221 | 0 | 0 |
| 1.06E+08 | C14H19orf68 | -2.96069 | 1.06E-130 | 9.44E-131 |
| 1.06E+08 | LOC105602432 | -3.23477 | 0 | 0 |
| 1.06E+08 | LOC105602979 | -2.69472 | 9.51E-103 | 7.22E-103 |
| 1.06E+08 | C18H14orf132 | -6.77839 | 1.19E-26 | 4.66E-27 |
| 1.06E+08 | CCDC85C | -3.24727 | 6.03E-297 | 9.69E-297 |
| 1.06E+08 | XRCC3 | -2.09412 | 1.20E-33 | 5.18E-34 |
| 1.06E+08 | ZFYVE21 | -2.21429 | 8.88E-49 | 4.54E-49 |
| 1.06E+08 | LOC105603599 | -2.62088 | 0 | 0 |
| 1.06E+08 | DEFB110 | 3.967466 | 0 | 0 |
| 1.06E+08 | MYLK4 | -4.43447 | 0 | 0 |
| 1.06E+08 | LOC105604082 | -5.08758 | 6.92E-231 | 9.26E-231 |
| 1.06E+08 | FLYWCH1 | -2.40628 | 1.79E-269 | 2.68E-269 |
| 1.06E+08 | LOC105604737 | -2.92204 | 9.75E-92 | 6.91E-92 |
| 1.06E+08 | LOC105604792 | 8.828008 | 0 | 0 |
| 1.06E+08 | LOC105604882 | -5.68326 | 0 | 0 |
| 1.06E+08 | ADIRF | -2.19414 | 1.63E-163 | 1.67E-163 |
| 1.06E+08 | LOC105605056 | -2.42792 | 7.61E-78 | 4.94E-78 |
| 1.06E+08 | LOC105605116 | -3.03822 | 0 | 0 |
| 1.06E+08 | LOC105605118 | -2.96997 | 2.49E-258 | 3.61E-258 |
| 1.06E+08 | LOC105605390 | -2.17443 | 2.25E-09 | 6.32E-10 |
| 1.06E+08 | CDR1 | -3.33705 | 2.09E-38 | 9.52E-39 |
| 1.06E+08 | NXT2 | -2.65889 | 0 | 0 |
| 1.06E+08 | MAMLD1 | -3.5927 | 0 | 0 |
| 1.06E+08 | LOC105605834 | -2.66144 | 0 | 0 |
| 1.06E+08 | LOC105605950 | -3.69416 | 3.25E-126 | 2.82E-126 |
| 1.06E+08 | LOC105606024 | -7.46319 | 7.43E-21 | 2.66E-21 |
| 1.06E+08 | LOC105606076 | 2.049763 | 2.27E-183 | 2.55E-183 |
| 1.06E+08 | LOC105606122 | -3.0024 | 2.07E-99 | 1.54E-99 |
| 1.06E+08 | LOC105606262 | -2.61515 | 0 | 0 |
| 1.06E+08 | LOC105606694 | -9.91908 | 0 | 0 |
| 1.06E+08 | LOC105606696 | -9.9991 | 0 | 0 |
| 1.06E+08 | LOC105606698 | 2.628794 | 0 | 0 |
| 1.06E+08 | SPINK13 | 3.048308 | 7.85E-290 | 1.24E-289 |
| 1.06E+08 | C2H9orf153 | 3.362289 | 0 | 0 |
| 1.06E+08 | RAB42 | 4.65656 | 0 | 0 |
| 1.06E+08 | DEFB134 | -6.42106 | 0 | 0 |
| 1.06E+08 | LOC105608603 | -5.01271 | 0 | 0 |
| 1.06E+08 | LOC105609102 | -2.71736 | 2.20E-53 | 1.17E-53 |
| 1.06E+08 | TGFBR3L | 2.858614 | 0 | 0 |
| 1.06E+08 | NPTXR | -2.32015 | 0 | 0 |
| 1.06E+08 | TUBB4A | 2.780617 | 0 | 0 |
| 1.06E+08 | LOC105609932 | 3.683551 | 3.73E-30 | 1.53E-30 |
| 1.06E+08 | LOC105610367 | -2.53796 | 2.74E-196 | 3.26E-196 |
| 1.06E+08 | NPW | 2.336531 | 9.99E-09 | 2.75E-09 |
| 1.06E+08 | LOC105611318 | 8.74972 | 0 | 0 |
| 1.06E+08 | LOC105611550 | 3.614898 | 0 | 0 |
| 1.06E+08 | LOC105611673 | -2.24832 | 0 | 0 |
| 1.06E+08 | LOC105612015 | -2.39699 | 3.66E-78 | 2.38E-78 |
| 1.06E+08 | TMEM210 | -2.72498 | 1.72E-10 | 4.99E-11 |
| 1.06E+08 | LOC105613233 | 13.52878 | 0 | 0 |
| 1.06E+08 | MEI4 | -4.1174 | 6.36E-268 | 9.46E-268 |
| 1.06E+08 | LOC105613399 | -3.79174 | 0 | 0 |
| 1.06E+08 | WFDC9 | -3.8741 | 0 | 0 |
| 1.06E+08 | LOC105613453 | -9.28931 | 1.29E-57 | 7.14E-58 |
| 1.06E+08 | PHLDA2 | -4.77714 | 3.55E-125 | 3.07E-125 |
| 1.06E+08 | LOC105613766 | -5.07082 | 0 | 0 |
| 1.06E+08 | RNASE9 | -5.75924 | 0 | 0 |
| 1.06E+08 | NSMF | 2.229443 | 1.54E-104 | 1.18E-104 |
| 1.06E+08 | LOC105616111 | -2.44191 | 3.63E-118 | 3.01E-118 |
| 1.06E+08 | LOC105616741 | -3.76634 | 3.53E-92 | 2.50E-92 |
| 1.06E+08 | LOC105616801 | 8.284168 | 0 | 0 |
| 1.06E+08 | LOC105616883 | -2.47639 | 0 | 0 |
| 1.07E+08 | LTB4R2 | 5.278561 | 1.22E-220 | 1.58E-220 |
| 1.07E+08 | LOC106990163 | -9.07731 | 0 | 0 |
| 1.07E+08 | LOC106990174 | -2.97915 | 0 | 0 |
| 1.07E+08 | CBY3 | -2.31531 | 4.80E-193 | 5.61E-193 |
| 1.07E+08 | SH3GL2 | -3.97124 | 0 | 0 |
| 1.07E+08 | LOC106990358 | -2.19299 | 3.45E-56 | 1.89E-56 |
| 1.07E+08 | SPINK14 | -12.0584 | 6.59E-247 | 9.20E-247 |
| 1.07E+08 | LOC106991088 | -5.57091 | 3.42E-68 | 2.07E-68 |
| 1.07E+08 | LOC106991447 | 6.208856 | 0 | 0 |
| 1.07E+08 | LOC106991561 | 8.016184 | 3.40E-29 | 1.38E-29 |
| 1.07E+08 | LOC106991619 | -2.39241 | 8.30E-25 | 3.17E-25 |
| 1.07E+08 | LOC106991702 | -2.18132 | 1.93E-81 | 1.29E-81 |
| 1.07E+08 | BDKRB1 | 3.323899 | 0 | 0 |
| 1.07E+08 | LOC106991842 | -2.87439 | 0 | 0 |
| 1.07E+08 | LOC106991857 | -2.62436 | 2.00E-78 | 1.31E-78 |
| BGI_novel_G000007 | BGI_novel_G000007 | -2.14541 | 2.76E-189 | 3.19E-189 |
| BGI_novel_G000015 | BGI_novel_G000015 | -2.81262 | 0 | 0 |
| BGI_novel_G000016 | BGI_novel_G000016 | 11.5518 | 1.78E-195 | 2.10E-195 |
| BGI_novel_G000030 | BGI_novel_G000030 | -6.38173 | 1.78E-188 | 2.05E-188 |
| BGI_novel_G000047 | BGI_novel_G000047 | 3.022922 | 0 | 0 |
| BGI_novel_G000053 | BGI_novel_G000053 | -6.50132 | 0 | 0 |
| BGI_novel_G000062 | BGI_novel_G000062 | -2.51039 | 2.38E-146 | 2.27E-146 |
| BGI_novel_G000070 | BGI_novel_G000070 | -3.0919 | 2.36E-67 | 1.42E-67 |
| BGI_novel_G000083 | BGI_novel_G000083 | -2.4458 | 2.54E-236 | 3.47E-236 |
| BGI_novel_G000084 | BGI_novel_G000084 | -2.58762 | 9.50E-113 | 7.66E-113 |
| BGI_novel_G000089 | BGI_novel_G000089 | -3.57489 | 4.34E-209 | 5.42E-209 |
| BGI_novel_G000097 | BGI_novel_G000097 | -3.61407 | 1.44E-232 | 1.94E-232 |
| BGI_novel_G000098 | BGI_novel_G000098 | -4.46673 | 0 | 0 |
| BGI_novel_G000119 | BGI_novel_G000119 | 8.179921 | 0 | 0 |
| BGI_novel_G000122 | BGI_novel_G000122 | -2.13591 | 4.16E-181 | 4.63E-181 |
| BGI_novel_G000131 | BGI_novel_G000131 | -2.87366 | 3.81E-210 | 4.77E-210 |
| BGI_novel_G000133 | BGI_novel_G000133 | -2.13431 | 3.07E-41 | 1.44E-41 |
| BGI_novel_G000135 | BGI_novel_G000135 | -2.04638 | 6.80E-170 | 7.21E-170 |
| BGI_novel_G000154 | BGI_novel_G000154 | -5.52963 | 1.04E-294 | 1.66E-294 |
| BGI_novel_G000175 | BGI_novel_G000175 | -3.67301 | 3.78E-56 | 2.07E-56 |
| BGI_novel_G000181 | BGI_novel_G000181 | -7.82483 | 1.76E-49 | 9.06E-50 |
| BGI_novel_G000204 | BGI_novel_G000204 | -2.83743 | 8.47E-103 | 6.43E-103 |
| BGI_novel_G000211 | BGI_novel_G000211 | -2.86005 | 2.07E-31 | 8.65E-32 |
| BGI_novel_G000217 | BGI_novel_G000217 | -3.04748 | 4.70E-229 | 6.24E-229 |
| BGI_novel_G000234 | BGI_novel_G000234 | 6.059778 | 0 | 0 |
| BGI_novel_G000240 | BGI_novel_G000240 | -5.99073 | 0 | 0 |
| BGI_novel_G000241 | BGI_novel_G000241 | -4.90612 | 0 | 0 |
| BGI_novel_G000242 | BGI_novel_G000242 | -6.68599 | 0 | 0 |
| BGI_novel_G000269 | BGI_novel_G000269 | 4.808647 | 0 | 0 |
| BGI_novel_G000270 | BGI_novel_G000270 | 4.488554 | 0 | 0 |
| BGI_novel_G000273 | BGI_novel_G000273 | 8.285267 | 0 | 0 |
| BGI_novel_G000280 | BGI_novel_G000280 | -3.66387 | 1.06E-112 | 8.56E-113 |
| BGI_novel_G000281 | BGI_novel_G000281 | -3.31069 | 2.25E-30 | 9.25E-31 |
| BGI_novel_G000283 | BGI_novel_G000283 | -5.15681 | 0 | 0 |
| BGI_novel_G000300 | BGI_novel_G000300 | -2.61206 | 2.36E-173 | 2.53E-173 |
| BGI_novel_G000316 | BGI_novel_G000316 | -2.1459 | 1.02E-125 | 8.80E-126 |
| BGI_novel_G000318 | BGI_novel_G000318 | -3.26582 | 9.17E-120 | 7.68E-120 |
| BGI_novel_G000321 | BGI_novel_G000321 | -10.3165 | 4.66E-198 | 5.56E-198 |
| BGI_novel_G000324 | BGI_novel_G000324 | 2.967639 | 0 | 0 |
| BGI_novel_G000330 | BGI_novel_G000330 | -2.34576 | 3.72E-283 | 5.80E-283 |
| BGI_novel_G000332 | BGI_novel_G000332 | -2.51218 | 2.14E-48 | 1.09E-48 |
| BGI_novel_G000334 | BGI_novel_G000334 | -3.1033 | 3.80E-114 | 3.08E-114 |
| BGI_novel_G000339 | BGI_novel_G000339 | 14.09213 | 0 | 0 |
| BGI_novel_G000340 | BGI_novel_G000340 | 11.20906 | 0 | 0 |
| BGI_novel_G000342 | BGI_novel_G000342 | 11.91926 | 6.16E-236 | 8.39E-236 |
| BGI_novel_G000343 | BGI_novel_G000343 | 9.548388 | 0 | 0 |
| BGI_novel_G000344 | BGI_novel_G000344 | 12.51111 | 0 | 0 |
| BGI_novel_G000345 | BGI_novel_G000345 | 11.7416 | 1.81E-215 | 2.31E-215 |
| BGI_novel_G000346 | BGI_novel_G000346 | 11.30776 | 2.31E-172 | 2.48E-172 |
| BGI_novel_G000347 | BGI_novel_G000347 | 10.87613 | 0 | 0 |
| BGI_novel_G000357 | BGI_novel_G000357 | 12.26084 | 1.28E-280 | 1.98E-280 |
| BGI_novel_G000360 | BGI_novel_G000360 | 12.81132 | 0 | 0 |
| BGI_novel_G000362 | BGI_novel_G000362 | 13.3049 | 0 | 0 |
| BGI_novel_G000363 | BGI_novel_G000363 | 13.57868 | 0 | 0 |
| BGI_novel_G000367 | BGI_novel_G000367 | -4.99855 | 1.04E-203 | 1.26E-203 |
| BGI_novel_G000368 | BGI_novel_G000368 | -2.86607 | 3.74E-175 | 4.04E-175 |
| BGI_novel_G000375 | BGI_novel_G000375 | -3.13186 | 6.45E-262 | 9.48E-262 |
| BGI_novel_G000385 | BGI_novel_G000385 | -2.12149 | 4.09E-179 | 4.50E-179 |
| BGI_novel_G000392 | BGI_novel_G000392 | -2.6209 | 4.46E-304 | 7.33E-304 |
| BGI_novel_G000395 | BGI_novel_G000395 | 6.946344 | 0 | 0 |
| BGI_novel_G000396 | BGI_novel_G000396 | 4.941314 | 1.28E-305 | 2.10E-305 |
| BGI_novel_G000407 | BGI_novel_G000407 | -2.85534 | 0 | 0 |
| BGI_novel_G000420 | BGI_novel_G000420 | -4.32113 | 9.95E-34 | 4.29E-34 |
| BGI_novel_G000442 | BGI_novel_G000442 | -3.58412 | 2.99E-121 | 2.52E-121 |
| BGI_novel_G000444 | BGI_novel_G000444 | -9.07759 | 8.32E-184 | 9.37E-184 |
| BGI_novel_G000445 | BGI_novel_G000445 | -5.98766 | 0 | 0 |
| BGI_novel_G000452 | BGI_novel_G000452 | -4.37709 | 2.53E-180 | 2.81E-180 |
| BGI_novel_G000481 | BGI_novel_G000481 | -2.15889 | 3.61E-48 | 1.83E-48 |
| BGI_novel_G000490 | BGI_novel_G000490 | -2.28011 | 5.86E-117 | 4.81E-117 |
| BGI_novel_G000496 | BGI_novel_G000496 | 5.007161 | 6.56E-206 | 8.08E-206 |
| BGI_novel_G000500 | BGI_novel_G000500 | -5.75917 | 3.94E-304 | 6.48E-304 |
| BGI_novel_G000517 | BGI_novel_G000517 | -2.78502 | 0 | 0 |
| BGI_novel_G000522 | BGI_novel_G000522 | -2.52402 | 9.96E-65 | 5.85E-65 |
| BGI_novel_G000547 | BGI_novel_G000547 | -2.92217 | 0 | 0 |
| BGI_novel_G000573 | BGI_novel_G000573 | -4.969 | 2.60E-89 | 1.82E-89 |
| BGI_novel_G000575 | BGI_novel_G000575 | -3.63186 | 1.86E-277 | 2.83E-277 |
| BGI_novel_G000576 | BGI_novel_G000576 | -2.6773 | 0 | 0 |
| BGI_novel_G000578 | BGI_novel_G000578 | -3.38897 | 0 | 0 |
| BGI_novel_G000590 | BGI_novel_G000590 | -2.02872 | 5.65E-27 | 2.23E-27 |
| BGI_novel_G000594 | BGI_novel_G000594 | -4.75367 | 7.68E-214 | 9.71E-214 |
| BGI_novel_G000602 | BGI_novel_G000602 | -4.77936 | 9.91E-33 | 4.20E-33 |
| BGI_novel_G000616 | BGI_novel_G000616 | -3.65654 | 1.42E-119 | 1.19E-119 |
| BGI_novel_G000618 | BGI_novel_G000618 | -2.12534 | 5.99E-21 | 2.15E-21 |
| BGI_novel_G000634 | BGI_novel_G000634 | -3.79737 | 6.09E-55 | 3.30E-55 |
| BGI_novel_G000651 | BGI_novel_G000651 | 2.874977 | 2.30E-121 | 1.94E-121 |
| BGI_novel_G000655 | BGI_novel_G000655 | -2.04919 | 2.46E-92 | 1.75E-92 |
| BGI_novel_G000657 | BGI_novel_G000657 | 5.626038 | 3.41E-37 | 1.53E-37 |
| BGI_novel_G000662 | BGI_novel_G000662 | 7.981544 | 3.59E-228 | 4.76E-228 |
| BGI_novel_G000663 | BGI_novel_G000663 | 4.938641 | 5.37E-180 | 5.94E-180 |
| BGI_novel_G000665 | BGI_novel_G000665 | 11.67477 | 3.24E-208 | 4.03E-208 |
| BGI_novel_G000666 | BGI_novel_G000666 | 11.01039 | 1.04E-147 | 1.00E-147 |
| BGI_novel_G000667 | BGI_novel_G000667 | 7.040898 | 6.54E-273 | 9.85E-273 |
| BGI_novel_G000669 | BGI_novel_G000669 | 6.427474 | 0 | 0 |
| BGI_novel_G000670 | BGI_novel_G000670 | 8.049559 | 0 | 0 |
| BGI_novel_G000684 | BGI_novel_G000684 | -3.54663 | 0 | 0 |
| BGI_novel_G000713 | BGI_novel_G000713 | -5.58873 | 9.29E-166 | 9.65E-166 |
| BGI_novel_G000718 | BGI_novel_G000718 | -3.04244 | 8.98E-205 | 1.10E-204 |
| BGI_novel_G000763 | BGI_novel_G000763 | -3.94407 | 1.97E-87 | 1.37E-87 |
| BGI_novel_G000774 | BGI_novel_G000774 | -7.70437 | 0 | 0 |
| BGI_novel_G000776 | BGI_novel_G000776 | -5.10864 | 4.63E-196 | 5.49E-196 |
| BGI_novel_G000786 | BGI_novel_G000786 | -2.08985 | 4.64E-177 | 5.06E-177 |
| BGI_novel_G000802 | BGI_novel_G000802 | 2.701345 | 0 | 0 |
| BGI_novel_G000821 | BGI_novel_G000821 | 5.698046 | 0 | 0 |
| BGI_novel_G000822 | BGI_novel_G000822 | 5.978015 | 0 | 0 |
| BGI_novel_G000823 | BGI_novel_G000823 | 5.80265 | 0 | 0 |
| BGI_novel_G000824 | BGI_novel_G000824 | 6.320085 | 4.93E-193 | 5.77E-193 |
| BGI_novel_G000825 | BGI_novel_G000825 | 5.750614 | 0 | 0 |
| BGI_novel_G000826 | BGI_novel_G000826 | 4.252982 | 1.65E-218 | 2.12E-218 |
| BGI_novel_G000827 | BGI_novel_G000827 | -2.85071 | 4.94E-74 | 3.11E-74 |
| BGI_novel_G000831 | BGI_novel_G000831 | -2.01877 | 6.32E-57 | 3.49E-57 |
| BGI_novel_G000832 | BGI_novel_G000832 | -3.16439 | 0 | 0 |
| BGI_novel_G000838 | BGI_novel_G000838 | 5.613573 | 1.03E-153 | 1.01E-153 |
| BGI_novel_G000864 | BGI_novel_G000864 | -2.07281 | 1.03E-130 | 9.21E-131 |
| BGI_novel_G000866 | BGI_novel_G000866 | -2.56054 | 6.74E-148 | 6.47E-148 |
| BGI_novel_G000867 | BGI_novel_G000867 | -9.81162 | 0 | 0 |
| BGI_novel_G000868 | BGI_novel_G000868 | -8.21614 | 7.08E-32 | 2.97E-32 |
| BGI_novel_G000890 | BGI_novel_G000890 | -2.29948 | 6.93E-180 | 7.66E-180 |
| BGI_novel_G000894 | BGI_novel_G000894 | -3.30226 | 1.85E-67 | 1.11E-67 |
| BGI_novel_G000898 | BGI_novel_G000898 | -2.36483 | 6.06E-202 | 7.35E-202 |
| BGI_novel_G000915 | BGI_novel_G000915 | -4.05556 | 4.96E-301 | 8.09E-301 |
| BGI_novel_G000927 | BGI_novel_G000927 | -2.79592 | 0 | 0 |
| BGI_novel_G000930 | BGI_novel_G000930 | -4.20213 | 2.65E-201 | 3.20E-201 |
| BGI_novel_G000931 | BGI_novel_G000931 | -5.49498 | 2.29E-201 | 2.77E-201 |
| BGI_novel_G000936 | BGI_novel_G000936 | -2.1548 | 2.82E-69 | 1.71E-69 |
| BGI_novel_G000943 | BGI_novel_G000943 | -4.06908 | 2.42E-181 | 2.70E-181 |
| BGI_novel_G000971 | BGI_novel_G000971 | -8.10302 | 2.48E-114 | 2.01E-114 |
| BGI_novel_G000983 | BGI_novel_G000983 | 8.826912 | 3.11E-174 | 3.35E-174 |
| BGI_novel_G000984 | BGI_novel_G000984 | 6.668411 | 0 | 0 |
| BGI_novel_G000985 | BGI_novel_G000985 | 3.278532 | 2.68E-214 | 3.40E-214 |
| BGI_novel_G000987 | BGI_novel_G000987 | 7.751945 | 0 | 0 |
| BGI_novel_G000988 | BGI_novel_G000988 | 4.585753 | 0 | 0 |
| BGI_novel_G000999 | BGI_novel_G000999 | -5.56049 | 1.05E-67 | 6.31E-68 |
| BGI_novel_G001001 | BGI_novel_G001001 | -2.49965 | 0 | 0 |
| BGI_novel_G001002 | BGI_novel_G001002 | 3.170175 | 0 | 0 |
| BGI_novel_G001005 | BGI_novel_G001005 | -2.04994 | 1.91E-223 | 2.50E-223 |
| BGI_novel_G001008 | BGI_novel_G001008 | -2.4826 | 9.89E-110 | 7.85E-110 |
| BGI_novel_G001009 | BGI_novel_G001009 | -2.34933 | 1.88E-161 | 1.91E-161 |
| BGI_novel_G001012 | BGI_novel_G001012 | 3.40311 | 3.25E-76 | 2.08E-76 |
| BGI_novel_G001014 | BGI_novel_G001014 | 7.844228 | 0 | 0 |
| BGI_novel_G001023 | BGI_novel_G001023 | -2.17851 | 1.53E-140 | 1.42E-140 |
| BGI_novel_G001031 | BGI_novel_G001031 | 4.883639 | 6.27E-50 | 3.24E-50 |
| BGI_novel_G001040 | BGI_novel_G001040 | -2.81194 | 1.55E-216 | 1.98E-216 |
| BGI_novel_G001044 | BGI_novel_G001044 | 4.72998 | 0 | 0 |
| BGI_novel_G001050 | BGI_novel_G001050 | -3.87778 | 9.15E-255 | 1.31E-254 |
| BGI_novel_G001060 | BGI_novel_G001060 | 12.4422 | 0 | 0 |
| BGI_novel_G001061 | BGI_novel_G001061 | 14.11521 | 0 | 0 |
| BGI_novel_G001063 | BGI_novel_G001063 | -2.53886 | 4.82E-74 | 3.04E-74 |
| BGI_novel_G001069 | BGI_novel_G001069 | -9.41909 | 1.83E-241 | 2.51E-241 |
| BGI_novel_G001070 | BGI_novel_G001070 | -9.88063 | 2.71E-79 | 1.78E-79 |
| BGI_novel_G001075 | BGI_novel_G001075 | -3.13768 | 9.58E-299 | 1.55E-298 |
| BGI_novel_G001080 | BGI_novel_G001080 | -5.72445 | 9.27E-39 | 4.23E-39 |
| BGI_novel_G001081 | BGI_novel_G001081 | -4.93663 | 8.17E-138 | 7.51E-138 |
| BGI_novel_G001088 | BGI_novel_G001088 | -4.43453 | 2.50E-269 | 3.74E-269 |
| BGI_novel_G001092 | BGI_novel_G001092 | -5.93908 | 2.89E-243 | 4.00E-243 |
| BGI_novel_G001095 | BGI_novel_G001095 | -4.16577 | 1.18E-283 | 1.85E-283 |
| BGI_novel_G001104 | BGI_novel_G001104 | 12.34796 | 0 | 0 |
| BGI_novel_G001110 | BGI_novel_G001110 | 4.005164 | 3.20E-215 | 4.07E-215 |
| BGI_novel_G001112 | BGI_novel_G001112 | -3.17695 | 2.51E-110 | 2.00E-110 |
| BGI_novel_G001116 | BGI_novel_G001116 | -5.21267 | 1.83E-197 | 2.18E-197 |
| BGI_novel_G001117 | BGI_novel_G001117 | -2.69834 | 0 | 0 |
| BGI_novel_G001119 | BGI_novel_G001119 | -8.1462 | 0 | 0 |
| BGI_novel_G001121 | BGI_novel_G001121 | 3.540502 | 4.34E-38 | 1.97E-38 |
| BGI_novel_G001126 | BGI_novel_G001126 | 4.285172 | 8.76E-111 | 6.99E-111 |
| BGI_novel_G001139 | BGI_novel_G001139 | 6.486635 | 3.20E-65 | 1.89E-65 |
| BGI_novel_G001147 | BGI_novel_G001147 | -4.3112 | 6.20E-237 | 8.47E-237 |
| BGI_novel_G001149 | BGI_novel_G001149 | -12.4285 | 9.55E-298 | 1.54E-297 |
| BGI_novel_G001150 | BGI_novel_G001150 | -5.2839 | 3.93E-192 | 4.58E-192 |
| BGI_novel_G001151 | BGI_novel_G001151 | -10.7206 | 8.79E-124 | 7.54E-124 |
| BGI_novel_G001152 | BGI_novel_G001152 | -10.5398 | 1.47E-112 | 1.18E-112 |
| BGI_novel_G001153 | BGI_novel_G001153 | -2.78293 | 5.30E-282 | 8.22E-282 |
| BGI_novel_G001162 | BGI_novel_G001162 | -2.40079 | 1.70E-22 | 6.25E-23 |
| BGI_novel_G001173 | BGI_novel_G001173 | -7.797 | 0 | 0 |
| BGI_novel_G001175 | BGI_novel_G001175 | -3.41621 | 1.06E-259 | 1.54E-259 |
| BGI_novel_G001200 | BGI_novel_G001200 | -2.24115 | 9.32E-105 | 7.16E-105 |
| BGI_novel_G001203 | BGI_novel_G001203 | -9.71817 | 0 | 0 |
| BGI_novel_G001206 | BGI_novel_G001206 | -9.48942 | 3.16E-64 | 1.85E-64 |
| BGI_novel_G001207 | BGI_novel_G001207 | -4.39433 | 0 | 0 |
| BGI_novel_G001223 | BGI_novel_G001223 | -7.55005 | 2.67E-174 | 2.88E-174 |
| BGI_novel_G001224 | BGI_novel_G001224 | -6.51758 | 1.61E-103 | 1.23E-103 |
| BGI_novel_G001228 | BGI_novel_G001228 | -5.00179 | 0 | 0 |
| BGI_novel_G001259 | BGI_novel_G001259 | -2.77539 | 0 | 0 |
| BGI_novel_G001264 | BGI_novel_G001264 | -6.26986 | 5.20E-225 | 6.83E-225 |
| BGI_novel_G001284 | BGI_novel_G001284 | 13.55306 | 0 | 0 |
| BGI_novel_G001297 | BGI_novel_G001297 | -4.28991 | 6.11E-65 | 3.60E-65 |
| BGI_novel_G001298 | BGI_novel_G001298 | -6.94376 | 3.89E-57 | 2.15E-57 |
| BGI_novel_G001303 | BGI_novel_G001303 | -2.1498 | 1.03E-228 | 1.37E-228 |
| BGI_novel_G001307 | BGI_novel_G001307 | -8.91418 | 6.23E-47 | 3.12E-47 |
| BGI_novel_G001308 | BGI_novel_G001308 | -9.3474 | 4.72E-232 | 6.35E-232 |
| BGI_novel_G001310 | BGI_novel_G001310 | -2.96843 | 7.94E-81 | 5.27E-81 |
| BGI_novel_G001319 | BGI_novel_G001319 | -4.43266 | 0 | 0 |
| BGI_novel_G001341 | BGI_novel_G001341 | -3.04968 | 3.42E-77 | 2.21E-77 |
| BGI_novel_G001344 | BGI_novel_G001344 | -2.233 | 0 | 0 |
| BGI_novel_G001347 | BGI_novel_G001347 | -2.6362 | 2.13E-57 | 1.18E-57 |
| BGI_novel_G001367 | BGI_novel_G001367 | -2.95039 | 3.94E-93 | 2.82E-93 |
| BGI_novel_G001375 | BGI_novel_G001375 | -6.24506 | 4.07E-199 | 4.88E-199 |
| BGI_novel_G001380 | BGI_novel_G001380 | 2.574036 | 0 | 0 |
| BGI_novel_G001381 | BGI_novel_G001381 | 9.890717 | 0 | 0 |
| BGI_novel_G001382 | BGI_novel_G001382 | 7.200192 | 0 | 0 |
| BGI_novel_G001388 | BGI_novel_G001388 | -5.05525 | 3.04E-169 | 3.21E-169 |
| BGI_novel_G001394 | BGI_novel_G001394 | -2.17195 | 4.88E-66 | 2.89E-66 |
| BGI_novel_G001397 | BGI_novel_G001397 | -2.59452 | 7.52E-90 | 5.29E-90 |
| BGI_novel_G001402 | BGI_novel_G001402 | -3.68149 | 4.39E-77 | 2.83E-77 |
| BGI_novel_G001418 | BGI_novel_G001418 | -2.93313 | 7.31E-96 | 5.33E-96 |
| BGI_novel_G001442 | BGI_novel_G001442 | -2.4105 | 4.80E-82 | 3.21E-82 |
| BGI_novel_G001451 | BGI_novel_G001451 | -2.1791 | 6.58E-11 | 1.93E-11 |
| BGI_novel_G001455 | BGI_novel_G001455 | -3.90677 | 1.75E-218 | 2.24E-218 |
| BGI_novel_G001488 | BGI_novel_G001488 | -5.55081 | 1.76E-143 | 1.65E-143 |
| BGI_novel_G001490 | BGI_novel_G001490 | -4.97382 | 0 | 0 |
| BGI_novel_G001515 | BGI_novel_G001515 | -5.5364 | 2.94E-103 | 2.24E-103 |
| BGI_novel_G001517 | BGI_novel_G001517 | -2.51834 | 1.84E-94 | 1.33E-94 |
| BGI_novel_G001533 | BGI_novel_G001533 | 5.595929 | 0 | 0 |
| BGI_novel_G001534 | BGI_novel_G001534 | 6.472771 | 0 | 0 |
| BGI_novel_G001541 | BGI_novel_G001541 | 3.855999 | 0 | 0 |
| BGI_novel_G001555 | BGI_novel_G001555 | -4.12679 | 0 | 0 |
| BGI_novel_G001556 | BGI_novel_G001556 | -4.25848 | 0 | 0 |
| BGI_novel_G001559 | BGI_novel_G001559 | -2.25015 | 0 | 0 |
| BGI_novel_G001560 | BGI_novel_G001560 | 11.64412 | 0 | 0 |
| BGI_novel_G001561 | BGI_novel_G001561 | 9.935815 | 0 | 0 |
| BGI_novel_G001562 | BGI_novel_G001562 | 12.35281 | 0 | 0 |
| BGI_novel_G001563 | BGI_novel_G001563 | -2.22774 | 1.77E-303 | 2.91E-303 |
| BGI_novel_G001566 | BGI_novel_G001566 | -3.55275 | 7.78E-165 | 8.04E-165 |
| BGI_novel_G001569 | BGI_novel_G001569 | -2.06077 | 1.18E-97 | 8.70E-98 |
| BGI_novel_G001579 | BGI_novel_G001579 | 4.212851 | 3.08E-156 | 3.07E-156 |
| BGI_novel_G001585 | BGI_novel_G001585 | -2.59827 | 0 | 0 |
| BGI_novel_G001586 | BGI_novel_G001586 | -3.93861 | 5.99E-118 | 4.95E-118 |
| BGI_novel_G001592 | BGI_novel_G001592 | -2.45824 | 2.65E-215 | 3.38E-215 |
| BGI_novel_G001600 | BGI_novel_G001600 | -2.13611 | 0 | 0 |
| BGI_novel_G001610 | BGI_novel_G001610 | -7.81062 | 7.30E-285 | 1.14E-284 |
| BGI_novel_G001620 | BGI_novel_G001620 | -2.46182 | 3.21E-95 | 2.33E-95 |
| BGI_novel_G001627 | BGI_novel_G001627 | -2.05579 | 1.55E-69 | 9.42E-70 |
| BGI_novel_G001633 | BGI_novel_G001633 | -2.05007 | 3.58E-56 | 1.96E-56 |
| BGI_novel_G001637 | BGI_novel_G001637 | -5.76858 | 2.41E-117 | 1.99E-117 |
| BGI_novel_G001638 | BGI_novel_G001638 | -4.42861 | 4.59E-94 | 3.31E-94 |
| BGI_novel_G001640 | BGI_novel_G001640 | -8.25886 | 0 | 0 |
| BGI_novel_G001641 | BGI_novel_G001641 | -5.37982 | 3.94E-214 | 5.00E-214 |
| BGI_novel_G001642 | BGI_novel_G001642 | -6.38351 | 0 | 0 |
| BGI_novel_G001648 | BGI_novel_G001648 | -3.95856 | 1.05E-160 | 1.06E-160 |
| BGI_novel_G001650 | BGI_novel_G001650 | -2.44925 | 4.79E-83 | 3.22E-83 |
| BGI_novel_G001653 | BGI_novel_G001653 | 3.606037 | 0 | 0 |
| BGI_novel_G001656 | BGI_novel_G001656 | 2.049505 | 6.31E-131 | 5.63E-131 |
| BGI_novel_G001657 | BGI_novel_G001657 | 2.115004 | 4.37E-110 | 3.48E-110 |
| BGI_novel_G001668 | BGI_novel_G001668 | -2.54034 | 0 | 0 |
| BGI_novel_G001671 | BGI_novel_G001671 | -3.1689 | 0 | 0 |
| BGI_novel_G001684 | BGI_novel_G001684 | -5.95732 | 0 | 0 |
| BGI_novel_G001685 | BGI_novel_G001685 | -11.3036 | 1.08E-167 | 1.13E-167 |
| BGI_novel_G001686 | BGI_novel_G001686 | -7.76059 | 0 | 0 |
| BGI_novel_G001701 | BGI_novel_G001701 | -3.35962 | 1.39E-226 | 1.84E-226 |
| BGI_novel_G001717 | BGI_novel_G001717 | -3.89158 | 1.93E-287 | 3.04E-287 |
| BGI_novel_G001722 | BGI_novel_G001722 | -4.34103 | 4.59E-124 | 3.95E-124 |
| BGI_novel_G001723 | BGI_novel_G001723 | -3.97477 | 2.09E-306 | 3.46E-306 |
| BGI_novel_G001734 | BGI_novel_G001734 | -2.92276 | 9.50E-30 | 3.89E-30 |
| BGI_novel_G001760 | BGI_novel_G001760 | -5.18996 | 3.28E-109 | 2.59E-109 |
| BGI_novel_G001763 | BGI_novel_G001763 | 5.676626 | 4.44E-239 | 6.08E-239 |
| BGI_novel_G001767 | BGI_novel_G001767 | 6.032133 | 3.00E-189 | 3.46E-189 |
| BGI_novel_G001768 | BGI_novel_G001768 | 10.24047 | 0 | 0 |
| BGI_novel_G001771 | BGI_novel_G001771 | 5.490045 | 0 | 0 |
| BGI_novel_G001782 | BGI_novel_G001782 | -3.14861 | 3.30E-250 | 4.66E-250 |
| BGI_novel_G001783 | BGI_novel_G001783 | -4.6358 | 0 | 0 |
| BGI_novel_G001809 | BGI_novel_G001809 | -3.61278 | 0 | 0 |
| BGI_novel_G001817 | BGI_novel_G001817 | -2.97779 | 0 | 0 |
| BGI_novel_G001821 | BGI_novel_G001821 | -3.73913 | 1.71E-278 | 2.62E-278 |
| BGI_novel_G001834 | BGI_novel_G001834 | -2.7181 | 3.42E-30 | 1.40E-30 |
| BGI_novel_G001837 | BGI_novel_G001837 | -5.61257 | 0 | 0 |
| BGI_novel_G001843 | BGI_novel_G001843 | -2.3169 | 2.65E-43 | 1.27E-43 |
| BGI_novel_G001869 | BGI_novel_G001869 | 2.125095 | 0 | 0 |
| BGI_novel_G001870 | BGI_novel_G001870 | -2.2472 | 3.93E-70 | 2.41E-70 |
| BGI_novel_G001874 | BGI_novel_G001874 | -2.75939 | 7.12E-12 | 2.13E-12 |
| BGI_novel_G001877 | BGI_novel_G001877 | -5.21231 | 9.81E-190 | 1.13E-189 |
| BGI_novel_G001884 | BGI_novel_G001884 | -2.26551 | 1.05E-180 | 1.17E-180 |
| BGI_novel_G001889 | BGI_novel_G001889 | 6.088848 | 1.73E-50 | 9.01E-51 |
| BGI_novel_G001916 | BGI_novel_G001916 | -4.37585 | 0 | 0 |
| BGI_novel_G001917 | BGI_novel_G001917 | -4.3273 | 6.31E-93 | 4.51E-93 |
| BGI_novel_G001920 | BGI_novel_G001920 | -5.96591 | 3.17E-27 | 1.25E-27 |
| BGI_novel_G001935 | BGI_novel_G001935 | -3.84428 | 2.54E-07 | 6.69E-08 |
| BGI_novel_G001971 | BGI_novel_G001971 | 2.422505 | 2.70E-07 | 7.10E-08 |
| BGI_novel_G001989 | BGI_novel_G001989 | 2.002601 | 2.75E-05 | 6.70E-06 |
| BGI_novel_G002030 | BGI_novel_G002030 | -9.11729 | 4.22E-204 | 5.16E-204 |
| BGI_novel_G002039 | BGI_novel_G002039 | -3.28494 | 1.40E-97 | 1.03E-97 |
| BGI_novel_G002040 | BGI_novel_G002040 | -8.29874 | 2.22E-33 | 9.51E-34 |
| BGI_novel_G002062 | BGI_novel_G002062 | -2.76844 | 1.22E-56 | 6.72E-57 |
| BGI_novel_G002085 | BGI_novel_G002085 | 10.74942 | 7.12E-129 | 6.28E-129 |
| BGI_novel_G002086 | BGI_novel_G002086 | 10.74942 | 7.12E-129 | 6.28E-129 |
