# Supplementary material for: Study on the region-specific expression of epididymis mRNA in the rams
Source: PLoS One. 2021 Jan 25;16(1):e0245933. doi: 10.1371/journal.pone.0245933 (PMC7833257; doi:10.1371/journal.pone.0245933)
Supplement: S9 Table — (DOCX) [file pone.0245933.s013.docx]

# S9 Table. The SEGs list in different regions of epididymis

| **Regions** | **Number of genes** | **Gene name** |
| --- | --- | --- |
| caput | 129 | HEY2, IZUMO1, ODF3B, HIVEP3, MFSD6L, PLCL1, QPRT, RTN1, LCNL1, TEKT1, C1H2orf54, PREX2, SNX31, S1PR3, INF2, EPHA6, FUT4, LY6G5C, CST11, RAP1GAP, KIAA0513, SLCO4C1, GCNT4, LOC101104518, ATP6V1B1, HS3ST3A1, SHB, LOC105613233, HSD3B7, MIXL1, LCN8, SPRY4, LCN6, FRMPD1, SLC16A10, BTG4, TREM1, CLRN3, VTCN1, PPP1R32, FUT1, TENM3, SLC9A2, LOC101108901, PIPOX, WIF1, DECR2, CACNG4, CYP2E1, LOC101111006, WNT11, ADAMTSL2, CDH17, SLC15A2, OVCH2, STEAP1, SLC22A16, PDE4B, TMEM37, STEAP2, SMPX, RAPGEF4, FAM78B, LTB4R, LRRC36, THEG, KIF5C, SPATA18, DCLK3, TMC8, EPHB1, CELF4, LOC101114861, CHGA, WDR86, RNF144A, LOC101115343, RUNX2, SIDT1, GPRIN2, CHRNA9, RHBDL3, DUSP2, ZNF536, ACTN3, SLC13A2, XYLB, NCMAP, LIPH, DOC2A, LOC101116795, LOC101116841, SLC34A1, VWDE, CCDC178, LCN10, PRSS27, ROPN1, LBX2, STEAP3, SHANK1, GNA15, CLEC11A, COL22A1, PRDM16, LOC101120179, C13H20orf85, GABRA2, AK7, ADCY8, ARHGDIG, SIK1, STRA8, HPD, LOC101123612, GPR75, LOC105604792, RAB42, NPW, TSPAN19, LOC105616801, LTB4R2 |
| corpus | 54 | LOC106991069, PAX4, MGAM, SLC6A15, LOC106991631, VGLL2, TVP23A, PRSS16, FAM19A5, GJD2, ABCC12, LOC101118164, GALR2, SLC1A3, LOC101115740, ZACN, GNAL, LOC105615088, LOC101110195, ITPKA, CADPS, TMEM89, PIWIL4, LOC105613768, LOC101113761, LOC101111669, KISS1, LOC106991896, LOC101111915, LOC106991725, LOC101116157, DLGAP1, KIF5A, LOC101119530, CLDN16, PROKR1, WFDC8, PLA2G2D, CCBE1, LOC105604152, LOC101109397, SDK1, ARL9, PRRG3, RGL3, LOC101114311, CREB5, MFSD4, LOC101113331, STC2, GDF3, DUSP8, LOC101112606, LOC105615953 |
| cauda | 99 | LOC101123536, FABP6, SLC5A1, SERPINA1, SPINK14, MGAT4C, LOC101118216, LOC101122803, RLBP1, LOC105606696, ALOX15B, SYT6, EXTL1, C20H6orf136, SELP, LOC101116570, ADAMTSL5, PTH2R, LOC101104222, NPPC, HOXA10, SLC16A6, CCL26, PHLDA2, TTPA, SRGAP3, LOC105601850, LOC101109939, TNNT3, THRSP, CA6, METRN, CST7, DNAJC6, TSPEAR, SYT2, STAC3, LOC101105265, TMPRSS7, SHISA6, GPHA2, LOXL4, MYLK4, LOC105609102, MUC20, DLX4, GLIS1, NPTXR, VGLL1, LOC101112296, SLC30A2, NIPAL4, S100A5, NOTCH1, MLPH, FLT4, ZNF365, SCEL, MMP17, EFNA3, RAPSN, DEGS2, C2H9orf91, LOC105608603, DGKK, KLHL31, C18H14orf132, RNF207, GJB7, NANOS1, LOC101117144, LOC101121082, ADRA1D, TAGAP, CSPG4, FMN2, GSDMA, LOC106991088, TUB, LOC105604082, AIM1L, ACE2, LOC101120060, RASGRF2, SH2B2, PRRT4, DOK7, ATP13A5, LOC101113259, STMN2, ZNF648, TMEM210, SEC14L5, MDGA2, LOC101113357, FHDC1, HPDL, CDR1, UCN3 |
