# Supplementary material for: Study on the region-specific expression of epididymis mRNA in the rams
Source: PLoS One. 2021 Jan 25;16(1):e0245933. doi: 10.1371/journal.pone.0245933 (PMC7833257; doi:10.1371/journal.pone.0245933)
Supplement: S10 Table — (DOCX) [file pone.0245933.s014.docx]

# S10 Table. 289 highly expressed genes in the caput of the epididymis

| **Gene ID** | **Other Gene ID** | **Caput_FPKM** | **Corpus_FPKM** | **Cauda_FPKM** |
| --- | --- | --- | --- | --- |
| 105613233 | LOC105613233 | 21.85 | 0.00 | 0.03 |
| 101105695 | TREM1 | 2.85 | 0.00 | 0.03 |
| 100568285 | CYP2E1 | 17.77 | 0.00 | 0.03 |
| 101120313 | LCN10 | 49.87 | 0.00 | 0.04 |
| 101118012 | ODF3B | 3.09 | 0.00 | 0.05 |
| 101117462 | CLRN3 | 5.40 | 0.00 | 0.05 |
| 101107683 | ATP6V1B1 | 8.49 | 0.00 | 0.05 |
| 443505 | SLC34A1 | 43.39 | 0.01 | 0.06 |
| 101103245 | SNX31 | 2.01 | 0.01 | 0.00 |
| 443203 | MS4A2 | 24.23 | 0.02 | 0.01 |
| 101104518 | LOC101104518 | 4.52 | 0.02 | 0.01 |
| 101117468 | PRSS27 | 3.11 | 0.02 | 0.01 |
| 101117383 | CCDC178 | 9.15 | 0.02 | 0.03 |
| 101103164 | TEKT1 | 5.94 | 0.02 | 0.06 |
| 101115624 | CHRNA9 | 7.03 | 0.02 | 0.00 |
| 101113028 | FAM78B | 1.46 | 0.02 | 0.03 |
| 101117158 | VWDE | 2.22 | 0.03 | 0.02 |
| 101116792 | DOC2A | 4.19 | 0.03 | 0.30 |
| 101103205 | C1H2orf54 | 4.14 | 0.04 | 0.04 |
| 101116841 | LOC101116841 | 3.22 | 0.04 | 0.26 |
| 101110872 | CAPSL | 6.56 | 0.05 | 0.00 |
| 101104919 | SLC13A2 | 18.31 | 0.05 | 0.03 |
| 101108928 | PIPOX | 4.65 | 0.05 | 0.05 |
| 100302705 | IZUMO1 | 9.43 | 0.05 | 0.44 |
| 101111505 | LOC101111505 | 27.93 | 0.05 | 5.26 |
| 780475 | CLDN2 | 408.03 | 0.05 | 5.37 |
| 101108090 | PPP1R32 | 7.10 | 0.06 | 0.32 |
| 101116544 | NCMAP | 12.52 | 0.06 | 0.02 |
| 101118813 | SHANK1 | 2.32 | 0.07 | 0.10 |
| 101105839 | MIXL1 | 5.66 | 0.07 | 0.01 |
| 101113262 | LRRC36 | 5.29 | 0.08 | 0.10 |
| 101107267 | TSPAN19 | 4.13 | 0.08 | 0.36 |
| 101108248 | FUT1 | 7.52 | 0.09 | 0.00 |
| 101113587 | THEG | 12.99 | 0.09 | 0.05 |
| 101103695 | EPHA6 | 1.57 | 0.09 | 0.08 |
| 101112114 | SCTR | 118.87 | 0.09 | 26.93 |
| 101118683 | LBX2 | 9.51 | 0.09 | 0.07 |
| 101114861 | LOC101114861 | 34.41 | 0.09 | 0.09 |
| 101105173 | HS3ST3A1 | 54.81 | 0.09 | 0.12 |
| 101104496 | GCNT4 | 248.86 | 0.09 | 0.16 |
| 106990147 | LTB4R2 | 3.09 | 0.10 | 0.07 |
| 101109506 | MMD2 | 57.22 | 0.10 | 12.74 |
| 101123612 | LOC101123612 | 2.14 | 0.10 | 0.15 |
| 101107134 | BTG4 | 2.28 | 0.11 | 0.09 |
| 101117095 | C13H20orf85 | 2.87 | 0.11 | 0.25 |
| 101121265 | GABRA2 | 3.45 | 0.11 | 0.06 |
| 106991447 | LOC106991447 | 7.87 | 0.11 | 1.79 |
| 101122594 | STRA8 | 4.87 | 0.12 | 0.03 |
| 101104490 | SLCO4C1 | 6.00 | 0.12 | 0.74 |
| 101122814 | HPD | 5.78 | 0.12 | 0.00 |
| 105604792 | LOC105604792 | 82.02 | 0.12 | 0.09 |
| 101112026 | SMPX | 22.86 | 0.13 | 0.72 |
| 101105058 | SMPDL3B | 14.11 | 0.13 | 2.21 |
| 101110347 | CACNG4 | 2.79 | 0.13 | 0.02 |
| 101114472 | DCLK3 | 4.85 | 0.13 | 0.12 |
| 101111412 | SLC15A2 | 33.77 | 0.14 | 0.04 |
| 101111048 | WNT11 | 2.78 | 0.14 | 0.09 |
| 101109259 | WIF1 | 5.31 | 0.15 | 0.18 |
| 105608211 | RAB42 | 3.75 | 0.15 | 0.24 |
| 101116109 | ROPN1 | 20.81 | 0.15 | 0.30 |
| 101116788 | LIPH | 3.39 | 0.16 | 0.19 |
| 101119401 | COL22A1 | 4.04 | 0.16 | 0.04 |
| 101104614 | MCCD1 | 8.92 | 0.16 | 1.35 |
| 101108901 | LOC101108901 | 2.50 | 0.17 | 0.00 |
| 101111709 | STEAP1 | 3.66 | 0.17 | 0.06 |
| 101106908 | FRMPD1 | 4.47 | 0.17 | 0.20 |
| 101108738 | SLC9A2 | 3.87 | 0.18 | 0.27 |
| 101118466 | ST8SIA6 | 173.26 | 0.18 | 18.74 |
| 101115728 | ZNF536 | 7.90 | 0.20 | 0.24 |
| 101116795 | LOC101116795 | 74.82 | 0.20 | 0.20 |
| 101106762 | LCN6 | 3374.68 | 0.21 | 0.23 |
| 101111717 | SLC22A16 | 5.62 | 0.22 | 0.81 |
| 100271925 | HEY2 | 12.07 | 0.22 | 0.09 |
| 101118952 | GNA15 | 3.80 | 0.23 | 0.68 |
| 101114662 | EPHB1 | 4.41 | 0.24 | 0.16 |
| 101120058 | ADAM28 | 1894.26 | 0.24 | 1.04 |
| 101121552 | AK7 | 8.05 | 0.26 | 0.37 |
| 101111574 | OVCH2 | 659.70 | 0.26 | 0.04 |
| 101103373 | S1PR3 | 4.92 | 0.27 | 0.12 |
| 101111183 | ADAMTSL2 | 8.02 | 0.27 | 0.25 |
| 101114110 | SPATA18 | 5.78 | 0.27 | 0.19 |
| 101111295 | CDH17 | 6.60 | 0.27 | 0.72 |
| 101102158 | MFSD6L | 3.79 | 0.28 | 0.36 |
| 443524 | INHBA | 13.73 | 0.28 | 0.82 |
| 101114837 | CELF4 | 15.11 | 0.29 | 0.14 |
| 105601862 | GPR75 | 2.24 | 0.31 | 0.08 |
| 101111960 | STEAP2 | 22.50 | 0.31 | 0.14 |
| 101115489 | RUNX2 | 5.91 | 0.31 | 0.33 |
| 101115601 | GPRIN2 | 2.04 | 0.33 | 0.13 |
| 101114907 | CHGA | 17.91 | 0.34 | 0.41 |
| 101115505 | SIDT1 | 2.57 | 0.34 | 0.30 |
| 101120004 | PRDM16 | 2.96 | 0.35 | 0.42 |
| 101115266 | RNF144A | 6.49 | 0.38 | 0.33 |
| 101108282 | TENM3 | 5.01 | 0.38 | 0.52 |
| 494440 | SLC4A4 | 6.05 | 0.39 | 0.81 |
| 101118766 | STEAP3 | 4.74 | 0.40 | 0.76 |
| 101119074 | CLEC11A | 3.05 | 0.41 | 0.35 |
| 101111006 | LOC101111006 | 15.55 | 0.43 | 0.43 |
| 101115554 | HS3ST3B1 | 50.91 | 0.43 | 1.46 |
| 443232 | CD40 | 3.62 | 0.43 | 0.49 |
| 101107103 | SLC16A10 | 11.79 | 0.44 | 0.54 |
| 101104418 | KIAA0513 | 2.33 | 0.46 | 0.38 |
| 101104100 | LY6G5C | 6007.54 | 0.46 | 0.35 |
| 101122156 | ARHGDIG | 2.47 | 0.46 | 0.39 |
| 105610450 | NPW | 2.28 | 0.47 | 0.17 |
| 101113240 | LTB4R | 18.51 | 0.48 | 0.37 |
| 101104262 | CST11 | 9467.42 | 0.49 | 0.61 |
| 101121187 | TEDDM1 | 460.14 | 0.51 | 2.42 |
| 101116261 | XYLB | 6.64 | 0.54 | 0.98 |
| 101110343 | CA8 | 30.82 | 0.54 | 2.47 |
| 101105557 | SHB | 4.80 | 0.55 | 0.60 |
| 105616801 | LOC105616801 | 197.17 | 0.55 | 0.39 |
| 443221 | RNASE10 | 6178.32 | 0.57 | 0.47 |
| 101101858 | HIVEP3 | 7.64 | 0.57 | 0.35 |
| 101121944 | ADCY8 | 15.91 | 0.57 | 0.48 |
| 101112761 | MS4A7 | 486.64 | 0.59 | 1.01 |
| 101115699 | DUSP2 | 406.28 | 0.59 | 0.70 |
| 105611318 | LOC105611318 | 281.23 | 0.59 | 14.56 |
| 101115343 | LOC101115343 | 8.80 | 0.59 | 0.42 |
| 101102970 | LCNL1 | 92.29 | 0.60 | 0.48 |
| 101110109 | DECR2 | 4.02 | 0.61 | 0.59 |
| 101107915 | VTCN1 | 4.81 | 0.61 | 0.76 |
| 101114540 | TMC8 | 3.28 | 0.62 | 0.78 |
| 101104089 | FUT4 | 3.58 | 0.62 | 0.51 |
| 101102372 | PLCL1 | 4.43 | 0.62 | 0.42 |
| 101115635 | RHBDL3 | 8.02 | 0.62 | 0.40 |
| 101111757 | PDE4B | 11.43 | 0.63 | 0.54 |
| 101113212 | CD164L2 | 4.92 | 0.64 | 1.03 |
| 101122042 | GPR153 | 7.26 | 0.67 | 1.12 |
| 101106589 | SPRY4 | 5.49 | 0.68 | 0.70 |
| 101105716 | HSD3B7 | 5.10 | 0.68 | 0.18 |
| 101111860 | TMEM37 | 11.11 | 0.68 | 0.73 |
| 101103244 | PREX2 | 6.61 | 0.69 | 0.94 |
| 101112196 | RAPGEF4 | 3.63 | 0.71 | 0.65 |
| 101102696 | QPRT | 10.17 | 0.72 | 0.22 |
| 101112021 | CLIC6 | 11.76 | 0.75 | 1.06 |
| 101113663 | KIF5C | 6.29 | 0.77 | 0.70 |
| 101116569 | CXXC5 | 17.95 | 0.78 | 2.28 |
| 101120527 | STRA6 | 12.04 | 0.78 | 1.80 |
| 101115372 | PLA2G4B | 9.23 | 0.79 | 1.77 |
| 101102907 | RTN1 | 8.03 | 0.82 | 0.46 |
| 101120179 | LOC101120179 | 6.21 | 0.82 | 0.74 |
| 101122497 | SIK1 | 4.20 | 0.84 | 0.96 |
| 101120418 | WISP1 | 32.28 | 0.85 | 6.83 |
| 101115118 | WDR86 | 8.00 | 0.85 | 0.50 |
| 101115917 | ACTN3 | 8.40 | 0.87 | 0.75 |
| 101119743 | ARHGEF16 | 5.16 | 0.87 | 1.29 |
| 101104377 | RAP1GAP | 5.39 | 0.89 | 0.38 |
| 101111562 | NXPH3 | 46.23 | 0.90 | 11.34 |
| 101106250 | LCN8 | 14686.46 | 0.90 | 0.67 |
| 654334 | SLCO2A1 | 9.91 | 0.92 | 0.39 |
| 101120123 | SLC5A3 | 5.18 | 0.95 | 1.21 |
| 101103676 | INF2 | 6.23 | 0.97 | 0.59 |
| 101103475 | SNX24 | 13.89 | 0.97 | 1.45 |
| 101123048 | ENPP1 | 23.06 | 0.97 | 4.02 |
| 443390 | OXT | 13.04 | 0.98 | 1.86 |
| 101110092 | CPEB1 | 4.83 | 1.00 | 0.38 |
| 101106626 | EGFLAM | 26.63 | 1.04 | 0.91 |
| 101104193 | GAL3ST3 | 12.02 | 1.08 | 0.65 |
| 101121383 | LRRC8B | 13.69 | 1.10 | 0.92 |
| 101109433 | LRRC23 | 8.52 | 1.15 | 1.00 |
| 101108560 | SYCP3 | 6.03 | 1.17 | 0.83 |
| 101109232 | STK32C | 4.73 | 1.17 | 0.59 |
| 101112956 | PXDN | 7.24 | 1.18 | 0.78 |
| 101111242 | LOC101111242 | 2338.91 | 1.18 | 82.72 |
| 101113635 | DDAH1 | 10.17 | 1.18 | 2.45 |
| 101107876 | CLDN10 | 89.97 | 1.19 | 1.15 |
| 101105504 | RASGRP1 | 7.40 | 1.20 | 1.54 |
| 101105202 | ARHGAP31 | 13.37 | 1.22 | 0.86 |
| 101116425 | TRIM29 | 49.09 | 1.23 | 3.28 |
| 101108281 | DYDC2 | 5.08 | 1.24 | 0.89 |
| 101103253 | RGSL1 | 3452.21 | 1.25 | 1.11 |
| 101122606 | ST3GAL1 | 34.73 | 1.26 | 2.51 |
| 101119848 | ITGA2 | 21.09 | 1.29 | 1.14 |
| 101105629 | VEGFC | 16.77 | 1.32 | 0.61 |
| 101110694 | SLC17A9 | 30.98 | 1.34 | 2.04 |
| 101108335 | APRT | 19.16 | 1.34 | 1.22 |
| 101110254 | MYBPH | 6.99 | 1.37 | 0.09 |
| 101106554 | GCNT2 | 5.57 | 1.37 | 0.68 |
| 101114660 | ZBTB7C | 14.28 | 1.41 | 1.17 |
| 101120553 | ZDHHC9 | 25.44 | 1.42 | 0.95 |
| 554335 | ACE | 16.67 | 1.42 | 1.31 |
| 101114859 | GALNT6 | 11.37 | 1.42 | 0.57 |
| 101104709 | GPC1 | 20.17 | 1.46 | 1.77 |
| 101103584 | LOC101103584 | 1223.48 | 1.58 | 1.58 |
| 101109816 | ADORA1 | 7.99 | 1.61 | 0.00 |
| 101105076 | ADAMTS10 | 15.66 | 1.61 | 0.94 |
| 101121631 | NTRK3 | 20.14 | 1.61 | 1.32 |
| 101113341 | LOC101113341 | 64.06 | 1.61 | 5.56 |
| 101119855 | LY6G5B | 2943.26 | 1.62 | 1.61 |
| 101118903 | CRYBB1 | 21.05 | 1.63 | 0.80 |
| 101110344 | ETV4 | 95.74 | 1.64 | 4.13 |
| 554323 | TGFB3 | 9.48 | 1.65 | 0.70 |
| 101117367 | EGLN3 | 12.11 | 1.67 | 1.80 |
| 101122041 | MRC2 | 28.93 | 1.68 | 1.45 |
| 101107650 | RGS10 | 8.81 | 1.71 | 2.07 |
| 101118602 | LCN9 | 6235.10 | 1.76 | 12.56 |
| 101105882 | S100G | 1137.17 | 1.77 | 0.35 |
| 101123150 | SEMA5A | 17.89 | 1.83 | 1.88 |
| 100302354 | SDR16C5 | 23.10 | 1.87 | 0.16 |
| 101112600 | PDGFC | 13.13 | 1.87 | 1.59 |
| 101105242 | SLC12A2 | 16.63 | 1.93 | 1.07 |
| 101103274 | DAAM2 | 24.74 | 1.97 | 1.37 |
| 101122914 | TMEM8A | 10.74 | 2.00 | 1.13 |
| 101115379 | SHISA2 | 15.23 | 2.00 | 2.47 |
| 105607151 | C2H9orf153 | 21.17 | 2.06 | 2.10 |
| 780521 | LEF1 | 55.64 | 2.09 | 1.63 |
| 443279 | CHI3L1 | 71.77 | 2.09 | 2.23 |
| 101120442 | KREMEN1 | 17.22 | 2.14 | 2.92 |
| 101102694 | PASK | 19.01 | 2.15 | 1.96 |
| 101120263 | TSHZ2 | 22.59 | 2.18 | 1.17 |
| 101111672 | ROM1 | 13.05 | 2.28 | 1.41 |
| 101108123 | CARD10 | 18.11 | 2.29 | 1.45 |
| 101105944 | NRIP3 | 24.46 | 2.30 | 0.94 |
| 101111479 | KCTD15 | 20.05 | 2.35 | 1.46 |
| 101113190 | S100B | 179.84 | 2.42 | 1.45 |
| 101111284 | CCDC69 | 26.09 | 2.44 | 1.23 |
| 101106094 | NCF2 | 19.17 | 2.45 | 2.13 |
| 101112277 | GATSL2 | 24.13 | 2.49 | 4.08 |
| 101104593 | ENAH | 42.97 | 2.63 | 1.83 |
| 101110747 | POC1B | 43.08 | 2.67 | 6.61 |
| 101109268 | LOC101109268 | 18.78 | 2.69 | 1.17 |
| 101104977 | ST3GAL5 | 12.70 | 2.77 | 0.98 |
| 101121743 | PDE9A | 14.36 | 2.79 | 1.21 |
| 101113519 | TPPP3 | 34.14 | 2.95 | 3.59 |
| 101120942 | ADGRG1 | 80.35 | 2.96 | 6.40 |
| 101120887 | ITGB5 | 34.38 | 3.09 | 2.62 |
| 100145856 | HEXA | 37.45 | 3.13 | 3.31 |
| 101113161 | PYCR1 | 20.84 | 3.15 | 3.94 |
| 101110890 | PITPNM1 | 54.86 | 3.34 | 2.73 |
| 101111472 | PLA2G4A | 21.11 | 3.45 | 2.57 |
| 101115418 | ETV5 | 46.24 | 3.51 | 3.68 |
| 101111208 | CBX8 | 18.66 | 3.71 | 3.65 |
| 106991729 | BDKRB1 | 39.79 | 3.91 | 2.40 |
| 443282 | GGTA2P | 65.64 | 4.10 | 6.31 |
| 105609445 | TGFBR3L | 58.21 | 4.24 | 2.90 |
| 101102499 | KCTD12 | 95.49 | 4.25 | 4.69 |
| 101106170 | SLC34A2 | 49.02 | 4.25 | 4.88 |
| 101117637 | P2RY14 | 75.17 | 4.29 | 18.29 |
| 101110878 | ISLR | 43.01 | 4.33 | 3.03 |
| 101122661 | RHOU | 18.13 | 4.36 | 2.45 |
| 101112685 | CST6 | 470.98 | 4.55 | 3.88 |
| 101108418 | ARRB1 | 24.91 | 4.72 | 4.64 |
| 101110105 | CRTAC1 | 35.01 | 4.91 | 0.51 |
| 101104949 | B4GALT4 | 295.18 | 4.98 | 6.54 |
| 101116016 | C26H8orf4 | 22.31 | 4.99 | 5.27 |
| 101105976 | LOC101105976 | 6944.62 | 5.32 | 914.18 |
| 101114832 | ACP6 | 39.12 | 5.35 | 5.80 |
| 101113769 | TEX30 | 23.28 | 5.62 | 4.93 |
| 101109408 | LITAF | 42.99 | 6.45 | 5.74 |
| 101122993 | LTBP3 | 32.93 | 6.46 | 5.03 |
| 105606076 | LOC105606076 | 26.67 | 6.47 | 4.96 |
| 100187549 | HPCAL1 | 92.30 | 6.53 | 3.27 |
| 101103913 | PMEPA1 | 33.70 | 6.87 | 3.78 |
| 101103384 | UBXN10 | 45.49 | 6.95 | 10.75 |
| 101113480 | SLC1A4 | 162.75 | 6.97 | 2.83 |
| 101116331 | MXRA7 | 28.55 | 6.98 | 5.00 |
| 100307035 | CAT | 153.64 | 7.26 | 19.61 |
| 101120587 | TMEM98 | 57.59 | 7.28 | 5.04 |
| 101117975 | RHBDL2 | 73.58 | 8.62 | 12.84 |
| 101108821 | CHPT1 | 90.28 | 8.66 | 6.25 |
| 101112806 | BHLHE41 | 46.04 | 8.70 | 9.00 |
| 101116327 | CARS2 | 95.98 | 9.37 | 11.70 |
| 443049 | PDXK | 76.70 | 9.88 | 19.28 |
| 101109421 | AKR7A2 | 128.97 | 11.00 | 7.21 |
| 101104845 | ENTPD6 | 80.58 | 11.53 | 12.77 |
| 105611550 | LOC105611550 | 144.49 | 11.70 | 7.32 |
| 101112385 | MON2 | 93.17 | 11.76 | 10.38 |
| 443411 | LOC443411 | 324.53 | 12.29 | 9.39 |
| 443115 | MMP2 | 301.13 | 12.89 | 4.65 |
| 101106452 | LOC101106452 | 57.12 | 13.96 | 9.93 |
| 101105675 | CD34 | 193.05 | 14.64 | 12.75 |
| 100462691 | CRISP3 | 348.53 | 16.49 | 13.57 |
| 101111279 | SNX2 | 124.64 | 19.09 | 31.35 |
| 101107655 | PXYLP1 | 138.42 | 20.57 | 33.73 |
| 678680 | LGMN | 336.81 | 20.58 | 23.45 |
| 101104858 | TPST2 | 473.65 | 22.72 | 22.61 |
| 101103632 | PLA2G2C | 119.87 | 26.97 | 19.27 |
| 101115062 | MUC15 | 579.78 | 29.97 | 139.91 |
| 101115125 | TMEM150C | 1673.00 | 52.67 | 182.40 |
| 443331 | TIMP1 | 1028.81 | 54.77 | 24.33 |
| 101108977 | RBP1 | 332.43 | 54.89 | 30.08 |
| 101112346 | RCN1 | 3215.60 | 57.82 | 409.93 |
| 101103917 | BSPH1 | 713.72 | 74.27 | 113.04 |
| 101108654 | LOC101108654 | 9779.92 | 94.22 | 191.40 |
| 101104633 | AP1S2 | 587.24 | 107.84 | 134.94 |
| 100913163 | GPX5 | 58448.62 | 117.60 | 45.47 |
| 105606698 | LOC105606698 | 1484.86 | 246.84 | 6.95 |
| 105603738 | DEFB110 | 7132.01 | 453.05 | 768.32 |
