# Supplementary material for: Study on the region-specific expression of epididymis mRNA in the rams
Source: PLoS One. 2021 Jan 25;16(1):e0245933. doi: 10.1371/journal.pone.0245933 (PMC7833257; doi:10.1371/journal.pone.0245933)
Supplement: S11 Table — (DOCX) [file pone.0245933.s015.docx]

# S11 Table. 136 highly expressed genes in the corpus of the epididymis

| **Gene ID** | **Other Gene ID** | **Corpus_FPKM** | **Caput_FPKM** | **Cauda_FPKM** |
| --- | --- | --- | --- | --- |
| 554328 | PAX4 | 2.89 | 0.00 | 0.01 |
| 101113407 | MGAM | 2.90 | 0.00 | 0.01 |
| 101118117 | GALR2 | 10.36 | 0.00 | 0.07 |
| 101110195 | LOC101110195 | 1.25 | 0.00 | 0.13 |
| 101111669 | LOC101111669 | 7.19 | 0.00 | 0.20 |
| 105613453 | LOC105613453 | 25.39 | 0.00 | 1.17 |
| 101103323 | GJD2 | 3.20 | 0.00 | 0.06 |
| 101103729 | VGLL2 | 14.34 | 0.01 | 0.02 |
| 101115740 | LOC101115740 | 2.04 | 0.01 | 0.08 |
| 101115034 | DUOXA1 | 39.01 | 0.01 | 6.79 |
| 101113761 | LOC101113761 | 4.15 | 0.02 | 0.20 |
| 101105537 | PRSS16 | 8.73 | 0.03 | 0.03 |
| 101113331 | LOC101113331 | 24.21 | 0.03 | 0.89 |
| 101113588 | UNC13A | 9.63 | 0.03 | 1.39 |
| 101118164 | LOC101118164 | 11.36 | 0.03 | 0.06 |
| 101103838 | PIWIL4 | 3.82 | 0.04 | 0.16 |
| 101112780 | CSTA | 41.50 | 0.05 | 3.77 |
| 105615088 | LOC105615088 | 1.43 | 0.05 | 0.10 |
| 101107719 | KISS1 | 2.88 | 0.05 | 0.22 |
| 101113073 | LOC101113073 | 470.44 | 0.06 | 16.27 |
| 101106273 | ZACN | 3.54 | 0.06 | 0.08 |
| 101103967 | GDF3 | 270.30 | 0.07 | 0.94 |
| 101119530 | LOC101119530 | 4.05 | 0.08 | 0.47 |
| 101105461 | GNAL | 4.91 | 0.09 | 0.10 |
| 101115303 | ABCC12 | 6.15 | 0.09 | 0.06 |
| 101107141 | CADPS | 3.86 | 0.09 | 0.15 |
| 105613768 | LOC105613768 | 13.74 | 0.10 | 0.17 |
| 101108621 | TVP23A | 3.44 | 0.13 | 0.02 |
| 106991725 | LOC106991725 | 3.61 | 0.13 | 0.30 |
| 105616123 | LOC105616123 | 11.30 | 0.14 | 1.03 |
| 106991069 | LOC106991069 | 2.81 | 0.17 | 0.00 |
| 101109766 | SH2D4B | 4.98 | 0.18 | 1.02 |
| 101112606 | LOC101112606 | 51.36 | 0.19 | 0.95 |
| 101105382 | CLDN16 | 2.90 | 0.20 | 0.48 |
| 101111915 | LOC101111915 | 9.18 | 0.20 | 0.27 |
| 101106916 | PLA2G2D | 4.92 | 0.20 | 0.64 |
| 443246 | SLC1A3 | 1.53 | 0.25 | 0.07 |
| 101119383 | KIF5A | 1.93 | 0.25 | 0.44 |
| 101123427 | IVL | 189.35 | 0.25 | 23.05 |
| 443361 | NPY1R | 15.41 | 0.26 | 2.60 |
| 106991631 | LOC106991631 | 5.28 | 0.27 | 0.02 |
| 106991896 | LOC106991896 | 1.72 | 0.27 | 0.25 |
| 101116157 | LOC101116157 | 5.72 | 0.27 | 0.35 |
| 105602979 | LOC105602979 | 30.85 | 0.29 | 1.92 |
| 105605056 | LOC105605056 | 66.07 | 0.30 | 1.80 |
| 105602646 | LOC105602646 | 8.91 | 0.30 | 1.02 |
| 101109833 | TMEM89 | 4.78 | 0.32 | 0.16 |
| 101109687 | PROKR1 | 17.80 | 0.33 | 0.50 |
| 106991450 | LOC106991450 | 16.64 | 0.34 | 1.38 |
| 101109910 | STC2 | 4.12 | 0.34 | 0.89 |
| 101109206 | MFRP | 36.99 | 0.34 | 3.30 |
| 105615953 | LOC105615953 | 12.94 | 0.36 | 0.97 |
| 101121083 | FAM19A5 | 4.03 | 0.36 | 0.03 |
| 101109034 | DEFB129 | 2904.21 | 0.38 | 145.43 |
| 101112976 | CREB5 | 3.61 | 0.40 | 0.88 |
| 101111824 | WFDC8 | 10.44 | 0.41 | 0.55 |
| 101114541 | MFSD4 | 21.37 | 0.41 | 0.88 |
| 101112340 | SPINT4 | 18652.02 | 0.42 | 318.95 |
| 101107979 | GOLT1A | 58.58 | 0.43 | 2.63 |
| 101117552 | MRO | 20.60 | 0.43 | 4.86 |
| 105615865 | LOC105615865 | 18.00 | 0.44 | 1.57 |
| 101112081 | WFDC13 | 2087.55 | 0.45 | 190.38 |
| 101111972 | DUOX1 | 58.18 | 0.48 | 11.86 |
| 105608895 | LOC105608895 | 10.15 | 0.56 | 1.62 |
| 101105792 | DLGAP1 | 4.03 | 0.58 | 0.38 |
| 101103188 | TDRD9 | 13.49 | 0.58 | 3.28 |
| 101114311 | LOC101114311 | 13.41 | 0.58 | 0.81 |
| 101117670 | ARL9 | 4.86 | 0.59 | 0.72 |
| 105605547 | PRRG3 | 6.48 | 0.61 | 0.75 |
| 105605116 | LOC105605116 | 203.42 | 0.63 | 3.39 |
| 101109397 | LOC101109397 | 15.33 | 0.64 | 0.69 |
| 105604152 | LOC105604152 | 5.56 | 0.70 | 0.66 |
| 101115696 | METTL7B | 252.85 | 0.80 | 41.67 |
| 101122905 | DUSP8 | 5.78 | 0.82 | 0.94 |
| 101110562 | SDK1 | 3.60 | 0.85 | 0.70 |
| 101107008 | SLC6A15 | 8.49 | 0.85 | 0.01 |
| 105605950 | LOC105605950 | 394.23 | 0.88 | 11.02 |
| 101117553 | CCBE1 | 6.00 | 0.92 | 0.64 |
| 101113074 | PLCB2 | 10.72 | 0.94 | 1.01 |
| 101120830 | RGL3 | 19.27 | 0.94 | 0.80 |
| 101118587 | GK | 9.27 | 0.96 | 1.53 |
| 101114059 | ASB2 | 11.65 | 0.96 | 2.61 |
| 101115627 | ITPKA | 5.64 | 0.99 | 0.14 |
| 101123371 | FAM131B | 9.75 | 0.99 | 1.89 |
| 101105168 | DIAPH3 | 5.62 | 1.03 | 1.05 |
| 101115959 | LOC101115959 | 8.89 | 1.04 | 0.57 |
| 101117454 | BSN | 6.05 | 1.07 | 0.09 |
| 101114470 | FAM174B | 4.47 | 1.10 | 0.58 |
| 101120081 | SPOCK1 | 17.09 | 1.10 | 0.38 |
| 101110974 | LOC101110974 | 72.13 | 1.25 | 2.44 |
| 101112133 | LYPD8 | 39.26 | 1.26 | 0.52 |
| 101119394 | GPAT3 | 6.82 | 1.33 | 1.73 |
| 101116862 | LOC101116862 | 16.12 | 1.41 | 1.87 |
| 101107949 | CERCAM | 14.58 | 1.49 | 3.25 |
| 101106849 | FSIP1 | 7.26 | 1.64 | 1.19 |
| 101108713 | TTC24 | 16.07 | 1.64 | 1.59 |
| 101119787 | RET | 35.11 | 1.65 | 11.56 |
| 101110813 | CARD11 | 36.76 | 1.67 | 4.48 |
| 101108745 | FUT5 | 38.88 | 1.84 | 8.32 |
| 101103681 | SYTL2 | 15.63 | 1.96 | 1.73 |
| 101106664 | PLA2G2F | 53.17 | 2.01 | 5.56 |
| 101116716 | SLC8A1 | 12.17 | 2.03 | 1.85 |
| 101119694 | RASEF | 9.22 | 2.06 | 1.95 |
| 780451 | CES5A | 3163.90 | 2.14 | 746.41 |
| 443019 | QPCT | 31.08 | 2.19 | 0.40 |
| 101108488 | SLC39A8 | 98.40 | 2.49 | 3.73 |
| 105603000 | GGT1 | 33.16 | 2.53 | 2.87 |
| 101111297 | MEDAG | 14.87 | 2.74 | 3.14 |
| 101105968 | IMPA2 | 17.03 | 3.16 | 4.10 |
| 101110769 | PKIA | 22.71 | 3.46 | 0.11 |
| 105602015 | LOC105602015 | 626.91 | 3.55 | 58.14 |
| 101116129 | RIPPLY1 | 20.13 | 3.68 | 0.04 |
| 101121758 | GLIPR1 | 39.65 | 3.75 | 2.20 |
| 101117587 | LOC101117587 | 3036.03 | 3.98 | 41.78 |
| 101101969 | RASGRP3 | 34.98 | 4.14 | 4.06 |
| 101115988 | LOC101115988 | 70.05 | 4.65 | 0.26 |
| 101116939 | PSTPIP1 | 49.08 | 4.74 | 4.39 |
| 101112423 | GGT5 | 46.95 | 5.18 | 4.24 |
| 101121563 | LOC101121563 | 23.53 | 5.82 | 2.53 |
| 101106905 | NINJ1 | 37.37 | 6.50 | 8.28 |
| 101117846 | MAN2B2 | 2808.85 | 6.53 | 67.53 |
| 101121820 | LOC101121820 | 34.92 | 8.52 | 7.70 |
| 101103697 | AQP7 | 276.10 | 8.69 | 0.96 |
| 101117712 | PATE2 | 243.02 | 11.28 | 43.40 |
| 101102187 | PRSS23 | 49.29 | 12.00 | 6.62 |
| 101115292 | ABCA5 | 274.93 | 12.52 | 20.09 |
| 101115732 | LOC101115732 | 416.88 | 14.22 | 1.00 |
| 105602415 | C1QTNF5 | 186.28 | 16.60 | 18.03 |
| 101108169 | FAM3D | 351.97 | 27.58 | 25.81 |
| 101120631 | RASSF4 | 138.12 | 32.85 | 22.15 |
| 101117691 | LOC101117691 | 205.19 | 35.46 | 18.31 |
| 101121831 | MECR | 182.03 | 37.53 | 23.16 |
| 101117955 | LOC101117955 | 484.03 | 43.22 | 41.62 |
| 101113986 | PEBP4 | 393.36 | 85.04 | 35.46 |
| 554321 | LTF | 1427.27 | 126.69 | 58.73 |
| 101113728 | LOC101113728 | 8298.61 | 1544.77 | 1123.17 |
