# Supplementary material for: Study on the region-specific expression of epididymis mRNA in the rams
Source: PLoS One. 2021 Jan 25;16(1):e0245933. doi: 10.1371/journal.pone.0245933 (PMC7833257; doi:10.1371/journal.pone.0245933)
Supplement: S12 Table — (DOCX) [file pone.0245933.s016.docx]

# S12 Table. 233 highly expressed genes in the cauda of the epididymis

| **Gene ID** | **Other Gene ID** | **Cauda_FPKM** | **Corpus_FPKM** | **Caput_FPKM** |
| --- | --- | --- | --- | --- |
| 101107907 | C20H6orf136 | 19.41 | 0.00 | 0.00 |
| 101106349 | UCN3 | 1.25 | 0.00 | 0.00 |
| 101119894 | GPD1 | 35.59 | 0.00 | 4.19 |
| 101120988 | PTH2R | 14.17 | 0.01 | 0.50 |
| 101117380 | LOXL4 | 5.50 | 0.01 | 0.17 |
| 101120035 | TMPRSS7 | 5.90 | 0.01 | 0.02 |
| 443388 | SERPINA1 | 44.38 | 0.01 | 0.14 |
| 101113126 | FMN2 | 2.89 | 0.02 | 0.08 |
| 101117966 | MMP17 | 3.95 | 0.02 | 0.04 |
| 101116570 | LOC101116570 | 18.23 | 0.02 | 0.02 |
| 101116029 | AIM1L | 2.56 | 0.02 | 0.14 |
| 101114598 | KCNIP3 | 49.86 | 0.03 | 2.66 |
| 105603239 | C18H14orf132 | 3.30 | 0.03 | 0.03 |
| 101112296 | LOC101112296 | 4.30 | 0.03 | 0.13 |
| 106990376 | SPINK14 | 44.01 | 0.04 | 0.00 |
| 101121682 | DOK7 | 2.34 | 0.04 | 0.02 |
| 101117861 | CA6 | 7.91 | 0.04 | 0.05 |
| 100272215 | MLPH | 4.05 | 0.05 | 0.49 |
| 101123536 | LOC101123536 | 55.91 | 0.05 | 0.04 |
| 101104292 | SEC14L5 | 2.12 | 0.06 | 0.01 |
| 101103696 | TSPEAR | 6.45 | 0.08 | 0.10 |
| 101117226 | VGLL1 | 4.42 | 0.09 | 0.05 |
| 101113259 | LOC101113259 | 2.21 | 0.09 | 0.08 |
| 101105265 | LOC101105265 | 5.96 | 0.10 | 0.04 |
| 101103269 | RLBP1 | 21.10 | 0.10 | 0.04 |
| 101113394 | EXTL1 | 19.41 | 0.10 | 0.07 |
| 101115130 | GJB7 | 3.24 | 0.10 | 0.52 |
| 101104163 | SHISA6 | 5.67 | 0.11 | 0.19 |
| 101104222 | LOC101104222 | 13.74 | 0.11 | 0.13 |
| 101120060 | LOC101120060 | 2.52 | 0.11 | 0.18 |
| 101102077 | SELL | 31.30 | 0.11 | 1.52 |
| 101117314 | SLC30A2 | 4.24 | 0.13 | 0.07 |
| 105604082 | LOC105604082 | 2.59 | 0.14 | 0.08 |
| 101119430 | GPHA2 | 5.63 | 0.14 | 0.02 |
| 105613568 | PHLDA2 | 10.19 | 0.15 | 0.37 |
| 101110739 | DGKK | 3.34 | 0.15 | 0.10 |
| 101118216 | LOC101118216 | 37.62 | 0.15 | 0.15 |
| 101108733 | MGAT4C | 42.59 | 0.16 | 0.05 |
| 101114565 | KLHL31 | 3.33 | 0.17 | 0.03 |
| 101121099 | MDGA2 | 2.10 | 0.17 | 0.25 |
| 101122152 | NANOS1 | 3.23 | 0.17 | 0.51 |
| 101117636 | KLHL14 | 31.82 | 0.17 | 3.07 |
| 101117144 | LOC101117144 | 3.15 | 0.19 | 0.07 |
| 101109628 | DLX4 | 4.70 | 0.19 | 0.16 |
| 101107321 | ATP13A5 | 2.30 | 0.20 | 0.03 |
| 101110189 | THRSP | 8.12 | 0.21 | 0.08 |
| 105606696 | LOC105606696 | 20.83 | 0.21 | 0.02 |
| 106991088 | LOC106991088 | 2.74 | 0.24 | 0.06 |
| 101108683 | TUB | 2.69 | 0.24 | 0.34 |
| 105605561 | CDR1 | 1.65 | 0.24 | 0.16 |
| 105608603 | LOC105608603 | 3.34 | 0.25 | 0.11 |
| 101119084 | HPDL | 1.68 | 0.26 | 0.12 |
| 101111237 | DNAJC6 | 6.81 | 0.26 | 0.18 |
| 101102747 | ZNF648 | 2.20 | 0.27 | 0.02 |
| 101104467 | TNNT3 | 8.15 | 0.27 | 0.26 |
| 443305 | SELP | 18.93 | 0.28 | 0.40 |
| 101117210 | GLIS1 | 4.63 | 0.30 | 0.17 |
| 101117815 | SH2B2 | 2.50 | 0.30 | 0.31 |
| 101111294 | STMN2 | 2.20 | 0.32 | 0.24 |
| 101122406 | MUC20 | 5.04 | 0.35 | 0.31 |
| 101105583 | GSDMA | 2.77 | 0.35 | 0.15 |
| 101103405 | SCEL | 3.96 | 0.36 | 0.10 |
| 101121185 | ALOX15B | 19.96 | 0.37 | 0.67 |
| 101106370 | SRGAP3 | 9.89 | 0.40 | 0.26 |
| 101116051 | FABP6 | 50.64 | 0.40 | 0.16 |
| 101123666 | FHDC1 | 1.77 | 0.41 | 0.34 |
| 101121934 | RASGRF2 | 2.51 | 0.41 | 0.40 |
| 101105108 | CSPG4 | 2.96 | 0.41 | 0.56 |
| 101120016 | RAPSN | 3.64 | 0.41 | 0.29 |
| 101117297 | CCL26 | 11.21 | 0.41 | 0.50 |
| 492300 | SLC5A1 | 46.26 | 0.43 | 0.04 |
| 101112653 | IL20RA | 29.47 | 0.45 | 4.56 |
| 100170316 | ADRA1D | 2.99 | 0.46 | 0.35 |
| 101121082 | LOC101121082 | 3.02 | 0.46 | 0.48 |
| 101113357 | LOC101113357 | 1.99 | 0.47 | 0.05 |
| 101103891 | PRRT4 | 2.47 | 0.47 | 0.23 |
| 101105239 | HOXA10 | 13.44 | 0.49 | 0.19 |
| 105612422 | TMEM210 | 2.18 | 0.51 | 0.32 |
| 101109975 | TAGAP | 2.97 | 0.52 | 0.67 |
| 101112833 | RASL10B | 15.02 | 0.53 | 1.09 |
| 101103622 | ACE2 | 2.55 | 0.54 | 0.31 |
| 101122233 | SYT6 | 19.74 | 0.54 | 0.02 |
| 101120047 | RASGEF1A | 5.59 | 0.55 | 1.22 |
| 101118389 | DEGS2 | 3.61 | 0.55 | 0.07 |
| 101116069 | SLC16A6 | 13.41 | 0.57 | 0.39 |
| 101104541 | S100A5 | 4.12 | 0.61 | 0.39 |
| 493773 | NPPC | 13.59 | 0.62 | 0.24 |
| 100526668 | TTPA | 10.13 | 0.63 | 0.00 |
| 101122160 | ZNF365 | 4.00 | 0.64 | 0.67 |
| 101114341 | SYT2 | 6.13 | 0.65 | 0.15 |
| 105601850 | LOC105601850 | 8.76 | 0.66 | 0.49 |
| 101123024 | STAC3 | 6.03 | 0.69 | 0.43 |
| 101109939 | LOC101109939 | 8.62 | 0.70 | 0.45 |
| 101117952 | RNF207 | 3.28 | 0.70 | 0.12 |
| 101107260 | LOC101107260 | 57.15 | 0.70 | 1.26 |
| 101115414 | EFNA3 | 3.82 | 0.73 | 0.24 |
| 101115020 | NOTCH1 | 4.07 | 0.73 | 0.85 |
| 101103211 | C2H9orf91 | 3.36 | 0.74 | 0.76 |
| 105603910 | MYLK4 | 5.12 | 0.75 | 0.24 |
| 101117299 | LOC101117299 | 17.17 | 0.77 | 1.80 |
| 101102355 | EVA1C | 7.46 | 0.78 | 1.05 |
| 101113410 | NIPAL4 | 4.12 | 0.83 | 0.29 |
| 105609482 | NPTXR | 4.58 | 0.84 | 0.93 |
| 101112874 | FCRL3 | 10.18 | 0.86 | 1.28 |
| 101110897 | METRN | 7.44 | 0.87 | 0.57 |
| 101119210 | ADAMTSL5 | 14.87 | 0.91 | 0.88 |
| 101107151 | GRK5 | 12.68 | 0.93 | 1.10 |
| 101122803 | LOC101122803 | 34.17 | 0.93 | 0.61 |
| 101103884 | LCLAT1 | 36.45 | 0.95 | 2.27 |
| 101106577 | LOC101106577 | 8.56 | 0.95 | 1.21 |
| 105605702 | MAMLD1 | 58.41 | 0.96 | 5.25 |
| 105609102 | LOC105609102 | 5.07 | 0.98 | 0.78 |
| 101117247 | FLT4 | 4.03 | 0.99 | 0.17 |
| 101105518 | CST7 | 7.42 | 0.99 | 0.41 |
| 101106194 | SDCBP2 | 4.77 | 1.00 | 0.21 |
| 101110032 | L1CAM | 25.86 | 1.01 | 0.42 |
| 101104256 | RTN4RL1 | 6.28 | 1.06 | 0.92 |
| 101109219 | LOC101109219 | 14.31 | 1.08 | 2.83 |
| 443488 | SCNN1A | 30.93 | 1.10 | 1.00 |
| 101118337 | TNC | 8.48 | 1.19 | 0.47 |
| 101118806 | EYA2 | 7.00 | 1.19 | 0.82 |
| 101116919 | SACS | 7.80 | 1.22 | 0.44 |
| 101114192 | C5H19orf24 | 8.61 | 1.23 | 1.10 |
| 101122001 | NOSTRIN | 5.44 | 1.27 | 1.00 |
| 101109916 | TGM5 | 20.89 | 1.29 | 0.04 |
| 101111169 | ABCA2 | 18.60 | 1.31 | 2.38 |
| 443038 | CAPN3 | 6.16 | 1.36 | 0.90 |
| 101108229 | ART3 | 31.33 | 1.39 | 1.57 |
| 101112406 | RAC3 | 9.74 | 1.40 | 1.11 |
| 101107790 | SALL2 | 30.55 | 1.43 | 7.28 |
| 101104679 | TAT | 42.76 | 1.44 | 2.74 |
| 101122895 | DNASE1L3 | 65.68 | 1.46 | 1.07 |
| 554254 | TLR7 | 13.90 | 1.47 | 1.52 |
| 101115829 | DAO | 11.62 | 1.47 | 2.27 |
| 101102136 | NCS1 | 11.79 | 1.47 | 2.04 |
| 101122707 | PLXDC1 | 6.11 | 1.48 | 0.97 |
| 101118949 | ABLIM3 | 15.70 | 1.49 | 1.39 |
| 101119804 | LOC101119804 | 12.50 | 1.49 | 0.45 |
| 101103367 | TFR2 | 19.61 | 1.50 | 0.46 |
| 101112033 | GUCY2F | 9.49 | 1.53 | 0.02 |
| 101123247 | PIK3R3 | 20.47 | 1.56 | 0.81 |
| 101103617 | SHE | 6.43 | 1.57 | 0.98 |
| 101104513 | NPL | 22.83 | 1.60 | 4.90 |
| 101104600 | UPK2 | 68.60 | 1.62 | 0.00 |
| 105605046 | ADIRF | 13.43 | 1.62 | 2.87 |
| 780509 | MB | 16.32 | 1.71 | 0.38 |
| 101116818 | GPSM1 | 34.02 | 1.74 | 3.59 |
| 101114004 | SLC4A8 | 11.49 | 1.79 | 0.89 |
| 101122618 | NNAT | 12.07 | 1.83 | 1.29 |
| 101106315 | LOC101106315 | 17.92 | 1.84 | 3.38 |
| 101102704 | NHSL2 | 8.18 | 1.87 | 0.66 |
| 101114027 | PDE10A | 17.47 | 1.94 | 0.64 |
| 101121159 | LOC101121159 | 10.26 | 1.94 | 0.27 |
| 101112591 | RYR1 | 11.10 | 2.01 | 0.09 |
| 101117042 | HIP1 | 21.36 | 2.03 | 2.12 |
| 105604882 | LOC105604882 | 24.92 | 2.04 | 0.48 |
| 101115022 | GRIP1 | 10.37 | 2.06 | 0.60 |
| 101102647 | SCG5 | 35.03 | 2.06 | 0.77 |
| 101103826 | PNMT | 36.55 | 2.08 | 0.73 |
| 101121444 | LAMA5 | 13.42 | 2.10 | 2.32 |
| 101121016 | PKIB | 19.29 | 2.13 | 3.66 |
| 101111650 | ID1 | 9.98 | 2.15 | 1.85 |
| 101114255 | NCK2 | 14.15 | 2.16 | 2.03 |
| 101122883 | GRAMD1A | 14.33 | 2.16 | 3.03 |
| 101118698 | EDIL3 | 27.71 | 2.19 | 0.55 |
| 101108409 | CDNF | 9.85 | 2.19 | 1.02 |
| 641305 | PI3 | 15.53 | 2.42 | 0.45 |
| 101109035 | LOC101109035 | 19.37 | 2.47 | 1.90 |
| 101119600 | TAGLN3 | 23.75 | 2.57 | 0.17 |
| 101107611 | NBEA | 11.18 | 2.57 | 1.29 |
| 101115012 | ASS1 | 106.03 | 2.63 | 1.87 |
| 101121716 | SEMA3B | 13.29 | 2.69 | 2.44 |
| 101103739 | TCAP | 61.96 | 2.70 | 0.58 |
| 101115830 | CLMN | 15.16 | 2.74 | 3.20 |
| 101111874 | KIAA1211L | 19.50 | 2.75 | 4.88 |
| 101103276 | CTPS1 | 15.21 | 2.90 | 2.54 |
| 101123341 | SH3BGR | 16.00 | 3.14 | 2.73 |
| 105604619 | FLYWCH1 | 13.07 | 3.16 | 2.41 |
| 101116803 | FMNL2 | 18.84 | 3.22 | 2.91 |
| 100171395 | RGN | 40.94 | 3.56 | 2.60 |
| 101108515 | ELOVL7 | 59.74 | 3.63 | 2.20 |
| 101117255 | JPH1 | 16.15 | 3.68 | 1.84 |
| 101116471 | PROM2 | 560.28 | 3.70 | 1.12 |
| 101118332 | PLAT | 21.04 | 3.80 | 3.99 |
| 101104335 | HSPB6 | 19.11 | 3.83 | 3.23 |
| 101102225 | VAMP5 | 19.23 | 4.27 | 4.39 |
| 101114114 | RRAGD | 19.29 | 4.30 | 1.63 |
| 101106245 | LOC101106245 | 20.63 | 4.60 | 2.94 |
| 101110626 | PFKFB4 | 21.44 | 4.65 | 1.57 |
| 101110855 | LOC101110855 | 106.49 | 4.94 | 7.61 |
| 101112452 | NEFM | 22.55 | 4.98 | 0.73 |
| 101106480 | PLA1A | 51.57 | 5.05 | 8.93 |
| 101101968 | CAPG | 125.07 | 5.17 | 13.19 |
| 101102875 | GALNT3 | 31.18 | 5.22 | 4.66 |
| 101115963 | PLK5 | 21.95 | 5.49 | 0.49 |
| 101119711 | CLEC12B | 71.57 | 5.62 | 0.03 |
| 101111442 | NT5DC3 | 26.46 | 5.65 | 2.06 |
| 101119095 | AFAP1L2 | 37.01 | 6.30 | 3.79 |
| 101123070 | TMEM120B | 39.57 | 6.71 | 5.59 |
| 101103631 | LOC101103631 | 35.04 | 6.86 | 2.37 |
| 101108802 | GUSB | 37.43 | 6.95 | 2.21 |
| 101108630 | PGM5 | 40.30 | 6.99 | 9.68 |
| 101120879 | HSPA12A | 28.85 | 7.12 | 5.06 |
| 101121924 | FOXRED2 | 41.22 | 8.23 | 1.72 |
| 101118975 | SMOX | 43.47 | 9.73 | 8.42 |
| 101104144 | IMPDH1 | 57.86 | 10.07 | 4.50 |
| 101105541 | ECM1 | 167.20 | 10.09 | 2.33 |
| 101114590 | TSC22D3 | 104.93 | 10.38 | 24.32 |
| 101120208 | S100A10 | 121.75 | 10.90 | 23.41 |
| 101103892 | SMIM3 | 85.21 | 12.10 | 12.92 |
| 101107799 | CPQ | 114.73 | 12.55 | 27.32 |
| 101107327 | SRGN | 169.74 | 13.92 | 28.92 |
| 101118266 | FGL2 | 88.48 | 15.02 | 8.41 |
| 101115996 | SLC44A4 | 122.11 | 15.20 | 28.82 |
| 101118487 | CTH | 90.12 | 15.74 | 6.18 |
| 101117541 | SLC29A1 | 87.06 | 16.42 | 12.68 |
| 101103365 | PLSCR4 | 79.34 | 17.48 | 7.25 |
| 101103077 | PPP1R1B | 134.59 | 18.80 | 14.89 |
| 100127212 | ST3GAL4 | 115.34 | 19.76 | 3.09 |
| 100462650 | SLC26A3 | 91.69 | 21.21 | 7.73 |
| 101111308 | WFDC2 | 729.78 | 22.03 | 111.08 |
| 443517 | CNN1 | 103.58 | 24.83 | 21.10 |
| 100170324 | DPP4 | 189.96 | 27.02 | 2.23 |
| 101105400 | HSD17B6 | 280.48 | 28.98 | 8.27 |
| 101111148 | FAM173A | 115.38 | 31.85 | 33.16 |
| 101114256 | ACTG2 | 313.32 | 47.61 | 75.67 |
| 101111615 | S100A14 | 433.90 | 59.75 | 29.22 |
| 101121663 | PRPS2 | 681.76 | 62.13 | 14.59 |
| 101119536 | SMARCA1 | 550.54 | 104.69 | 37.34 |
| 101108954 | PEBP1 | 667.69 | 135.47 | 129.81 |
| 101113149 | GPX4 | 882.40 | 196.62 | 142.73 |
| 101118551 | PLD3 | 2071.02 | 446.66 | 88.76 |
| 106990163 | LOC106990163 | 11918.51 | 770.66 | 21.82 |
