# Supplementary material for: Study on the region-specific expression of epididymis mRNA in the rams
Source: PLoS One. 2021 Jan 25;16(1):e0245933. doi: 10.1371/journal.pone.0245933 (PMC7833257; doi:10.1371/journal.pone.0245933)
Supplement: S13 Table — (DOCX) [file pone.0245933.s017.docx]

# S13 Table. Summary of SEGs annotated to ion metabolism in different regions of epididymis

| **Region** | **Genes** |
| --- | --- |
| Caput | LTB4R2, FRMPD1, ITGA10, ADCY8, HPD, RAB42, AK7, MT3, THBS4, LIPH, GNA15, RSG1, CYP2E1, SH3RF3, CAPSL, FSTL4, TMC8, PLCL1, TEKT1, RSPH4A, ROPN1, LOC101119853, PCDH10, ATP6V1B1, SLC9A2, GABRA2, SLC34A1, SLC13A2, SLCO2A1, CDH17, MS4A2, RNF144A, S1PR3, RIMS3, PDE4B |
| Corpus | SLC1A3, GNAL, ZACN, CADPS, LOC101110202, CALB1, ABCC12, ZSCAN30, SYN3, KIF5A, PRRG3 |
| Cauda | NOS2, SLC5A1, KIT, OSBP2, SCEL, ACE2, SHISA6, DIRAS2, TUB, ROR1, STMN2, 3-Sep, SLC16A6, SLC30A2, MMP17, DEGS2, GLRA3, TCHHL1, LOC101120969, CKMT2, STAC3, LOC106991088 |
